# AtomAccess: A Predictive Tool for Molecular Design and Its Application to the Targeted Synthesis of Dysprosium Single-Molecule Magnets

**DOI:** 10.1021/jacs.3c08841

**Published:** 2023-10-05

**Authors:** Gemma K. Gransbury, Sophie C. Corner, Jon G. C. Kragskow, Peter Evans, Hing Man Yeung, William J. A. Blackmore, George F. S. Whitehead, Iñigo J. Vitorica-Yrezabal, Meagan S. Oakley, Nicholas F. Chilton, David P. Mills

**Affiliations:** Department of Chemistry, The University of Manchester, Oxford Road, Manchester M13 9PL, U.K.

## Abstract

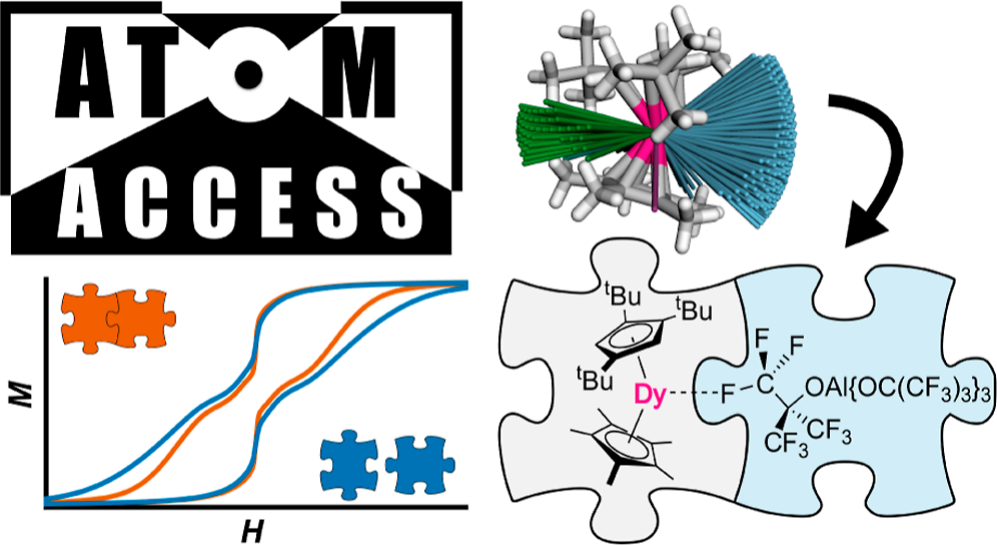

Isolated dysprosocenium cations, [Dy(Cp^R^)_2_]^+^ (Cp^R^ = substituted cyclopentadienyl), have recently been shown to exhibit superior single-molecule magnet (SMM) properties over closely related complexes with equatorially bound ligands. However, gauging the crossover point at which the Cp^R^ substituents are large enough to prevent equatorial ligand binding, but small enough to approach the metal closely and generate strong crystal field splitting has required laborious synthetic optimization. We therefore created the computer program AtomAccess to predict the accessibility of a metal binding site and its ability to accommodate additional ligands. Here, we apply AtomAccess to identify the crossover point for equatorial coordination in [Dy(Cp^R^)_2_]^+^ cations in silico and hence predict a cation that is at the cusp of stability without equatorial interactions, viz., [Dy(Cp^ttt^)(Cp*)]^+^ (Cp^ttt^ = C_5_H_2_^*t*^Bu_3_-1,2,4, Cp* = C_5_Me_5_). Upon synthesizing this cation, we found that it crystallizes as either a contact ion-pair, [Dy(Cp^ttt^)(Cp*){Al[OC(CF_3_)_3_]_4_-κ-F}], or separated ion-pair polymorph, [Dy(Cp^ttt^)(Cp*)][Al{OC(CF_3_)_3_}_4_]·C_6_H_6_. Upon characterizing these complexes, together with their precursors, yttrium and yttrium-doped analogues, we find that the contact ion-pair shows inferior SMM properties to the separated ion-pair, as expected, due to faster Raman and quantum tunneling of magnetization relaxation processes, while the Orbach region is relatively unaffected. The experimental verification of the predicted crossover point for equatorial coordination in this work tests the limitations of the use of AtomAccess as a predictive tool and also indicates that the application of this type of program shows considerable potential to boost efficiency in exploratory synthetic chemistry.

## Introduction

A limiting factor for the application of single-molecule magnets (SMMs) in high-density data storage is the requirement for expensive liquid helium cooling for them to retain magnetic information.^1−3^ For the last 2 decades, lanthanide (Ln) complexes with highly axial ligand fields have been frequently targeted, as these can provide the highest effective barriers to magnetic reversal (*U*_eff_) by stabilizing the most magnetic and destabilizing the least magnetic *m*_*J*_ states of selected Ln ions [e.g., Dy(III) and Tb(III)] along the molecular anisotropy axis; conversely, other heavy Ln ions such as Er(III) and Yb(III) require equatorial ligand fields to give high *U*_eff_ values.^4−13^ Isolated dysprosocenium cations, [Dy(Cp^R^)_2_]^+^ (Cp^R^ = substituted cyclopentadienyl), and related derivatives, have recently provided SMMs with 100 s magnetic blocking temperatures (*T*_100_) that are tantalizingly close to what can be achieved with cheaper liquid nitrogen cooling (77 K).^14−24^ The remarkable SMM properties of dysprosocenium cations arise from a combination of their highly axial ligand fields and molecular rigidity imposed by the multihapto π-aromatic carbocyclic rings, which maximize magnetic anisotropy and inhibit molecular vibrations that promote magnetic relaxation.^1−3,5,25,26^

The size of the Cp^R^ ligands, dictated by the substituents, determines the axiality of the dysprosocenium cations. The largest *U*_eff_ values are obtained for [Dy(Cp^R^)_2_]^+^ cations when the Cp^R^ ligands are close to the Dy(III) ion and the Cp^R^_centroid_···Dy···Cp^R^_centroid_ angle approaches linearity.^18,26^ Calculations on relatively small dysprosocenium cations such as [Dy(Cp*)_2_]^+^ (Cp* = C_5_Me_5_) predict very slow Orbach relaxation due to limited vibrations that are off-resonance with electronic transitions.^26^ However, due to predominantly electrostatic Ln bonding regimes,^27^ the reduced ligand steric bulk of Cp* allows for significant equatorial interactions with weakly coordinating anions (WCAs) and donor solvents; these typically disrupt axiality, promote magnetic relaxation, and reduce magnetic hysteresis temperatures (*T*_H_) compared to isolated dysprosocenium cations (which have reached up to 80 K).^18,28−34^ As the multistep syntheses of air- and moisture-sensitive target molecules can be arduous, we envisaged that in silico screening could provide a more efficient approach to identifying isolable dysprosocenium cations in advance of laboratory work. Data sets and programs that can be used to make systematic predictions of the effect of ligand steric bulk on metal coordination spheres include the Tolman cone angle,^35^ Solid-G,^36^ and (minimum) percent buried volume,% *V*_bur(min)_;^37,38^ however, these approaches do not consider if ligands or substituents may act in concert to prevent coordination.

Here, we present the ray-tracing program AtomAccess, which calculates the sizes of accessible coordination sites in a compound (as a percentage solid angle); AtomAccess has recently been used to rationalize the effects of steric hindrance on selectivity in the reactions of aminium radicals.^39^ In this work, we have used AtomAccess to survey a crystallographic database to benchmark coordination site size and then used AtomAccess on simulated molecular conformations of potential target molecules to predict the smallest isolable dysprosocenium cations, which should exhibit the most favorable SMM properties (see above). These analyses indicated that the intermediate-sized dysprosocenium cation [Dy(Cp^ttt^)(Cp*)]^+^ (Cp^ttt^ = C_5_H_2_^*t*^Bu_3_-1,2,4) prefers a single equatorial coordination site but is right at the cusp of excluding equatorial interactions. We envisaged that the attempted synthesis of this cation could provide a proof-of-concept of the AtomAccess code and test the limitations of its predictive power. Hence, we then tested these hypotheses experimentally by preparing and characterizing both the contact ion-pair (CIP) [Dy(Cp^ttt^)(Cp*){Al[OC(CF_3_)_3_]_4_-κ-F}], where the WCA {Al[OC(CF_3_)_3_]_4_-κ-F} coordinates equatorially to the Dy(III) center by a monodentate interaction through a single fluorine atom, and the separated ion-pair (SIP) polymorph [Dy(Cp^ttt^)(Cp*)][Al{OC(CF_3_)_3_}_4_]·C_6_H_6_, which does not exhibit a significant transverse interaction with the WCA. This unprecedented example of the [Al{OC(CF_3_)_3_}_4_]^−^ anion coordinating to Dy provides an opportunity to examine the effect of very weak equatorial interactions on SMM properties. These complexes, together with their precursors and yttrium and doped analogues, have been characterized by NMR and ATR-IR spectroscopy, elemental analysis, powder and single crystal X-ray diffraction (PXRD/SCXRD), and SQUID magnetometry. Returning to the hypothesis that we could obtain a smaller dysprosocenium complex and possibly identify an improved SMM, and variable-temperature and -field magnetic measurements reveal that both the CIP and SIP show high-temperature SMM behavior that is typical of dysprosocenium cations, with the former complex showing inferior SMM properties due to faster Raman relaxation caused by the equatorially bound WCA. We find that the Orbach relaxation process and the axiality of the complexes are largely unaffected by the coordination of the WCA. Density functional theory (DFT) and complete active space self-consistent field spin–orbit (CASSCF-SO) calculations are respectively performed to characterize the Dy···F interaction and disentangle the influence of WCA binding on SMM behavior.

## Results

### AtomAccess Algorithm

**Figure 1:**
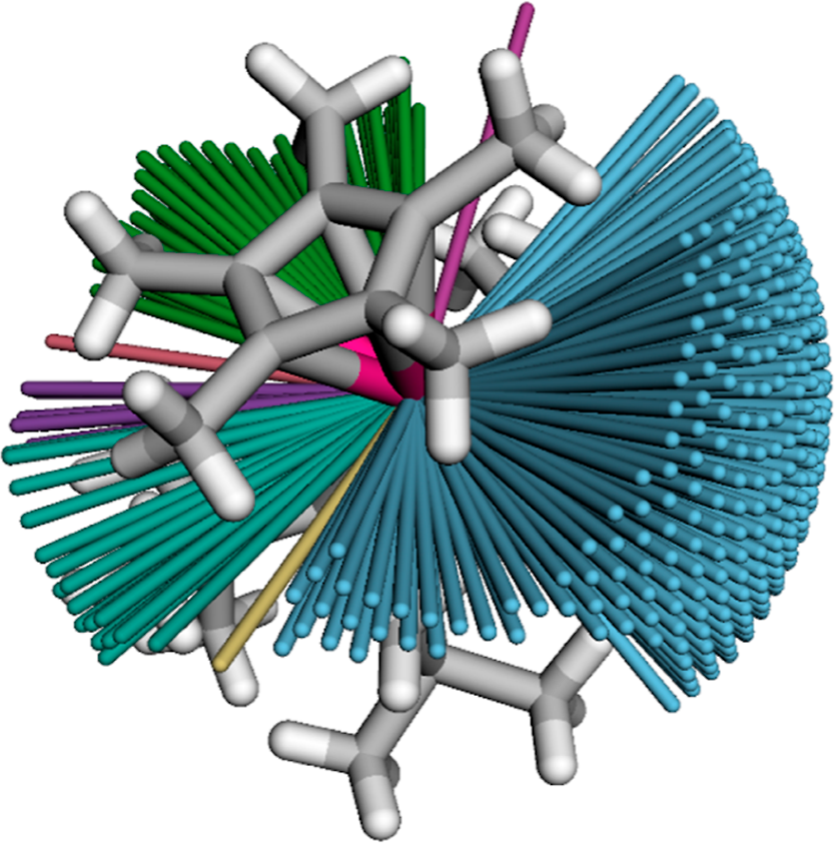
Ray tracing of accessible sites in the [Dy(Cp^ttt^)(Cp*)]^+^ cation with AtomAccess using atomic coordinates from a SCXRD data set obtained herein.^45^ Dy: pink, C: gray, H: white; largest cluster: light blue (12.2%); minor clusters: green (2.2%), teal (0.7%), purple (0.3%), peach, yellow, and magenta (0.04%).

AtomAccess is written in Python (≥v3.9), is open source^40^ and licensed under GNU GPLv3, and is available on the PyPI package repository;^41^ the program is scriptable and useable on both command-line and web interfaces (Figure S3),^42,43^ where the latter allows for interactive visualization (Figure 1). AtomAccess quantifies the accessibility of an atom in a molecule as the size of the largest binding site that is identified (light blue cluster in Figure 1); binding sites are calculated as the largest continuous fraction of solid angle that is not obscured by surrounding atoms. The selected atom is treated as a point source of light, from which a collection of rays emanate on a Zaremba–Conroy–Wolfsberg (ZCW) angular grid (with density index ρ as described by Levitt^44^), evenly sampling points on the surface of a sphere. Intersections of the ray vectors with the surrounding atoms (spheres defined with van der Waals atomic radii^43^) are calculated up to a radial cutoff (*r*_max_) from the central atom (see Supporting Information for details); here, we set *r*_max_ to encompass the entire molecule. Unblocked rays represent points on the surface of a sphere of radius *r*_max_ from the central atom and can be clustered with a single-linkage agglomerative clustering algorithm in Scikit-learn (see Supporting Information for details),^44^ such that adjacent points are clustered together (Figure 1).

The size of a potential binding site in AtomAccess is calculated as the number of unblocked rays in a cluster as a percentage of the total number of rays, which for the ZCW angular grid is equivalent to the percentage of solid angle (% SA) subtended by that cluster. Highly sterically hindered complexes have small largest clusters. AtomAccess reports the total percentage of unblocked rays (comparable to 100-*G* in Solid-G, which treats the central atom as a point source of light; where the amount of light that is blocked by atoms of ligand frameworks is expressed as a percentage value, *G*),^36^ along with the sizes of all clusters. In our experience, the size of the largest cluster is a good metric for assessing the accessibility of a metal in a complex when only steric considerations are taken into account; AtomAccess is therefore ideally suited for Ln complexes with predominantly ionic bonding regimes. We find that angular grid densities 9 ≤ ρ ≤ 11 are optimal as smaller values yield inaccurate sizes of the binding sites and larger values result in merged clusters through unphysically small gaps (Figure S4); herein, we use ρ = 10 (Table S1). For compounds with low steric hindrance, the % SA of the largest cluster approaches the total percentage of unblocked rays (Figure S5).

### AtomAccess Calculations

**Figure 2:**
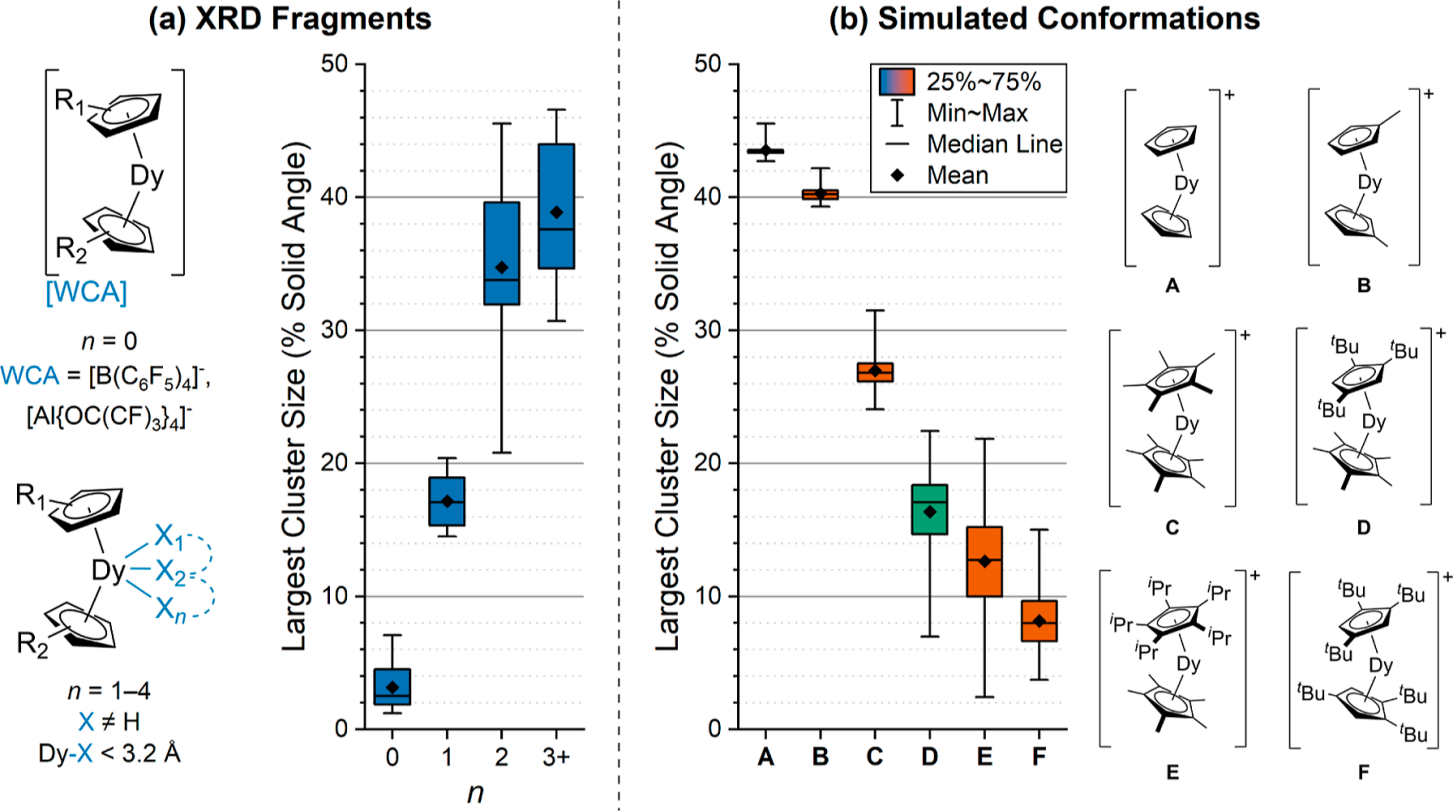
Distributions of the largest cluster sizes for dysprosium bis-cyclopentadienyl and phospholyl fragments determined by AtomAccess. (a) Fragments (black) taken from the Cambridge Structural Database and grouped by *n*, the number of ancillary ligand non-H atoms within 3.2 Å of Dy. Cp^R1^, Cp^R2^ include C_5_H_5_, C_5_H_4_Me, C_5_H_4_^i^Pr, C_5_H_4_^*t*^Bu, C_5_H_4_SiMe_3_, C_5_H_4_SiMe_2_^*t*^Bu, indenyl, C_5_H_3_^*t*^Bu_2_-1,3, C_5_H_3_(SiMe_3_)_2_-1,3, C_5_H_2_Ph-1-(*p*-tolyl)_2_-3,4, C_5_H_2_(*p*-tolyl)_3_-1,2,4, C_5_H_2_^*t*^Bu_3_-1,2,4 (Cp^ttt^), C_5_H_2_(SiMe_3_)_3_-1,2,4, C_5_HMe_4_, C_5_H^i^Pr_4_, C_5_Me_5_ (Cp*), C_5_Me^i^Pr_4_, C_5_Et^i^Pr_4_, C_5_^i^Pr_5_, C_4_PMe_2_-2,3-^t^Bu_2_-1,4, C_4_PMe_2_-2,3-(SiMe_3_)_2_-1,4, and C_4_PEt_4_. (b) Simulated conformations of fragments **A**–**F** generated in OpenBabel^48^ using standardized initial {Dy(C_5_)_2_}^+^ coordinates (Dy···Cp_centroid_ = 2.375 Å).

We sought to explore the utility of AtomAccess in identifying synthetically isolable, low-coordinate Ln complexes. Although this first proof-of-concept study could have been applied to any number of research problems, we resolved to determine if the smallest dysprosocenium cation that AtomAccess predicted could be isolated, with the aim of synthesizing an SMM with a high *U*_eff_ value (see above). To achieve this end, we used AtomAccess to determine the size of equatorial binding sites in 290 dysprosium metallocene and derivatized metallocene fragments (Table S2), using atomic coordinates extracted from the Cambridge Structural Database (CSD; see Supporting Information for details),^46^ to correlate the size of vacant coordination sites with observed equatorial ligand coordination. These data sets included the isolated dysprosocenium, bis-phospholyl, and mixed phospholyl/cyclopentadienyl cations [Dy(Cp^ttt^)_2_]^+^, [Dy(C_4_PMe_2_^*t*^Bu_2_)_2_]^+^, [Dy(C_5_R^i^Pr_4_)_2_]^+^ (R = H, Me, Et, ^i^Pr), [Dy(C_5_^i^Pr_5_)(Cp*)]^+^, and [Dy(C_5_^i^Pr_5_)(C_4_PEt_4_)]^+^; we also included {Dy(Cp^R^)_2_}^+^, {Dy(Cp^R^)(C_4_P^R^)}^+^, and {Dy(C_4_P^R^)_2_}^+^ fragments (C_4_P^R^ = substituted phospholyl) obtained from parent crystal structures, with ancillary ligand(s), counterions, cocrystallized solvent, and/or other metal complex fragments removed (Figure 2a). A unique set of atomic coordinates was generated for each crystallographically unique Dy atom as well as for each disordered component in a structure. The fragments were grouped by the number of equatorial interactions with Dy in the parent structure, *n*, taken as the number of non-hydrogen atoms within 3.2 Å of Dy (excluding Cp^R^ or C_4_P^R^ atoms).

Figure 2a shows how AtomAccess can identify the propensity of a dysprosocenium fragment to allow for equatorial coordination in a particular geometry. Isolated dysprosocenium cations have a maximum cluster size of 7.1% SA and a mean largest cluster size of 3.2% SA, reflecting their highly sterically hindered geometries. Fragments with a single equatorial coordination site, *n* = 1, have binding sites of 14.5–20.4% SA, enabling the coordination of a single halide (Cl, Br, I) or borohydride ligand; these compounds are often used as precursors to isolated dysprosocenium cations.^14−19,22,47^ Dysprosocenium fragments that allow for the coordination of multiple ligands or multidentate ligands (*n* ≥ 2) have a minimum largest cluster of 20.8% SA. Hence, the threshold for single equatorial interactions from this data set is between 7.1 and 14.5% SA, and ∼20.5% SA is the threshold for multiple equatorial interactions.

We wanted to identify new {Dy(Cp^R^)_2_}^+^ synthetic targets with small substituents, in order to allow for a close approach of the Cp^R^ ligands to increase the axial crystal field and improve SMM performance.^26^ The workflow we adopted was as follows: (1) {Dy(Cp^R^)_2_}^+^ fragments were built from a standardized {Dy(C_5_)_2_}^+^ framework (Table S4); (2) diverse conformational isomers of {Dy(Cp^R^)_2_}^+^ were generated with a genetic algorithm in OpenBabel;^48^ and (3) the size of the largest cluster for each conformer was determined with AtomAccess (see Supporting Information for further details). AtomAccess is scriptable and can be interfaced with any conformer generation algorithm. Our strategy was driven by the aims to generate a conformer set that was unbiased by energies (which may be overcome by additional coordination), to rapidly screen target molecules, and to test if a simple and computationally cheap approach could deliver the {Dy(Cp^R^)_2_}^+^ fragments required for coordination prediction with AtomAccess. Starting from a standardized {Dy(C_5_)_2_}^+^ framework allows for the facile construction of derivatives, while the method chosen for conformer production is considerably less computationally demanding than performing DFT geometry optimizations or molecular dynamics simulations. Although DFT-generated conformers would be a more thorough approach, this would not meet our rapid-screening aim, and hence, we made a pragmatic compromise. The strategy used is limited in that it cannot predict the degree of flexibility of the ancillary ligands to distort and move to accommodate additional ligand interactions.

Our chosen method was benchmarked with common {Dy(Cp^R^)_2_}^+^ fragments **A**–**C** (Figure 2b) that consistently exhibit equatorial interactions^28−34^ and known isolated dysprosocenium cations (**E**, **F**). Using AtomAccess and our conformer generation methodology, we see that fragments **A**–**C** reasonably reproduce the largest cluster distributions from XRD (Table S5) and correctly predict two or more equatorial interactions (largest clusters, ≥24.1% SA). The distributions obtained for **E** and **F** confirm that a single equatorial coordination site can be accommodated (i.e., conformations exist with >14.5% SA), but also that **E** and **F** can rearrange to shield the Dy(III) ion from equatorial coordination (i.e., conformations exist with clusters ≤2.4 and ≤3.7% SA, respectively).

The heteroleptic ligand system in **E** allows for short Dy···Cp^R^ distances and an axial ligand field,^18^ but we sought to decrease the Dy···Cp^R^ distances further by substitution of the bulky C_5_^i^Pr_5_ ligand with the smaller Cp^ttt^ ligand. We therefore investigated {Dy(Cp^ttt^)(Cp*)}^+^ (**D**) as a potential synthetic target by using the process described above. Fragment **D** is less sterically hindered than **E** and **F**, but it is not as accessible as **C** (Figure 2b, Table S5). Comparing AtomAccess results on the generated conformers of **D** to the thresholds derived from XRD data, the mean (16.4% SA) and central 50% of the distribution (14.7–18.4% SA) indicate a preference for a single equatorial coordination, while the most open conformation (22.4% SA) could accommodate two atoms equatorially. The most sterically hindered conformation of **D** can restrict all clusters to ≤7.0% SA, and thus, this cation should be on the cusp of excluding equatorial interactions. We therefore resolved to attempt the synthesis of the [Dy(Cp^ttt^)(Cp*)]^+^ cation to test the validity of the AtomAccess code as a predictive computational tool, to explore the limit of equatorial interactions, and to target the isolation of a dysprosocenium cation that could potentially exhibit a stronger axial ligand field and hence improved SMM properties over previously reported examples.

### Synthesis

**Scheme 1:**
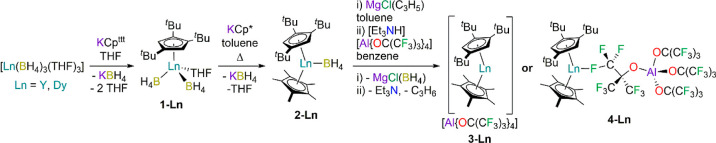
Synthesis of **3-Ln** and **4-Ln** via **1-Ln** and **2-Ln**

Axial complexes featuring {Ln(Cp^ttt^)(Cp*)} motifs were prepared by multistep procedures that adapted a combination of literature protocols to give complexes **1–4-Ln** (Scheme 1).^18,19,47,49−53^ The separate salt metathesis reactions of the Ln borohydride solvates [Ln(BH_4_)_3_(THF)_3_] (Ln = Y, Dy) with KCp^ttt^ in THF gave the monoring complexes [Ln(Cp^ttt^)(BH_4_)_2_(THF)] (**1-Ln**; Ln = Y, Dy); the syntheses of **1-Ln** (Ln = Dy, Y, Lu) have also recently been reported by Price et al.^54^ Complexes **1-Ln** were isolated and reacted with KCp* in toluene at reflux to afford the heteroleptic bis-Cp^R^ Ln complexes [Ln(Cp^ttt^)(Cp*)(BH_4_)] (**2-Ln**). Complexes **1–2-Ln** were all obtained in good yields (65–77%), following workup and recrystallization from *n*-hexane. We initially attempted to use a perfluorotetraphenylborate WCA, as used with the majority of previously isolated dysprosocenium cations.^14−18^ However, separate small scale reactions of **2-Dy** with either [(SiEt_3_)_2_(μ-H)][B(C_6_F_5_)_4_] or [CPh_3_][B(C_6_F_5_)_4_] in benzene consistently gave the boronium complex [BH(Cp^ttt^)][BH(Cp*)][B(C_6_F_5_)_4_]_2_ (**5**), indicating that the [B(C_6_F_5_)_4_]^−^ anion is not well suited for isolation of the target SIP or CIP {Dy(Cp^ttt^)(Cp*)}^+^ complexes. Considering the reduced steric bulk of the target [Dy(Cp^ttt^)(Cp*)]^+^ cation compared to literature dysprosocenium cations, we decided to pursue a strategy using a bulky perfluoroalkoxyaluminate that is even more weakly coordinating and has not previously been shown to bind to rare earth metals in the solid state.^55^

The separate reactions of **2-Ln** with allylmagnesium chloride in toluene gave crude products formulated as “[Ln(Cp^ttt^)(Cp*)(C_3_H_5_)]”, which were separated from MgX_2_ (X = Cl, BH_4_) byproducts by trituration and filtration and dried under vacuum. The allyl intermediates were treated with [NEt_3_H][Al{OC(CF_3_)_3_}_4_] in benzene, and following workup, crude powders tentatively assigned as “[{Ln(Cp^ttt^)(Cp*)}{Al[OC(CF_3_)_3_]_4_}]” were obtained in moderate yields (58–72% based on **2-Ln**). Recrystallization of these powders was performed from aliquots frozen in benzene, layered with *n*-hexane, and stored at 6 °C. On some occasions, crystals of the SIP complexes [Ln(Cp^ttt^)(Cp*)][Al{OC(CF_3_)_3_}_4_]·C_6_H_6_ (yields based on **2-Ln**: **3-Y·C**_**6**_**H**_**6**_: 40%, **3-Dy·C**_**6**_**H**_**6**_: 54%) were obtained, and in other recrystallization attempts, the CIP polymorphs [Ln(Cp^ttt^)(Cp*){Al[OC(CF_3_)_3_]_4_-κ-F}] (yields based on **2-Ln**: **4-Y**: 49%; **4-Dy**: 58%) formed, experimentally validating the predictions extracted from AtomAccess that the [Dy(Cp^ttt^)(Cp*)]^+^ cation is at the limit of forming transverse interactions.

As the SIP and CIP were isolated under the same recrystallization conditions on separate occasions, we attempted to purposefully target these polymorphs selectively. However, despite numerous recrystallization attempts where concentrations, solvent proportions, and temperatures were varied, we were unable to determine experimental conditions where one polymorph preferentially formed over the other; the flexibility of the geometries of coordinatively unsaturated lanthanide complexes due to the large cation size and nondirectional bonding^27^ enables several energetically equivalent metal coordination environments, offering many opportunities for polymorph formation. We found that the CIP polymorph was identified in the majority of these experiments, but we cannot provide a meaningful statistical analysis. We are confident from analysis of PXRD data that the **3-Dy·C**_**6**_**H**_**6**_ and **4-Dy** samples used to obtain the characterization data herein show high bulk phase purities (see below). During a recrystallization attempt of “[{Dy(Cp^ttt^)(Cp*)}{Al[OC(CF_3_)_3_]_4_}]” at room temperature, a small crop of crystals of [{Dy(Cp^ttt^)(Cp*)}_2_(μ-F)][Al{OC(CF_3_)_3_}_4_] (**6-Dy·C**_**6**_**H**_**14**_) were obtained. This dinuclear complex is assumed to form via the abstraction of F^–^ from the [Al{OC(CF_3_)_3_}_4_]^−^ anion; similar observations have previously been seen for this WCA.^56^ An aliquot of “[{Dy(Cp^ttt^)(Cp*)}{Al[OC(CF_3_)_3_]_4_}]” in frozen toluene layered with *n*-hexane stored at −30 °C did not give a crystalline material, and the addition of DCM to the amorphous product led to rapid decomposition; solvents that could bind strongly to the Dy(III) center were avoided, while the binding of halobenzenes will form a separate study.

### Bulk Solid-State Characterization

Elemental analysis was performed on samples of **1-Ln**, **2-Ln**, “[{Ln(Cp^ttt^)(Cp*)}{Al[OC(CF_3_)_3_]_4_}]”, **3-Ln·C**_**6**_**H**_**6**_, and **4-Ln** (see Supporting Information for full details of supporting characterization data). Powders of “[{Ln(Cp^ttt^)(Cp*)}{Al[OC(CF_3_)_3_]_4_}]” were washed multiple times with *n*-hexane and benzene, with volatiles removed in vacuo after each wash, but were found to contain trace amounts of nitrogen (<0.25% N based upon the assumed molecular weight). ATR-IR spectroscopy (Figures S11–S22) showed that isostructural Dy/Y pairs all had overlapping features and that similar absorptions were seen for “[{Ln(Cp^ttt^)(Cp*)}{Al[OC(CF_3_)_3_]_4_}]”, **3-Ln·C**_**6**_**H**_**6**_, and **4-Ln**, indicating that the bulk structural features of the SIP and CIP are similar in the solid state. The phase purities of microcrystalline batches of **3-Dy·C**_**6**_**H**_**6**_, **4-Dy,** and a doped sample of the CIP in a diamagnetic matrix (**5%Dy@4-Y**) were determined by PXRD (Figures S24–S30 and Tables S6 and S7), with Pawley refinement^57^ of **3-Dy·C**_**6**_**H**_**6**_ and **4-Dy** data generally showing good agreement with SCRXD data sets. Le Bail refinement^58^ of **5%Dy@4-Y** data indicates two CIP phases are present, with a major phase consistent with bulk **4-Dy** and a minor phase that matches the SCXRD data set for **5%Dy@4-Y**. Some unassigned peaks were present in all PXRD diffractograms; however, the low quantity and crystallinity of the contaminant phases linked to varied relative peak intensities due to the preferential orientation of crystallites (small sample size) precluded accurate identification and quantification of the minor phases observed.

### Solution NMR Spectroscopy

Multinuclear solution NMR spectroscopy was of limited use for characterization of **1-Dy** and **2-Dy** due to paramagnetic broadening, though a ^1^H NMR spectrum of **1-Dy** could be tentatively interpreted (Figure S36), and the ^1^H, ^13^C{^1^H}, and ^11^B{^1^H} NMR spectra of diamagnetic Y(III) analogues **1-Y** and **2-Y** were fully assigned (Figures S31–S35 and S38–S42). The solution magnetic susceptibilities of **1-Dy** (μ_eff_ = 10.68 μ_B_) and **2-Dy** (μ_eff_ = 10.71 μ_B_) determined by the Evans method in *d*_6_-benzene at 298 K (Figures S37 and S44)^59^ are in excellent agreement with the predicted free-ion value for Dy(III) (μ_eff_ = 10.63 μ_B_).^27^ We found that “[{Y(Cp^ttt^)(Cp*)}{Al[OC(CF_3_)_3_]_4_}]”, **3-Y·C**_**6**_**H**_**6**_, and **4-Y** do not readily dissolve in C_6_D_6_ and rapidly decompose in CD_2_Cl_2_; however, the ^1^H, ^13^C{^1^H}, ^19^F, and ^19^F{^1^H} NMR spectra of these samples dissolved in fluorobenzene all contained the expected number of signals at the chemical shifts anticipated (Figures S45–S51 and S56–S58). The ^1^H and ^13^C{^1^H} NMR spectra of a sample of “[{Y(Cp^ttt^)(Cp*)}{Al[OC(CF_3_)_3_]_4_}]” dissolved in fluorobenzene with a C_4_D_8_O insert (Figures S45 and S46) revealed the presence of ca. 0.25 equiv of residual triethylamine, which is consistent with the detection of trace amounts of nitrogen in elemental analysis experiments (see above). The ^19^F{^1^H} NMR spectra of “[{Dy(Cp^ttt^)(Cp*)}{Al[OC(CF_3_)_3_]_4_}]”, **3-Dy·C**_**6**_**H**_**6**_, and **4-Dy** (Figures S53, S55 and S60) each exhibited one signal at ca. −82 ppm for the [Al{OC(CF_3_)_3_}_4_]^−^ anion, together with a very broad, paramagnetically shifted resonance for fluorobenzene at ca. −120 ppm (υ_1/2_ =1700–2900 Hz), indicating that the solvent coordinates in solution; similar conclusions can be drawn from the broad signals arising from fluorobenzene in the ^1^H NMR spectra of these samples (Figures S52, S54, and S59).

### Single Crystal X-ray Diffraction and DFT Studies

**Figure 3:**
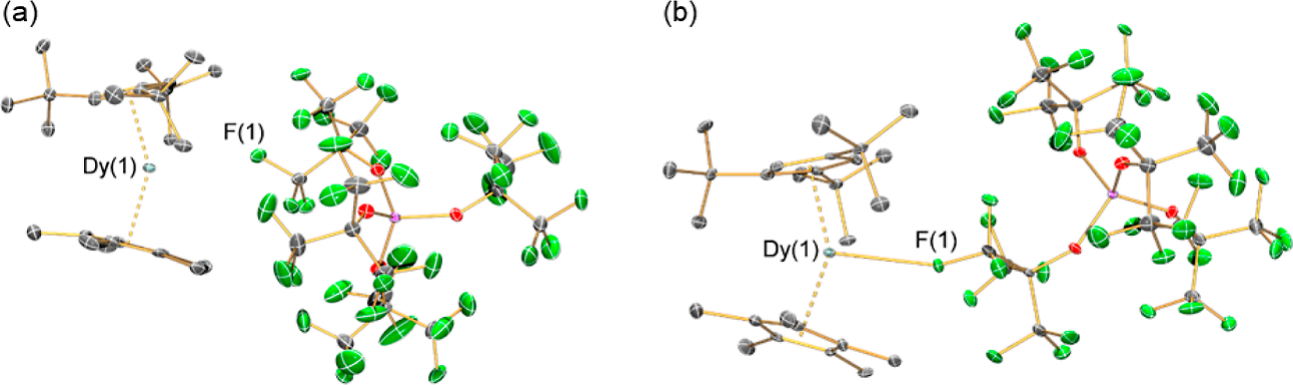
SCXRD structures of (a) SIP **3-Dy·C**_**6**_**H**_**6**_ and (b) CIP **4-Dy** with select atom labeling (Dy: cyan, C: gray, O: red, F: green, Al: purple). Displacement ellipsoids are set at 30% probability levels; hydrogen atoms and lattice solvent in **3-Dy·C**_**6**_**H**_**6**_ and **4-Dy** are omitted for clarity.

The solid-state structures of **1–4-Ln**, **5%Dy@4-Y**, **5,** and **6-Dy** were determined by SCXRD; the structures of **1-Ln** have also recently been reported by Price et al.^54^ (the SIP **3-Dy·C**_**6**_**H**_**6**_ and CIP **4-Dy** are depicted in Figure 3 and selected metrical parameters are compiled in Table 1; see Figures S64–S72 and Tables S8–S13 for all other structures and supporting crystallographic data). As **1–4-Y** are structurally analogous to Dy homologues, numerous similar complexes to **1-Ln** and **2-Ln** are known in the literature,^18,47,49−51^ and the **5%Dy@4-Y** SCXRD data set represents a *P*1̅ polymorph present only as a minor CIP phase in bulk **5%Dy@4-Y**, we limit our discussion to **3-Dy·C**_**6**_**H**_**6**_ and **4-Dy** here for brevity. The SIP **3-Dy·C**_**6**_**H**_**6**_ cocrystallizes with benzene in the *Pbca* space group, while no lattice solvent is present for the CIP **4-Dy**, which crystallizes in *P*2_1_/*c*. The shortest Dy···Dy distances are 9.3356(4) and 10.4588(10) Å for the SIP and CIP, respectively.

**Table 1:** Selected Interatomic Distances (Å) and Angles (°) for SIP **3-Dy·C**_**6**_**H**_**6**_ and CIP **4-Dy**

	**3-Dy·C**_**6**_**H**_**6**_	**4-Dy**
largest cluster size, % SA^a^	12.2	13.2
Dy(1)···Cp^ttt^_centroid_	2.297(2) Å	2.3155(5) Å
Dy(1)···Cp*_centroid_	2.314(2) Å	2.2994(4) Å
Dy(1)···F(1)/Dy(1)–F(1)	3.144(4) Å	2.812(4) Å
shortest Dy(1)···H	2.518 Å	2.622 Å
shortest Dy(1)···C	2.952(6) Å	2.982(7) Å
F(1)–C(29)	1.372(7) Å	1.384(6) Å
shortest Dy(1)···Dy(1)	9.3356(4) Å	10.4588(10) Å
Cp^ttt^_centroid_···Dy(1)···Cp*_centroid_	149.15(9)°	148.26(2)°
Cp^ttt^_centroid_···Dy(1)–F(1)	98.40(9)°	108.60(8)°
Cp*_centroid_···Dy(1)–F(1)	112.13(10)°	103.00(8)°
Dy–F(1)–C(29)	141.4(3)°	149.2(4)°

a Calculated using AtomAccess from experimentally obtained cation atomic coordinates.

The [Al{OC(CF_3_)_3_}_4_]^−^ anion does not appear to significantly coordinate to the Dy center in the SIP (shortest Dy···F distance: 3.144(4) Å), whereas it appears to weakly bind in a κ^1^-F fashion in the CIP (Dy···F: 2.812(4) Å) (sum of covalent radii for Dy and F is 2.31 Å).^60^ In both **3-Dy·C**_**6**_**H**_**6**_ and **4-Dy,** the [Al{OC(CF_3_)_3_}_4_]^−^ anion interacts with the cation through the closest F atom, resulting in an activation of the C–F bond that is greater for **4-Dy** (1.383(6) Å) than **3-Dy·C**_**6**_**H**_**6**_ (1.372(6) Å).^61^ Gas-phase DFT optimizations significantly change the geometry of both **3-Dy** and **4-Dy** (Dy replaced with Y for computational simplification), generating multiple shorter Y···F interactions and pushing the Cp^R^ rings apart, precluding a detailed analysis of the strength of the Y···F interaction relative to crystal packing forces. Single-point DFT calculations on the CIP and SIP SCXRD geometries showed only slight differences in partial Mulliken charges (Table S14); however, analysis of the electron density with quantum theory of atoms in molecules (QTAIM)^62−64^ at the bond critical point, ρ(BCP), provides insight into the strength of the interactions in comparison to other Y···F interactions (ρ(BCP)-**3-Y** = 0.002 au; ρ(BCP)-**4-Y** = 0.018 au; average ρ(BCP)-**3-Y(opt)** = 0.024 au; average ρ(BCP)-**4-Y(opt)** = 0.025 au; Table S15). These data support a Y···F interaction in both **3-Y** and **4-Y**, which is vanishingly small in the former complex and shorter and stronger in the latter, where it is of the same order of magnitude as the optimized Y···F interactions (2.18–2.44 Å).

The metrical parameters of the {Dy(Cp^ttt^)(Cp*)}^+^ core of **4-Dy** (Dy···Cp^ttt^_centroid_: 2.315(3) Å; Dy···Cp*_centroid_: 2.300(4) Å; Cp^ttt^_centroid_···Dy···Cp*_centroid_: 148.21(11)°) are not changed significantly by equatorial anion binding c.f. **3-Dy·C**_**6**_**H**_**6**_ (Dy···Cp^ttt^_centroid_: 2.297(2) Å; Dy···Cp*_centroid_: 2.314(2) Å; Cp^ttt^_centroid_···Dy···Cp*_centroid_: 149.15(9)°). We used AtomAccess to examine the SCXRD structures, and found that the largest cluster size for the {Dy(Cp^ttt^)(Cp*)}^+^ fragment is 12.2% SA for **3-Dy·C**_**6**_**H**_**6**_ and 13.2% SA for **4-Dy**; these values lie within the expected range for one or no equatorial ligands deduced from our CSD search. Cation–anion interactions will always persist, even for the most WCAs;^55^ thus the threshold for equatorial coordination as determined by AtomAccess is a continuum rather than a discrete value. For the {Dy(Cp^ttt^)(Cp*)}^+^ cation, the SA of **4-Dy** corresponds to a weak but distinct Dy···F equatorial interaction, and the SA of **3-Dy·C**_**6**_**H**_**6**_ results in a significantly longer Dy···F distance.

### Magnetism

Solid-state magnetic data were obtained on **3-Dy·C**_**6**_**H**_**6**_, **4-Dy,** and “[{Dy(Cp^ttt^)(Cp*)}{Al[OC(CF_3_)_3_]_4_}]” in order to determine their SMM properties. We were not able to ensure the purity of the amorphous sample, so we do not discuss these data in detail, but they are consistently in accord with a heterogeneous mixture of CIP and SIP forms; we note that there are no structural data available to confirm this hypothesis. The magnetic susceptibility-temperature products (χ*T*) measured in a 0.1 T dc field at 300 K are 12.75 cm^3^ K mol^–1^ for **3-Dy·C**_**6**_**H**_**6**_ and 13.13 cm^3^ K mol^–1^ for **4-Dy**, with the deviation from the Dy(III) free-ion value (^6^H_15/2_, χ*T* = 14.17 cm^3^ K mol^–1^)^27^ expected due to significant splitting of the *m*_*J*_ states by the crystal field. The χ*T* values decrease steadily with temperature until ca. 25 K (11.56 cm^3^ K mol^–1^ for **3-Dy·C**_**6**_**H**_**6**_ and 12.21 cm^3^ K mol^–1^ for **4-Dy**) due to thermal depopulation of excited *m*_*J*_ states, while below this temperature, there is a sharp decrease in χ*T* associated with the onset of slow magnetic dynamics (Figures S73 and S74).

**Figure 4:**
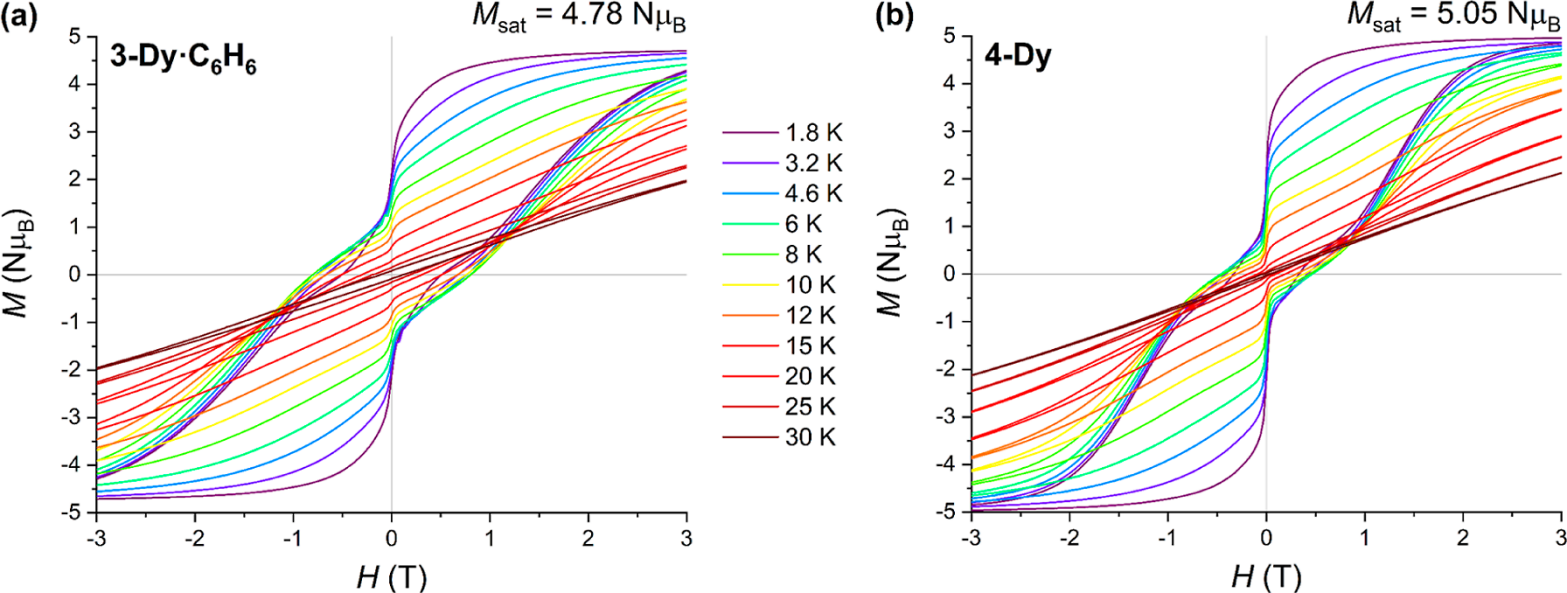
Temperature dependence of magnetic hysteresis loops of (a) **3-Dy·C**_**6**_**H**_**6**_ and (b) **4-Dy** at 1.8–30 K. Sweep rate is 22 Oe s^–1^.

Zero-field-cooled (ZFC) and field-cooled (FC) dc magnetic susceptibility measurements showed an irreversible point (*T*_irrev_) of 40 K for **3-Dy·C**_**6**_**H**_**6**_ and 28 K for **4-Dy**, where χ diverges by >1% depending on the history of the sample (Figures S77 and S79). The ZFC peak (*T*_peak_) values are 15 K (**3-Dy·C**_**6**_**H**_**6**_) and 18 K (**4-Dy**); we note that the initial magnetization is high due to an imperfect zero-field condition^65^ and a second minor fast relaxation channel (see below). Magnetization vs applied dc field measurements were performed over a range of temperatures with a sweep rate of 22 Oe s^–1^, giving butterfly-shaped open hysteresis loops at low temperatures (Figure 4) that are typical of Ln SMMs; the rapid loss of magnetization at zero field is due to quantum tunneling of magnetization (QTM).^66^ The magnitude of the QTM step is greater for **4-Dy** than for **3-Dy·C**_**6**_**H**_**6**_ (Figure S83) and the hysteresis loops close at lower temperatures, with *T*_H_ = 52 K for **3-Dy·C**_**6**_**H**_**6**_ and 36 K for **4-Dy**. Magnetization saturation values of 4.78 N μ_B_ for **3-Dy·C**_**6**_**H**_**6**_ and 5.05 N μ_B_ for **4-Dy** measured at 2 K and 7 T are close to the expected value of 5.00 N μ_B_ for a pure *m*_*J*_ = ±15/2 ground state (Figures S85 and S87).^65^

The dynamic magnetic behavior of **3-Dy·C**_**6**_**H**_**6**_ and **4-Dy** were probed along with the amorphous “[{Dy(Cp^ttt^)(Cp*)}{Al[OC(CF_3_)_3_]_4_}]”, the doped CIP **5%Dy@4-Y**, and **4-Dy** in benzene (biphasic mixture; dissolution in halobenzene solvents results in solvent coordination). Ac susceptibility measurements (0.1–1000 Hz) in zero dc field revealed maxima in the out-of-phase susceptibilities (χ_M_″) between 66 and 102 K for all samples, indicative of slow magnetic relaxation. A minor second relaxation channel was identified for **3-Dy·C**_**6**_**H**_**6**_ and **4-Dy** as a broad ill-defined peak at low temperatures (≤45 K), while a substantial second relaxing component was identified for **4-Dy** in benzene as a high-frequency shoulder in χ_M_″ (Supporting Information Section S9.2). Complexes **3-Dy·C**_**6**_**H**_**6**_ and **4-Dy** were further characterized using the “waveform” method of Hilgar et al.,^67^ which employs a square wave magnetic field (0.18–28 mHz, ±8 Oe) around zero dc field. In- and out-of-phase susceptibilities were then extracted using CC-FIT2^68,69^ via a discrete Fourier transform (Supporting Information Section S9.3).^67^ Ac and waveform data were fit to the generalized Debye model or double generalized Debye model in CC-FIT2 to extract relaxation times (τ_debye_) and distributions.^68,69^ Magnetic relaxation at lower temperatures were characterized with dc magnetization decay measurements. Data were fit to a stretched exponential function, or a sum of two stretched exponential functions as required (Supporting Information Section S9.4). Some of us have recently shown that *e*^⟨lnτ⟩^ extracted from stretched exponential fits to magnetization decays is the most accurate way of characterizing magnetic relaxation on long time scales, providing the best agreement with τ_debye_ (Supporting Information Section S9.5).^69,70^ Estimated standard deviations for ac, waveform, and dc data were derived from logarithmic relaxation times.^69,70^

**Figure 5:**
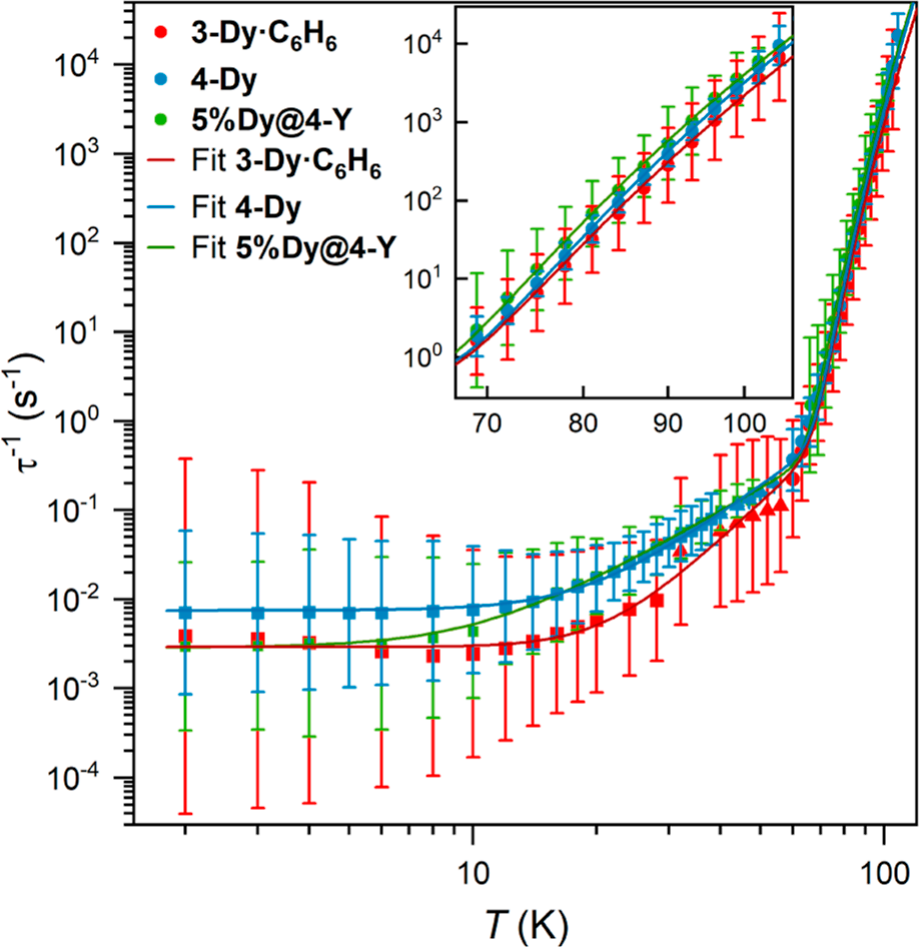
Temperature-dependent magnetic relaxation rates of SIP **3-Dy·C**_**6**_**H**_**6**_ (red), CIP **4-Dy** (blue), and doped CIP **5%Dy@4-Y** (green). Inset shows the magnified Orbach region. Relaxation rates are derived from ac susceptibility data (circles), waveform data (triangles), and dc magnetization decays (squares). Error bars represent one standard deviation of the logarithmic distribution of relaxation rates. Lines of best fit are given by τ^–1^ = 10^–*Q*^ + 10^*R*^*T*^*n*^ + 10^–A^ exp[−U_eff_/*k*_B_*T*]; n.b. τ_QTM_ = 10^Q^, *C* = 10^*R*^ and τ_0_ = 10^*A*^.

The temperature dependence of relaxation rates for **3-Dy·C**_**6**_**H**_**6**_, **4-Dy,** and **5%Dy@4-Dy** were fit to a sum of Orbach (exponential), Raman (power law), and QTM (constant) processes (τ^–1^ = 10^–*Q*^ + 10^*R*^*T*^*n*^ + 10^–*A*^ exp[−*U*_eff_/*k*_B_*T*], τ_QTM_ = 10^*Q*^, C = 10^*R*^ and τ_0_ = 10^*A*^), and key SMM parameters are reported with their estimated standard deviations (ESD; Figure 5, individual plots in Figures S154–S156, Table 2). The effective barrier to magnetic reversal (*U*_eff_) and Orbach prefactor (τ_0_^–1^) for **3-Dy·C**_**6**_**H**_**6**_ and **4-Dy** are very similar and reflect similar central rates in the Orbach region. Central rates are noticeably faster for **4-Dy** in the intermediate and low temperature ranges and correspond to a larger Raman prefactor (*C*) and faster QTM rate (τ_QTM_^–1^). On dilution of the CIP in a diamagnetic matrix, the Orbach and Raman rates for the major (≥90%) component are unchanged, but the QTM rate decreases to become similar to **3-Dy·C**_**6**_**H**_**6**_. For all compounds, the relaxation time is equal to 100 s in the Raman regime, leading to a higher 100 s blocking temperature for **3-Dy·C**_**6**_**H**_**6**_ (*T*_100_ = 28 K) than **4-Dy** and **5%Dy@4-Y** (*T*_100_ = 15 K). Slower relaxation in the QTM region for **3-Dy·C**_**6**_**H**_**6**_ is consistent with the smaller zero-field QTM step observed in the low temperature hysteresis loops. Distributions in relaxation rates are uniformly broader for **3-Dy·C**_**6**_**H**_**6,**_ and at most temperatures, the one ESD range encompasses the **4-Dy** one ESD range. All distributions become broader upon lowering temperature.

**Table 2:** Selected SMM Parameters for **3-Dy·C**_**6**_**H**_**6**_, **4-Dy,** and **5%Dy@4-Y** with Estimated Standard Deviations

	**3-Dy·C**_**6**_**H**_**6**_	**4-Dy**	**5%Dy@4-Y**
*U*_eff_/cm^–^^1^	1221(25)	1265(15)	1209(11)
*A* (τ_0_ = 10^*A*^/s)	–11.0(2)	–11.4(1)	–11.16(8)
*R* (*C* = 10^*R*^/s^–^^1^ K^–^^*n*^)	–8.3(5)	–6.4(3)	–5.3(1)
*n*	4.3(3)	3.4(2)	2.67(6)
*Q* (τ_QTM_ = 10^*Q*^/s)	2.54(7)	2.13(7)	2.54(4)
*U*_eff_ (calc.)/cm^–^^1^	1289	1254	
*T*_H_/K	52	36	28
*T*_100_/K	28	15	15

Relaxation data for **4-Dy** in benzene and “[{Dy(Cp^ttt^)(Cp*)}{Al[OC(CF_3_)_3_]_4_}]” had multiple components present (Figures S158 and S159), consistent with the samples being biphasic and/or amorphous. The ac relaxation rates for the major component (85%) of **4-Dy** in benzene and “[{Dy(Cp^ttt^)(Cp*)}{Al[OC(CF_3_)_3_]_4_}]” agree with those for **3-Dy·C**_**6**_**H**_**6**_ and **4-Dy** (Figures S161 and S162). The sample of **4-Dy** in benzene has broad rate distributions in the Raman region, which split into two even broader components in the QTM region; the rates encompass those for **3-Dy·C**_**6**_**H**_**6**_ and **4-Dy** (Figure S158). Amorphous “[{Dy(Cp^ttt^)(Cp*)}{Al[OC(CF_3_)_3_]_4_}]” has two components in the Raman and QTM region; the faster-relaxing component accounts for 70% of the magnetization at low temperature and has almost identical relaxation rates and distributions to **4-Dy** (Figure S163). The slower-relaxing component has rates that are very similar to those observed for **3-Dy·C**_**6**_**H**_**6**_ when both rates are measured using dc decays (Figure S164d).

### Ab Initio Calculations

The electronic structures of **3-Dy·C**_**6**_**H**_**6**_ and **4-Dy** were investigated by CASSCF-SO calculations using OpenMolcas^71^ and the atomic coordinates from SCXRD data sets (see Supporting Information for details). In both complexes, the ground state principal magnetic axis (*g*_*z*_) traverses the two Cp^R^, and the axiality of each ground doublet is confirmed by both their Ising-like *g* values (*g*_*x*_ ∼ *g*_*y*_ ∼ 0, *g*_*z*_ = 19.9) and dominant *m*_*J*_ = ± 15/2 character **3-Dy·C**_**6**_**H**_**6**_ (99.6%) and **4-Dy** (99.4%). The remaining doublets are similarly pure (>98%) for the first five excited states for **3-Dy·C**_**6**_**H**_**6**_ and the first four for **4-Dy**; upon the loss of purity, the remaining states of **4-Dy** are mixed to a greater degree than those of **3-Dy·C**_**6**_**H**_**6**_. The first excited states (*m*_*J*_ = ± 13/2) lie 449 cm^–1^ (**3-Dy·C**_**6**_**H**_**6**_) and 435 cm^–1^ (**4-Dy**) above the ground states. Calculated *U*_eff_ values are determined by the energy of the lowest excited state, for which the *g*_*z*_ vector deviates significantly (>10°) from that of the ground state; this occurs at the sixth excited state, predicted at 1289 cm^–1^ for **3-Dy·C**_**6**_**H**_**6**_ and 1254 cm^–1^ for **4-Dy**. The calculated values of *U*_eff_ lie within 3σ of the experimental values and are close to each other, consistent with experimental observations. We note that χ*T* values measured for both **3-Dy·C**_**6**_**H**_**6**_ and **4-Dy** are lower than the CASSCF-SO-predicted values across all temperatures (Figures S73 and S74), but they are similar to each other and the amorphous product. As we were unable to obtain a geometry-optimized structure of the Y(III) analogue of the CIP that was reasonably similar to the XRD structure, this precluded a fully ab initio study of the effects of coordination of the WCA on spin-phonon coupling and Orbach magnetic relaxation pathways at present. Solid-state spin-phonon coupling calculations are out of reach for these low-symmetry and large molecules at this time.

## Discussion

The isolation of both SIP and CIP polymorphs of the {Dy(Cp^ttt^)(Cp*)}^+^ fragment with the WCA [Al{OC(CF_3_)_3_}_4_]^−^ has substantiated the computational prediction made by AtomAccess calculations that this cation spans a crossover point for equatorial coordination. The mean Dy···Cp^R^_centroid_ distances of the SIP **3-Dy·C**_**6**_**H**_**6**_ (2.305(3) Å) and CIP **4-Dy** (2.308(5) Å) are comparable to the literature complexes [Dy(C_5_^i^Pr_4_H)_2_][B(C_6_F_5_)_4_] (2.29(1) Å),^17^ [Dy(Cp^ttt^)_2_][B(C_6_F_5_)_4_] (2.316(3) Å),^14^ and [Dy(C_5_^i^Pr_5_)(Cp*)][B(C_6_F_5_)_4_] (2.290(2) Å), showing that the limit of this design criterion has been reached and is not improved by reducing Cp^R^ steric bulk.^18^ The Cp^R^_centroid_···Dy···Cp^R^_centroid_ angles of **3-Dy·C**_**6**_**H**_**6**_ (149.15(9)°) and **4-Dy** (148.26(2)°) are more acute than [Dy(Cp^ttt^)_2_][B(C_6_F_5_)_4_] (152.56(7)°)^14^ and [Dy(C_5_^i^Pr_5_)(Cp*)][B(C_6_F_5_)_4_] (162.507(1)°)^18^ and are more similar to the corresponding angle in [Dy(C_5_^i^Pr_4_H)_2_][B(C_6_F_5_)_4_] (147.2(8)°).^17^ The acute angle makes the Dy center less axial and more susceptible to interactions with equatorial donor solvents and WCAs. To the best of our knowledge, the CIP complexes **4-Ln** exhibit the first structurally authenticated examples of κ^1^-F-binding of the [Al{OC(CF_3_)_3_}_4_]^−^ anion to any f-block metal ion.^72−79^ We attribute the polymorphism-induced structural changes of **3-Dy·C**_**6**_**H**_**6**_ and **4-Dy** to differences in crystal packing forces,^80^ which should be energetically similar to the weak coordination of one of the F atoms of a CF_3_ group in [Al{OC(CF_3_)_3_}_4_]^−^ to the {Dy(Cp^ttt^)(Cp*)}^+^ moiety.

The magnetic properties of **3-Dy·C**_**6**_**H**_**6**_ and **4-Dy** are similar to other dysprosocenium SMMs in the literature.^14−18^ The SIP shows superior SMM behavior to the CIP as evidenced by history-dependent effects being observed up to higher temperatures, with significant differences in both *T*_irrev_ values (40 vs 28 K) and *T*_H_ (52 vs 36 K); the significant differences in dynamic magnetic properties between these samples are in accord with the results from PXRD, which show high phase purities. The maximum hysteresis temperature of [Dy(C_5_^i^Pr_4_H)_2_][B(C_6_F_5_)_4_] (*T*_H_ = 32 K) is similar to the CIP, while that of [Dy(Cp^ttt^)_2_][B(C_6_F_5_)_4_] (*T*_H_ = 60 K)^14^ is slightly higher than the SIP, and [Dy(C_5_^i^Pr_5_)(Cp*)][B(C_6_F_5_)_4_] is higher still (*T*_H_ = 80 K).^18^ Weak coordination of [Al{OC(CF_3_)_3_}_4_]^−^ has a minor effect on the static crystal field potential, as evidenced by ab initio calculations and nearly superimposable relaxation data for the SIP and CIP in all environments (pure, doped, amorphous and in benzene) in the Orbach-dominated temperature region (Figures 5, S160–S162). While the experimental *U*_eff_ values (CIP: 1265 ± 15 cm^–1^ vs SIP: 1221 ± 25 cm^–1^) are not statistically equivalent, they are similar and are within error of the corresponding value for [Dy(Cp^ttt^)_2_][B(C_6_F_5_)_4_]^14^ (1237 ± 28 cm^–1^);^68^ the CIP is also within error of [Dy(C_5_^i^Pr_4_H)_2_][B(C_6_F_5_)_4_]^17^ (1286 ± 14 cm^–1^),^68^ and both the CIP and SIP are lower than that of the best-performing dysprosocenium SMM to date, [Dy(C_5_^i^Pr_5_)(Cp*)][B(C_6_F_5_)_4_]^18^ (1550 ± 7 cm^–1^).^68^ While the relaxation rates in the Orbach region, which are controlled by high-energy intramolecular vibrations, are almost identical for the SIP and CIP, this is not true in the Raman region, which is dictated by low-energy molecular rotations and intermolecular vibrations (pseudoacoustic phonons).^81^ As we observe that the components of the magnetic relaxation rates in “[{Dy(Cp^ttt^)(Cp*)}{Al[OC(CF_3_)_3_]_4_}]” are similar to the rates for **3-Dy·C**_**6**_**H**_**6**_ and **4-Dy**, we posit that the coordination of the WCA increases the impact of the pseudoacoustic phonons on the [Dy(Cp^ttt^)(Cp*)]^+^ cation for the CIP relative to the SIP,^82^ and that this is not impacted by crystallinity. Attempted solution measurements were inconclusive and are broadly consistent with the presence of SIP and CIP, with very broad distributions in geometries.

Conformational analysis and AtomAccess calculations reveal that the [Dy(Cp^ttt^)(Cp*)]^+^ cation is flexible and can accommodate a broad range of Dy environments, consistent with the large variance of relaxation rates in the magnetic data for SIP **3-Dy·C**_**6**_**H**_**6**_ and CIP **4-Dy** in benzene. The coordination of [Al{OC(CF_3_)_3_}_4_]^−^ in **4-Dy** reduces the overall size of the atomic displacement parameters in the SCXRD structure relative to those in **3-Dy·C**_**6**_**H**_**6**_ and appears to pin both the cation and anion in place, reducing both the flexibility of the [Dy(Cp^ttt^)(Cp*)]^+^ cation and the distributions of relaxation rates. The ESDs of all relaxation rates increase upon entering the QTM region, and the QTM rates vary significantly among the five [Dy(Cp^ttt^)(Cp*)]^+^ samples, indicating that the QTM process is the most sensitive to the distribution of Dy environments. Upon doping in a diamagnetic host, the QTM rates of the CIP decrease, in accord with dipolar fields being critical for enabling QTM. The faster QTM rate for **4-Dy** than **3-Dy·C**_**6**_**H**_**6**_ is likely a combination of the coordination of the WCA resulting in minor changes to the ground doublet and increased dipolar interactions from the shorter Dy···Dy nearest neighbor distances (Table 1). The QTM tunneling times for **3-Dy·C**_**6**_**H**_**6**_ (347 s) and **4-Dy** (135 s) are close to that of [Dy(C_5_^i^Pr_4_H)_2_][B(C_6_F_5_)_4_]^17^ (*e*^⟨lnτ⟩^ = 363 s at 2 K),^69^ which has a similarly acute Cp^R^_centroid_···Dy···Cp^R^_centroid_ angle. Although the coordination of the WCA in the CIP promotes faster magnetic relaxation, it is noteworthy that the differences between the SMM behavior of the SIP and CIP in this work are relatively small compared to the more detrimental equatorial interactions seen for {Dy(Cp^R^)_2_}^+^ fragments previously described in the literature.^14−21,28−34^

## Conclusions

We have shown that as the [Dy(Cp^ttt^)(Cp*)]^+^ cation is of intermediate steric bulk, it can form both separated ion-pair and contact ion-pair polymorphs with a weakly coordinating perfluoroalkoxyaluminate anion in the solid state; the contact ion-pair polymorph provides an unprecedented binding mode of this weakly coordinating anion to a rare earth metal ion. The separated ion-pair polymorph exhibits magnetic behavior that is in accord with previously reported isolated dysprosocenium SMMs, while the weak equatorial interaction in the contact ion-pair is shown to diminish magnetic properties; the combination of relatively small Cp^R^ rings leads to larger Cp^R^···Dy···Cp^R^ angles but does not provide shorter Dy···Cp^R^ distances, so these complexes have lower axiality than the best-performing dysprosocenium SMMs.^14−18^ Surprisingly, the Orbach relaxation rate does not appear to be significantly affected as the axiality of the crystal field is not substantially reduced in the contact ion-pair polymorph. However, anion coordination enhances the impact of localized pseudoacoustic phonons, resulting in accelerated Raman relaxation.

The observation of both separated ion-pair and contact ion-pair forms of [Dy(Cp^ttt^)(Cp*)]^+^ has confirmed the prediction made by AtomAccess that this cation is at the borderline of forming interactions with additional ligands. These results test the limitations of the AtomAccess code, which uses a simple line of sight solid angle approach and treats potential ligands as hard spheres with estimated bond lengths. These approximations and the strategy employed in our screening of target molecules do not consider specific interactions and the energy differences of binding compared to crystal packing forces and molecular deformations. However, we anticipate that the rapid screening provided by AtomAccess can be used in combination with more expensive calculations once synthetic targets have been refined; these can then be used to determine energetic penalties of molecular distortions of specific ancillary ligands when accommodating energetically favorable additional ligand binding at a metal site.

While the smaller [Dy(Cp*)_2_]^+^ cation is anticipated to be a high performance SMM,^26,29^ AtomAccess has confirmed that its coordination site is significantly larger than [Dy(Cp^ttt^)(Cp*)]^+^. The isolation of [Dy(Cp*)_2_]^+^ will therefore be challenging and may require an alternative synthetic approach, such as using a more weakly coordinating anion than [Al{OC(CF_3_)_3_}_4_]^−^.^55^ Cooperative effects from increasing the charges on aromatic rings and therefore decreasing Dy···ring_centroid_ distances appears to be a more attractive approach to increase the axiality of Dy SMMs, as has recently been shown for Dy borolide systems.^23,24^ This work has demonstrated that AtomAccess is both a powerful analytical and predictive tool, as it accurately projects the size and topology of potential atomic binding sites; we therefore envisage that this program can be applied in the future to a wealth of challenges in exploratory synthetic chemistry.

## Supporting Information Available

Additional experimental details, materials, methods, and data (PDF)

## Notes

Additional research data supporting this publication are available from Figshare at https://figshare.com/articles/dataset/AtomAccess_A_predictive_tool_for_molecular_design_and_its_application_to_the_targeted_synthesis_of_dysprosium_single-molecule_magnets/22147196.
