# Supplementary material for: AtomAccess: A Predictive Tool for Molecular Design and Its Application to the Targeted Synthesis of Dysprosium Single-Molecule Magnets
Source: J Am Chem Soc. 2023 Oct 5;145(41):22814–25. doi: 10.1021/jacs.3c08841 (PMC10591469; doi:10.1021/jacs.3c08841)
Supplement: Supplementary file 1 — ja3c08841_si_001.pdf [file ja3c08841_si_001.pdf]

*Supplementary Information for:*

# ***AtomAccess*: A predictive tool for molecular design and its application to the targeted synthesis of dysprosium single-molecule magnets**

*Gemma K. Gransbury,‡ Sophie C. Corner,‡ Jon G. C. Kragskow, Peter Evans, Hing Man Yeung, William J. A. Blackmore, George F. S. Whitehead, Iñigo J. Vitorica-Yrezabal, Meagan S. Oakley, Nicholas F. Chilton* and David P. Mills**

Department of Chemistry, The University of Manchester, Oxford Road, Manchester, M13 9PL, U.K.

**Contents**

1. *AtomAccess* Calculations ....................S3

1.1 AtomAccess Program ....................S3

1.2 X-ray Diffraction Database ....................S11

1.3 Conformational Analysis ....................S27

2. General Synthetic Methods ....................S30

3. Synthesis ....................S31

4. Infrared Spectroscopy ....................S39

5. Powder X-ray Diffraction ....................S46

6. NMR Spectroscopy ....................S52

7. Single Crystal X-ray Diffraction ....................S69

8. DFT Calculations ....................S85

9. Magnetic Measurements ....................S87

9.1 Temperature- and Field-Swept Magnetic Measurements ....................S87

9.2 Ac Magnetic Measurements ....................................................................................S100

9.3 Waveform Measurements ........................................................................................S117

9.4 Magnetization Decays...............................................................................................S144

9.5 Relaxation Profiles.....................................................................................................S171

10. CASSCF-SO Calculations ......................................................................................S194

11. References...............................................................................................................S203

## 1. ***AtomAccess* Calculations**

### *1.1 AtomAccess Program*

*Defining Rays and Spheres*

The AtomAccess program performs a ray-tracing calculation for a set of rays emanating from a single central atom of a molecule chosen by the user, finding all intersections of these rays with the other atoms of the molecule. For simplicity, *AtomAccess* first shifts the molecule such that the coordinates of the central atom define the origin of the coordinate system [0, 0, 0]. Then, a set of rays are created which emanate outwards from the origin, with directions specified by the ZCW algorithm, such that they evenly sample the surface of a sphere. All other atoms other than the central atom are then treated as hard spheres with van der Waals (vdW) radii. We disregard all atoms which have no intersection with a spherical region of radius $r_{\mathrm{max}}$ centered on the central atom. For the remaining atoms, every possible intersection point of every ray with every atom is calculated using the ray-sphere interaction method (*vide infra*).

*Ray-Sphere Interaction*

Consider the interaction of a single unit-ray $\hat{\mathbf{u}}$ which emanates from the origin O and may or may not intersect a sphere of radius $r$, with a center specified by the position vector $\vec{\mathbf{C}}$ (Figure S1). Assuming that the origin is not enclosed within this sphere, we seek to find the intersection point(s) $p_1$ and $p_2$ where the ray enters and leaves the sphere, if they exist at all.

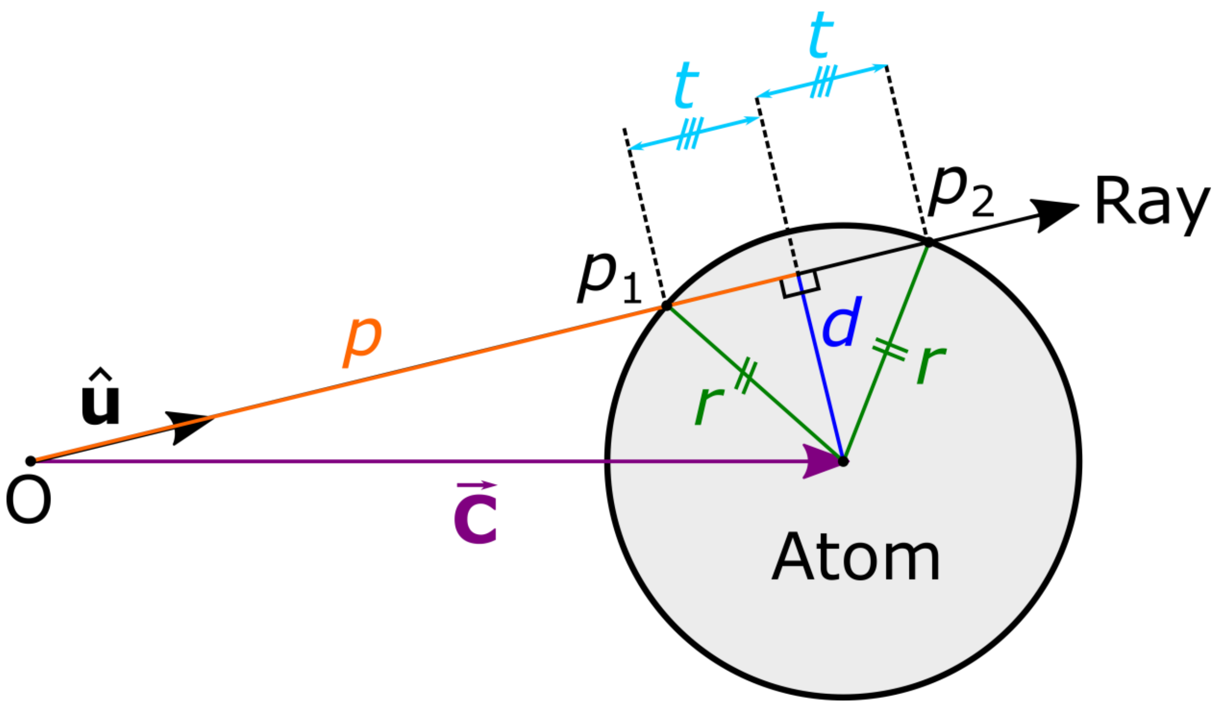


**Figure S1.** Ray tracing diagram for determining ray intersection with atoms. O: origin, $\hat{\mathbf{u}}$: ray unit vector, $\vec{\mathbf{C}}$: vector from origin to atom center, *r*: vdW radius of atom.

The dot product $p = \hat{\mathbf{u}} \cdot \vec{\mathbf{C}}$ defines the projection of $\vec{\mathbf{C}}$ onto the ray (**Error! Reference source n ot found.**). If $p$ is negative, then $\vec{\mathbf{C}}$ and $\hat{\mathbf{u}}$ are in opposite directions and we immediately conclude that there is no intersection. For rays with positive *p*, we define the quantity *d* as the shortest distance between the centre of the sphere and the ray. Using Pythagoras' Theorem $d^2 = |\vec{\mathbf{C}}|^2 - p^2$. We then have three possibilities:

1. $d^2 > r^2$: The ray does not intersect with the sphere,
2. $d^2 = r^2$: The ray intersects with the sphere once,
3. $d^2 < r^2$: The ray intersects with the sphere twice.

In the final two cases, the intersections occur at a distance $p \pm t$ along the ray (**Error! R eference source not found.**), where $t^2 = r^2 - d^2$. Then the intersection points $p_1$ and $p_2$ are defined by the vector equations:

$$p_1 = 0 + (p - t)\hat{\mathbf{u}}$$

$$p_2 = 0 + (p + t)\hat{\mathbf{u}}$$

where for a single intersection point (case 2) $t = 0$ and $p_1 = p_2$. *AtomAccess* calculates both $p_1$ and $p_2$, but only the former is retained as this intersection point is closest to the origin. We denote a ray *blocked* if it intersects with at least one atom with $p_1 < r_{\mathrm{max}}$; while all rays with no intersections are *unblocked*.

*Clustering Rays*

In order to cluster together unblocked rays into meaningful groups we use the single-linkage agglomerative clustering algorithm implemented in Scikit-Learn.[1] This successively merges individual rays to form clusters by considering the Cartesian distance between the tip of each unit ray. Rays are merged when this distance falls below a given threshold, which we define as the maximum 8th nearest neighbor distance (Figure S2), for a set of unit rays defined by the ZCW algorithm.

*AtomAccess* is usable via a command line interface, using the following command:

```
atom_access <molecule.xyz>
```

where <molecule.xyz> is the filename in .xyz format. The default settings are to calculate the accessibility of the first atom with a ZCW density of 10 (2584 rays, Table S1) and a $r_{\mathrm{max}}$ of 5 Å. In the command line the default settings are changed by adding additional arguments to the command above. To change the atom of interest:

```
--atom <integer> or -a <integer>
```

where <integer> is the index of the atom of interest (first atom listed is atom 1). To change the density of rays:

```
--density <integer> or -d <integer>
```

where <integer> is the ZCW density, ρ (Table S1). To change the radial cutoff, $r_{\mathrm{max}}$:

`--cutoff <number>` or `-c <number>`

where `<number>` is $r_{\max}$ in Å. Additional options available in the command line version include the ability to suppress printing to the terminal and suppress the output header with `-quiet` or `-q`, to save the unblocked ray python objects using pickle with `--save_rays` or `-sr`, and to plot the unblocked rays using plotly (reduced functionality compared to the web interface) with `--plot` or `-p`. Results are given as a text file (.out) with the same name as the input .xyz file, that also includes the time and date, .xyz input file name, ρ value and $r_{\max}$ value. Additional details of *AtomAccess* submodules are given in the *AtomAccess* manual.[2]

Figure S3 details the user interface on the *AtomAccess* webpage. The file can be uploaded using the "Select .xyz file" button. The default settings can be changed on the webpage by adjusting the "Centre", "Density" and "Radial Cutoff" boxes. The visualization of the molecule, atomic labels and rays can be toggled on or off. The user can adjust the style of the molecule (stick or space-fill), the ray style (spheres or cylinders) and the ray color (by cluster or single color). The left panel allows the molecule to be rotated and magnified, and the viewer control panel allows the orientation of a molecule to be replicated when returning to the website. The image can be downloaded with the "Download image" icon and the results are given under "Ray Information".

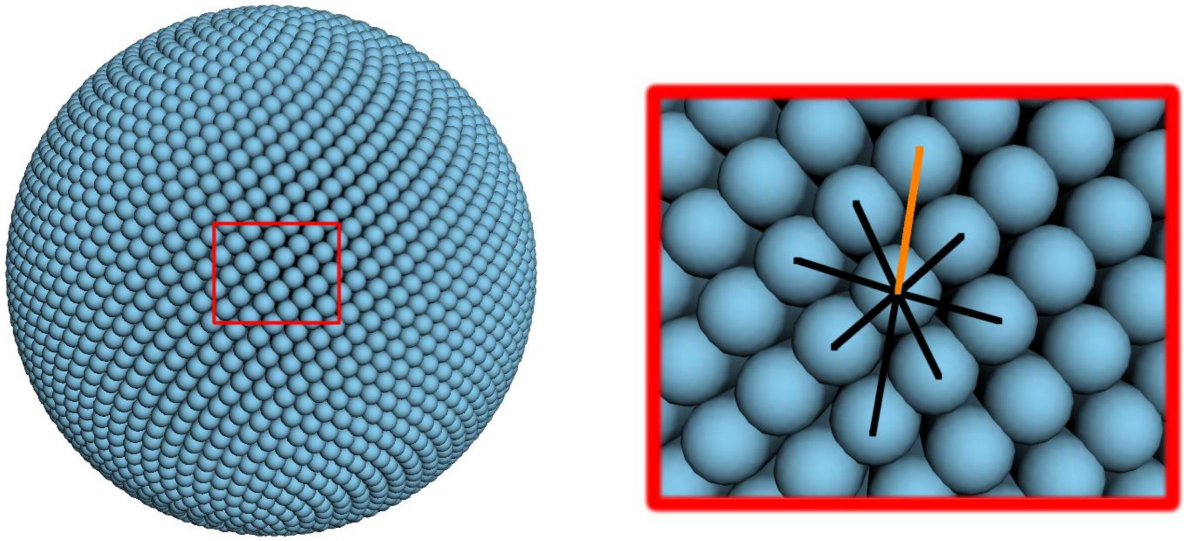

**Figure S2.** ZCW rays on a unit sphere illustrating the eighth nearest neighbor distance (orange), and distances to the seven closest neighbors (black) for a particular ray

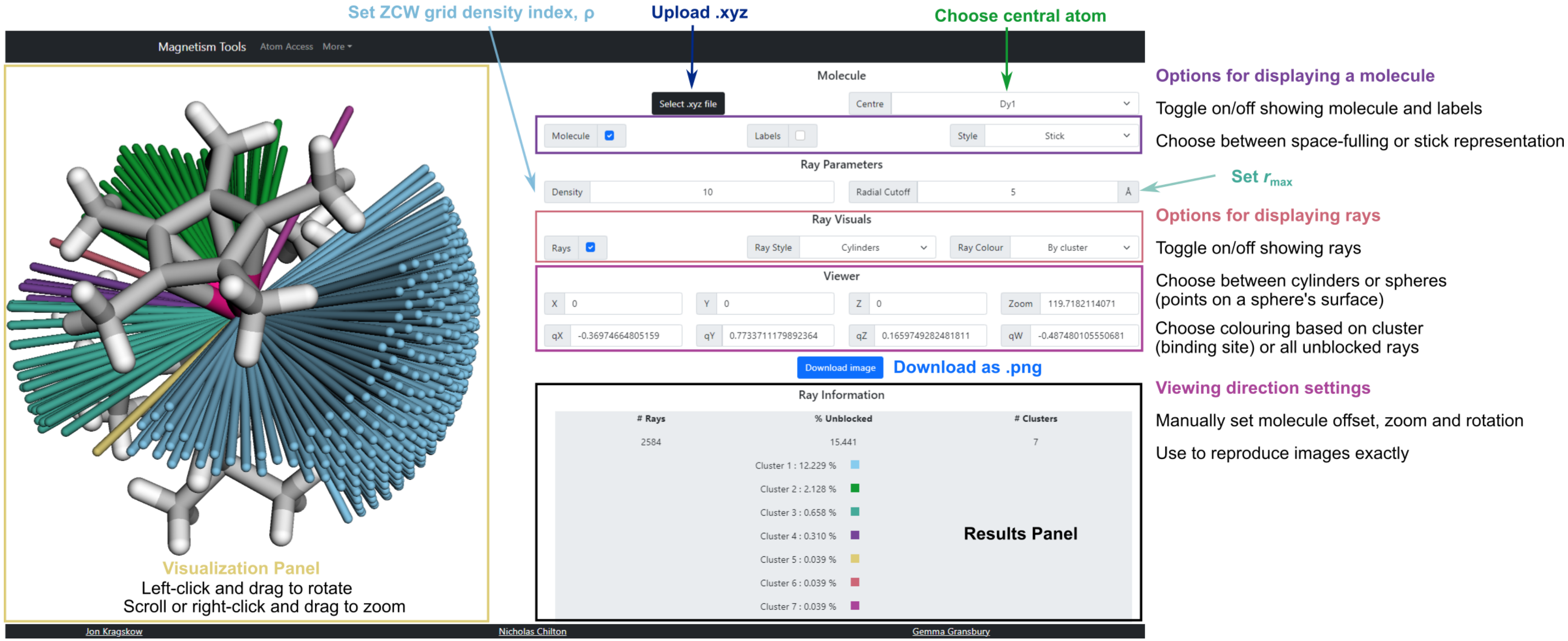


**Figure S3.** Annotated web interface for *AtomAccess*, available at https://magnetism-tools.manchester.ac.uk/apps/atom_access_app showing $\{Dy(Cp^{ttt})(Cp^{*})\}^{+}$ from **3-Dy·C6H6**

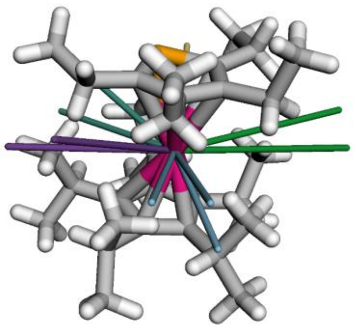

**ρ = 4**
largest cluster 2.083% SA
5 clusters

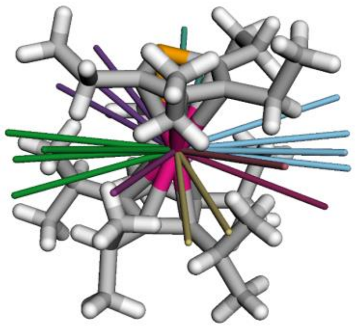

**ρ = 5**
largest cluster 1.717%
8 clusters

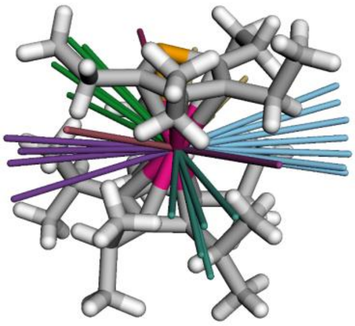

**ρ = 6**
largest cluster 1.857% SA
8 clusters

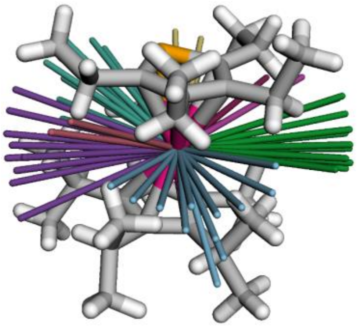

**ρ = 7**
largest cluster 1.967% SA
8 clusters

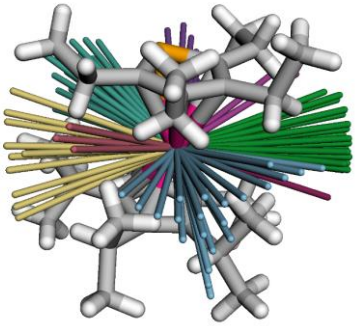

**ρ = 8**
largest cluster 2.229% SA
8 clusters

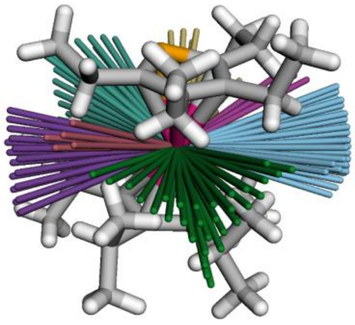

**ρ = 9**
largest cluster 2.066% SA
7 clusters

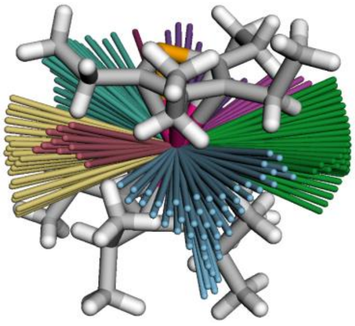

**ρ = 10**
largest cluster 2.012% SA
8 clusters

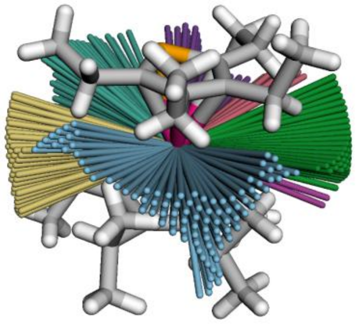

**ρ = 11**
largest cluster 2.368% SA
8 clusters

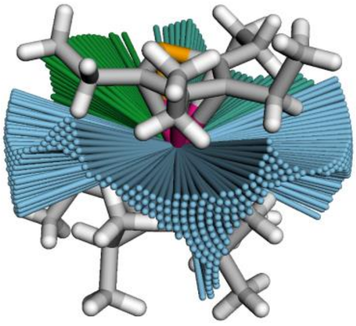

**ρ = 12**
largest cluster 5.395% SA
3 clusters

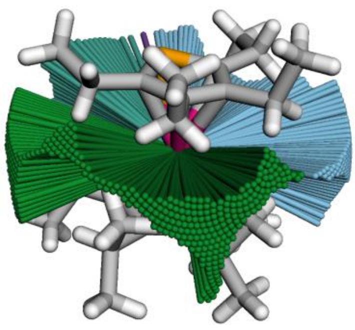

**ρ = 13**
largest cluster 3.426% SA
4 clusters

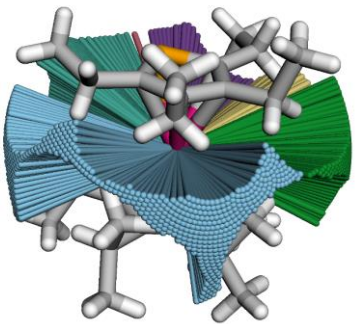

**ρ = 14**
largest cluster 3.297% SA
6 clusters

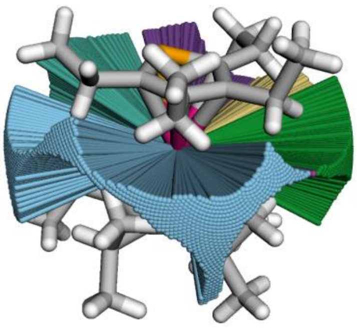

**ρ = 15**
largest cluster 3.329% SA
8 clusters

**Figure S4.** Clustering dependence on ρ for $\{Dy(C_5{}^iPr_5)(C_4PEt_4)\}^+$ (GAYPIJ_1d).[3] Clustered rays are indicated by different colors, with the largest cluster in pale blue.

**Table S1.** Errors in total % unblocked rays relative to high density ZCW grid (ρ = 15) calculated on combined crystal structure and simulated conformation databases, and single-linkage distance threshold

| ρ | Mean Unsigned Error | Maximum Unsigned Error | Number of rays | Single-linkage distance threshold |
|---|---|---|---|---|
| | (% Solid Angle) | (% Solid Angle) | | |
| 3 | 1.437 | 6.847 | 89 | 0.69352894 |
| 4 | 0.941 | 4.462 | 144 | 0.55179242 |
| 5 | 0.725 | 3.166 | 233 | 0.43319823 |
| 6 | 0.456 | 2.257 | 377 | 0.34210750 |
| 7 | 0.308 | 1.467 | 610 | 0.26988293 |
| 8 | 0.233 | 1.071 | 987 | 0.21244729 |
| 9 | 0.163 | 0.709 | 1597 | 0.16711945 |
| 10 | 0.110 | 0.466 | 2584 | 0.13131158 |
| 11 | 0.081 | 0.394 | 4181 | 0.10328090 |
| 12 | 0.058 | 0.254 | 6765 | 0.08132305 |
| 13 | 0.039 | 0.195 | 10946 | 0.06399453 |
| 14 | 0.034 | 0.206 | 17711 | 0.05033967 |
| 15 | n/a | n/a | 28657 | 0.03958930 |

*1.2 X-ray Diffraction Database*

The database of dysprosium metallocene and derivatized metallocene fragments was built from the Cambridge Structural Database (CSD), version 5.43 (September 2022).[4] Derivatized metallocenes refer to substituted rings and those including heteroatoms in the ring. Parent structures were identified by searching the CSD with Conquest 2022.2.0[5] with the following criteria:

- Contains Dy with two $\eta^5$-coordinated five-membered aromatic rings. At the time of searching this included $\{Dy(Cp^{R1})(Cp^{R2})\}^+$, $\{Dy(Cp^R)(C_4P^R)\}^+$, $\{Dy(C_4P^R)_2\}^+$ and $\{Dy(\eta^5\text{-indene})_2\}^+$
- No more than two five-membered aromatic rings per Dy i.e. no $\{Dy(Cp^R)_3\}^+$, as the $\{Dy(Cp^R)_2\}^+$ fragment is not uniquely identified
- Formal oxidation state Dy(III)
- Each $Cp^R/C_4P$ coordinates to a single Dy center
- The two $Cp^R/C_4P^R$ rings are not tethered to each other
- The $Cp^R/C_4P^R$ rings do not have substituents that equatorially coordinate to Dy e.g. cyclometallates, pendant THF, ether or thioethers. Structures with close contacts to formally neutral C atoms were not excluded on this basis.
- No structures containing Dy-hydrides
- Coordinates determined including H atoms
- If a structure is repeated in the database, both were retained only if the space group and unit cell was different. In the case of the same space group and unit cell, the structure measured at lower temperature was retained.

The parent structures were classified by *n*, the number of ancillary (non-$Cp^R/C_4P^R$) non-H atoms within 3.2 Å of Dy. This is a proxy for the number of equatorial interactions, the large value is chosen to include bonds with large atoms I, As and Sb. In the case of

$[Dy(Cp^*)_2(THF)\{Sn(Ph_3)_3\}]$ (CSD refcode TOJVIA), the Dy–Sn bond is 3.2390(7) Å and is included when counting *n* (*n* = 2). The crystal structures include cations, anions, neutral complexes, salt-bridged complexes, dimers, trimers, oligomers, and polymers.

For each parent crystal structure, the atomic coordinates of the $\{Dy(Cp^{R1})(Cp^{R2})\}^+$, $\{Dy(Cp^R)(C_4P^R)\}^+$ or $\{Dy(C_4P^R)_2\}^+$ fragment were obtained by removing ancillary ligand(s), counterions and co-crystallized solvent. A set of atomic coordinates was generated for each crystallographically unique $\{Dy(Cp^{R1})(Cp^{R2})\}^+$ unit (1, 2, 3, …) and for each disordered component (a, b, c, …), considering whether the disorder was correlated or not. The fragments were given unique labels derived the CSD refcode of the original structure and these numbers and letters e.g. the $\{Dy(Cp^*)_2\}^+$ fragment generated from $[Dy(Cp^*)_2(THF)\{Sn(Ph_3)_3\}]$ is TOJVIA_1a as there is one crystallographically unique Dy and no disorder. One exception was $[Dy(C_5{}^iPr_5)_2]^+$ (CSD refcode WIRGUC), which has ten $^i$Pr groups each disordered over two positions, with minimal correlation in disorder, and Dy disordered over four positions, corresponding to approximately $2^{12}$ = 4096 unique conformations. To reduce this to a manageable 32 conformations, only components with > 0.3 occupancy were included, while avoiding steric clashing of $^i$Pr groups.

*AtomAccess* was used to calculate the size of the largest cluster and the total % unblocked for the XRD database and the $\{Dy(Cp^{ttt})(Cp^*)\}^+$ fragments from **2-Dy**, **3-Dy·$C_6H_6$** and **4-Dy** with ρ = 3–15. The *AtomAccess* results with ρ = 10 are given in Table S2 and Table S3, respectively. All discussion of *AtomAccess* results assumes ρ = 10; the data from other densities were used to assess the error in the total % unblocked and determine the appropriate ρ value to use. The size of the largest cluster and the equatorial coordination number, *n*, were correlated with the total % unblocked, the $Cp^{R1}_{centroid}\cdots Dy\cdots Cp^{R2}_{centroid}$ angle and the mean $Dy\cdots Cp_{centroid}$ distance (Figures S5–S7). Box plots were constructed illustrating the correlation of the largest cluster size against *n* (Figure 2), the individual $\{Dy(Cp^{R1})(Cp^{R2})\}^+$ fragments (Figure S8) and

the {Dy(Cp$^{R1}$)(Cp$^{R2}$)}$^+$ fragment grouped by whether the isolated [Dy(Cp$^{R1}$)(Cp$^{R2}$)]$^+$ cation has been crystallized (Figure S9).

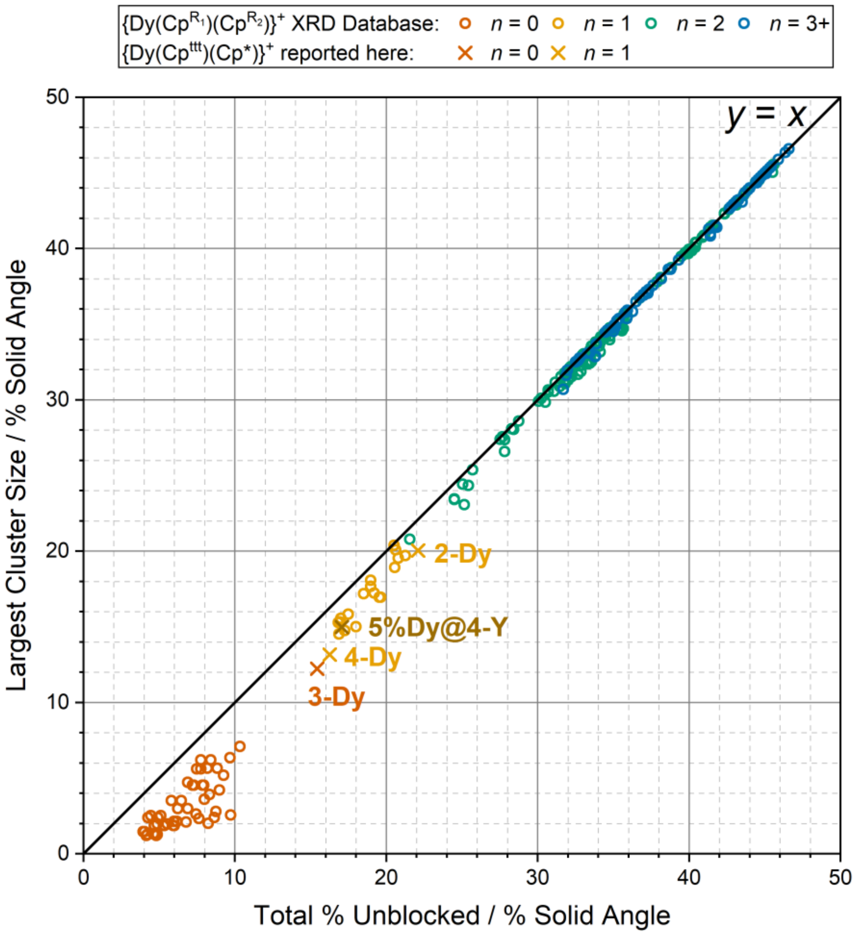


**Figure S5.** Comparison of Total % Unblocked, Largest Cluster (% SA) and equatorial coordination number ($n$) for XRD database of {Dy(Cp$^{R1}$)(Cp$^{R2}$)}$^+$ fragments, as calculated with *AtomAccess*.

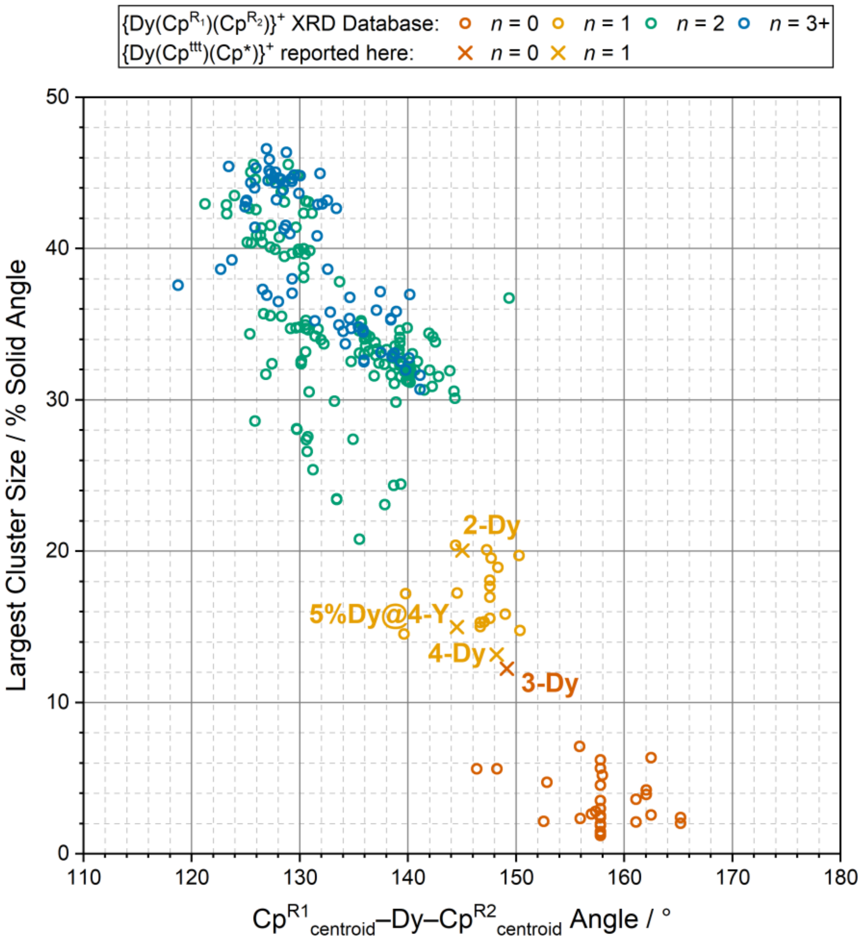


**Figure S6.** Correlation of Largest Cluster (% SA) calculated with *AtomAccess*, the $Cp^{R1}_{centroid}\cdots Dy\cdots Cp^{R2}_{centroid}$ angle and the equatorial coordination number ($n$) for XRD database of $\{Dy(Cp^{R1})(Cp^{R2})\}^+$ fragments.

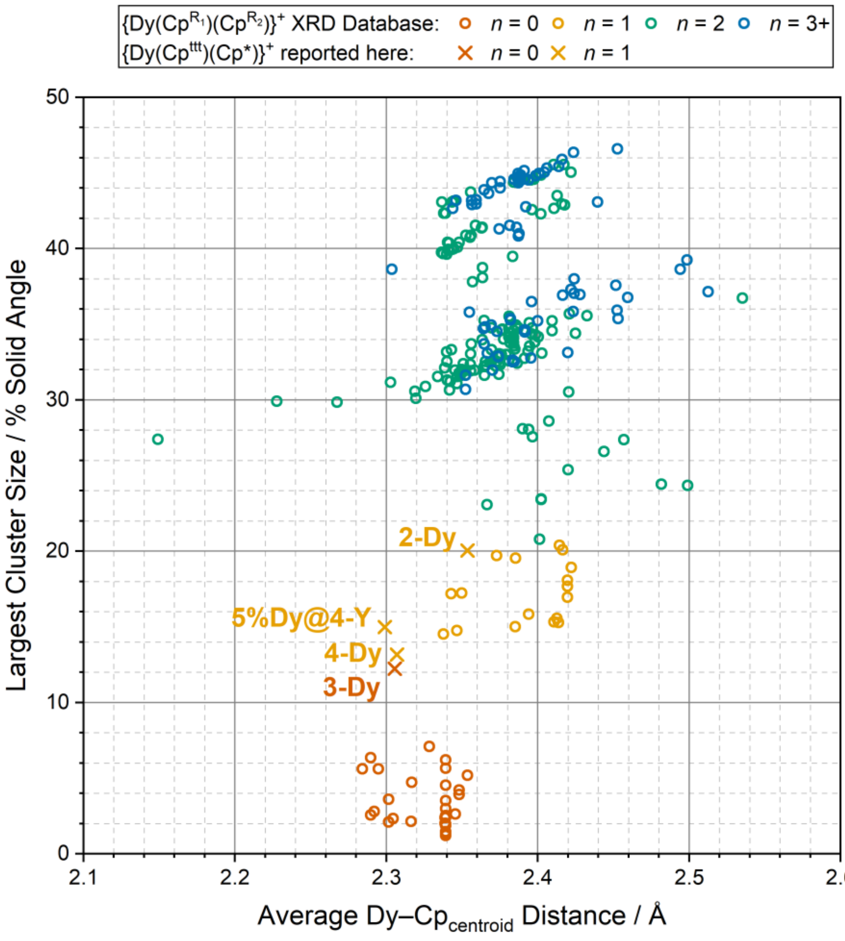


**Figure S7.** Correlation of Largest Cluster (% SA) calculated with *AtomAccess*, the mean Dy···Cp$_{centroid}$ distance and the equatorial coordination number ($n$) for XRD database of $\{Dy(Cp^{R1})(Cp^{R2})\}^+$ fragments.

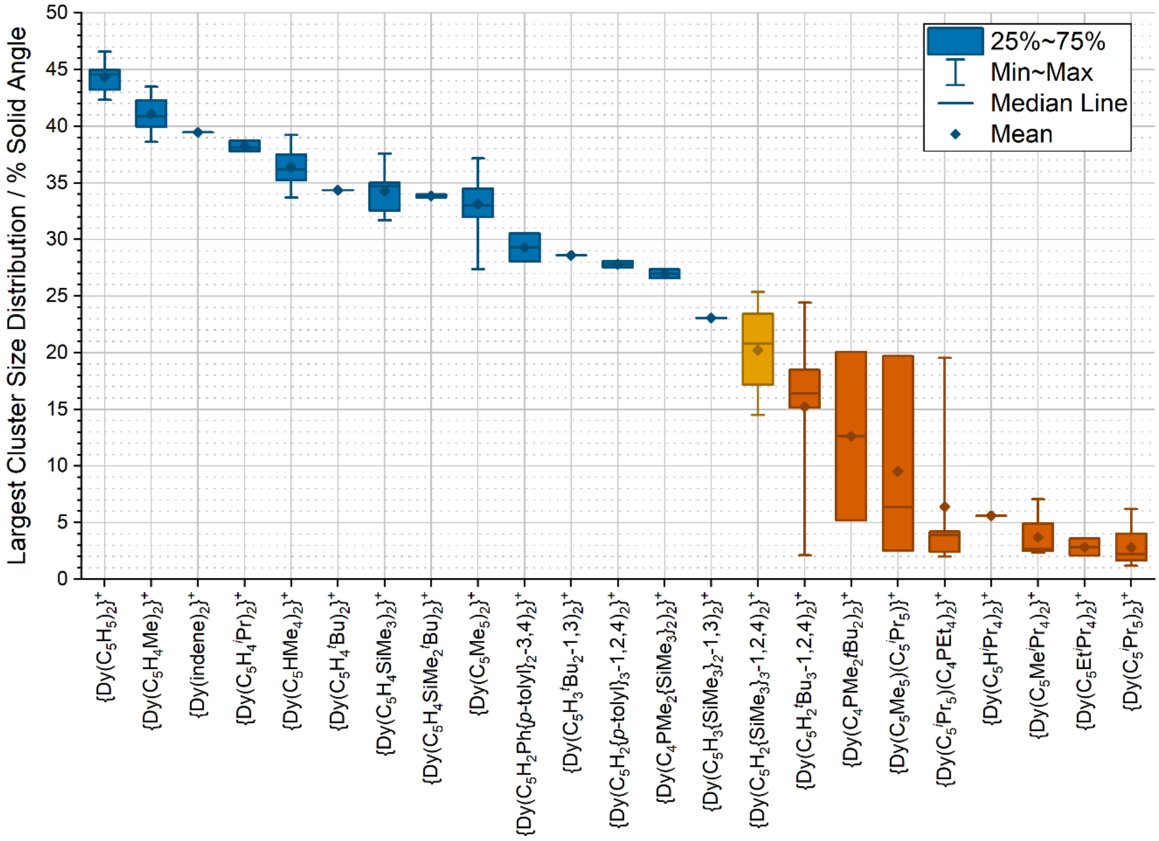


**Figure S8.** Largest cluster size distribution (% SA) of various $\{Dy(Cp^R)_2\}^+$ fragments calculated on crystal coordinates with *AtomAccess*. Data in blue are $\{Dy(Cp^R)_2\}^+$ which have only been reported with supporting equatorial interactions. Data in red are $\{Dy(Cp^R)_2\}^+$ where isolated $[Dy(Cp^R)_2]^+$ has been reported for that $Cp^R$. Data in yellow are associated with $\{Dy(C_5H_2(SiMe_3)_3\text{-}1,2,4)_2\}^+$, which is yet to be isolated as $[Dy(C_5H_2(SiMe_3)_3\text{-}1,2,4)_2]^+$ due to side reactions between abstraction agents and $\{C_5H_2(SiMe_3)_3\text{-}1,2,4\}^-$ or facile decomposition of $[Dy(C_5H_2(SiMe_3)_3\text{-}1,2,4)_2]^+$.[6]

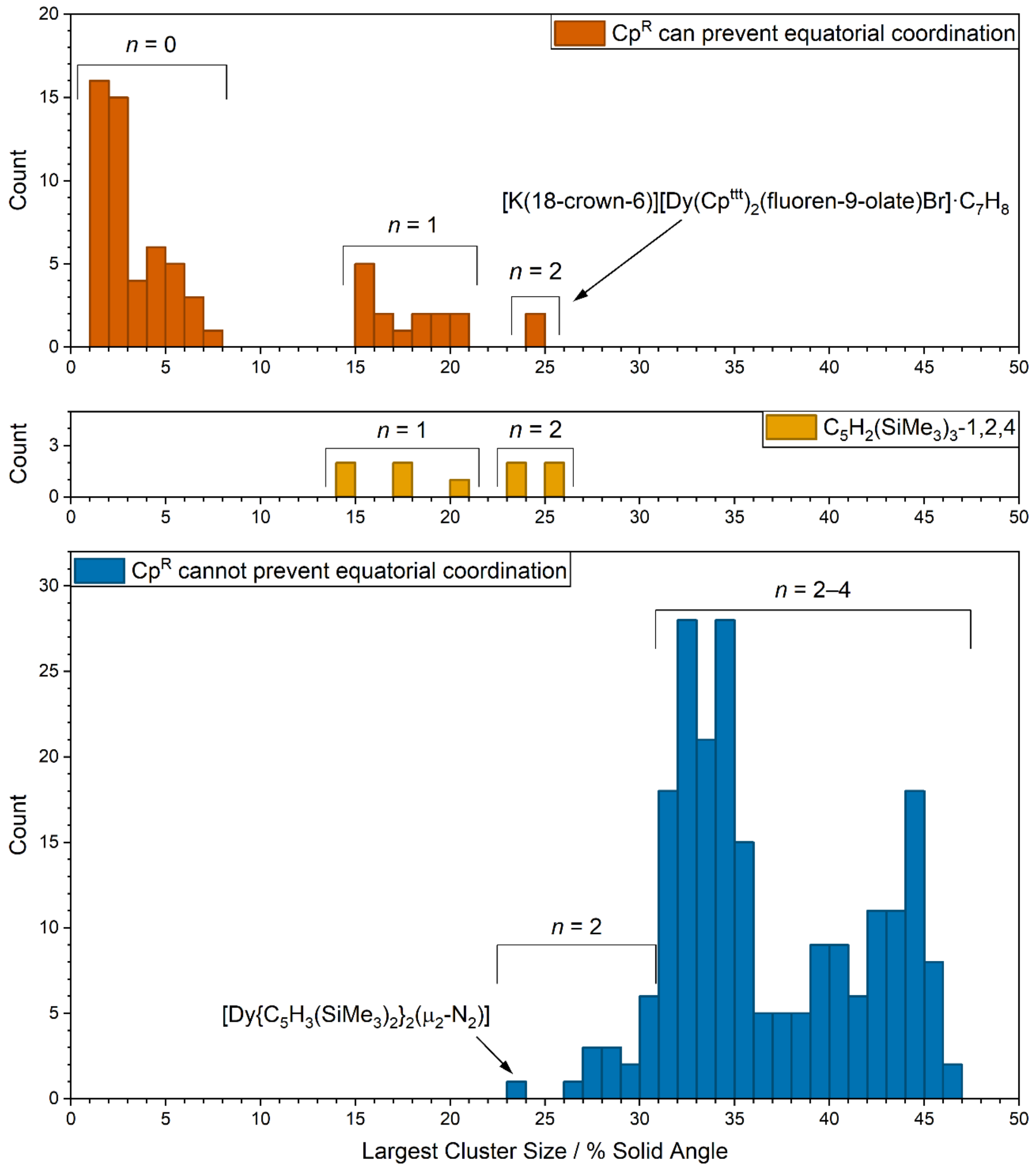


**Figure S9.** Histogram of the largest cluster size distribution (% SA) of $\{Dy(Cp^R)_2\}^+$ fragments calculated on crystal coordinates with *AtomAccess*. Data in blue are $\{Dy(Cp^R)_2\}^+$ always reported with supporting equatorial interactions ($n$ = 2–4). Data in red are associated with $Cp^R$ where $\{Dy(Cp^R)_2\}^+$ have been reported without equatorial interactions ($n$ = 0–2). Data in yellow are associated with $\{Dy(C_5H_2\{SiMe_3\}_3\text{-}1,2,4)_2\}^+$ ($n$ = 1–2), which is yet to be isolated as $[Dy\{C_5H_2(SiMe_3)_3\text{-}1,2,4\}_2]^+$ due to side reactions between abstraction agents and $\{C_5H_2(SiMe_3)_3\text{-}1,2,4\}^-$ or facile decomposition of $[Dy\{C_5H_2(SiMe_3)_3\text{-}1,2,4\}_2]^+$.[6]

**Table S2.** *AtomAccess* calculations on database of $\{Dy(Cp^R)_2\}^+$, $\{Dy(Cp^R)(C_4P^R)\}^+$ and $\{Dy(C_4P^R)_2\}^+$ fragments with coordinates taken from the Cambridge Structural Database version 5.43 (including updates until September 2022). *n* = number of non-H ancillary ligand atoms within 3.2 Å of Dy in the original structure, Unique Code is CSD Refcode followed by a number identifying the crystallographically unique fragment in the unit cell, followed by a letter for different disordered components.

| *n* | Unique Code | $Cp^{R1}$ | $Cp^{R2}$ | Total % Unblocked | Largest Cluster |
|---|---|---|---|---|---|
| | | | | (% SA) | (% SA) |
| 0 | DUBKET_1a | $C_4PMe_2{}^tBu_2$ | $C_4PMe_2{}^tBu_2$ | 9.249 | 5.186 |
| 0 | BAWLOD_1a | $C_5H_2{}^tBu_3$-1,2,4 | $C_5H_2{}^tBu_3$-1,2,4 | 6.889 | 4.721 |
| 0 | MEKDOY_1a | $C_5H_2{}^tBu_3$-1,2,4 | $C_5H_2{}^tBu_3$-1,2,4 | 5.960 | 2.128 |
| 0 | MEKDOY_1b | $C_5H_2{}^tBu_3$-1,2,4 | $C_5H_2{}^tBu_3$-1,2,4 | 6.153 | 2.128 |
| 0 | WIRHEN_1a | $C_5Et{}^iPr_4$ | $C_5Et{}^iPr_4$ | 6.772 | 2.090 |
| 0 | WIRHEN_1b | $C_5Et{}^iPr_4$ | $C_5Et{}^iPr_4$ | 7.972 | 3.599 |
| 0 | WIRHAJ_1a | $C_5H{}^iPr_4$ | $C_5H{}^iPr_4$ | 7.740 | 5.611 |
| 0 | WIRHAJ_2a | $C_5H{}^iPr_4$ | $C_5H{}^iPr_4$ | 7.469 | 5.611 |
| 0 | WIRHIR_1a | $C_5Me{}^iPr_4$ | $C_5Me{}^iPr_4$ | 8.746 | 2.786 |
| 0 | WIRHIR_1b | $C_5Me{}^iPr_4$ | $C_5Me{}^iPr_4$ | 7.430 | 2.632 |
| 0 | WIRHIR_2a | $C_5Me{}^iPr_4$ | $C_5Me{}^iPr_4$ | 7.624 | 2.322 |
| 0 | WIRHIR_2b | $C_5Me{}^iPr_4$ | $C_5Me{}^iPr_4$ | 10.333 | 7.082 |
| 0 | GAYPIJ_1a | $C_5{}^iPr_5$ | $C_4PEt_4$ | 8.630 | 2.399 |
| 0 | GAYPIJ_1b | $C_5{}^iPr_5$ | $C_4PEt_4$ | 8.320 | 3.909 |
| 0 | GAYPIJ_1c | $C_5{}^iPr_5$ | $C_4PEt_4$ | 8.978 | 4.218 |
| 0 | GAYPIJ_1d | $C_5{}^iPr_5$ | $C_4PEt_4$ | 8.243 | 2.012 |
| 0 | WIRGUC_1a | $C_5{}^iPr_5$ | $C_5{}^iPr_5$ | 5.998 | 1.858 |
| 0 | WIRGUC_1b | $C_5{}^iPr_5$ | $C_5{}^iPr_5$ | 4.450 | 2.515 |
| 0 | WIRGUC_1c | $C_5{}^iPr_5$ | $C_5{}^iPr_5$ | 7.933 | 4.528 |
| 0 | WIRGUC_1d | $C_5{}^iPr_5$ | $C_5{}^iPr_5$ | 5.921 | 1.858 |
| 0 | WIRGUC_1e | $C_5{}^iPr_5$ | $C_5{}^iPr_5$ | 7.856 | 4.528 |
| 0 | WIRGUC_1f | $C_5{}^iPr_5$ | $C_5{}^iPr_5$ | 4.876 | 1.277 |
| 0 | WIRGUC_1g | $C_5{}^iPr_5$ | $C_5{}^iPr_5$ | 4.605 | 1.316 |
| 0 | WIRGUC_1h | $C_5{}^iPr_5$ | $C_5{}^iPr_5$ | 4.799 | 1.200 |
| 0 | WIRGUC_1i | $C_5{}^iPr_5$ | $C_5{}^iPr_5$ | 6.889 | 2.980 |

| | | | | | |
|---|---|---|---|---|---|
| 0 | WIRGUC_1j | $C_5{}^iPr_5$ | $C_5{}^iPr_5$ | 8.824 | 5.650 |
| 0 | WIRGUC_1k | $C_5{}^iPr_5$ | $C_5{}^iPr_5$ | 6.463 | 3.522 |
| 0 | WIRGUC_1l | $C_5{}^iPr_5$ | $C_5{}^iPr_5$ | 8.398 | 6.192 |
| 0 | WIRGUC_1m | $C_5{}^iPr_5$ | $C_5{}^iPr_5$ | 5.341 | 1.858 |
| 0 | WIRGUC_1n | $C_5{}^iPr_5$ | $C_5{}^iPr_5$ | 5.534 | 1.974 |
| 0 | WIRGUC_1o | $C_5{}^iPr_5$ | $C_5{}^iPr_5$ | 4.915 | 2.399 |
| 0 | WIRGUC_1p | $C_5{}^iPr_5$ | $C_5{}^iPr_5$ | 5.108 | 2.515 |
| 0 | WIRGUC_1q | $C_5{}^iPr_5$ | $C_5{}^iPr_5$ | 5.341 | 1.858 |
| 0 | WIRGUC_1r | $C_5{}^iPr_5$ | $C_5{}^iPr_5$ | 7.276 | 4.528 |
| 0 | WIRGUC_1s | $C_5{}^iPr_5$ | $C_5{}^iPr_5$ | 5.263 | 1.858 |
| 0 | WIRGUC_1t | $C_5{}^iPr_5$ | $C_5{}^iPr_5$ | 7.198 | 4.528 |
| 0 | WIRGUC_1u | $C_5{}^iPr_5$ | $C_5{}^iPr_5$ | 4.025 | 1.471 |
| 0 | WIRGUC_1v | $C_5{}^iPr_5$ | $C_5{}^iPr_5$ | 4.218 | 1.277 |
| 0 | WIRGUC_1w | $C_5{}^iPr_5$ | $C_5{}^iPr_5$ | 3.947 | 1.471 |
| 0 | WIRGUC_1x | $C_5{}^iPr_5$ | $C_5{}^iPr_5$ | 4.141 | 1.200 |
| 0 | WIRGUC_1y | $C_5{}^iPr_5$ | $C_5{}^iPr_5$ | 6.231 | 2.980 |
| 0 | WIRGUC_1z | $C_5{}^iPr_5$ | $C_5{}^iPr_5$ | 8.166 | 5.650 |
| 0 | WIRGUC_1aa | $C_5{}^iPr_5$ | $C_5{}^iPr_5$ | 5.805 | 3.522 |
| 0 | WIRGUC_1ab | $C_5{}^iPr_5$ | $C_5{}^iPr_5$ | 7.740 | 6.192 |
| 0 | WIRGUC_1ac | $C_5{}^iPr_5$ | $C_5{}^iPr_5$ | 4.683 | 1.858 |
| 0 | WIRGUC_1ad | $C_5{}^iPr_5$ | $C_5{}^iPr_5$ | 4.876 | 1.974 |
| 0 | WIRGUC_1ae | $C_5{}^iPr_5$ | $C_5{}^iPr_5$ | 4.257 | 2.399 |
| 0 | WIRGUC_1af | $C_5{}^iPr_5$ | $C_5{}^iPr_5$ | 4.683 | 1.316 |
| 0 | LIRQUB_1a | $C_5{}^iPr_5$ | $C_5Me_5$ | 9.675 | 6.347 |
| 0 | LIRQUB_1b | $C_5{}^iPr_5$ | $C_5Me_5$ | 9.714 | 2.554 |
| 1 | UGUCIJ_1a | $C_4PMe_2{}^tBu_2$ | $C_4PMe_2{}^tBu_2$ | 20.627 | 20.085 |
| 1 | FAYYEN_1a | $C_5H_2(SiMe_3)_3$-1,2,4 | $C_5H_2(SiMe_3)_3$-1,2,4 | 17.260 | 14.745 |
| 1 | UGUCEF_1a | $C_5H_2(SiMe_3)_3$-1,2,4 | $C_5H_2(SiMe_3)_3$-1,2,4 | 19.195 | 17.221 |
| 1 | UGUCEF_2a | $C_5H_2(SiMe_3)_3$-1,2,4 | $C_5H_2(SiMe_3)_3$-1,2,4 | 18.498 | 17.183 |
| 1 | UGUCEF_3a | $C_5H_2(SiMe_3)_3$-1,2,4 | $C_5H_2(SiMe_3)_3$-1,2,4 | 16.873 | 14.512 |
| 1 | BAWLIX_1a | $C_5H_2{}^tBu_3$-1,2,4 | $C_5H_2{}^tBu_3$-1,2,4 | 16.989 | 15.557 |
| 1 | BAWLIX01_1a | $C_5H_2{}^tBu_3$-1,2,4 | $C_5H_2{}^tBu_3$-1,2,4 | 16.834 | 15.286 |
| 1 | BAWLIX01_1b | $C_5H_2{}^tBu_3$-1,2,4 | $C_5H_2{}^tBu_3$-1,2,4 | 20.511 | 20.395 |
| 1 | LEZZIA_1a | $C_5H_2{}^tBu_3$-1,2,4 | $C_5H_2{}^tBu_3$-1,2,4 | 17.492 | 15.828 |
| 1 | LEZZOG_1a | $C_5H_2{}^tBu_3$-1,2,4 | $C_5H_2{}^tBu_3$-1,2,4 | 16.912 | 15.325 |
| 1 | LEZZUM_1a | $C_5H_2{}^tBu_3$-1,2,4 | $C_5H_2{}^tBu_3$-1,2,4 | 17.995 | 15.015 |

| | | | | | |
|---|---|---|---|---|---|
| 1 | LIBBAA_1a | $C_5H_2{}^tBu_3$-1,2,4 | $C_5H_2{}^tBu_3$-1,2,4 | 20.550 | 18.924 |
| 1 | LIBBEE_1a | $C_5H_2{}^tBu_3$-1,2,4 | $C_5H_2{}^tBu_3$-1,2,4 | 18.963 | 18.073 |
| 1 | LIBBEE_1b | $C_5H_2{}^tBu_3$-1,2,4 | $C_5H_2{}^tBu_3$-1,2,4 | 19.543 | 16.950 |
| 1 | LIBBEE_1c | $C_5H_2{}^tBu_3$-1,2,4 | $C_5H_2{}^tBu_3$-1,2,4 | 19.621 | 16.950 |
| 1 | LIBBEE_1d | $C_5H_2{}^tBu_3$-1,2,4 | $C_5H_2{}^tBu_3$-1,2,4 | 18.963 | 17.686 |
| 1 | GAYQEG_1a | $C_5{}^iPr_5$ | $C_4PEt_4$ | 20.782 | 19.543 |
| 1 | LIRWUH_1a | $C_5{}^iPr_5$ | $C_5Me_5$ | 21.246 | 19.698 |
| 2 | UGUCOP_1a | $C_4PMe_2(SiMe_3)_2$ | $C_4PMe_2(SiMe_3)_2$ | 27.825 | 26.587 |
| 2 | UGUCOP_2a | $C_4PMe_2(SiMe_3)_2$ | $C_4PMe_2(SiMe_3)_2$ | 27.825 | 27.361 |
| 2 | XAHGAS_1a | $C_5H_2$(*p*-tolyl)$_3$-1,2,4 | $C_5H_2$(*p*-tolyl)$_3$-1,2,4 | 28.289 | 28.096 |
| 2 | XAHGAS_1b | $C_5H_2$(*p*-tolyl)$_3$-1,2,4 | $C_5H_2$(*p*-tolyl)$_3$-1,2,4 | 27.670 | 27.554 |
| 2 | FAYREG_1a | $C_5H_2(SiMe_3)_3$-1,2,4 | $C_5H_2(SiMe_3)_3$-1,2,4 | 21.556 | 20.782 |
| 2 | FAYROQ_1a | $C_5H_2(SiMe_3)_3$-1,2,4 | $C_5H_2(SiMe_3)_3$-1,2,4 | 24.497 | 23.413 |
| 2 | FAYROQ_1b | $C_5H_2(SiMe_3)_3$-1,2,4 | $C_5H_2(SiMe_3)_3$-1,2,4 | 24.497 | 23.452 |
| 2 | FAYSIL_1a | $C_5H_2(SiMe_3)_3$-1,2,4 | $C_5H_2(SiMe_3)_3$-1,2,4 | 25.697 | 25.387 |
| 2 | FAYSIL_1b | $C_5H_2(SiMe_3)_3$-1,2,4 | $C_5H_2(SiMe_3)_3$-1,2,4 | 25.697 | 25.387 |
| 2 | XAHGEW_1a | $C_5H_2$Ph(*p*-tolyl)$_2$-3,4 | $C_5H_2$Ph(*p*-tolyl)$_2$-3,4 | 30.689 | 30.534 |
| 2 | XAHGEW_1b | $C_5H_2$Ph(*p*-tolyl)$_2$-3,4 | $C_5H_2$Ph(*p*-tolyl)$_2$-3,4 | 28.406 | 28.057 |
| 2 | LIBBII_1a | $C_5H_2{}^tBu_3$-1,2,4 | $C_5H_2{}^tBu_3$-1,2,4 | 25.039 | 24.420 |
| 2 | UGUCUV_1a | $C_5H_2{}^tBu_3$-1,2,4 | $C_5H_2{}^tBu_3$-1,2,4 | 25.426 | 24.342 |
| 2 | IDOBAE_1a | $C_5H_3(SiMe_3)_2$-1,3 | $C_5H_3(SiMe_3)_2$-1,3 | 25.155 | 23.065 |
| 2 | XUTMUU_1a | $C_5H_3({}^tBu)_2$-1,3 | $C_5H_3({}^tBu)_2$-1,3 | 28.754 | 28.599 |
| 2 | TOJVEW_1a | $C_5H_4{}^iPr$ | $C_5H_4{}^iPr$ | 37.887 | 37.810 |
| 2 | TOJVEW_2a | $C_5H_4{}^iPr$ | $C_5H_4{}^iPr$ | 38.816 | 38.738 |
| 2 | TOJVEW_2b | $C_5H_4{}^iPr$ | $C_5H_4{}^iPr$ | 38.119 | 38.080 |
| 2 | FUXSOI_1a | $C_5H_4Me$ | $C_5H_4Me$ | 40.441 | 40.364 |
| 2 | FUXSOI_2a | $C_5H_4Me$ | $C_5H_4Me$ | 40.480 | 40.402 |
| 2 | FUXSOI_3a | $C_5H_4Me$ | $C_5H_4Me$ | 40.441 | 40.402 |
| 2 | FUXZUV_1a | $C_5H_4Me$ | $C_5H_4Me$ | 40.867 | 40.751 |
| 2 | FUXZUV_2a | $C_5H_4Me$ | $C_5H_4Me$ | 40.402 | 40.093 |
| 2 | FUXZUV_3a | $C_5H_4Me$ | $C_5H_4Me$ | 40.132 | 39.938 |
| 2 | HUKLAC_1a | $C_5H_4Me$ | $C_5H_4Me$ | 41.022 | 40.867 |
| 2 | HUKLAC_2a | $C_5H_4Me$ | $C_5H_4Me$ | 40.983 | 40.867 |
| 2 | HUKLAC_3a | $C_5H_4Me$ | $C_5H_4Me$ | 41.409 | 41.370 |
| 2 | HUKLEG_1a | $C_5H_4Me$ | $C_5H_4Me$ | 43.111 | 42.879 |
| 2 | HUKLEG_2a | $C_5H_4Me$ | $C_5H_4Me$ | 43.576 | 43.498 |
| 2 | HUKLEG_3a | $C_5H_4Me$ | $C_5H_4Me$ | 43.034 | 42.957 |

| | | | | | |
|---|---|---|---|---|---|
| 2 | TEFZOV_1a | $C_5H_4Me$ | $C_5H_4Me$ | 41.563 | 41.525 |
| 2 | TEFZOV_1b | $C_5H_4Me$ | $C_5H_4Me$ | 41.409 | 41.409 |
| 2 | YAFQOO_1a | $C_5H_4Me$ | $C_5H_4Me$ | 39.938 | 39.667 |
| 2 | YAFQOO_2a | $C_5H_4Me$ | $C_5H_4Me$ | 39.667 | 39.628 |
| 2 | YAFQOO_3a | $C_5H_4Me$ | $C_5H_4Me$ | 40.170 | 39.861 |
| 2 | YAJHUP_1a | $C_5H_4Me$ | $C_5H_4Me$ | 40.209 | 39.977 |
| 2 | YAJHUP_2a | $C_5H_4Me$ | $C_5H_4Me$ | 39.783 | 39.745 |
| 2 | YAJHUP_3a | $C_5H_4Me$ | $C_5H_4Me$ | 39.977 | 39.938 |
| 2 | ZAHWUD_1a | $C_5H_4Me$ | $C_5H_4Me$ | 42.337 | 42.299 |
| 2 | ZAHWUD_2a | $C_5H_4Me$ | $C_5H_4Me$ | 42.608 | 42.570 |
| 2 | ZAHWUD_3a | $C_5H_4Me$ | $C_5H_4Me$ | 42.647 | 42.647 |
| 2 | VINVIY_1a | $C_5H_4SiMe_2{}^tBu$ | $C_5H_4SiMe_2{}^tBu$ | 34.752 | 33.978 |
| 2 | VINVUK_1a | $C_5H_4SiMe_2{}^tBu$ | $C_5H_4SiMe_2{}^tBu$ | 33.901 | 33.707 |
| 2 | CEBNAB_1a | $C_5H_4SiMe_3$ | $C_5H_4SiMe_3$ | 33.359 | 32.392 |
| 2 | GUXQEY_1a | $C_5H_4SiMe_3$ | $C_5H_4SiMe_3$ | 34.133 | 33.166 |
| 2 | GUXQIC_1a | $C_5H_4SiMe_3$ | $C_5H_4SiMe_3$ | 33.553 | 32.546 |
| 2 | KEGVEB_1a | $C_5H_4SiMe_3$ | $C_5H_4SiMe_3$ | 35.410 | 35.255 |
| 2 | KEGVEB_2a | $C_5H_4SiMe_3$ | $C_5H_4SiMe_3$ | 34.791 | 34.752 |
| 2 | KEGVIF_1a | $C_5H_4SiMe_3$ | $C_5H_4SiMe_3$ | 35.294 | 34.675 |
| 2 | KEGVIF_2a | $C_5H_4SiMe_3$ | $C_5H_4SiMe_3$ | 35.913 | 35.526 |
| 2 | KEGVOL_1a | $C_5H_4SiMe_3$ | $C_5H_4SiMe_3$ | 33.243 | 32.392 |
| 2 | KEGVOL_2a | $C_5H_4SiMe_3$ | $C_5H_4SiMe_3$ | 34.868 | 34.791 |
| 2 | KEGVOL_3a | $C_5H_4SiMe_3$ | $C_5H_4SiMe_3$ | 32.663 | 31.695 |
| 2 | KEGVOL_4a | $C_5H_4SiMe_3$ | $C_5H_4SiMe_3$ | 33.437 | 32.585 |
| 2 | ZOMTON_1a | $C_5H_4SiMe_3$ | $C_5H_4SiMe_3$ | 34.752 | 34.211 |
| 2 | ZOMTON_2a | $C_5H_4SiMe_3$ | $C_5H_4SiMe_3$ | 35.410 | 34.714 |
| 2 | ZOMTON01_1a | $C_5H_4SiMe_3$ | $C_5H_4SiMe_3$ | 35.642 | 34.714 |
| 2 | PAPMOI_1a | $C_5H_4{}^tBu$ | $C_5H_4{}^tBu$ | 34.559 | 34.365 |
| 2 | DEKBEB_1a | $C_5H_5$ | $C_5H_5$ | 44.853 | 44.853 |
| 2 | DUFCOW10_1a | $C_5H_5$ | $C_5H_5$ | 43.073 | 43.073 |
| 2 | DUFCOW10_2a | $C_5H_5$ | $C_5H_5$ | 43.189 | 43.150 |
| 2 | DUFCOW10_3a | $C_5H_5$ | $C_5H_5$ | 43.073 | 43.073 |
| 2 | DUFDEN11_1a | $C_5H_5$ | $C_5H_5$ | 42.337 | 42.337 |
| 2 | MAMZOS_1a | $C_5H_5$ | $C_5H_5$ | 44.582 | 44.582 |
| 2 | MAMZOS_1b | $C_5H_5$ | $C_5H_5$ | 45.550 | 45.550 |
| 2 | NAPHAN_1a | $C_5H_5$ | $C_5H_5$ | 45.511 | 45.046 |
| 2 | TANSUX_1a | $C_5H_5$ | $C_5H_5$ | 45.588 | 45.550 |

| | | | | | |
|---|---|---|---|---|---|
| 2 | TESJEH_1a | $C_5H_5$ | $C_5H_5$ | 44.389 | 44.389 |
| 2 | VEBYEH_1a | $C_5H_5$ | $C_5H_5$ | 43.731 | 43.731 |
| 2 | XAMBOE_1a | $C_5H_5$ | $C_5H_5$ | 42.337 | 42.337 |
| 2 | XAMCAR_1a | $C_5H_5$ | $C_5H_5$ | 44.621 | 44.543 |
| 2 | LEPGIZ_1a | $C_5HMe_4$ | $C_5HMe_5$ | 35.720 | 35.681 |
| 2 | LEPGIZ_2a | $C_5HMe_4$ | $C_5HMe_5$ | 35.720 | 35.565 |
| 2 | XANPAH_1a | $C_5HMe_4$ | $C_5HMe_5$ | 34.752 | 34.636 |
| 2 | XANPAH_2a | $C_5HMe_4$ | $C_5HMe_5$ | 35.062 | 34.946 |
| 2 | BATSEY_1a | $C_5Me_5$ | $C_5Me_5$ | 35.565 | 34.559 |
| 2 | EMUHAY_1a | $C_5Me_5$ | $C_5Me_5$ | 32.740 | 32.740 |
| 2 | EMUHAY_1b | $C_5Me_5$ | $C_5Me_5$ | 31.927 | 31.656 |
| 2 | EQUQAK_1a | $C_5Me_5$ | $C_5Me_5$ | 30.689 | 30.650 |
| 2 | ETOJOP_1a | $C_5Me_5$ | $C_5Me_5$ | 32.546 | 32.353 |
| 2 | ETOJOP_2a | $C_5Me_5$ | $C_5Me_5$ | 33.553 | 33.088 |
| 2 | ETOJOP_2b | $C_5Me_5$ | $C_5Me_5$ | 31.153 | 31.153 |
| 2 | ETOKEG_1a | $C_5Me_5$ | $C_5Me_5$ | 33.437 | 33.204 |
| 2 | ETOKEG_2a | $C_5Me_5$ | $C_5Me_5$ | 32.933 | 32.546 |
| 2 | ETOKEG_2b | $C_5Me_5$ | $C_5Me_5$ | 33.553 | 33.553 |
| 2 | FAKJEK_1a | $C_5Me_5$ | $C_5Me_5$ | 32.005 | 31.308 |
| 2 | FAKJEK_1b | $C_5Me_5$ | $C_5Me_5$ | 34.172 | 34.172 |
| 2 | FAKJEK_2a | $C_5Me_5$ | $C_5Me_5$ | 34.404 | 34.404 |
| 2 | FAKJEK_2b | $C_5Me_5$ | $C_5Me_5$ | 30.495 | 29.837 |
| 2 | FAKJEK_2c | $C_5Me_5$ | $C_5Me_5$ | 31.076 | 30.573 |
| 2 | FAKJEK_2d | $C_5Me_5$ | $C_5Me_5$ | 32.933 | 32.933 |
| 2 | FAKJIO_1a | $C_5Me_5$ | $C_5Me_5$ | 32.237 | 31.540 |
| 2 | FAKJIO_2a | $C_5Me_5$ | $C_5Me_5$ | 32.895 | 32.276 |
| 2 | FAKJIO_2b | $C_5Me_5$ | $C_5Me_5$ | 31.463 | 30.882 |
| 2 | FAKJOU_1a | $C_5Me_5$ | $C_5Me_5$ | 31.772 | 31.772 |
| 2 | FAKJOU_1b | $C_5Me_5$ | $C_5Me_5$ | 32.469 | 32.469 |
| 2 | FAKJOU_2a | $C_5Me_5$ | $C_5Me_5$ | 32.663 | 32.430 |
| 2 | FAKJOU_2b | $C_5Me_5$ | $C_5Me_5$ | 32.121 | 32.121 |
| 2 | FAKKEL_1a | $C_5Me_5$ | $C_5Me_5$ | 32.856 | 31.889 |
| 2 | FAKKEL_1b | $C_5Me_5$ | $C_5Me_5$ | 32.546 | 32.546 |
| 2 | FAKKEL_1c | $C_5Me_5$ | $C_5Me_5$ | 31.734 | 31.231 |
| 2 | FAKKEL_1d | $C_5Me_5$ | $C_5Me_5$ | 33.050 | 33.050 |
| 2 | FAKKEL_2a | $C_5Me_5$ | $C_5Me_5$ | 31.811 | 31.618 |
| 2 | FAKKEL_2b | $C_5Me_5$ | $C_5Me_5$ | 33.824 | 33.824 |

| | | | | | |
|---|---|---|---|---|---|
| 2 | FAKKEL_2c | $C_5Me_5$ | $C_5Me_5$ | 30.070 | 29.915 |
| 2 | FAKKEL_2d | $C_5Me_5$ | $C_5Me_5$ | 36.726 | 36.726 |
| 2 | FAKKEL_2e | $C_5Me_5$ | $C_5Me_5$ | 27.515 | 27.399 |
| 2 | FAKKEL_2f | $C_5Me_5$ | $C_5Me_5$ | 30.224 | 30.108 |
| 2 | HULRAK_1a | $C_5Me_5$ | $C_5Me_5$ | 33.475 | 33.320 |
| 2 | HUMBID_1a | $C_5Me_5$ | $C_5Me_5$ | 33.282 | 33.050 |
| 2 | IWUWEE_1a | $C_5Me_5$ | $C_5Me_5$ | 34.211 | 34.017 |
| 2 | IWUWII_1a | $C_5Me_5$ | $C_5Me_5$ | 34.211 | 34.056 |
| 2 | IWUWII_2a | $C_5Me_5$ | $C_5Me_5$ | 33.707 | 33.359 |
| 2 | IWUWOO_1a | $C_5Me_5$ | $C_5Me_5$ | 33.359 | 33.011 |
| 2 | IWUWUU_1a | $C_5Me_5$ | $C_5Me_5$ | 33.940 | 33.785 |
| 2 | IWUWUU_2a | $C_5Me_5$ | $C_5Me_5$ | 34.056 | 33.553 |
| 2 | IWUXAB_1a | $C_5Me_5$ | $C_5Me_5$ | 34.598 | 34.443 |
| 2 | IWUXAB_2a | $C_5Me_5$ | $C_5Me_5$ | 34.443 | 34.172 |
| 2 | JUHGIF_1a | $C_5Me_5$ | $C_5Me_5$ | 31.811 | 31.076 |
| 2 | KEPREF_1a | $C_5Me_5$ | $C_5Me_5$ | 32.005 | 31.850 |
| 2 | KEPREF_1b | $C_5Me_5$ | $C_5Me_5$ | 32.159 | 32.005 |
| 2 | KEPREF_2a | $C_5Me_5$ | $C_5Me_5$ | 31.927 | 31.927 |
| 2 | KEPREF_2b | $C_5Me_5$ | $C_5Me_5$ | 31.889 | 31.579 |
| 2 | KEPRUV_1a | $C_5Me_5$ | $C_5Me_5$ | 33.514 | 33.320 |
| 2 | MAYXUI_1a | $C_5Me_5$ | $C_5Me_5$ | 34.094 | 33.746 |
| 2 | MAYXUI_1b | $C_5Me_5$ | $C_5Me_5$ | 34.481 | 34.133 |
| 2 | MAYXUI_1c | $C_5Me_5$ | $C_5Me_5$ | 34.133 | 33.785 |
| 2 | MAYXUI_1d | $C_5Me_5$ | $C_5Me_5$ | 34.598 | 34.598 |
| 2 | MAYXUI_2a | $C_5Me_5$ | $C_5Me_5$ | 34.752 | 34.752 |
| 2 | QAMVOT_1a | $C_5Me_5$ | $C_5Me_5$ | 34.520 | 34.288 |
| 2 | QAMVOT_2a | $C_5Me_5$ | $C_5Me_5$ | 35.217 | 35.217 |
| 2 | TOJVIA_1a | $C_5Me_5$ | $C_5Me_5$ | 35.449 | 35.101 |
| 2 | UHUTOJ_1a | $C_5Me_5$ | $C_5Me_5$ | 32.198 | 31.966 |
| 2 | UHUTUP_1a | $C_5Me_5$ | $C_5Me_5$ | 33.437 | 32.430 |
| 2 | XARFII_1a | $C_5Me_5$ | $C_5Me_5$ | 32.508 | 32.314 |
| 2 | XARFII_2a | $C_5Me_5$ | $C_5Me_5$ | 32.779 | 32.237 |
| 2 | YEZBOW_1a | $C_5Me_5$ | $C_5Me_5$ | 31.540 | 31.540 |
| 2 | YEZBOW_1b | $C_5Me_5$ | $C_5Me_5$ | 33.011 | 33.011 |
| 2 | YEZBOW_1c | $C_5Me_5$ | $C_5Me_5$ | 32.198 | 32.198 |
| 2 | YEZBOW_1d | $C_5Me_5$ | $C_5Me_5$ | 31.966 | 31.966 |
| 2 | PUMJUE_1a | Indene | Indene | 39.474 | 39.474 |
| 3 | VACHUF_1a | $C_5H_4Me$ | $C_5H_4Me$ | 41.409 | 40.983 |

| | | | | | |
|---|---|---|---|---|---|
| 3 | WEDVIN_1a | $C_5H_4Me$ | $C_5H_4Me$ | 41.834 | 41.409 |
| 3 | WEDVIN_2a | $C_5H_4Me$ | $C_5H_4Me$ | 38.622 | 38.622 |
| 3 | WEDVIN_2b | $C_5H_4Me$ | $C_5H_4Me$ | 43.498 | 43.073 |
| 3 | WEDVUZ_1a | $C_5H_4Me$ | $C_5H_4Me$ | 41.293 | 41.293 |
| 3 | WEDVUZ_2a | $C_5H_4Me$ | $C_5H_4Me$ | 41.718 | 41.525 |
| 3 | AKUHUK_1a | $C_5H_5$ | $C_5H_5$ | 45.898 | 45.898 |
| 3 | AKUKAT_1a | $C_5H_5$ | $C_5H_5$ | 45.163 | 45.163 |
| 3 | BASQIZ_1a | $C_5H_5$ | $C_5H_5$ | 42.686 | 42.647 |
| 3 | BEXLIA_1a | $C_5H_5$ | $C_5H_5$ | 44.853 | 44.853 |
| 3 | BIFNEK_1a | $C_5H_5$ | $C_5H_5$ | 45.046 | 45.046 |
| 3 | FIGXEZ_1a | $C_5H_5$ | $C_5H_5$ | 44.466 | 44.350 |
| 3 | IMOXAJ_1a | $C_5H_5$ | $C_5H_5$ | 44.427 | 44.427 |
| 3 | JEXREK_1a | $C_5H_5$ | $C_5H_5$ | 44.543 | 44.543 |
| 3 | JEXREK_2a | $C_5H_5$ | $C_5H_5$ | 44.814 | 44.814 |
| 3 | JEXREK_3a | $C_5H_5$ | $C_5H_5$ | 45.085 | 44.969 |
| 3 | JEXREK_4a | $C_5H_5$ | $C_5H_5$ | 43.189 | 43.189 |
| 3 | MAGFAD_1a | $C_5H_5$ | $C_5H_5$ | 43.885 | 43.885 |
| 3 | MAGFAD_2a | $C_5H_5$ | $C_5H_5$ | 43.266 | 43.228 |
| 3 | MECCUT_1a | $C_5H_5$ | $C_5H_5$ | 44.543 | 44.505 |
| 3 | OCOMUQ_1a | $C_5H_5$ | $C_5H_5$ | 42.918 | 42.918 |
| 3 | OCOMUQ_2a | $C_5H_5$ | $C_5H_5$ | 42.957 | 42.957 |
| 3 | ROCKUR_1a | $C_5H_5$ | $C_5H_5$ | 44.969 | 44.969 |
| 3 | TESHEF_1a | $C_5H_5$ | $C_5H_5$ | 44.698 | 44.698 |
| 3 | TESHEF_2a | $C_5H_5$ | $C_5H_5$ | 44.427 | 44.350 |
| 3 | TESHOP_1a | $C_5H_5$ | $C_5H_5$ | 44.582 | 44.582 |
| 3 | LEPHAS_1a | $C_5HMe_4$ | $C_5HMe_5$ | 38.777 | 38.622 |
| 3 | LEPHAS_2a | $C_5HMe_4$ | $C_5HMe_5$ | 39.319 | 39.241 |
| 3 | LEPHOG_1a | $C_5HMe_4$ | $C_5HMe_5$ | 37.268 | 37.036 |
| 3 | LEPHOG_1b | $C_5HMe_4$ | $C_5HMe_5$ | 38.158 | 38.003 |
| 3 | LEPJEY_1a | $C_5HMe_4$ | $C_5HMe_5$ | 35.797 | 35.797 |
| 3 | LEPJUO_1a | $C_5HMe_4$ | $C_5HMe_5$ | 33.862 | 33.707 |
| 3 | MAVKED_1a | $C_5HMe_4$ | $C_5HMe_5$ | 36.494 | 36.494 |
| 3 | VACHIT_1a | $C_5HMe_4$ | $C_5HMe_5$ | 36.997 | 36.920 |
| 3 | HOLJEZ_1a | $C_5Me_5$ | $C_5Me_5$ | 36.958 | 36.958 |
| 3 | HOLJEZ_2a | $C_5Me_5$ | $C_5Me_5$ | 36.262 | 35.836 |
| 3 | HOLJUP_1a | $C_5Me_5$ | $C_5Me_5$ | 35.913 | 35.913 |
| 3 | HOLJUP_2a | $C_5Me_5$ | $C_5Me_5$ | 36.765 | 36.765 |

| | | | | | |
|---|---|---|---|---|---|
| 3 | HOLJUP_2b | $C_5Me_5$ | $C_5Me_5$ | 35.875 | 35.372 |
| 3 | HOLJUP_2c | $C_5Me_5$ | $C_5Me_5$ | 35.217 | 35.217 |
| 3 | HOLJUP_2d | $C_5Me_5$ | $C_5Me_5$ | 37.152 | 37.152 |
| 3 | VUQHIB_1a | $C_5Me_5$ | $C_5Me_5$ | 35.139 | 34.830 |
| 3 | XARFUU_1a | $C_5Me_5$ | $C_5Me_5$ | 32.779 | 32.779 |
| 3 | XARGEF_1a | $C_5Me_5$ | $C_5Me_5$ | 33.437 | 33.127 |
| 3 | YEZCOX_1a | $C_5Me_5$ | $C_5Me_5$ | 33.282 | 33.088 |
| 4 | FOYMUD_1a | $C_5H_4Me$ | $C_5H_4Me$ | 42.763 | 42.763 |
| 4 | MEXVUG_1a | $C_5H_4Me$ | $C_5H_4Me$ | 41.409 | 40.828 |
| 4 | CEBLIH_1a | $C_5H_4SiMe_3$ | $C_5H_4SiMe_3$ | 37.345 | 37.307 |
| 4 | CUKYOY_1a | $C_5H_4SiMe_3$ | $C_5H_4SiMe_3$ | 37.616 | 37.577 |
| 4 | DANPEN_1a | $C_5H_5$ | $C_5H_5$ | 44.002 | 44.002 |
| 4 | JEXQIN_1a | $C_5H_5$ | $C_5H_5$ | 43.189 | 43.189 |
| 4 | MECDAA_1a | $C_5H_5$ | $C_5H_5$ | 43.653 | 43.653 |
| 4 | OHUYUM_1a | $C_5H_5$ | $C_5H_5$ | 44.659 | 44.659 |
| 4 | ROCJEA_1a | $C_5H_5$ | $C_5H_5$ | 46.362 | 46.362 |
| 4 | WEDHUJ_1a | $C_5H_5$ | $C_5H_5$ | 45.317 | 45.317 |
| 4 | WEDHUJ_2a | $C_5H_5$ | $C_5H_5$ | 45.433 | 45.433 |
| 4 | WEYWII_1a | $C_5H_5$ | $C_5H_5$ | 46.594 | 46.594 |
| 4 | XILDON_1a | $C_5H_5$ | $C_5H_5$ | 44.659 | 44.621 |
| 4 | DOMLUP_1a | $C_5Me_5$ | $C_5Me_5$ | 31.695 | 30.689 |
| 4 | DOMLUP_1b | $C_5Me_5$ | $C_5Me_5$ | 31.889 | 31.618 |
| 4 | VAGMOJ_1a | $C_5Me_5$ | $C_5Me_5$ | 31.966 | 31.966 |
| 4 | VAGMOJ_1b | $C_5Me_5$ | $C_5Me_5$ | 32.546 | 32.546 |
| 4 | VAGMOJ_2a | $C_5Me_5$ | $C_5Me_5$ | 32.546 | 32.546 |
| 4 | VAGMOJ_2b | $C_5Me_5$ | $C_5Me_5$ | 32.663 | 32.624 |
| 4 | VAGMOJ_2c | $C_5Me_5$ | $C_5Me_5$ | 32.508 | 32.508 |
| 4 | VAGMOJ_2d | $C_5Me_5$ | $C_5Me_5$ | 32.624 | 32.585 |
| 4 | VAGMOJ_2e | $C_5Me_5$ | $C_5Me_5$ | 35.294 | 35.294 |
| 4 | VAGMOJ_2f | $C_5Me_5$ | $C_5Me_5$ | 35.372 | 35.372 |
| 4 | VAGMOJ_2g | $C_5Me_5$ | $C_5Me_5$ | 35.294 | 35.294 |
| 4 | VAGMOJ_2h | $C_5Me_5$ | $C_5Me_5$ | 35.372 | 35.372 |
| 4 | VAGMOJ_2i | $C_5Me_5$ | $C_5Me_5$ | 33.824 | 32.895 |
| 4 | VAGMOJ_2j | $C_5Me_5$ | $C_5Me_5$ | 33.824 | 32.895 |
| 4 | VAGMOJ_2k | $C_5Me_5$ | $C_5Me_5$ | 33.785 | 32.856 |
| 4 | VAGMOJ_2l | $C_5Me_5$ | $C_5Me_5$ | 33.785 | 32.856 |
| 4 | VAGMOJ_2m | $C_5Me_5$ | $C_5Me_5$ | 34.559 | 34.559 |

| | | | | | |
|---|---|---|---|---|---|
| 4 | VAGMOJ_2n | $C_5Me_5$ | $C_5Me_5$ | 34.481 | 34.481 |
| 4 | VAGMOJ_2o | $C_5Me_5$ | $C_5Me_5$ | 34.598 | 34.598 |
| 4 | VAGMOJ_2p | $C_5Me_5$ | $C_5Me_5$ | 34.636 | 34.636 |
| 4 | VUQHIB_2a | $C_5Me_5$ | $C_5Me_5$ | 34.907 | 34.714 |
| 4 | YEZCIR_1a | $C_5Me_5$ | $C_5Me_5$ | 35.062 | 34.946 |
| 4 | YEZCIR_1a | $C_5Me_5$ | $C_5Me_5$ | 34.985 | 34.520 |

**Table S3.** *AtomAccess* analysis on $\{Dy(Cp^{ttt})(Cp^{*})\}^{+}$ fragments reported herein

| Parent complex | Largest cluster | Total % Unblocked |
|---|---|---|
| | (% SA) | (% SA) |
| **2-Dy** | 20.046 | 22.098 |
| **3-Dy·$C_6H_6$** | 12.229 | 15.441 |
| **4-Dy** | 13.158 | 16.254 |
| **5%Dy@4-Y** | 14.977 | 17.067 |

*1.3 Conformational Analysis*

Conformers of **A**–**F** were generated in *OpenBabel* 2.4.1[7] using a genetic search algorithm to generate diverse conformers,[8] optimized for maximum root-mean-squared-distance (RMSD) diversity. Ten thousand conformers were requested to capture all possible conformations (144–700 conformers generated per initial geometry), with 20 or 50 children and a 0.2 mutation probability in each generation. The algorithm considers rotation of dihedral angles and applies a steric filter to remove conformations with atoms too close together. Bond lengths are fixed, and only rotations that change non-hydrogen atom positions are considered.

Initial geometries were constructed from two $Dy(C_5)_2$ sets of coordinates (Table S4). The $C_5$ bond distances and angles were taken from the crystal structure of $[Dy(Cp^{ttt})_2][B(C_6F_5)_4]{\cdot}CH_2Cl_2$, with the $Dy{\cdots}Cp_{centroid}$ distances modified to the median XRD value of 2.375 Å (Figure S10) and the $Cp_{centroid}{\cdots}Dy{\cdots}Cp_{centroid}$ angle changed to 180°. One of the $Dy(C_5)_2$ frames is eclipsed (as in the original structure) and the other is staggered and has $C_5$ rings perpendicular to $Dy{\cdots}Cp_{cent}$. The appropriate substituents for molecules **A**–**F** were appended to the $Dy(C_5)_2$ framework in GaussView,[9] using standard geometries (C–C 1.54 Å, C–H 1.07 Å). The $^tBu$ geometries in $Cp^{ttt}$ were taken directly from the crystal structure of $[Dy(Cp^{ttt})_2][B(C_6F_5)_4]{\cdot}CH_2Cl_2$. In structures with methyl groups attached to the Cp ring, initial geometries were provided with C–H bonds at 90° or –90° to the $C_5$ plane.

*AtomAccess* was used to calculate the size of the largest cluster and the total % unblocked for all conformations of **A**–**F** with $\rho$ = 3–15. Discussion of *AtomAccess* results assumes $\rho$ = 10; the data from other densities were used to assess the error in the total % unblocked for various $\rho$ (see §1.1). Given the large numbers of conformations we have included only summary data and comparison to *AtomAccess* calculations on XRD fragments in Table S5.

**Table S4.** Coordinates of $Dy(C_5)_2$ frames used to build $\{Dy(Cp^{R1})(Cp^{R2}\}^+$ for conformation analysis

| | | | |
|---|---|---|---|
| Staggered initial coordinates | | | |
| Dy | 0 | 0 | 0 |
| C | –2.4068119 | 0.8513686 | –0.6806020 |
| C | –1.8301132 | 1.8597859 | 0.1742614 |
| C | –1.7630326 | 1.2982263 | 1.4787526 |
| C | –2.6791427 | –0.2706748 | 0.1504125 |
| C | –2.2956995 | –0.0197293 | 1.4730582 |
| C | 2.6358952 | 0.3139243 | –0.2212091 |
| C | 2.4803214 | –0.7611628 | 0.6755050 |
| C | 2.1980881 | –0.0464124 | –1.5231882 |
| C | 1.9115765 | –1.8117075 | –0.0816708 |
| C | 1.7489186 | –1.4136182 | –1.4453197 |
| Eclipsed initial coordinates | | | |
| Dy | 0 | 0 | 0 |
| C | –2.4008314 | 0.8760871 | –0.6702771 |
| C | –1.8095814 | 1.8791371 | 0.1809528 |
| C | –1.7423814 | 1.3180771 | 1.4856528 |
| C | –2.6811214 | –0.2422729 | 0.1630528 |
| C | –2.2889314 | 0.0058071 | 1.4836729 |
| C | 2.5284934 | 0.3114951 | –0.8746629 |
| C | 2.7096724 | –0.1991558 | 0.4255377 |
| C | 2.1462825 | –1.4963647 | 0.4090727 |
| C | 1.6531521 | –1.8105110 | –0.8957665 |
| C | 1.8852464 | –0.6422992 | –1.7072354 |

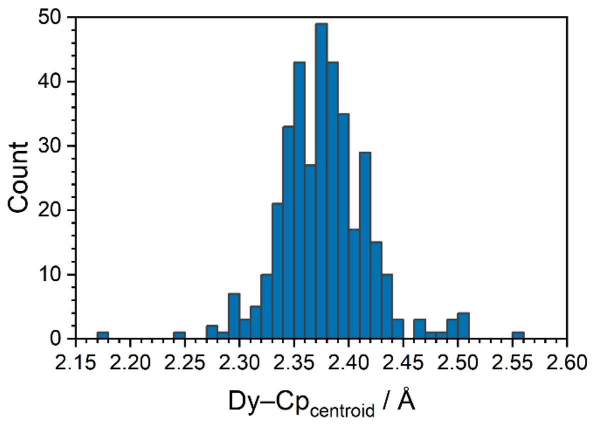


**Figure S10.** Distribution of Dy···$Cp^R_{centroid}$ distances in unique $\{Dy(C_5)_2\}$ fragments in the CSD. Median value is 2.375 Å.

**Table S5.** Comparison of largest cluster distributions (% SA), $Cp_{centroid}\cdots Dy\cdots Cp_{centroid}$ angles and number of structures for $\{Dy(Cp^{R1})(Cp^{R2}\}^+$ XRD fragments, $\{Dy(Cp^{ttt})(Cp^*)\}^+$ fragments reported herein, and simulated conformations.

| | Fragment | **A** | **B** | **C** | **D** | **E** | **F** |
|---|---|---|---|---|---|---|---|
| Largest Cluster (% SA) | XRD minimum | 42.3 | 38.6 | 27.4 | 12.3 | 2.6 | 2.1 |
| | XRD mean | 44.3 | 41.1 | 33.1 | 15.1[a] | 9.5[a] | 15.2 |
| | XRD maximum | 46.6 | 43.5 | 37.2 | 20.0 | 19.7 | 24.4 |
| | Conformer minimum | 42.7 | 39.3 | 24.1 | 7.0 | 2.4 | 3.7 |
| | Conformer mean | 43.5 | 40.3 | 27.0 | 16.4 | 12.6 | 8.2 |
| | Conformer maximum | 45.5 | 42.2 | 31.5 | 22.4 | 21.8 | 15.0 |
| $Cp_{centroid}$–Dy–$Cp_{centroid}$ (°) | XRD minimum | 123.4 | 121.2 | 131.4 | 145.0 | 150.3 | 138.7 |
| | XRD mean | 128.5 | 127.3 | 138.4 | 147.5[a] | 158.4[a] | 147.3 |
| | XRD maximum | 133.4 | 132.6 | 149.4 | 149.1 | 162.5 | 152.9 |
| | Conformer minimum | 108.1 | 108.1 | 123.9 | 125.6 | 129.4 | 119.3 |
| | Conformer mean | 143.7 | 143.7 | 152.5 | 158.1 | 161.4 | 153.0 |
| | Conformer maximum | 179.6 | 179.6 | 179.6 | 179.6 | 179.6 | 175.4 |
| Number of XRD fragments | | 42 | 31 | 99 | 3 | 3 | 16 |
| Number of conformers | | 288 | 864 | 585 | 1076 | 1950 | 931 |

*a* Not statistically significant because of the low number of XRD fragments

## 2. General Synthetic Methods

All manipulations were performed in an inert atmosphere (argon) with rigorous exclusion of oxygen and water using Schlenk line and glovebox techniques. THF and toluene were dried over columns charged with alumina, *n*-hexane was dried by refluxing over potassium, fluorobenzene was dried by stirring over alumina and anhydrous benzene was purchased from Sigma-Aldrich. THF, fluorobenzene and benzene were stored over 4 Å molecular sieves, whilst toluene and *n*-hexane were stored over potassium mirrors. All solvents were degassed before use. For NMR spectroscopy $C_6D_6$ and $C_4D_8O$ were dried by refluxing over K, then vacuum transferred and degassed by three freeze-pump-thaw cycles before use. The reagents $[Y(BH_4)_3(THF)_3]$,[10] $[Dy(BH_4)_3(THF)_3]$,[10] $KCp^{ttt}$,[11] KCp*,[12] $[HNEt_3][Al\{OC(CF_3)_3\}_4]$,[13] $[(SiEt_3)_2(\mu\text{-}H)][B(C_6F_5)_4]$[14,15] and $[CPh_3][B(C_6F_5)_4]$[16] were synthesized according to literature procedures. $^{1}H$ (400 and 500 MHz), $^{13}C$ (126 MHz), $^{11}B$ (128 MHz**)** and $^{19}F$ (376 MHz) NMR spectra were obtained on a Bruker Avance III 400 or 500 MHz spectrometer at 298 K and were referenced to the solvent used, to external $SiMe_4$ ($^{1}H$, $^{13}C$), $H_3BO_3/D_2O$ ($^{11}B$) or $C_7H_5F_3/CDCl_3$ ($^{19}F$). ATR-IR spectra were recorded on a Bruker Alpha spectrometer with a Platinum-ATR module. Elemental analysis was carried out by Mr Martin Jennings and Mrs Anne Davies at the Microanalytical service, Department of Chemistry, the University of Manchester. Elemental analysis results for **1-4-Ln** frequently showed lower carbon values than expected; this was ascribed to a combination of the experimental conditions employed[17] and carbide formation, which is particularly common for fluorine-rich complexes like **3-Ln** and **4-Ln**.[18]

## 3. Synthesis

**[Y(Cp$^{ttt}$)(BH$_4$)$_2$(THF)] (1-Y).** THF (50 mL) was added to a mixture of [Y(BH$_4$)$_3$(THF)$_3$] (1.7488 g, 5.0 mmol) and KCp$^{ttt}$ (1.3626 g, 5 mmol). The reaction mixture was stirred at room temperature for 48 h. Following the removal of THF *in vacuo*, toluene (50 mL) was added to extract the product and the resultant light yellow solution was filtered. Volatiles were removed under vacuum to give a light yellow oil, which was dissolved in *n*-hexane (10 mL). The resultant solution was stored at –30 °C overnight to give colorless crystals. The supernatant was decanted and the residual solvent was removed *in vacuo* to afford the product **1-Y** (1.5692 g, 3.7 mmol, 74%). Anal. Calcd for $C_{21}H_{45}B_2OY$: C, 59.47; H, 10.70. Found: C, 56.63; H, 10.75. $^1$H NMR (500.19 MHz, $C_6D_6$, 298 K): δ = 6.40 (s, 2H, Cp-*H*), 3.54 (br s, 4H, THF-C*H*$_2$O), 1.55 (s, 18H, C(C*H*$_3$)$_3$), 1.26 (s, 9H, C(C*H*$_3$)$_3$), 1.04 (br q, 8H, $^1J_{BH}$ = 85.8 Hz, B*H*$_4$), 1.02 (br s, 4H, THF-C*H*$_2$). $^{13}$C{$^1$H} NMR (125.79 MHz, $C_6D_6$, 298 K): δ = 137.9 (Cp-*C*C(CH$_3$)$_3$), 111.4 (Cp-*C*H), 73.8 (THF-*C*H$_2$O), 34.3 (*C*(CH$_3$)$_3$), 34.1 (C(*C*H$_3$)$_3$), 32.8 (*C*(CH$_3$)$_3$), 31.9 (C(*C*H$_3$)$_3$), 24.8 (THF-*C*H$_2$). $^{11}$B{$^1$H} NMR (160.48 MHz, $C_6D_6$, 298 K): δ = –22.72 (*B*H$_4$). $^{11}$B NMR (128.38 MHz, $C_6D_6$, 298 K): δ = –22.74 (p, $^1J_{BH}$ = 85.8 Hz, *B*H$_4$). FTIR (ATR, microcrystalline): $\tilde{\nu}$ = 2957 (s, C-H stretch), 2901 (m, C-H stretch), 2869 (m, C-H stretch), 2464 (s, B-H$_t$ stretch), 2283(w), 2226 (w), 2201 (br m, B-H$_b$ stretch), 2137 (br m, B-H$_b$ stretch), 1459 (m, C-H bending), 1360 (s), 1257 (s), 1237 (s), 1178 (s, B-H$_b$ deformation), 1093 (s, C-O stretch), 1003 (s), 839 (s), 797 (s), 678 (m), 559 (m) cm$^{-1}$.

**[Dy(Cp$^{ttt}$)(BH$_4$)$_2$(THF)] (1-Dy).** Complex **1-Dy** was prepared by following analogous synthetic and work-up procedures to **1-Y** from [Dy(BH$_4$)$_3$(THF)$_3$] (2.1267 g, 5.0 mmol) and KCp$^{ttt}$ (1.3696 g, 5 mmol). The product **1-Dy** was obtained as yellow crystals (1.729 g, 3.5 mmol, 69%). Anal. Calcd for $C_{21}H_{45}B_2DyO$: C, 50.68; H, 9.11. Found: C, 50.49; H, 9.30. $\mu_{eff}$ product = 10.68 $\mu_B$ (Evans method, $C_6D_6$, 298 K). $^1$H NMR (400.09 MHz, $C_6D_6$, 298 K): δ = –

11.05 (br s, 4H, THF-C$H_2$), –33.64 (br s, 9H, $\upsilon_{1/2}$ ~ 600 Hz, C(C$H_3$)$_3$), –83.03 (br s, 4H, $\upsilon_{1/2}$ ~ 620 Hz, THF-C$H_2$), –114.38 (br s, 18H, $\upsilon_{1/2}$ ~ 720 Hz, C(C$H_3$)$_3$). The paramagnetism of **1-Dy** precluded the assignment of its $^{13}$C{$^1$H} and $^{11}$B{$^1$H} NMR spectra. FTIR (ATR, microcrystalline): $\tilde{\nu}$ = 2945 (m, C-H stretch), 2892 (m, C-H stretch), 2429 (m, B-H$_t$ stretch), 2345 (w), 2125 (m, B-H$_b$ stretch), 1424 (m, C-H bending), 1245 (s), 1089 (s, C-O stretch), 997 (m), 997 (m), 820 (s), 754 (s), 684 (m), 627 (m), 506 (m), 425 (m) cm$^{-1}$.

**[Y(Cp$^{ttt}$)(Cp*)(BH$_4$)] (2-Y).** Toluene (20 mL) was added to a mixture of **1-Y** (1.2724 g, 3.0 mmol) and KCp* (0.5230 g, 3.0 mmol). The reaction mixture was heated to reflux with stirring for 16 h to form a light yellow solution and white precipitate. Filtration and removal of volatiles *in vacuo* afforded a light yellow solid. *n*-Hexane (10 mL) was added to re-dissolve the solid, and the solution was concentrated under vacuum to *ca.* 2 mL. Colorless crystals formed overnight when stored at –30 °C. The supernatant was decanted and the crystals dried under vacuum to yield the product **2-Y** (0.9212 g, 2.0 mmol, 65%). Anal. Calcd for $C_{27}H_{48}BY$: C, 68.65; H, 10.24. Found: C, 65.46; H, 10.25. $^1$H NMR (400.13 MHz, $C_6D_6$, 298 K): δ = 6.48 (br s, 2H, Cp-*H*), 2.00 (s, 15H, Cp-C$H_3$), 1.39 (s, 18H, C(C$H_3$)$_3$), 1.27 (br m, 4H, B$H_4$), 1.16 (s, 9H, C(C$H_3$)$_3$). $^{13}$C{$^1$H} NMR (100.60 MHz, $C_6D_6$, 298 K): δ = 135.9 (Cp-*C*C(CH$_3$)$_3$), 122.0 (Cp-*C*(CH$_3$)), 117.9 (Cp-*C*H), 34.2 (*C*(CH$_3$)$_3$), 33.0 (*C*(CH$_3$)$_3$), 32.4 (C(*C*H$_3$)$_3$), 31.4 (C(*C*H$_3$)$_3$), 12.4 (Cp-C(*C*H$_3$)). $^{11}$B{$^1$H} NMR (128.38 MHz, $C_6D_6$, 298 K): δ = –16.74 (*B*H$_4$). $^{11}$B NMR (128.38 MHz, $C_6D_6$, 298 K): δ = –16.72 (br p, $^1J_{BH}$ = 86.3 Hz, *B*H$_4$). FTIR (ATR, microcrystalline): $\tilde{\nu}$ = 2955 (s, C-H stretch), 2904 (s, C-H stretch), 2864 (s, C-H stretch), 2413 (s, B-H$_t$ stretch), 2370 (m, B-H$_t$ stretch), 2238 (w), 2123 (s, B-H$_b$ stretch), 1459 (s, C-H bending), 1358 (s), 1239 (s), 1124 (s, BH$_2$ deformation), 1021 (s), 836 (s), 802 (s), 695 (m), 682 (m), 549 (m), 444 (m) cm$^{-1}$.

**[Dy(Cp$^{ttt}$)(Cp*)(BH$_4$)] (2-Dy).** Complex **2-Dy** was prepared by following analogous synthetic and work-up procedures to **2-Y** from **1-Dy** (1.4931 g, 3.0 mmol) and KCp* (0.5230 g, 3.0 mmol). The product **2-Dy** was obtained as yellow crystals (1.2612 g, 2.3 mmol, 77%). Anal. Calcd for $C_{27}H_{48}BDy$: C, 59.40; H, 8.86. Found: C, 58.34; H, 8.95. $\mu_{eff}$ product = 10.71 $\mu_B$ (Evans method, $C_6D_6$, 298 K). The paramagnetism of **2-Dy** precluded the assignment of its $^1$H, $^{13}$C{$^1$H} and $^{11}$B{$^1$H} NMR spectra. FTIR (ATR, microcrystalline): $\tilde{\nu}$ = 2957 (s, C-H stretch), 2904 (s, C-H stretch), 2867 (C-H stretch), 2411 (s, B-H$_t$ stretch), 2370 (m, B-H$_t$ stretch), 2236 (w), 2121 (s, B-H$_b$ stretch), 1461 (m, C-H bending), 1356 (s), 1269 (s), 1122 (s, $BH_2$ deformation), 1023 (m), 836 (s), 696 (m), 684 (m), 546 (w), 444 (m) cm$^{-1}$.

**[Y(Cp$^{ttt}$)(Cp*)][Al{OC(CF$_3$)$_3$}$_4$] (3-Y).** Complex **2-Y** (0.7558 g, 1.6 mmol) was dissolved in toluene (20 mL). MgCl($C_3H_5$) (0.9 mL, 1.8 mmol, 2.0 M solution in THF) was added dropwise to this solution to form an orange reaction mixture, which was stirred for 3 h. Volatiles were removed *in vacuo* and a 10:1 *n*-hexane:dioxane solution (2 x 30 mL) was added to triturate the reaction mixture, with the volatiles removed *in vacuo* after stirring for 15 min on each occasion. *n*-Hexane (2 x 20 mL) was used to extract the crude product and the solvent was removed under vacuum to afford crude "[Y(Cp$^{ttt}$)(Cp*)($C_3H_5$)]" as a yellow foam (0.7768 g, 1.6 mmol). [NEt$_3$H][Al{OC(CF$_3$)$_3$}$_4$] (1.6253 g, 1.5 mmol) and benzene (15 mL) were added and the reaction mixture was stirred overnight; a biphasic mixture resulted that consisted of a yellow oil and a light yellow solution. Volatiles were removed under vacuum, benzene (20 mL) was added, the mixture was stirred for 15 min and the solvent was removed *in vacuo*; this process was repeated with *n*-hexane (20 mL). The pale yellow solid was washed with benzene (20 mL) and *n*-hexane (2 x 30 mL). Residual volatiles were removed *in vacuo* to afford crude "[{Y(Cp$^{ttt}$)(Cp*)}{Al[OC(CF$_3$)$_3$]$_4$}]" contaminated with a trace amount of triethylamine (*ca*. 0.25 eq.) as a pale yellow powder (1.3404 g, 0.92 mmol, 58%). Benzene (0.5 mL) was added

to an aliquot of “[{Y(Cp$^{ttt}$)(Cp*)}{Al[OC($CF_3$)$_3$]$_4$}]” (0.150 g, 0.10 mmol); the resultant oil was frozen, layered with *n*-hexane (10 mL) and stored at 6 °C to give colorless crystals of **3-Y·$C_6H_6$** (0.104 g, 0.069 mmol, 69%).

Characterization data for “[{Y(Cp$^{ttt}$)(Cp*)}{Al[OC($CF_3$)$_3$]$_4$}]” with 0.25 eq. of $NEt_3$: Anal. Calcd for $C_{44.5}H_{47.75}AlF_{36}NO_4Y$: C, 36.86; H, 3.32; N, 0.24. Found: C, 35.86; H, 3.31; N, 0.13. $^1$H NMR (400.13 MHz, $C_6H_5F$ with a $C_4D_8O$ insert, 298 K): δ = 6.41 (s, 2H, Cp-*H*), 2.64 (q, $^1J_{HH}$ = 7.3 Hz, 1.5H, N(C*H*$_2$CH$_3$)$_3$), 1.86 (s, 15H, Cp-C*H*$_3$), 1.30 (s, 18H, C(C*H*$_3$)$_3$), 1.17 (s, 9H, C(C*H*$_3$)$_3$), 0.92 (t, $^1J_{HH}$ = 7.3 Hz, 3H, N(CH$_2$C*H*$_3$)$_3$). $^{13}$C{$^1$H} NMR (100.60 MHz, $C_6H_5F$ with a $C_4D_8O$ insert, 298 K): δ = 140.1 (Cp-*C*C(CH$_3$)$_3$), 125.7 (Cp-*C*C(CH$_3$)$_3$), 123.2 (Cp-*C*(CH$_3$)), 120.3 (Cp-*C*H), 34.3 (*C*(CH$_3$)$_3$), 32.8 (*C*(CH$_3$)$_3$), 32.3 (C(*C*H$_3$)$_3$), 30.8 (C(*C*H$_3$)$_3$), 11.6 (Cp-C(*C*H$_3$)), 8.2 (N(CH$_2$*C*H$_3$)$_3$). $^{19}$F{$^1$H} NMR (376.46 MHz, $C_6H_5F$ with a $C_6D_6$ insert, 298 K): δ = –75.1 ([Al{OC(C*F*$_3$)$_3$}$_4$]$^-$), –113.3 ($C_6H_5$*F*). $^{19}$F NMR (376.46 MHz, $C_6H_5F$ with a $C_6D_6$ insert, 298 K): δ = –75.2 ([Al{OC(C*F*$_3$)$_3$}$_4$]$^-$), –113.5 (m, $C_6H_5$*F*). FTIR (ATR, microcrystalline): $\tilde{\nu}$ = 2966 (w, C-H stretch), 2920 (w, C-H stretch), 2873 (w, C-H stretch), 1461 (w), 1350 (s, C-O stretch), 1297 (s), 1272 (s, C-F stretch), 1237 (s), 1213 (s), 1161 (s), 1065 (w), 970 (s), 830 (s, Al-O stretch), 756 (w), 725 (s), 690 (w), 559 (s), 536 (s), 440 (s) cm$^{-1}$.

Characterization data for **3-Y·$C_6H_6$**: Anal. Calcd for $C_{49}H_{50}AlF_{36}O_4Y$: C, 39.16; H, 3.35. Found: C, 35.70; H, 3.11. $^1$H NMR (400.13 MHz, $C_6H_5F$ with a $C_6D_6$ insert, 298 K): δ = 6.40 (s, 2H, Cp-*H*), 1.85 (s, 15H, Cp-C*H*$_3$), 1.29 (s, 18H, C(C*H*$_3$)$_3$), 1.17 (s, 9H, C(C*H*$_3$)$_3$). $^{13}$C{$^1$H} NMR (100.60 MHz, $C_6H_5F$ with a $C_6D_6$ insert, 298 K): δ = 140.4 (Cp-*C*C(CH$_3$)$_3$), 126.0 (Cp-*C*C(CH$_3$)$_3$), 123.6 (Cp-*C*(CH$_3$)), 120.6 (Cp-*C*H), 34.6 (*C*(CH$_3$)$_3$), 33.1 (*C*(CH$_3$)$_3$), 32.7 (C(*C*H$_3$)$_3$), 31.1 (C(*C*H$_3$)$_3$), 11.9 (Cp-C(*C*H$_3$)). $^{19}$F{$^1$H} NMR (376.46 MHz, $C_6H_5F$ with a $C_6D_6$ insert, 298 K): δ = –75.2 ([Al{OC(C*F*$_3$)$_3$}$_4$]$^-$), –113.4 ($C_6H_5$*F*). FTIR (ATR, microcrystalline): $\tilde{\nu}$ = 2968 (w, C-H stretch), 2927 (w, C-H stretch), 2875 (w, C-H stretch),

1465 (w), 1352 (s, C-O stretch), 1297 (s), 1274 (s, C-F stretch), 1239 (s), 1213 (s), 1161 (s), 1063 (w), 1021 (w), 970 (s), 830 (s, Al-O stretch), 754 (w), 725 (s), 682 (w), 561 (s), 536 (s), 442 (s) $cm^{-1}$.

**[Dy(Cp$^{ttt}$)(Cp*)][Al{OC($CF_3$)$_3$}$_4$] (3-Dy).** The allyl complex "[Dy(Cp$^{ttt}$)(Cp*)($C_3H_5$)]" (1.0872 g, 1.9 mmol) was prepared as a crude yellow-orange foam by following analogous synthetic and work-up procedures to "[Y(Cp$^{ttt}$)(Cp*)($C_3H_5$)]" from **2-Dy** (1.0920 g, 2.0 mmol) and MgCl($C_3H_5$) (1.1 mL, 2.2 mmol, 2.0 M solution in THF). Following the subsequent addition of [$NEt_3$H][Al{OC($CF_3$)$_3$}$_4$] (1.9247 g, 1.8 mmol) and benzene (15 mL), analogous work up procedures with multiple *n*-hexane/benzene washes were used to obtain crude "[{Dy(Cp$^{ttt}$)(Cp*)}{Al[OC($CF_3$)$_3$]$_4$}]" contaminated with an unknown amount of triethylamine as a yellow powder (2.1575 g, 1.4 mmol, 72%). Benzene (1 mL) was added to an aliquot of "[{Dy(Cp$^{ttt}$)(Cp*)}{Al[OC($CF_3$)$_3$]$_4$}]" (0.250 g, 0.16 mmol); the resultant oil was frozen, layered with *n*-hexane (15 mL) and stored at 6 °C to form yellow-orange crystals of **3-Dy·$C_6H_6$** (0.1882 g, 0.12 mmol, 75%). On one occasion during a recrystallization attempt of "[{Dy(Cp$^{ttt}$)(Cp*)}{Al[OC($CF_3$)$_3$]$_4$}]" at room temperature a small crop of pale yellow crystals of [{Dy(Cp$^{ttt}$)(Cp*)}$_2$($\mu$-F)][Al{OC($CF_3$)$_3$}$_4$] (**6-Dy·$C_6H_{14}$**) were identified by single crystal XRD.

Characterization data for "[{Dy(Cp$^{ttt}$)(Cp*)}{Al[OC($CF_3$)$_3$]$_4$}]": Anal. Calcd for $C_{49}H_{50}AlDyF_{36}O_4$: C, 37.34; H, 3.20; N, 0.00. Found: C, 34.94; H, 3.13; N, 0.19. Assuming a trace amount of $NEt_3$ (0.25 eq. in "[{Dy(Cp$^{ttt}$)(Cp*)}{Al[OC($CF_3$)$_3$]$_4$}]"): Anal. Calcd for $C_{44.5}H_{47.75}AlDyF_{36}NO_4$: C, 35.08; H, 3.16; N, 0.23. Found: C, 34.94; H, 3.13; N, 0.19. The paramagnetism of "[{Dy(Cp$^{ttt}$)(Cp*)}{Al[OC($CF_3$)$_3$]$_4$}]" precluded the assignment of its $^{1}$H, and $^{13}$C{$^{1}$H} NMR spectra. $^{19}$F{$^{1}$H} NMR (376.46 MHz, $C_6H_5F$, 298 K): $\delta$ = –82.2 ([Al{OC(C*F*$_3$)$_3$}$_4$]$^-$), –120.2 ($\upsilon_{1/2}$ ~ 2900 Hz, $C_6H_5$*F*). FTIR (ATR, microcrystalline): $\tilde{\nu}$ = 2968

(w, C-H stretch), 2925 (w, C-H stretch), 2869 (w, C-H stretch), 1352 (s, C-O stretch), 1296 (s), 1274 (s, C-F stretch), 1239 (s), 1211 (s), 1165 (s), 1067 (w), 972 (s), 832 (s, Al-O stretch), 725 (s), 561 (s), 536 (s), 442 (s) $cm^{-1}$.

Characterization data for **3-Dy·$C_6H_6$**: Anal. Calcd for $C_{49}H_{50}AlDyF_{36}O_4$: C, 37.34; H, 3.20. Found: C, 33.99; H, 3.07. The paramagnetism of **3-Dy·$C_6H_6$** precluded the assignment of its $^1$H and $^{13}$C{$^1$H} NMR spectra. $^{19}$F{$^1$H} NMR (376.46 MHz, $C_6H_5F$, 298 K): δ = –82.9 ([Al{OC(C*F*$_3$)$_3$}$_4$]$^-$), –117.2 ($\upsilon_{1/2}$ ~ 1700 Hz, $C_6H_5$*F*). FTIR (ATR, microcrystalline): $\tilde{\nu}$ = 2964 (w, C-H stretch), 2927 (w, C-H stretch), 2871 (w, C-H stretch), 1459 (w), 1340 (w), 1350 (s, C-O stretch), 1297 (s), 1272 (s, C-F stretch), 1237 (s), 1211 (s), 1165 (s), 1093 (m), 1023 (m), 970 (s), 828 (s, Al-O stretch), 754 (w), 725 (s), 686 (w), 561 (s), 536 (s), 444 (s) $cm^{-1}$.

**[Y(Cp$^{ttt}$)(Cp*){Al[OC($CF_3$)$_3$]$_4$-κ-*F*}] (4-Y).** Benzene (0.5 mL) was added to an aliquot of "[{Y(Cp$^{ttt}$)(Cp*)}{Al[OC($CF_3$)$_3$]$_4$}]" (0.200 g, 0.14 mmol); this was frozen, layered with *n*-hexane (10 mL) and stored at 6 °C to obtain **4-Y** as yellow crystals (0.170 g, 0.12 mmol, 85%). Anal. Calcd for $C_{43}H_{44}AlF_{36}O_4Y$: C, 36.25; H, 3.11. Found: C, 35.92; H, 3.16. $^1$H NMR (400.13 MHz, $C_6H_5F$ with a $C_6D_6$ insert, 298 K): δ = 6.41 (s, 2H, Cp-*H*), 1.85 (s, 15H, Cp-C*H*$_3$), 1.31 (s, 18H, C(C*H*$_3$)$_3$), 1.17 (s, 9H, C(C*H*$_3$)$_3$). $^{13}$C{$^1$H} NMR (100.60 MHz, $C_6H_5F$ with a $C_6D_6$ insert, 298 K): δ = 140.4 (Cp-*C*C($CH_3$)$_3$), 126.0 (Cp-*C*C($CH_3$)$_3$), 122.3 (Cp-*C*($CH_3$)), 120.6 (Cp-*C*H), 34.6 (*C*($CH_3$)$_3$), 33.1 (*C*($CH_3$)$_3$), 32.7 (C(*C*H$_3$)$_3$), 31.1 (C(*C*H$_3$)$_3$), 11.9 (Cp-C(*C*H$_3$)). $^{19}$F{$^1$H} NMR (376.46 MHz, $C_6H_5F$ with a $C_6D_6$ insert, 298 K): δ = –74.6 ([Al{OC(C*F*$_3$)$_3$}$_4$]$^-$), –112.9 ($C_6H_5$*F*). FTIR (ATR, microcrystalline): $\tilde{\nu}$ = 2967 (w, C-H stretch), 2927 (w, C-H stretch), 2869 (w, C-H stretch), 1467 (w), 1354 (s, C-O stretch), 1299 (s), 1274 (s, C-F stretch), 1239 (s), 1208 (s), 1165 (s), 1060 (w), 1021 (w), 970 (s), 832 (s, Al-O stretch), 756 (w), 725 (s), 682 (w), 561 (s), 536 (s), 444 (s) $cm^{-1}$.

**[Dy(Cp$^{ttt}$)(Cp*){Al[OC(CF$_3$)$_3$]$_4$-κ-*F*}] (4-Dy).** Benzene (1 mL) was added to an aliquot of "[{Dy(Cp$^{ttt}$)(Cp*)}{Al[OC(CF$_3$)$_3$]$_4$}]" (0.200 g, 0.13 mmol); this was frozen, layered with *n*-hexane (15 mL) and stored at 6 °C to obtain **4-Dy** as yellow-orange crystals (0.162 g, 0.11 mmol, 81%). Anal. Calcd for $C_{43}H_{44}AlDyF_{36}O_4$: C, 34.47; H, 2.98. Found: C, 33.75; H, 3.02. The paramagnetism of **4-Dy** precluded the assignment of its $^1$H, and $^{13}$C{$^1$H} NMR spectra. $^{19}$F{$^1$H} NMR (376.46 MHz, $C_6H_5F$, 298 K): δ = –81.9 ([Al{OC(C*F*$_3$)$_3$}$_4$]$^-$), –119.5 ($\upsilon_{1/2}$ ~ 2400 Hz, $C_6H_5$*F*). FTIR (ATR, microcrystalline): $\tilde{\nu}$ = 2966 (m, C-H stretch), 2918 (w, C-H stretch), 2869 (w, C-H stretch), 1461 (w), 1352 (s, C-O stretch), 1296 (s), 1274 (s, C-F stretch), 1239 (s), 1211 (s), 1165 (s), 1073 (m), 1021 (w), 970 (s), 832 (s, Al-O stretch), 754(w), 725 (s), 561 (s), 536 (s), 442 (s) cm$^{-1}$.

**[5%Dy@Y(Cp$^{ttt}$)(Cp*){Al[OC(CF$_3$)$_3$]$_4$-κ-*F*}] (5%Dy@4-Y).** Benzene (0.5 mL) was added to a mix of "[{Y(Cp$^{ttt}$)(Cp*)}{Al[OC(CF$_3$)$_3$]$_4$}]" (0.135 g, 0.095 mmol) and "[{Dy(Cp$^{ttt}$)(Cp*)}{Al[OC(CF$_3$)$_3$]$_4$}]" (0.007 g, 0.005 mmol); this was frozen, layered with *n*-hexane (10 mL) and stored at 6 °C to obtain **5%@4-Y** as yellow crystals (0.117 g, 0.082 mmol, 82%). Anal. Calcd for $C_{43}H_{44}AlDy_{0.05}F_{36}O_4Y_{0.95}$: C, 36.16; H, 3.11. Found: C, 35.78; H, 3.53. $^1$H NMR (400.13 MHz, $C_6H_5F$ with a $D_2O$ insert, 298 K): δ = 6.66 (s, 2H, Cp-*H*), 2.11 (s, 15H, Cp-C*H*$_3$), 1.55 (s, 18H, C(C*H*$_3$)$_3$), 1.43 (s, 9H, C(C*H*$_3$)$_3$). $^{13}$C{$^1$H} NMR (100.60 MHz, $C_6H_5F$ with a $D_2O$ insert, 298 K): δ = 140.4 (Cp-*C*C(CH$_3$)$_3$), 126.0 (Cp-*C*C(CH$_3$)$_3$), 123.0 (Cp-*C*(CH$_3$)), 120.1 (Cp-*C*H), 34.6 (C(*C*H$_3$)$_3$), 33.1 (C(*C*H$_3$)$_3$), 32.7 (C(*C*H$_3$)$_3$), 31.1 (C(*C*H$_3$)$_3$), 11.9 (Cp-C(*C*H$_3$)). $^{19}$F{$^1$H} NMR (376.46 MHz, $C_6H_5F$ with a $D_2O$ insert, 298 K): δ = –75.5 ([Al{OC(CF$_3$)$_3$}$_4$]$^-$), –113.9 ($C_6H_5F$). FTIR (ATR, microcrystalline): ṽ = 2967 (w, C-H stretch), 2927 (w, C-H stretch), 2869 (w, C-H stretch), 1467 (w), 1354 (s, C-O stretch), 1299 (s), 1274 (s, C-F stretch), 1239 (s), 1208 (s), 1165 (s), 1060 (w), 1021 (w), 970 (s), 832 (s, Al-O stretch), 756 (w), 725 (s), 682 (w), 561 (s), 536 (s), 444 (s) cm$^{-1}$.

**[BH(Cp$^{ttt}$)][BH(Cp*)][B($C_6F_5$)$_4$]$_2$ (5).** Separate small scale reactions of **2-Dy** (0.055 g, 0.10 mmol) with either [($SiEt_3$)$_2$($\mu$-H)][B($C_6F_5$)$_4$] (0.091 g, 0.10 mmol) or [$CPh_3$][B($C_6F_5$)$_4$] (0.092 g, 0.10 mmol) in benzene (0.25 mL), which were subsequently layered with *n*-hexane (2 mL), lead to the formation of several crystals of **5**. These were identified by single crystal X-ray diffraction. An improved dataset (**5·$C_6H_5F$**) was obtained when recrystallization was performed from a solution of fluorobenzene (0.25 mL) layered with *n*-hexane (2 mL). No further characterization data for **5** were obtained.

## 4. Infrared Spectroscopy

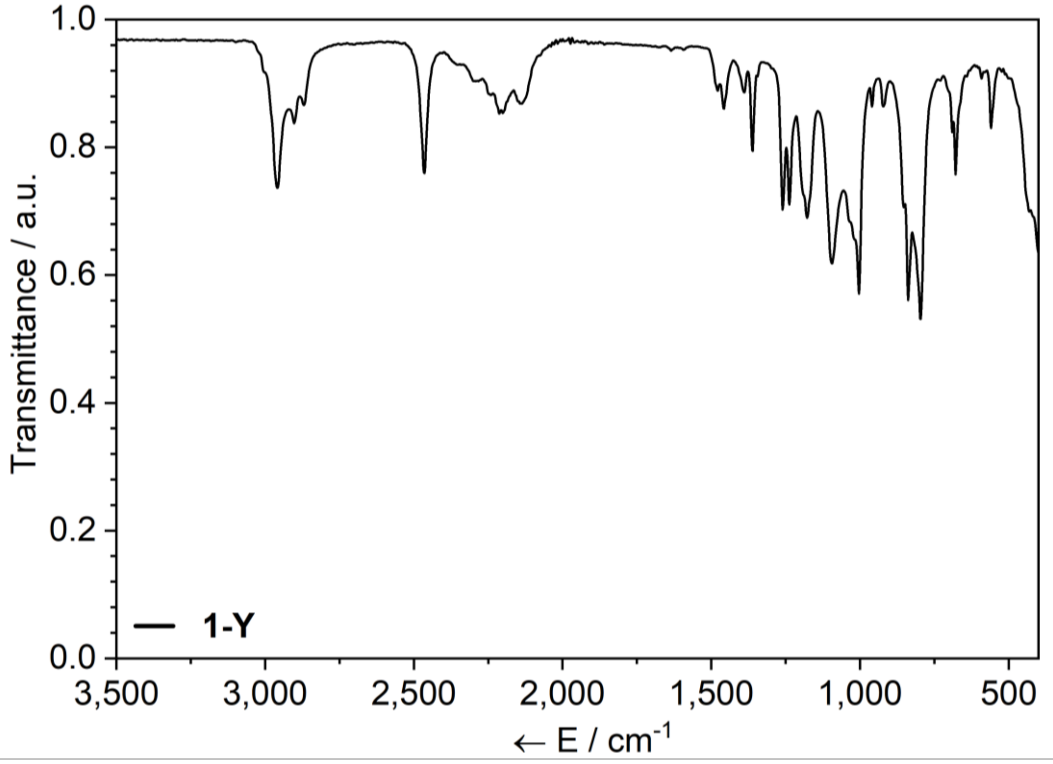


**Figure S11.** ATR-IR spectrum of **1-Y**, recorded as a microcrystalline powder.

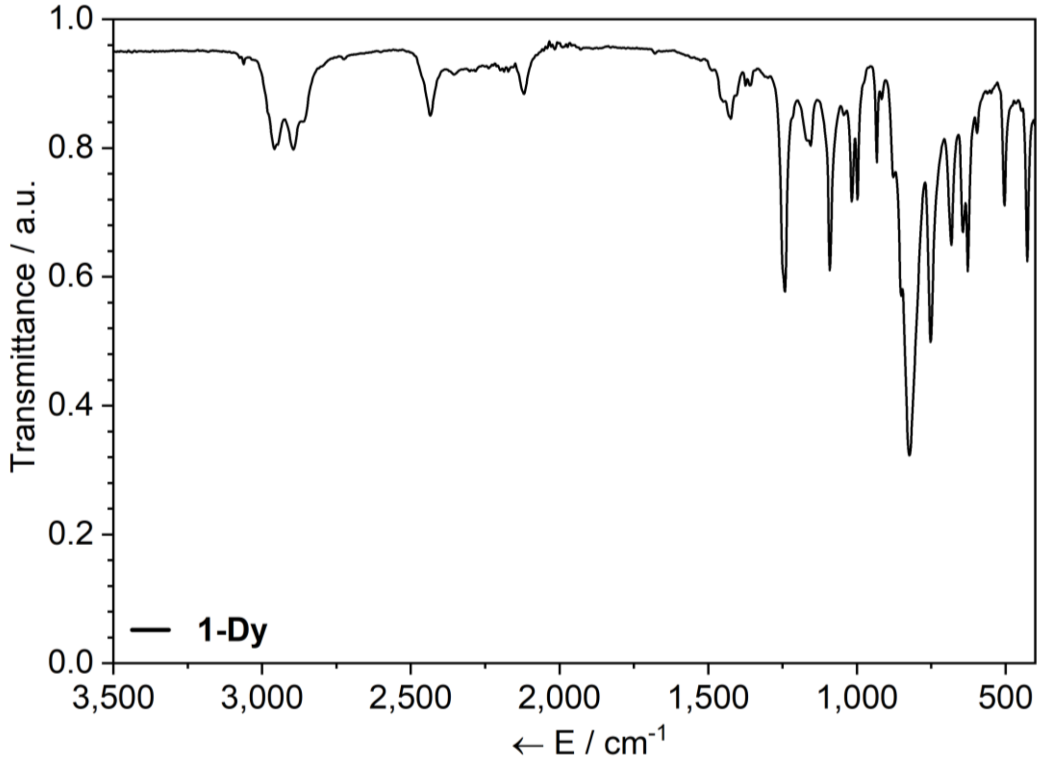


**Figure S12.** ATR-IR spectrum of **1-Dy**, recorded as a microcrystalline powder.

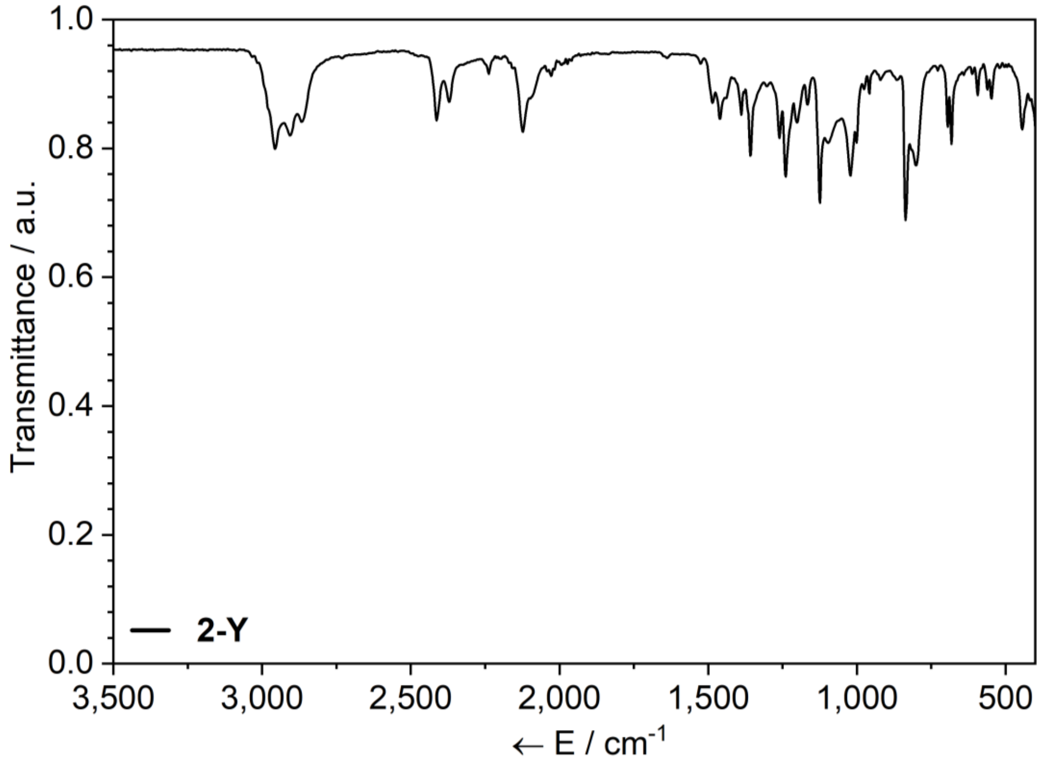


**Figure S13.** ATR-IR spectrum of **2-Y**, recorded as a microcrystalline powder.

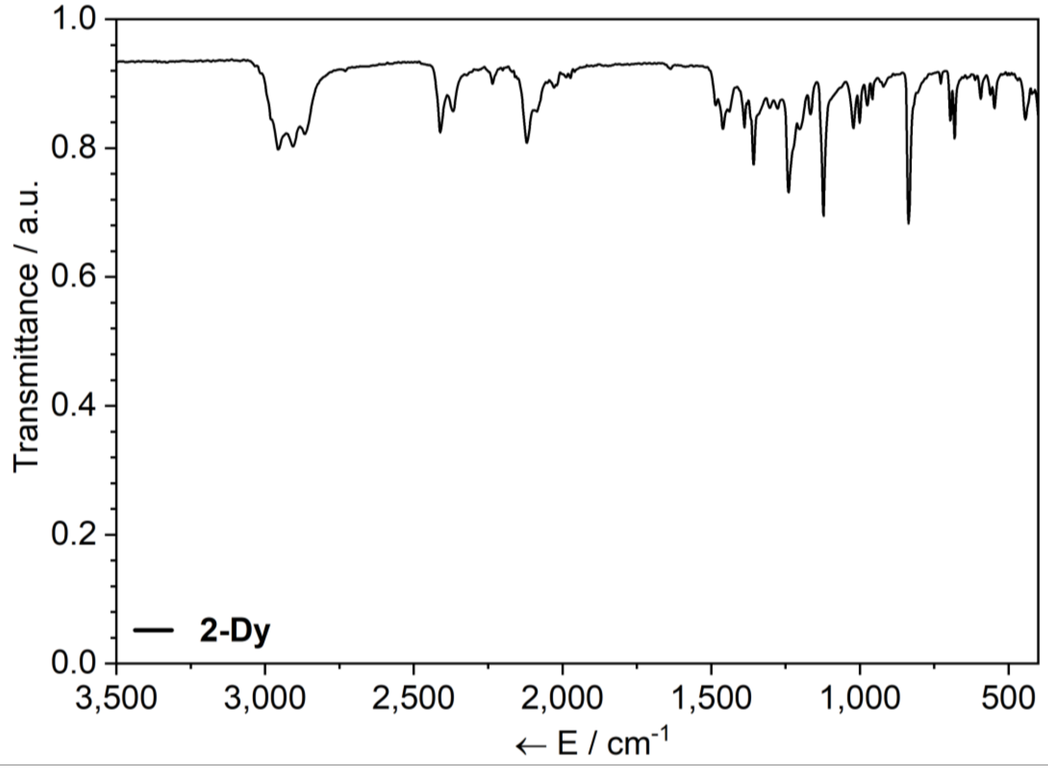


**Figure S14.** ATR-IR spectrum of **2-Dy**, recorded as a microcrystalline powder.

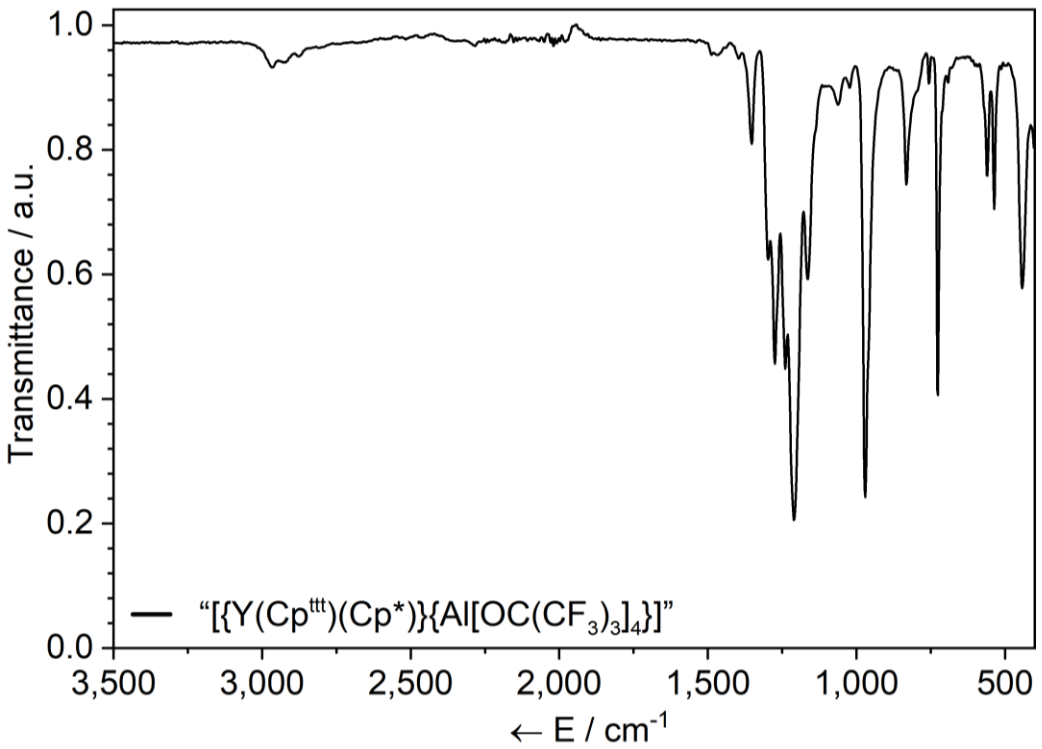


**Figure S15.** ATR-IR spectrum of "[{Y(Cp$^{ttt}$)(Cp*)}{Al[OC($CF_3$)$_3$]$_4$}]", recorded as a powder.

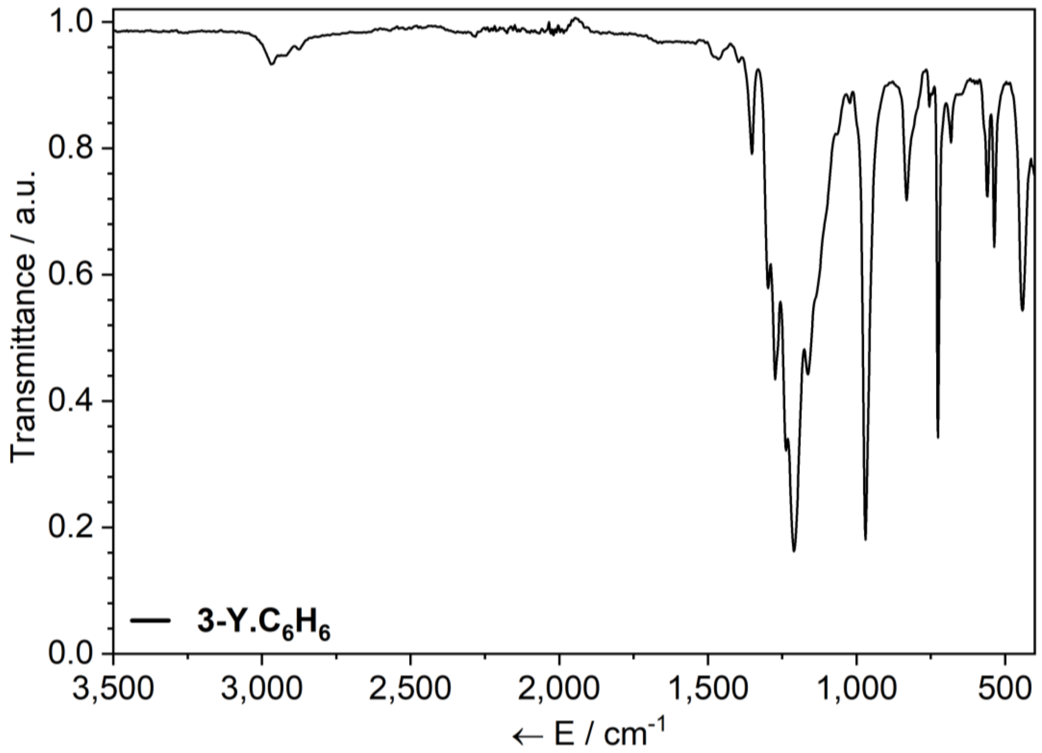


**Figure S16.** ATR-IR spectrum of **3-Y·$C_6H_6$**, recorded as a microcrystalline powder.

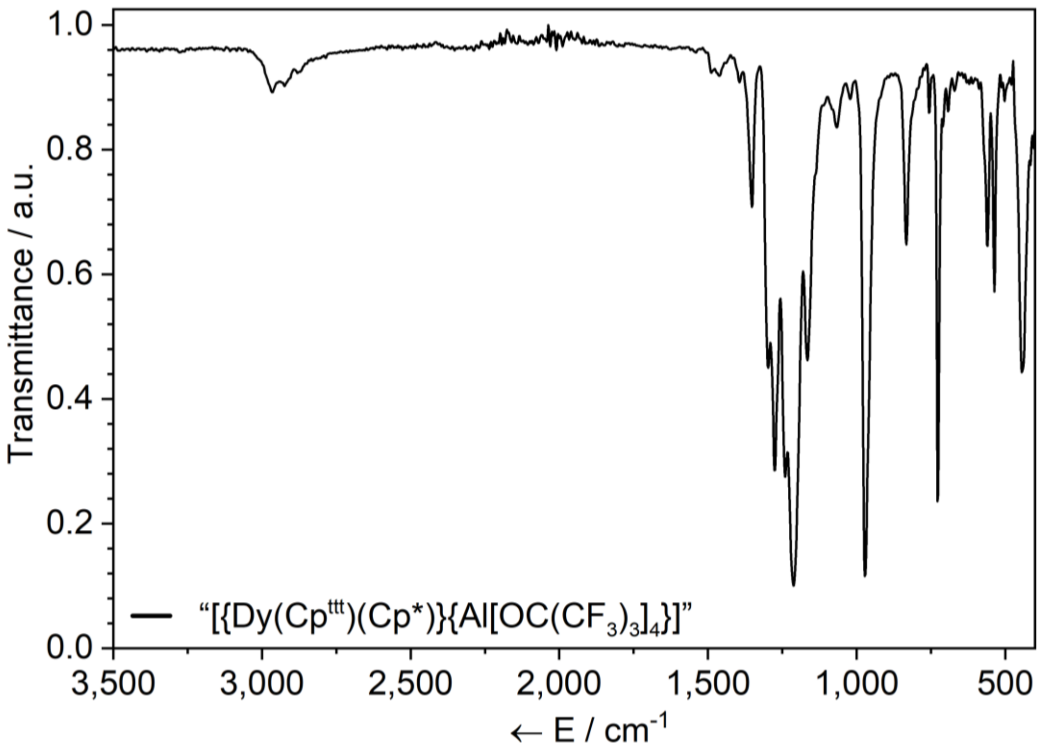


**Figure S17.** ATR-IR spectrum of"[{Dy(Cp$^{ttt}$)(Cp*)}{Al[OC($CF_3$)$_3$]$_4$}]", recorded as a powder.

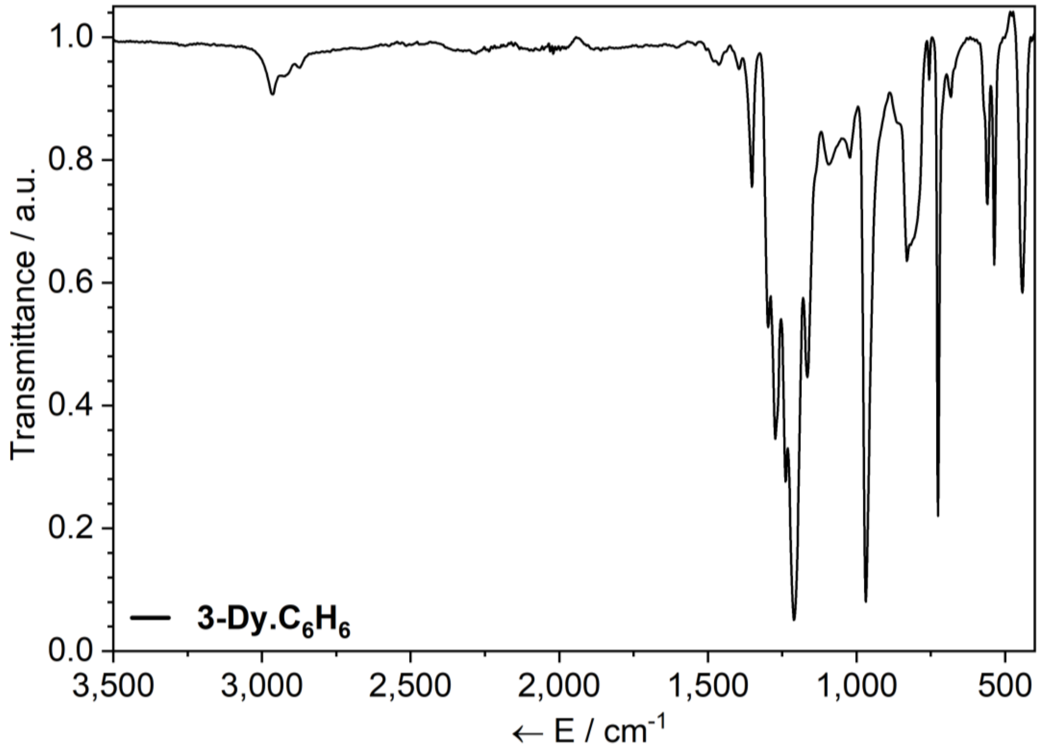


**Figure S18.** ATR-IR spectrum of **3-Dy·$C_6H_6$**, recorded as a microcrystalline powder.

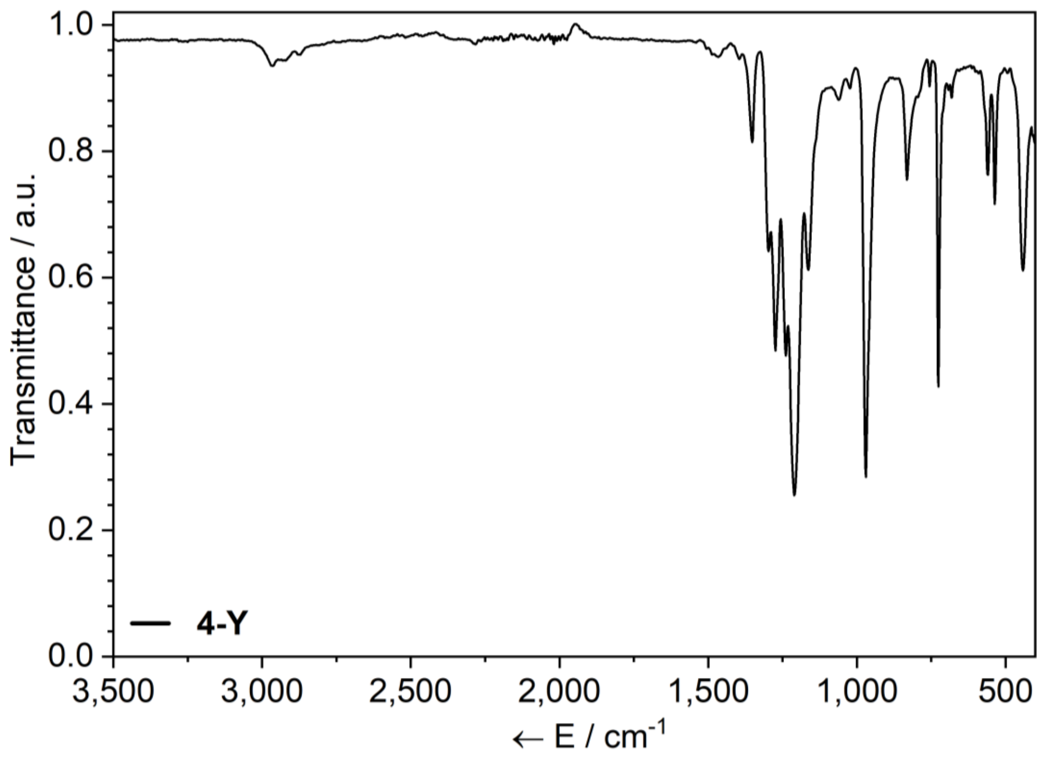


**Figure S19.** ATR-IR spectrum of **4-Y**, recorded as a microcrystalline powder.

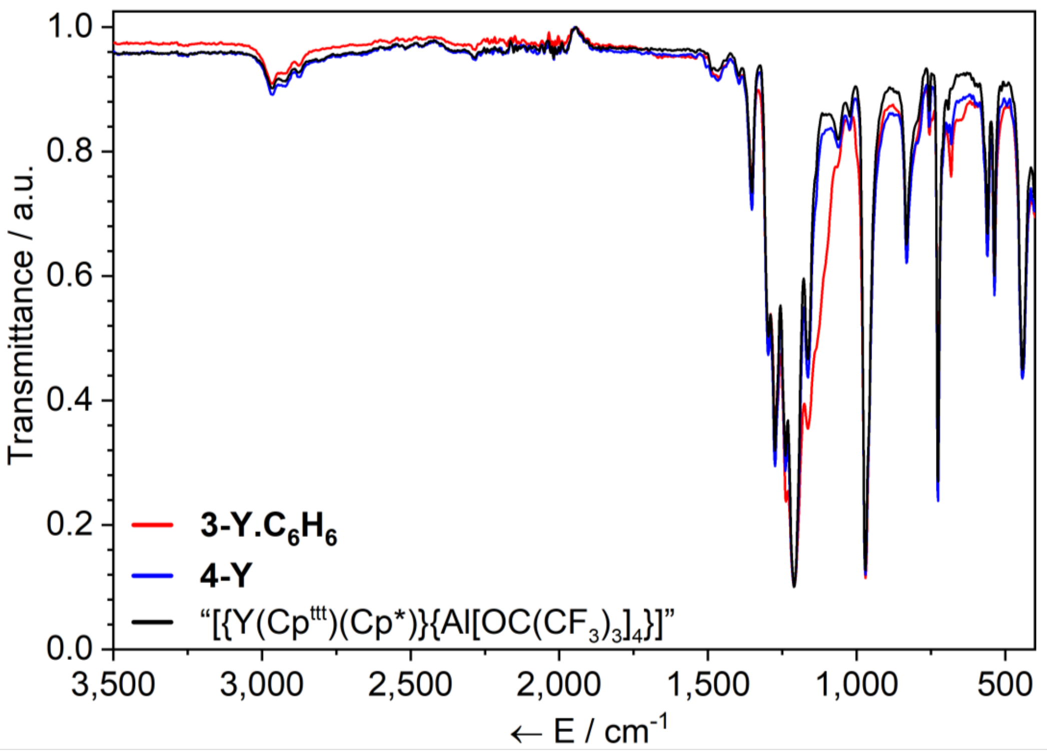


**Figure S20.** Overlaid ATR-IR spectra recorded for "[{Y(Cp$^{ttt}$)(Cp*)}{Al[OC($CF_3$)$_3$]$_4$}]", **3-Y·$C_6H_6$** and **4-Y**. The data are normalized.

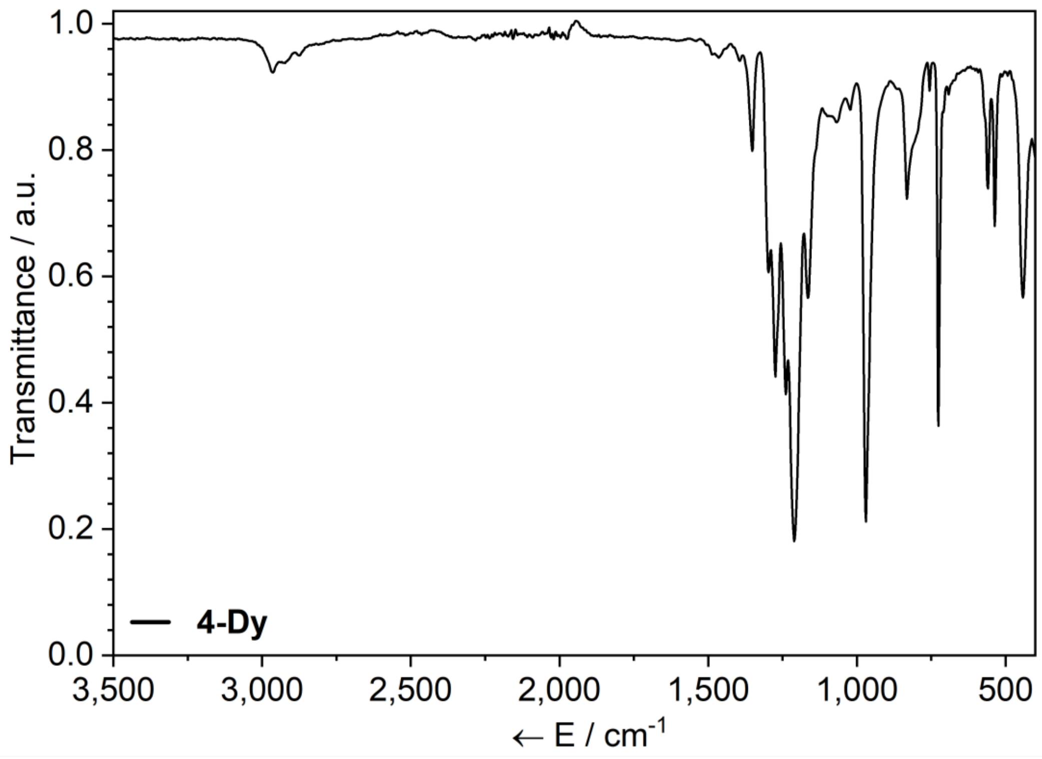


**Figure S21.** ATR-IR spectrum of **4-Dy**, recorded as a microcrystalline powder.

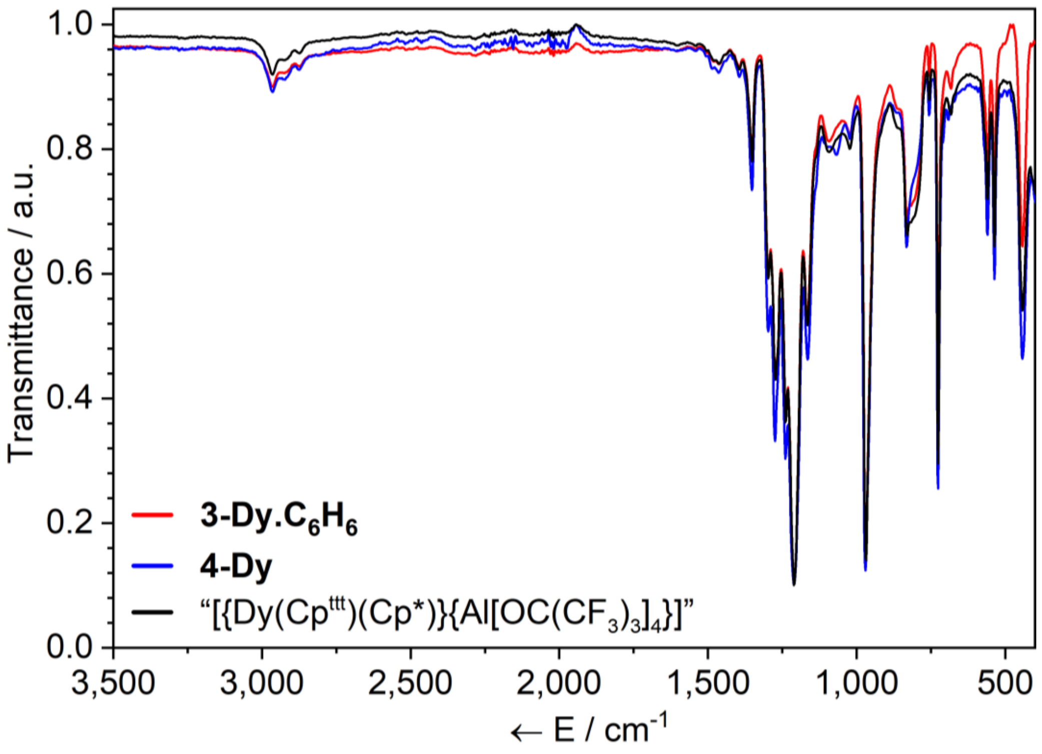


**Figure S22.** Overlaid ATR-IR spectra recorded for "[{Dy($Cp^{ttt}$)(Cp*)}{Al[OC($CF_3$)$_3$]$_4$}]", **3-Dy** and **4-Dy**. The data are normalized.

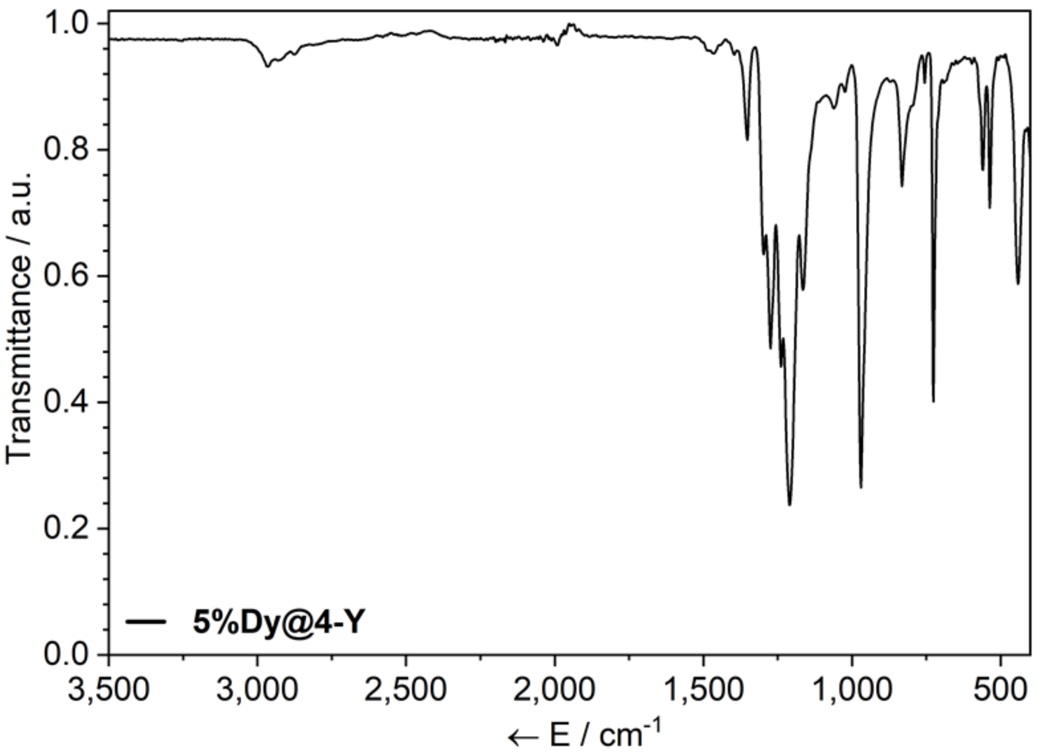


**Figure S23.** ATR-IR spectrum of **5%Dy@4-Y**, recorded as a microcrystalline powder.

## 5. Powder X-ray Diffraction

**Data collection.** X-ray diffraction data of microcrystalline samples of **3-Dy·$C_6H_6$**, **4-Dy** and **5%Dy@4-Y** mounted with a minimum amount of Fomblin® were collected at 100 K using a Rigaku FR-X rotating anode single crystal X-ray diffractometer using Cu Kα radiation (λ = 1.5418 Å) with a Hypix-6000HE detector and an Oxford Cryosystems nitrogen flow gas system. Data were collected between 3–70 °θ, with a detector distance of 150 mm and a beam divergence of 1.0 or 1.5 mRad.[19] X-ray data were collected using CrysAlisPro software.[20]

**Data processing.** The instrument was calibrated using silver behenate as standard. Then, X-ray data were reduced and integrated using CrysAlisPro software.[20] Pawley refinement with the unit cells obtained from the crystal structures were performed using TOPAS software.[21,22] Le Bail profile analysis was performed using JANA2006 software.[23]

**Data analysis.** The data are compiled in Figures S24–S30 and Tables S6 and S7. For **3-Dy·$C_6H_6$** there are some unfitted peaks in the diffractogram that match with the peak positions of **4-Dy**, e.g. 2θ = 7.78°. The number of unfitted peaks is small and their intensity is weak, so this minor component cannot be reliably identified or quantified. For **4-Dy** there are some unfitted peaks in the diffractogram that match with the peak positions calculated for **6-Dy·$C_6H_{14}$** (2θ = 5.6, 6.8, 9.2, 9.4 and 11°); again the small number, low intensity and relative broadness of unfitted peaks (due to lower crystallinity of contaminant phase) preclude reliable assignment and quantification. There are also more factors (preferential orientation of crystallites, etc) that affect the individual intensities of the diffracted peaks, which reduces the agreement of experimental and theoretical patterns and prevents reliable quantification of any contaminant phases. Qualitatively, the Pawley refinements match well with the experimental data. The diffractogram of **5%Dy@4-Y** indicates the sample is a combination of two polymorphs, the major polymorph is in the $P2_1/c$ space group shared by the **4-Dy** sample and the minor polymorph is $P\bar{1}$ that matches the SCXRD **5%Dy@4-Y** data. For **5%Dy@4-Y** there

are some unfitted peaks in the diffractogram (2θ = 4.1, 6.3, 8.1°); the small number and low intensity of unfitted peaks preclude reliable assignment and quantification.

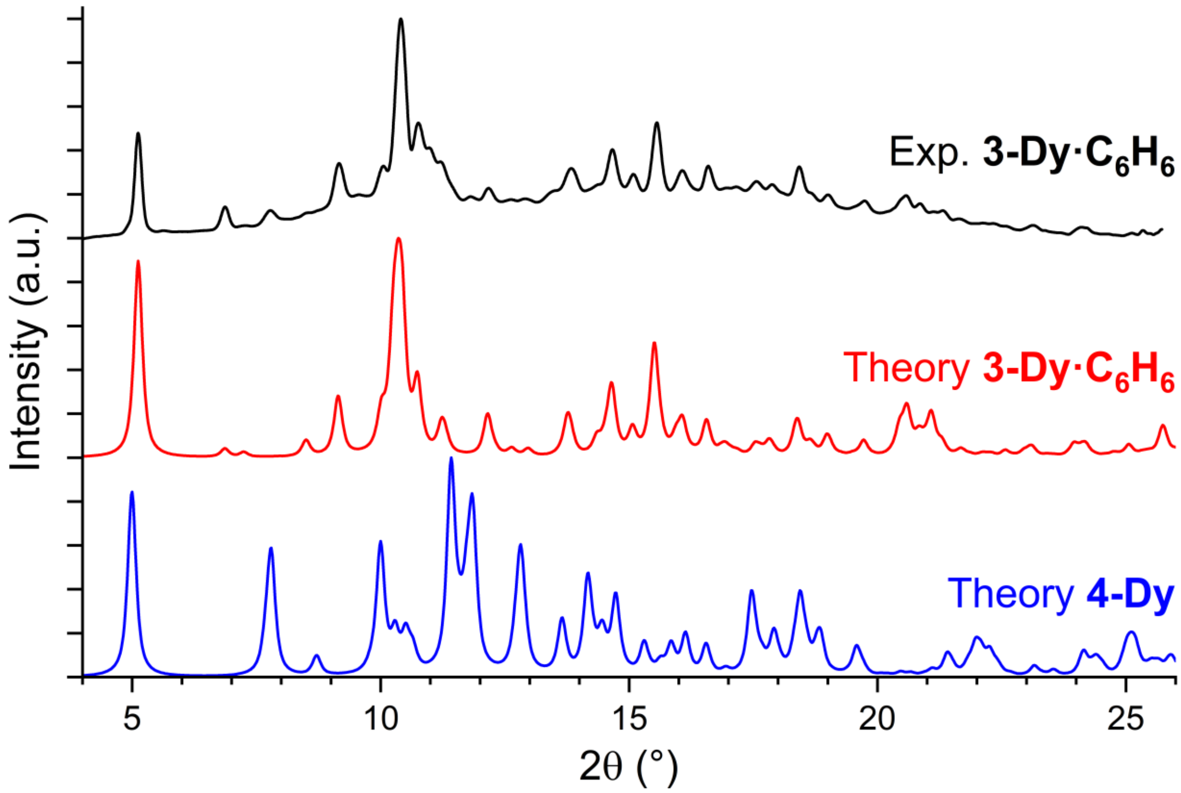


**Figure S24.** Comparison of powder X-ray diffraction pattern of **3-Dy·$C_6H_6$** with theoretical patterns of **3-Dy·$C_6H_6$** and **4-Dy** derived from crystallographic parameters.

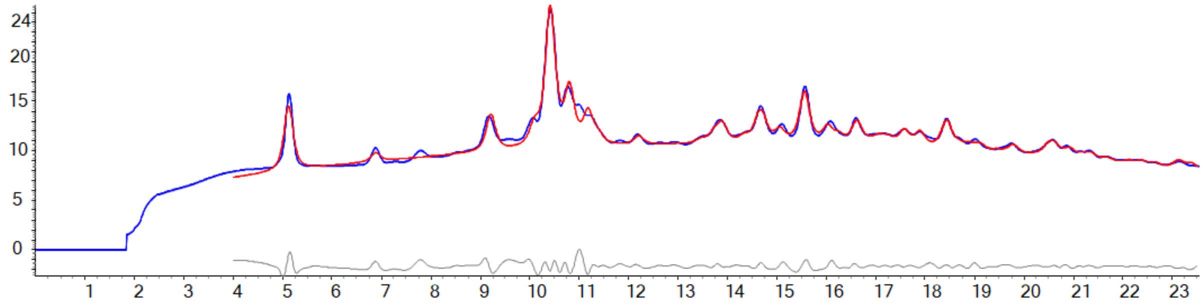


**Figure S25.** Pawley refinement analysis of **3-Dy·$C_6H_6$**; experimental data (blue), calculated model from crystallographic parameters (red) and the difference (grey). Pawley refinement Rwp 2.633; Rwp′ = 14.91. Pawley refinement was determined using the unit cell values from the crystal structure. *x*-axis 2θ (°), *y*-axis intensity (a.u.).

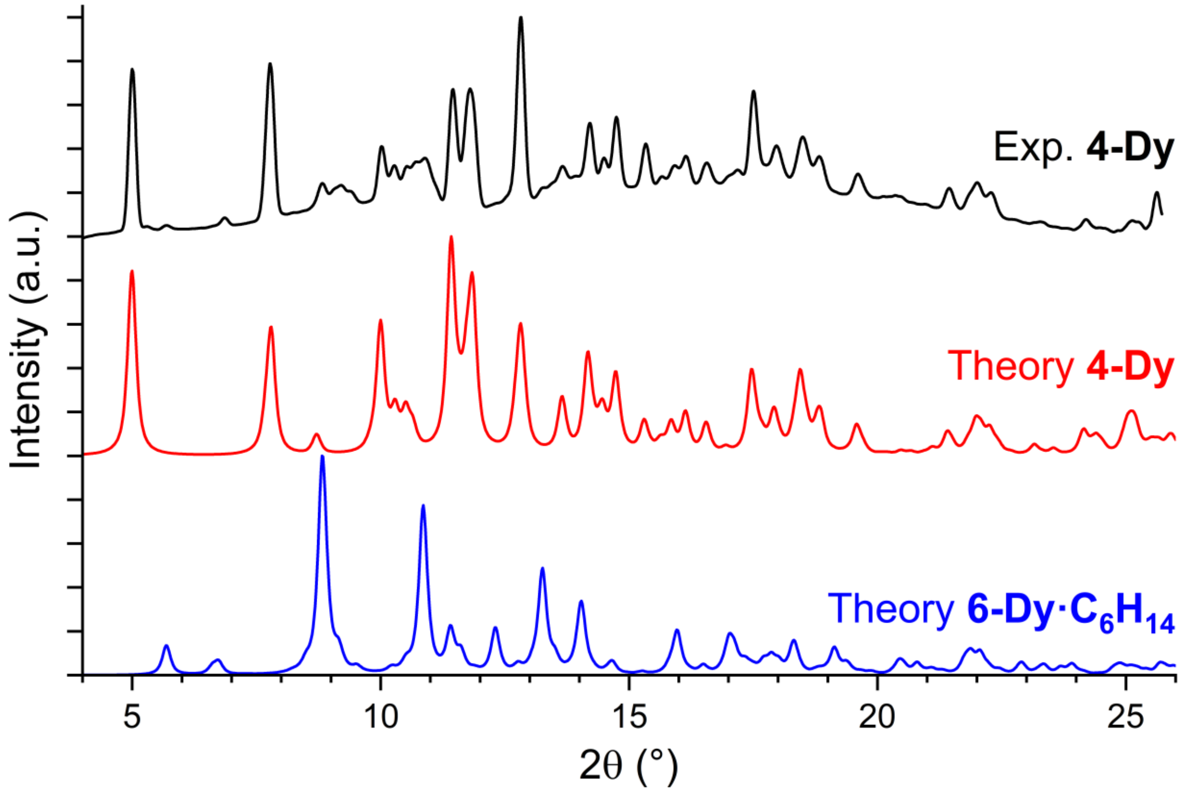


**Figure S26.** Comparison of powder X-ray diffraction pattern of **4-Dy** with theoretical patterns of **4-Dy** and **6-Dy·$C_6H_{14}$** derived from crystallographic parameters.

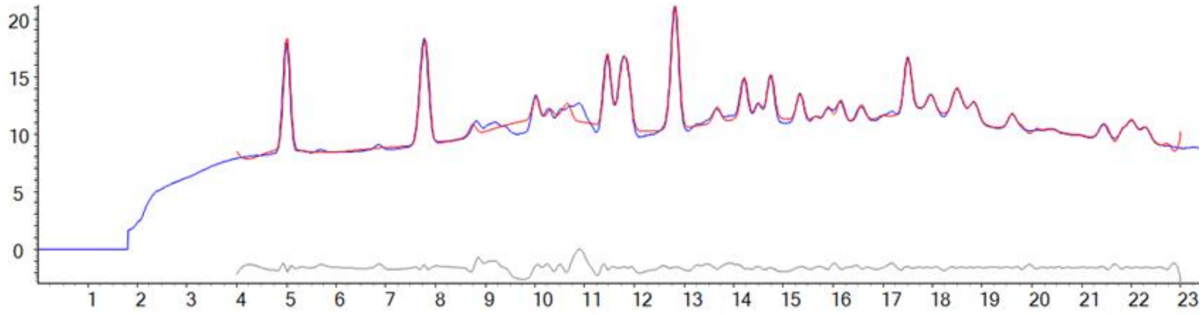


**Figure S27.** Pawley refinement analysis of **4-Dy**; experimental data (blue), calculated model from crystallographic parameters (red) and the difference (grey). Pawley refinement Rwp 2.950; Rwp′ = 12.11. Pawley refinement was determined using the unit cell values from the crystal structure. *x*-axis 2θ (°), *y*-axis intensity (a.u.).

**Table S6.** Unit cell values obtained from Pawley refinement results.

| Complex | a | b | c | α | β | γ |
|---|---|---|---|---|---|---|
| **3-Dy·$C_6H_6$** | 19.31(2) | 17.61(2) | 34.89(4) | 90 | 90 | 90 |
| **4-Dy** | 19.91(1) | 15.07(1) | 20.28(1) | 90 | 117.43(2) | 90 |

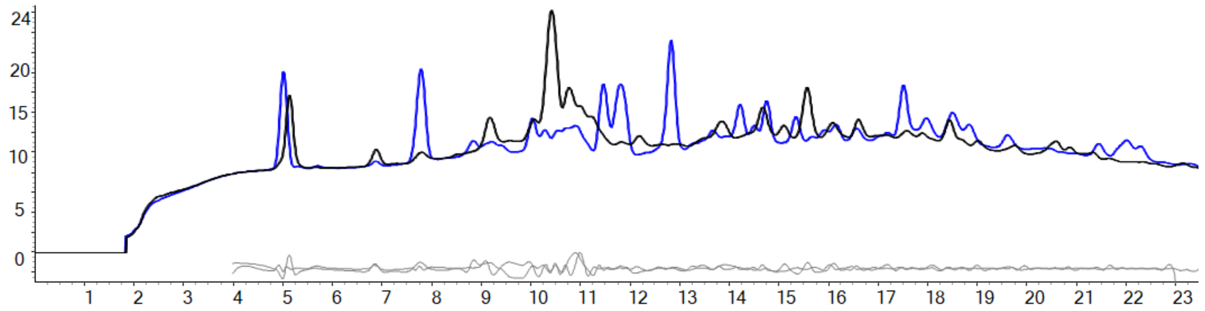


**Figure S28.** Overlaid powder X-ray diffraction patterns of **3-Dy·$C_6H_6$** (black) and **4-Dy** (blue). *x*-axis 2θ (°), *y*-axis intensity (a.u.).

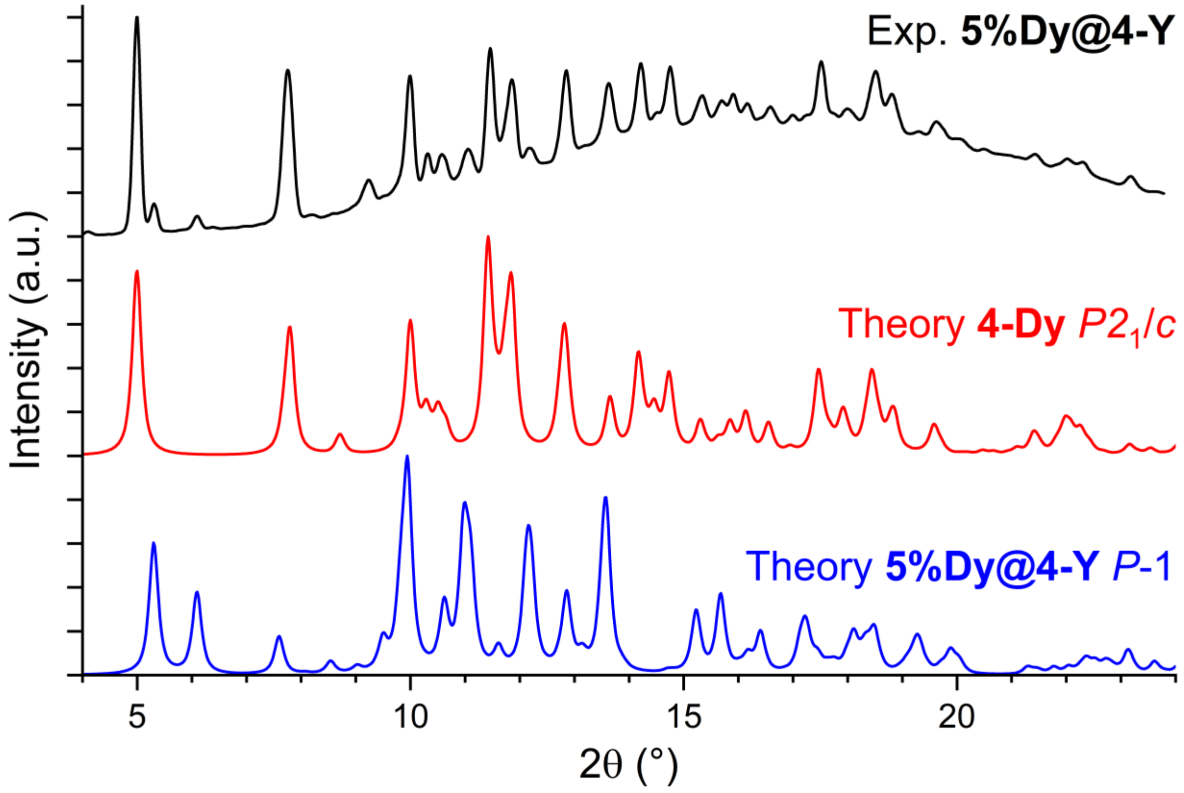


**Figure S29.** Comparison of powder X-ray diffraction pattern of **5%Dy@4-Y** with theoretical patterns of **4-Dy** ($P2_1$/c phase) and **5%Dy@4-Y** ($P\bar{1}$ phase) derived from crystallographic parameters.

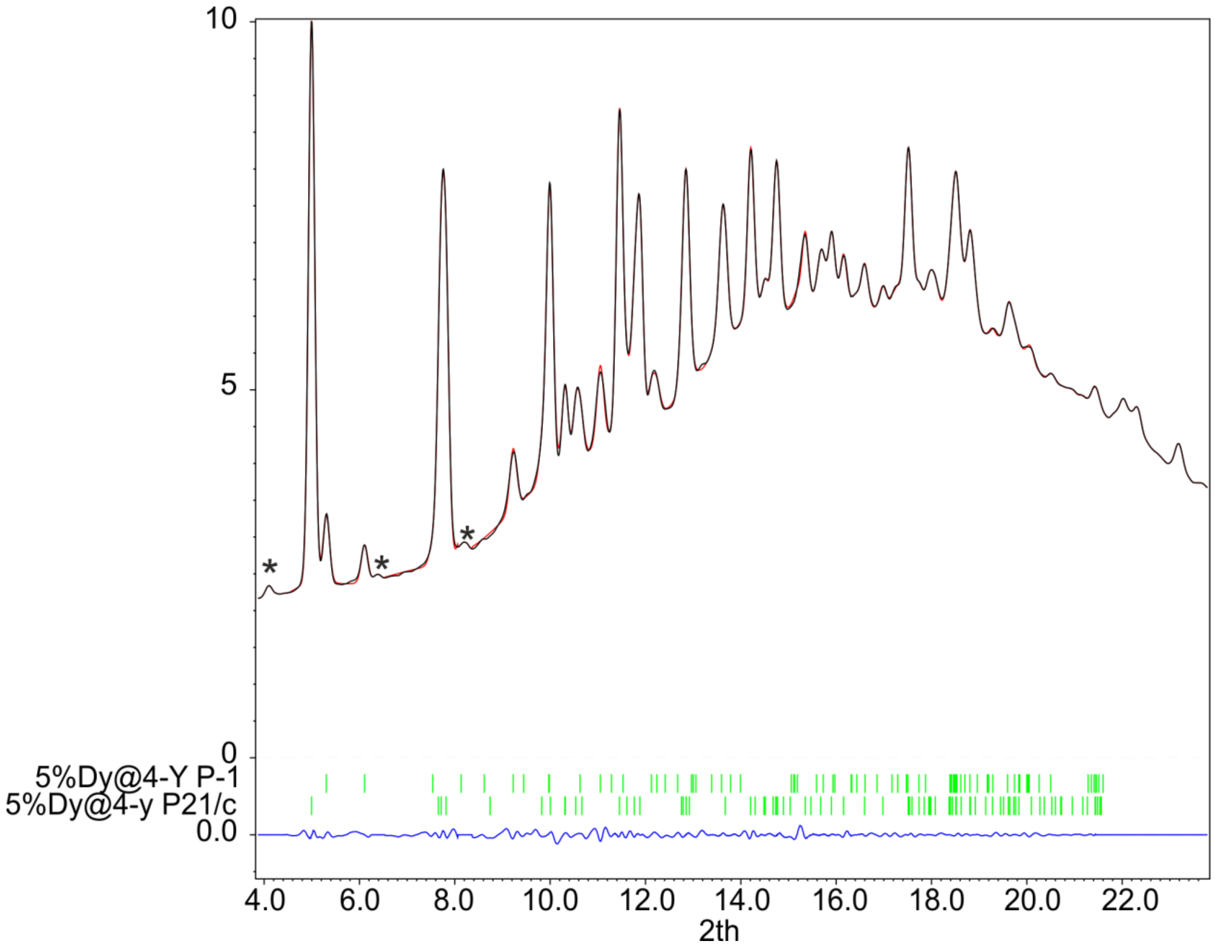


**Figure S30.** Le Bail profile refinement analysis of **5%Dy@4-Y**; experimental data (black), calculated fit from crystallographic parameters (red) and the difference (blue). Pawley refinement Rwp = 0.37; Rwp′ = 0.55. Le Bail profile refinement was determined using the unit cell values from the crystal structure. x-axis 2θ (°), y-axis intensity (a.u.).

**Table S7.** Unit cell values obtained from Le Bail profile refinement results on **5%Dy@4-Y**.

| **Phase** | **a** | **b** | **c** | **α** | **β** | **γ** |
|---|---|---|---|---|---|---|
| $P2_1/c$ | 19.8695(9) | 15.0237(5) | 20.2373(6) | 90 | 117.3053(19) | 90 |
| $P\bar{1}$ | 11.0881(8) | 14.8655(12) | 16.8433(15) | 84.463(10) | 84.946(13) | 78.7022(4) |

## 6. NMR Spectroscopy

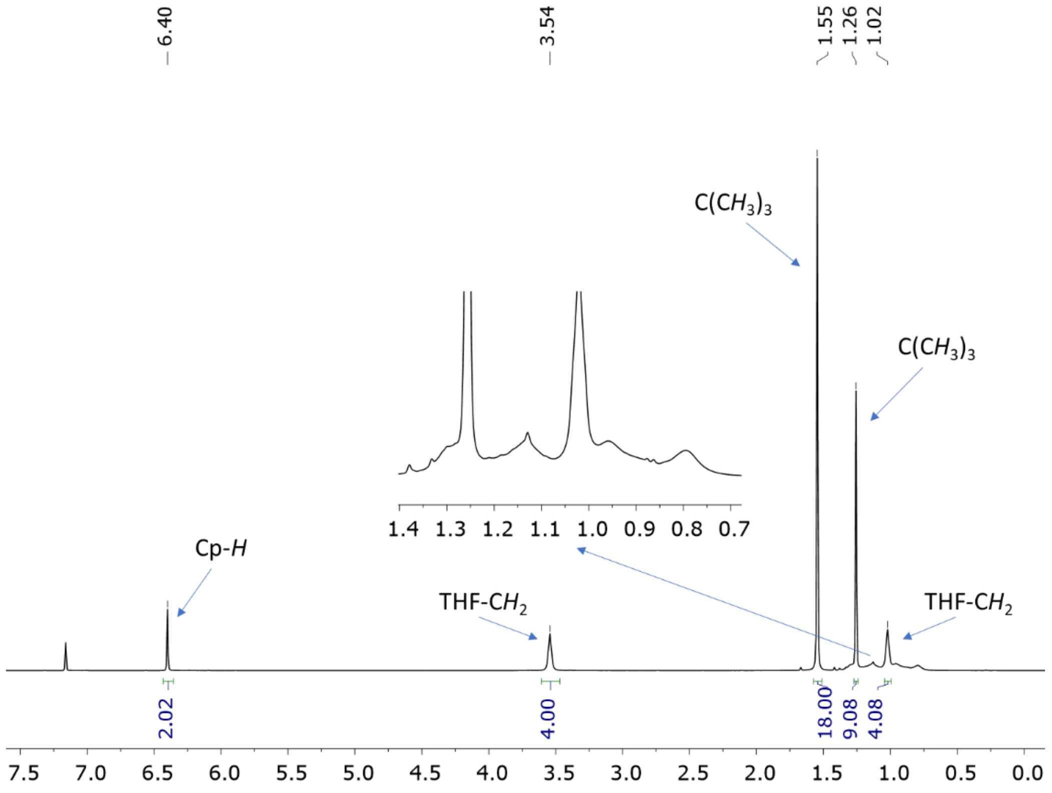


**Figure S31.** $^{1}$H NMR spectrum of **1-Y** (500 MHz) in $C_6D_6$.

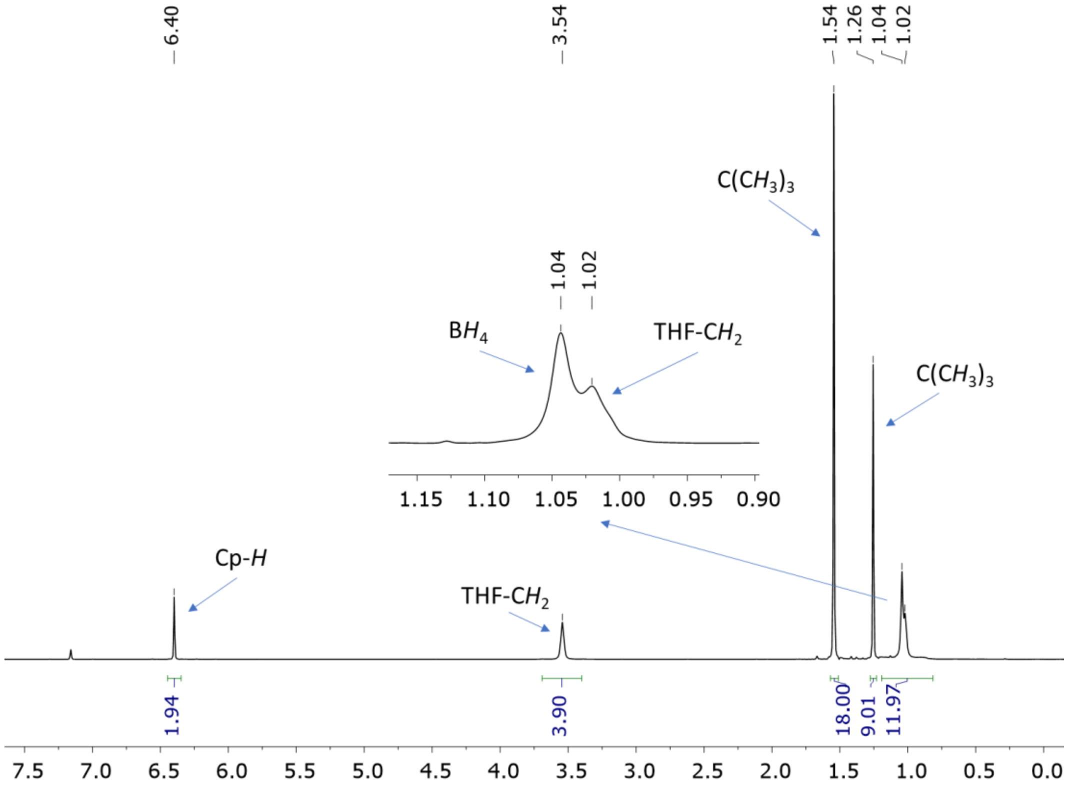


**Figure S32.** $^{1}$H{$^{11}$B} NMR spectrum of **1-Y** (500 MHz) in $C_6D_6$.

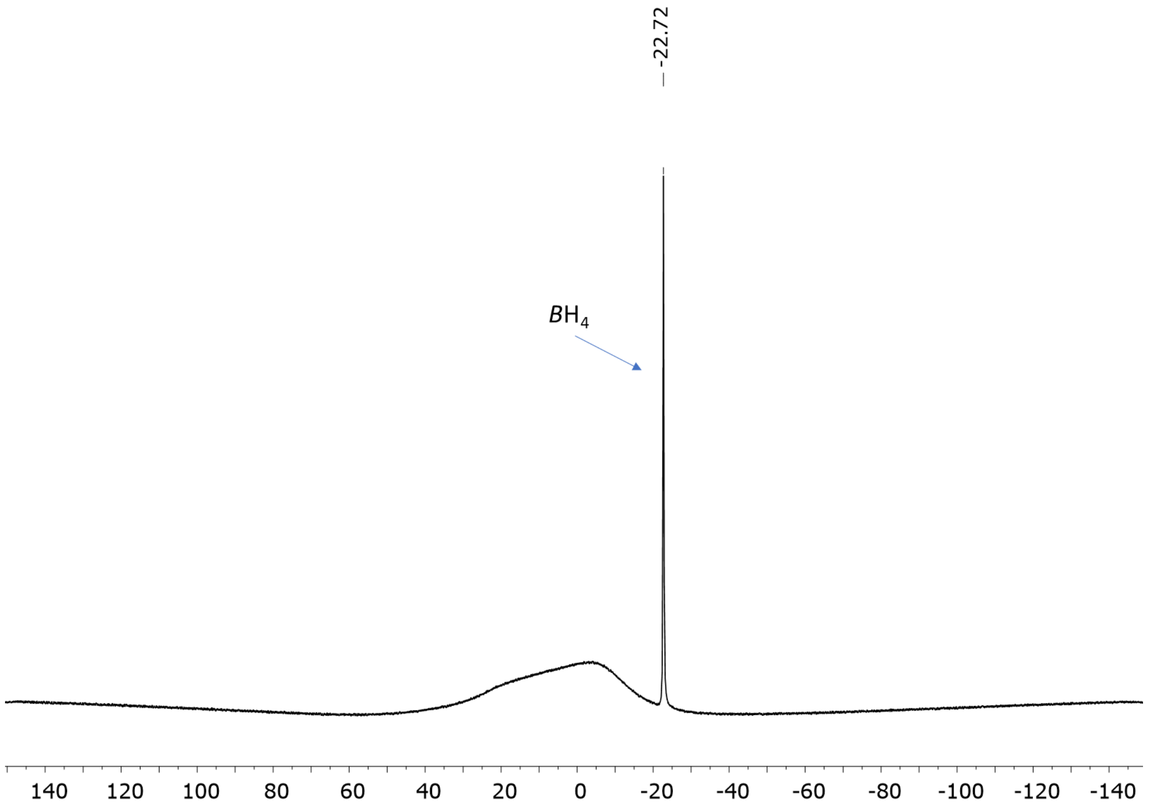


**Figure S33.** $^{11}B\{^{1}H\}$ NMR spectrum of **1-Y** (160 MHz) in $C_6D_6$.

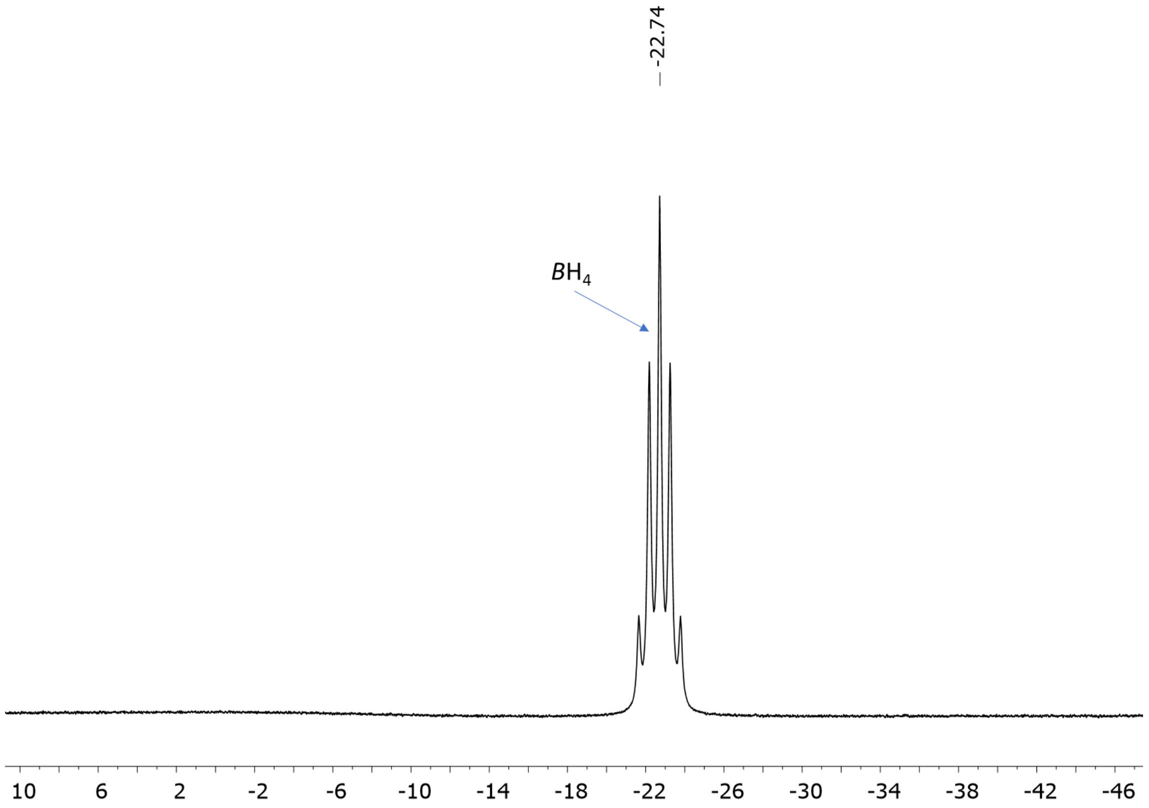


**Figure S34.** $^{11}B$ NMR spectrum of **1-Y** (160 MHz) in $C_6D_6$.

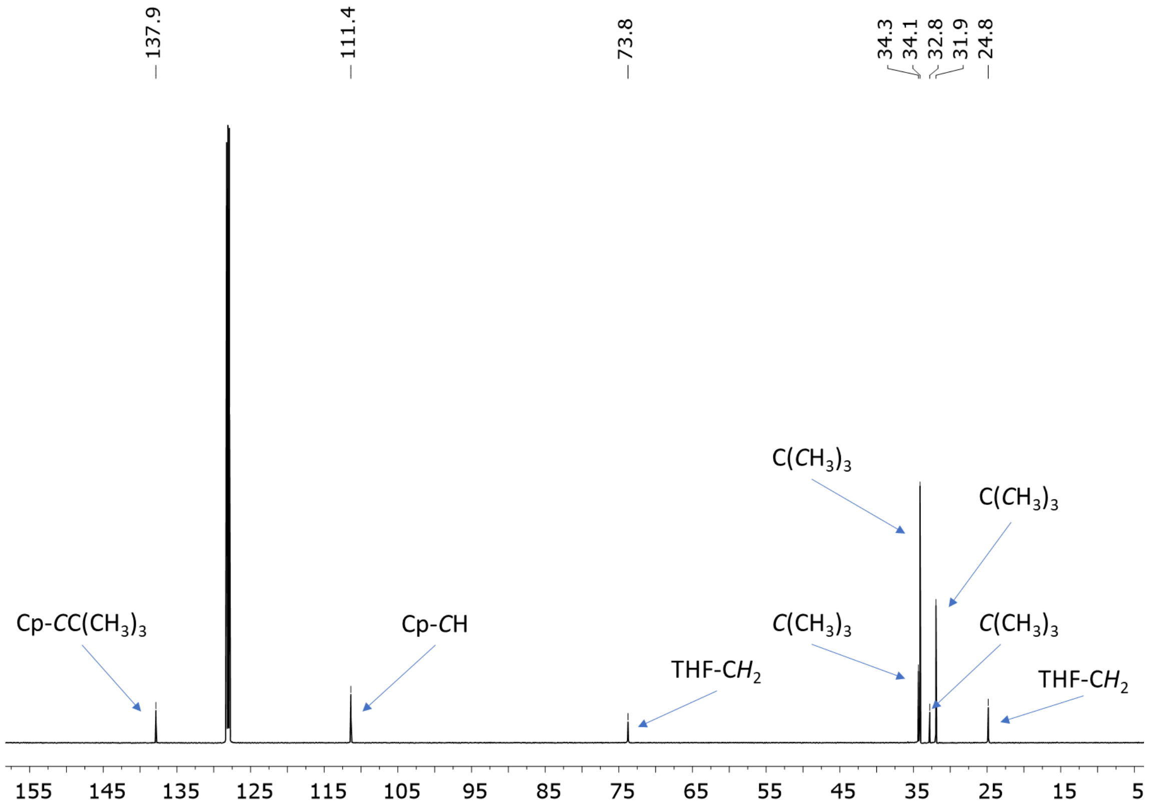


**Figure S35.** $^{13}C\{^1H\}$ NMR spectrum of **1-Y** (126 MHz) in $C_6D_6$.

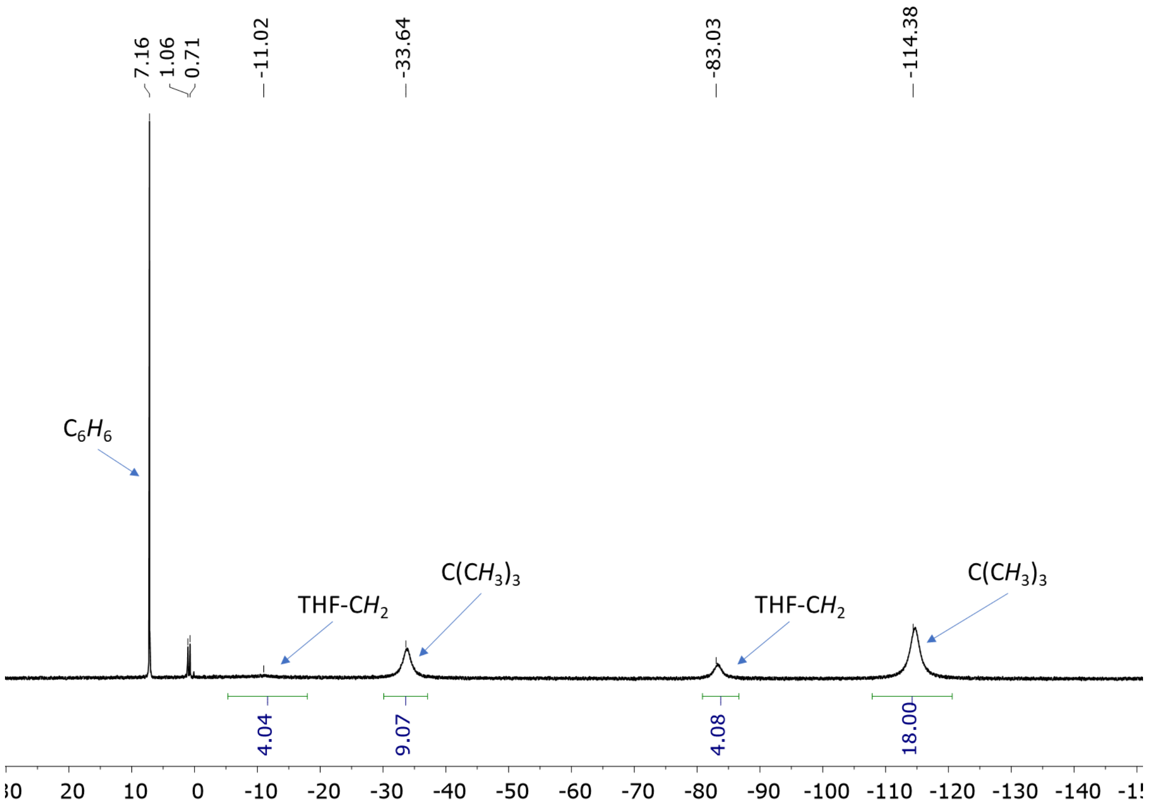


**Figure S36.** $^1H$ NMR spectrum of **1-Dy** (400 MHz) in $C_6D_6$; full spectral range 100 to –300 ppm.

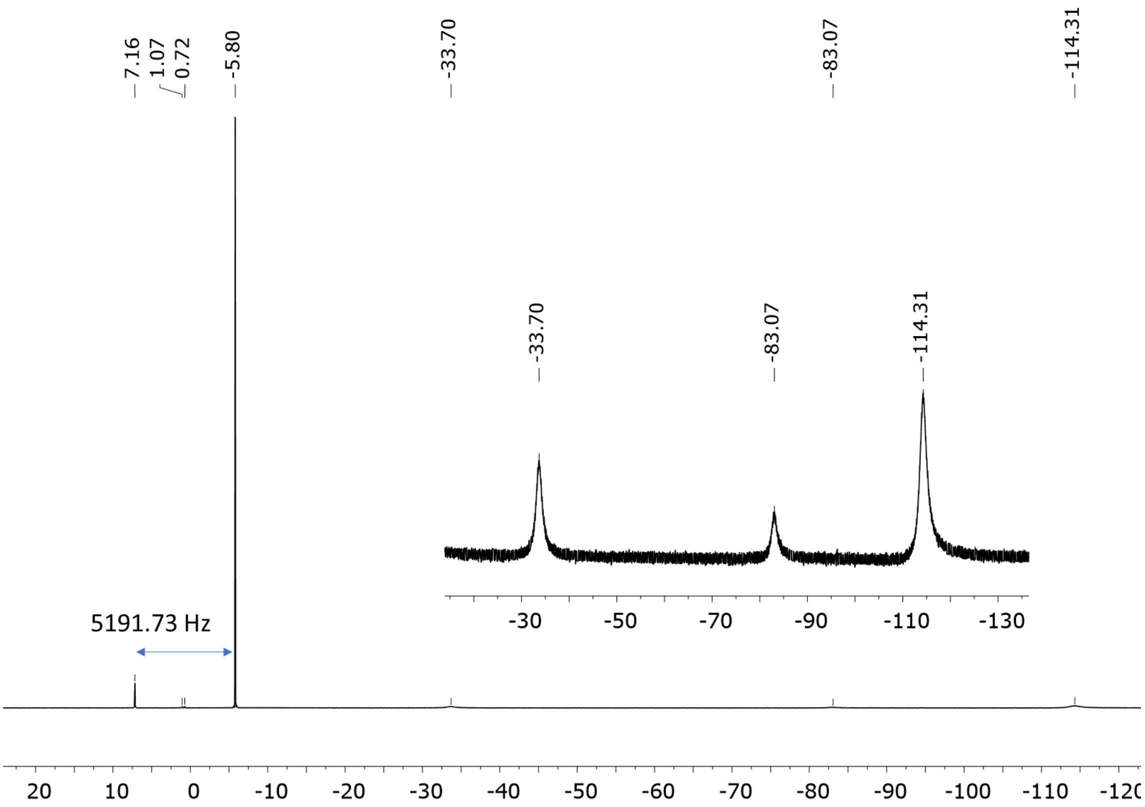


**Figure S37.** $^{1}$H NMR spectrum of **1-Dy** (400 MHz) in $C_6D_6$ with a $C_6D_6$ insert; full spectral range 100 to –300 ppm.

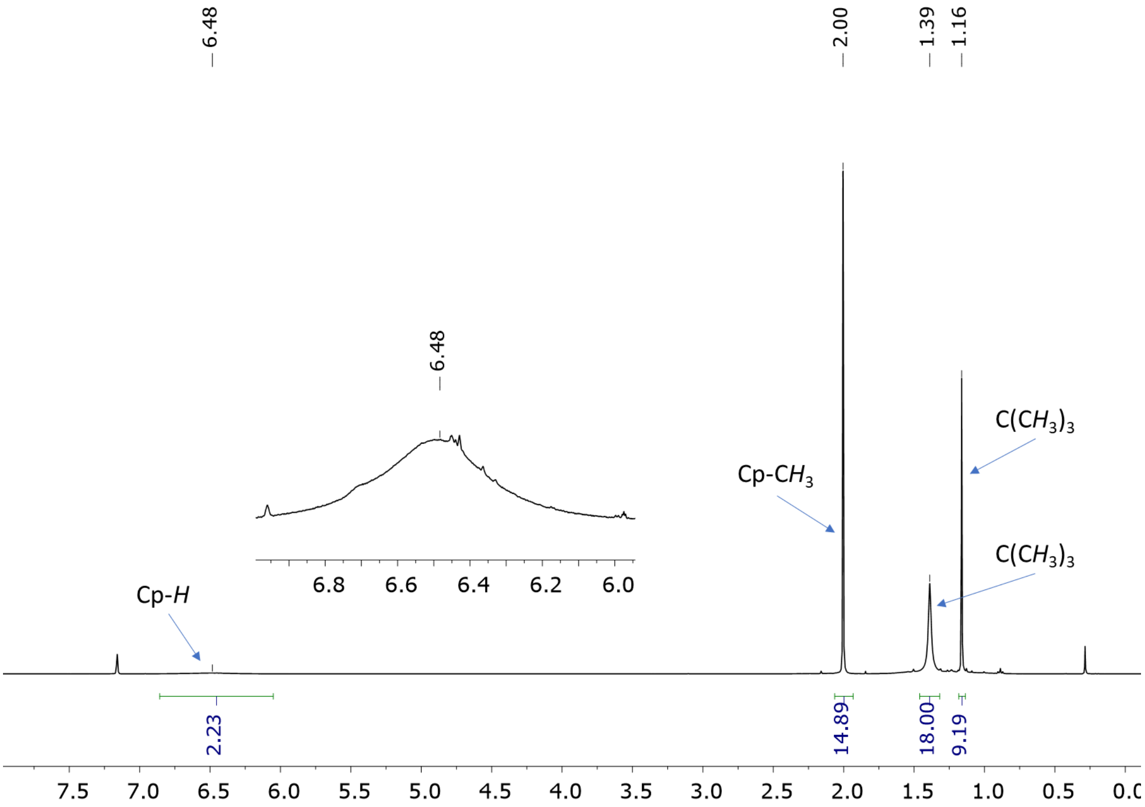


**Figure S38.** $^{1}$H NMR spectrum of **2-Y** (400 MHz) in $C_6D_6$.

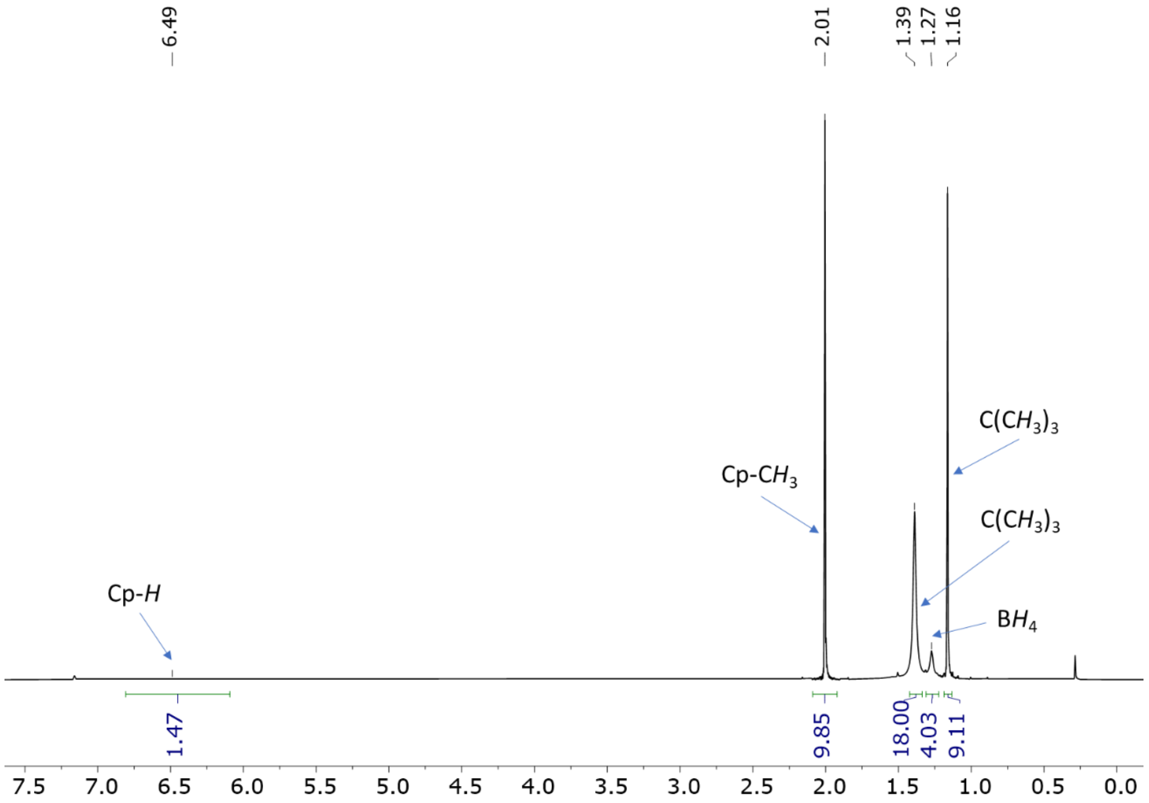


**Figure S39.** $^{1}H\{^{11}B\}$ NMR spectrum of **2-Y** (400 MHz) in $C_6D_6$.

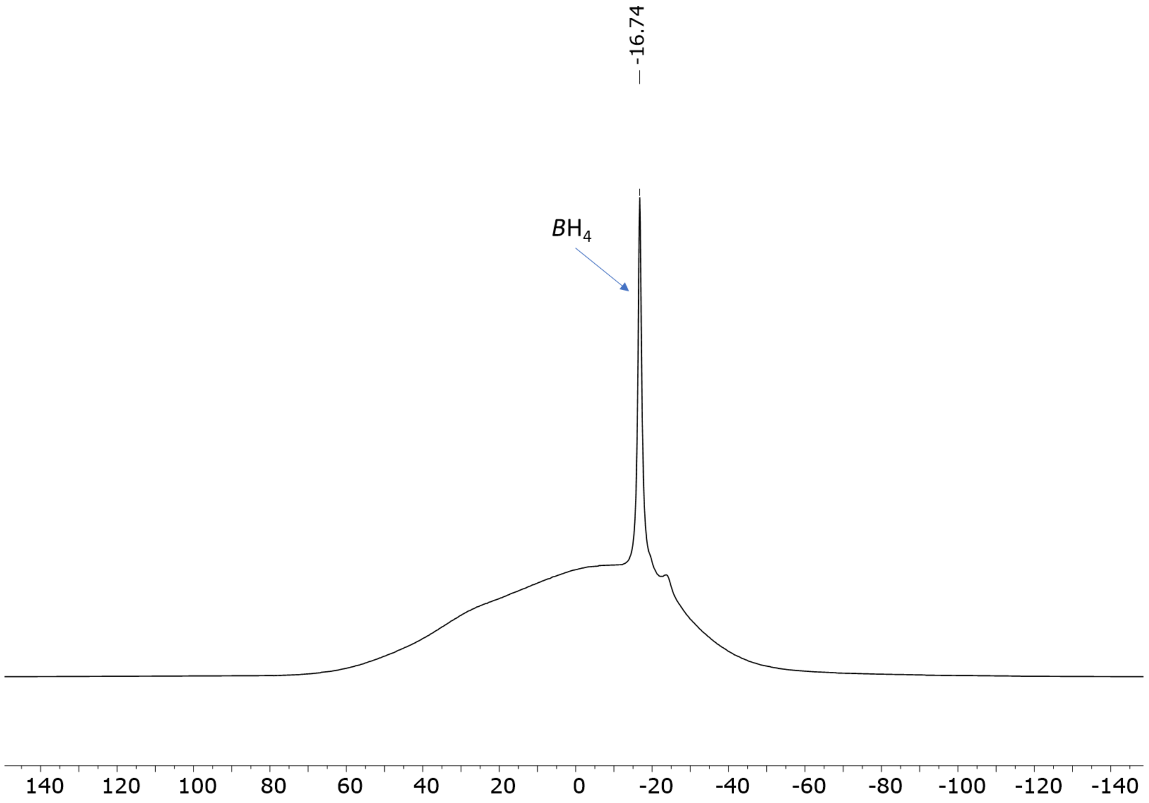


**Figure S40.** $^{11}B\{^{1}H\}$ NMR spectrum of **2-Y** (128 MHz) in $C_6D_6$.

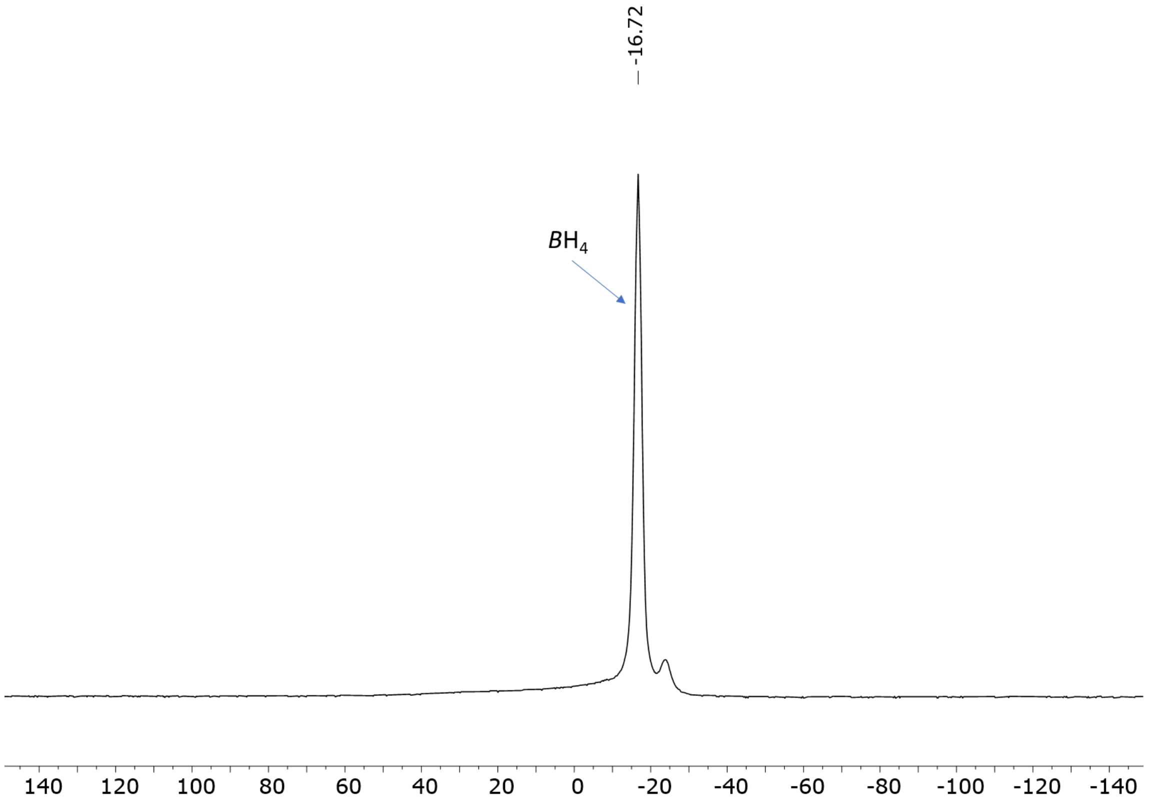


**Figure S41.** $^{11}B$ NMR spectrum of **2-Y** (128 MHz) in $C_6D_6$.

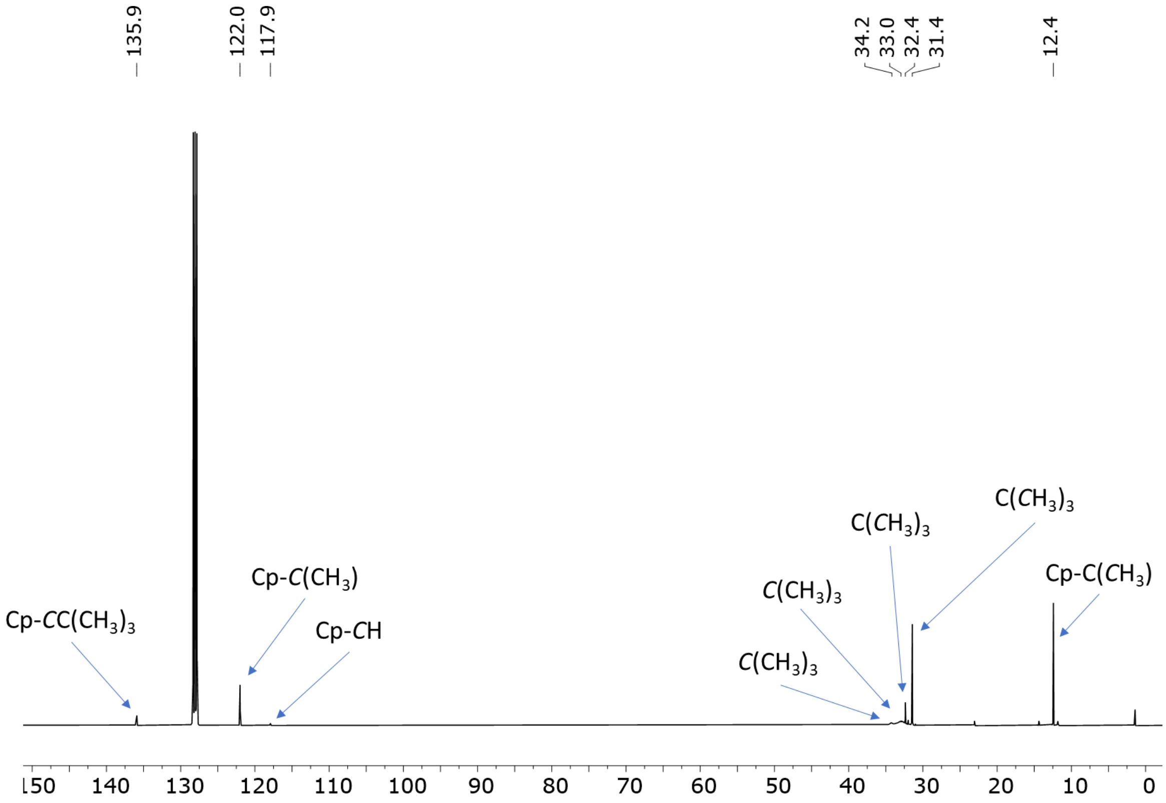


**Figure S42.** $^{13}C\{^1H\}$ NMR spectrum of **2-Y** (100 MHz) in $C_6D_6$.

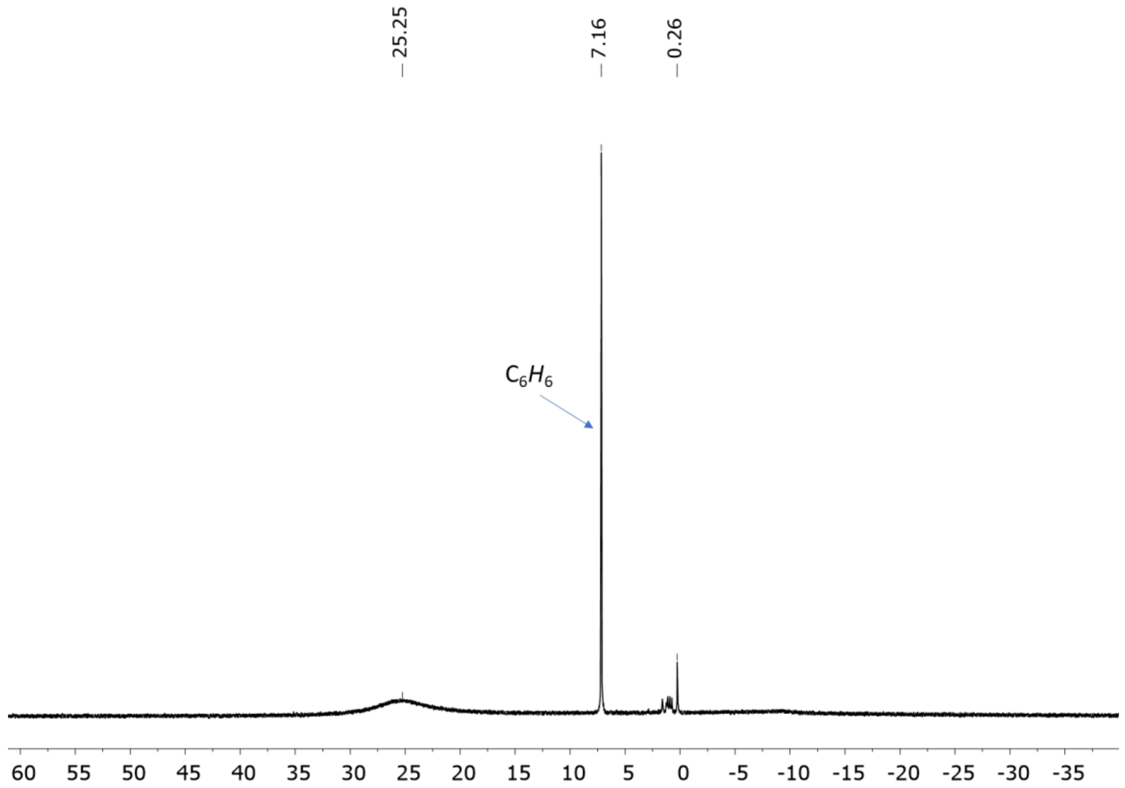


**Figure S43.** $^{1}$H NMR spectrum of **2-Dy** (400 MHz) in $C_6D_6$; full spectral range 100 to –300 ppm.

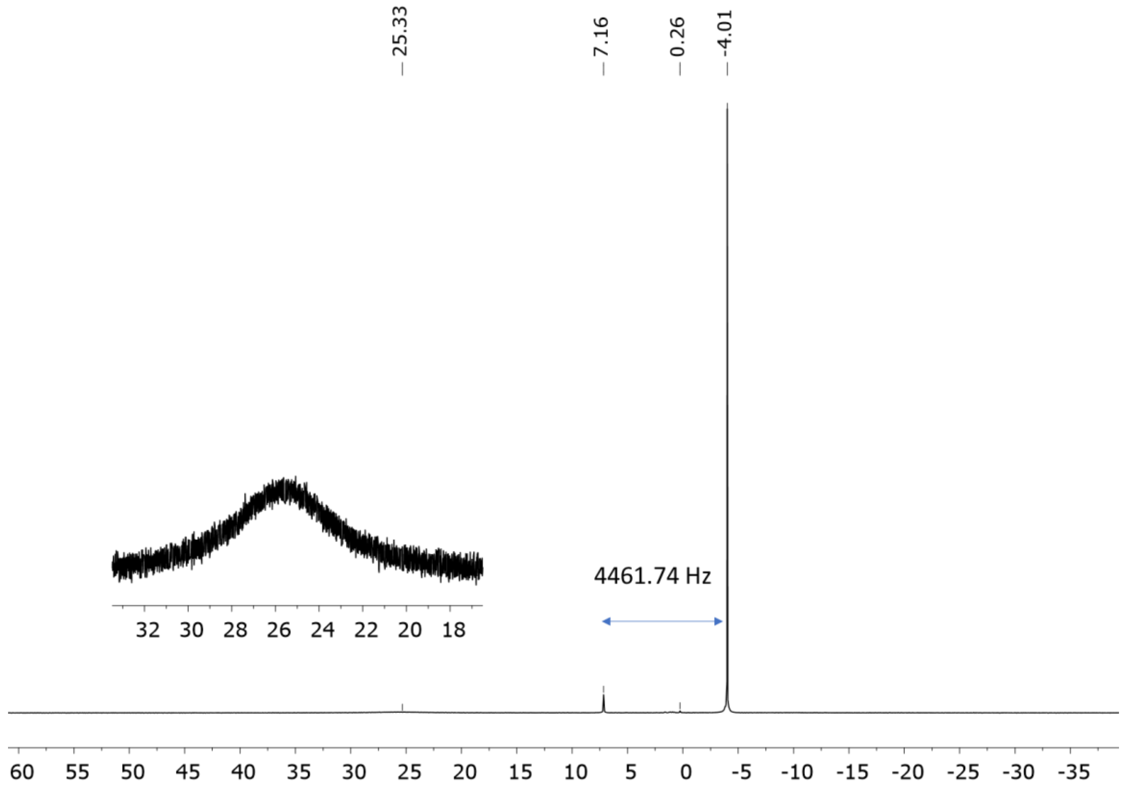


**Figure S44.** $^{1}$H NMR spectrum of **2-Dy** (400 MHz) in $C_6D_6$ with a $C_6D_6$ insert; full spectral range 100 to –300 ppm.

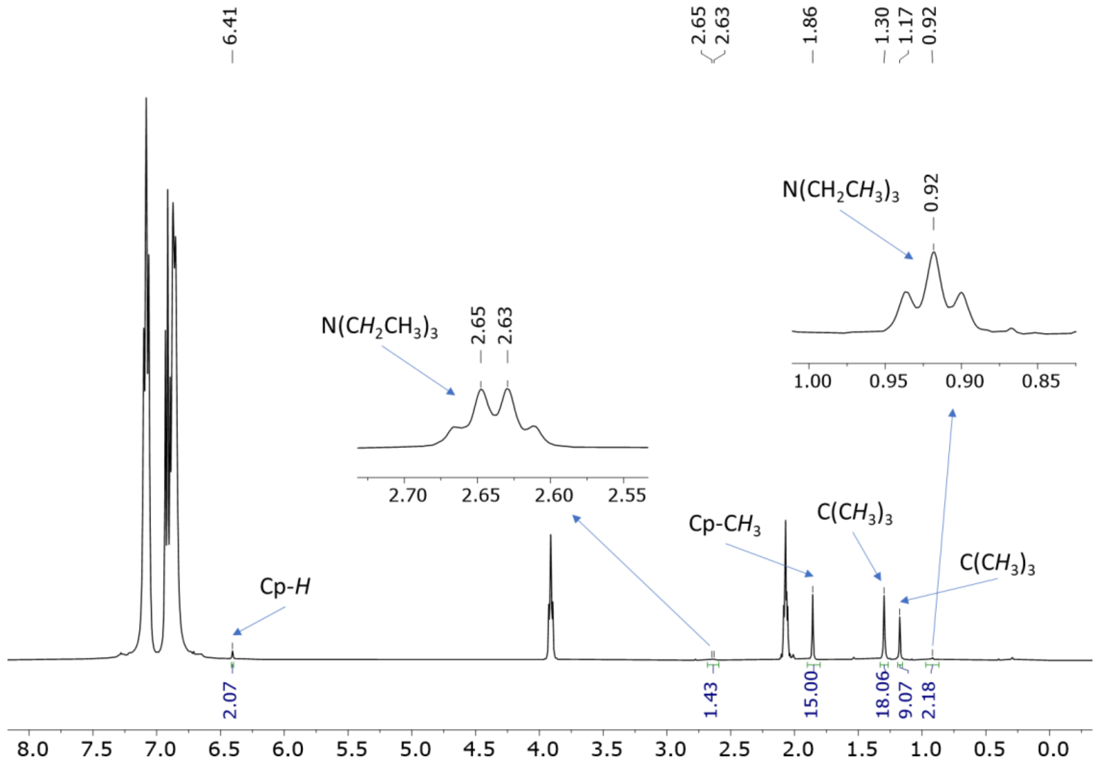


**Figure S45.** $^{1}H$ NMR spectrum of "[{Y(Cp$^{ttt}$)(Cp*)}{Al[OC($CF_3$)$_3$]$_4$}]" (400 MHz) in $C_6H_5F$ with a $C_4D_8O$ insert.

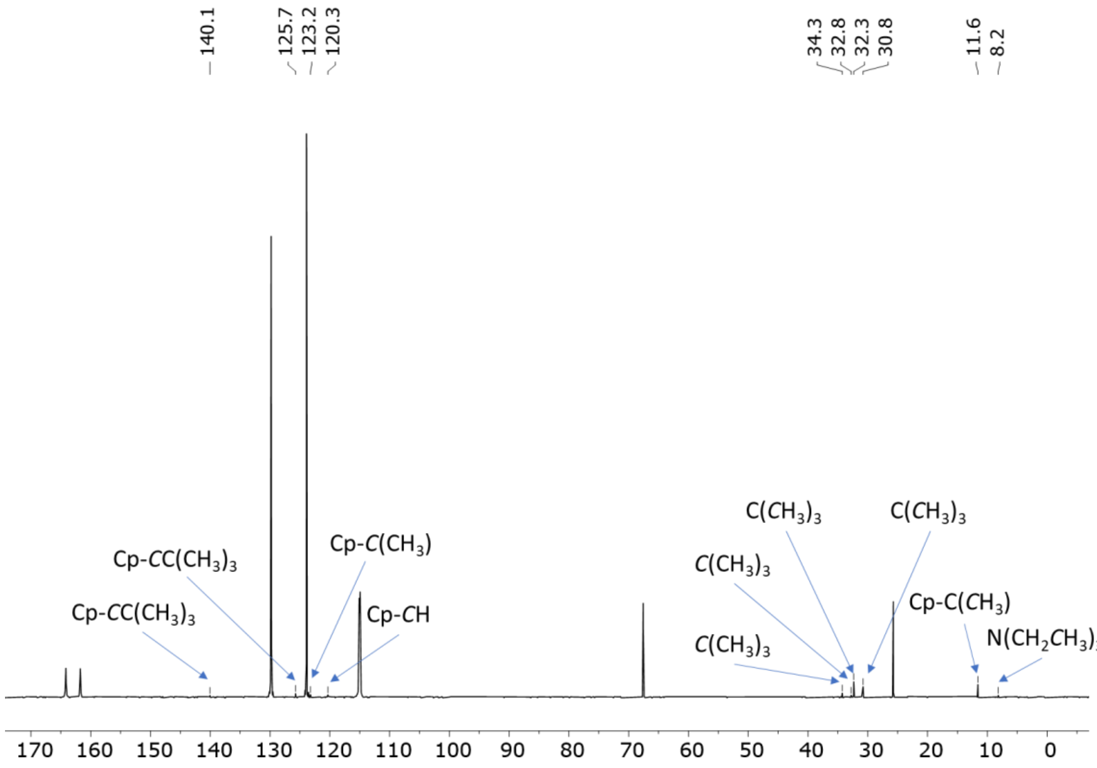


**Figure S46.** $^{13}C\{^{1}H\}$ NMR spectrum of "[{Y(Cp$^{ttt}$)(Cp*)}{Al[OC($CF_3$)$_3$]$_4$}]" (100 MHz) in $C_6H_5F$ with a $C_4D_8O$ insert.

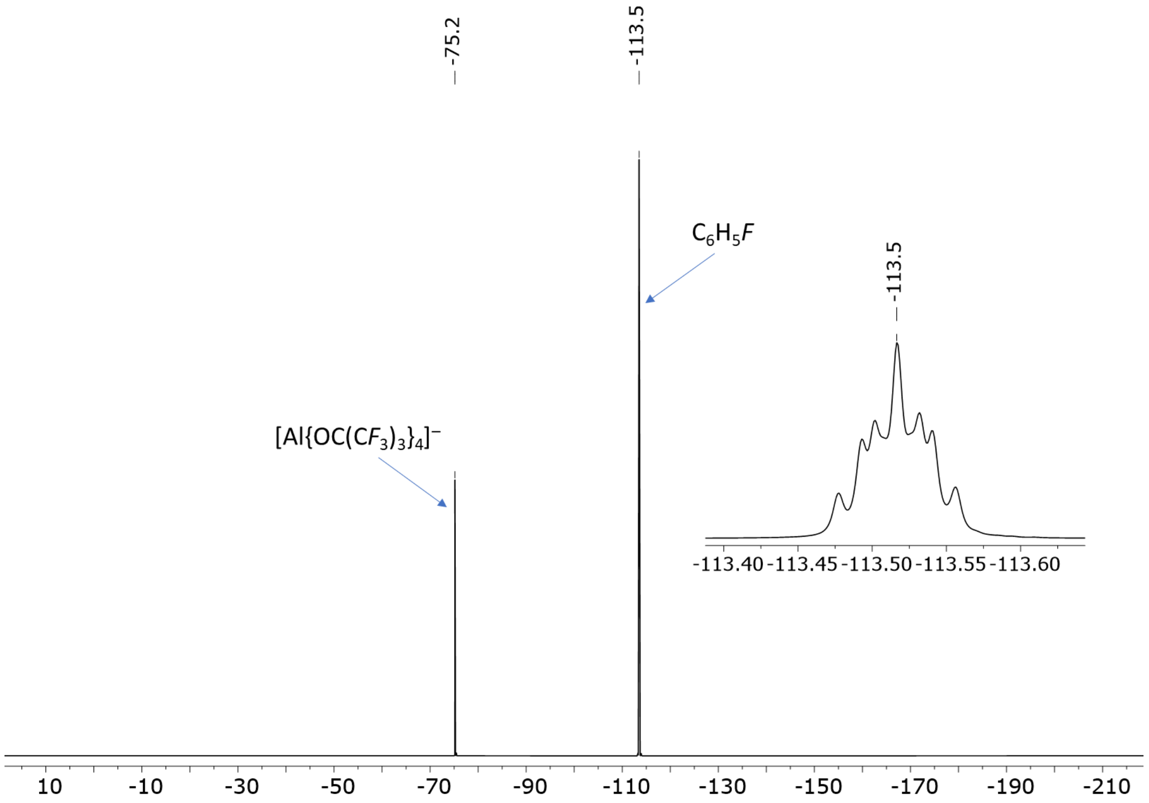


**Figure S47.** $^{19}$F NMR spectrum of “[{Y(Cp$^{ttt}$)(Cp*)}{Al[OC(CF$_3$)$_3$]$_4$}]” (376 MHz) in $C_6H_5F$ with a $C_6D_6$ insert.

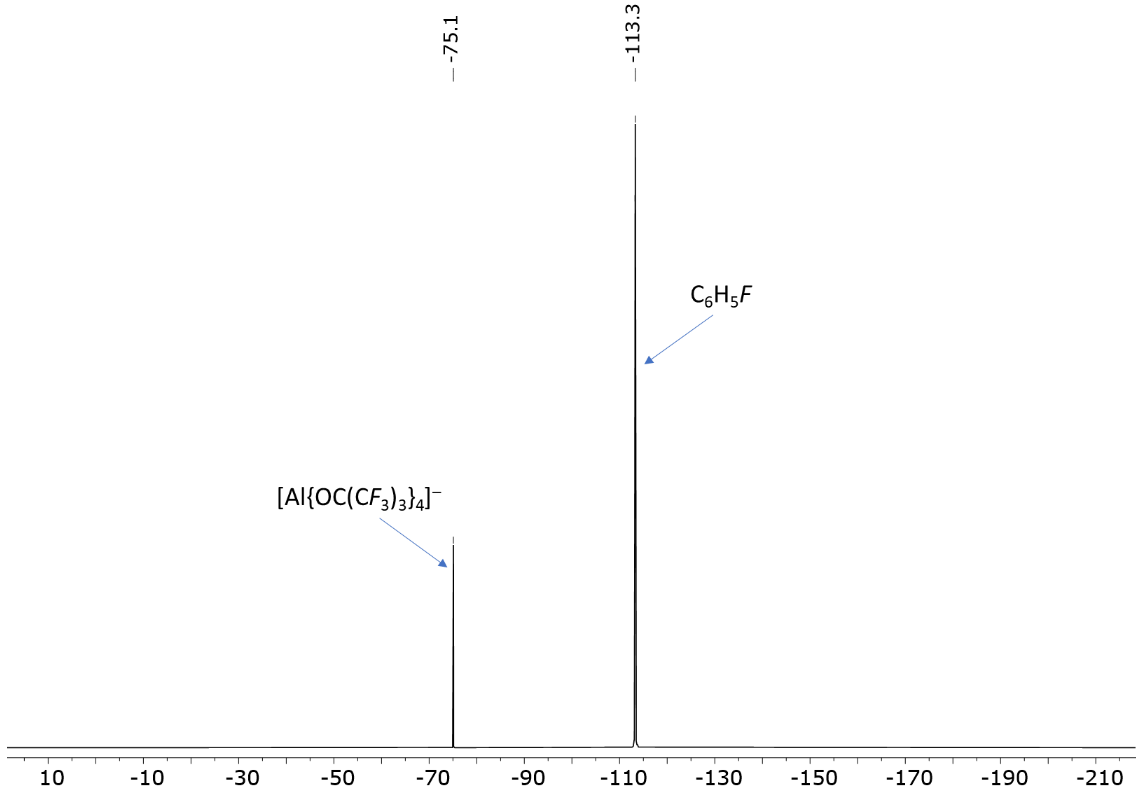


**Figure S48.** $^{19}$F{$^1$H} NMR spectrum of “[{Y(Cp$^{ttt}$)(Cp*)}{Al[OC(CF$_3$)$_3$]$_4$}]” (376 MHz) in $C_6H_5F$ with a $C_6D_6$ insert.

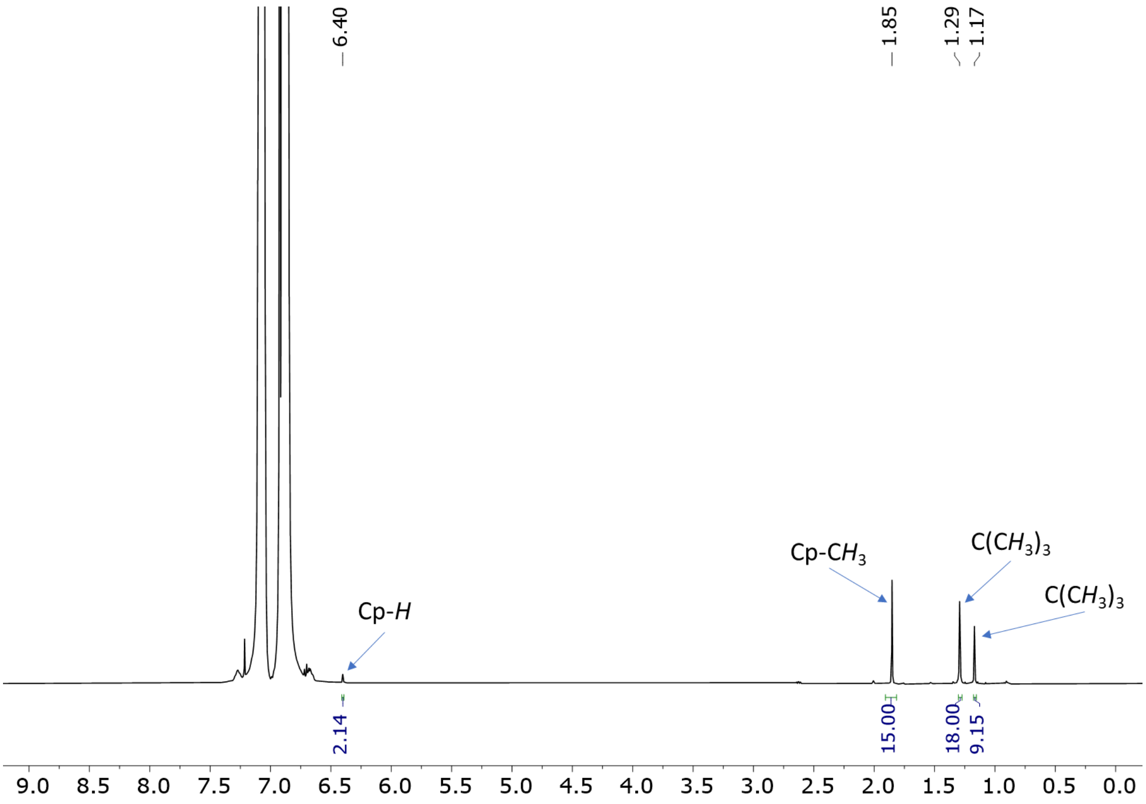


**Figure S49.** $^{1}$H NMR spectrum of **3-Y·C$_6$H$_6$** (400 MHz) in $C_6H_5F$ with a $C_6D_6$ insert.

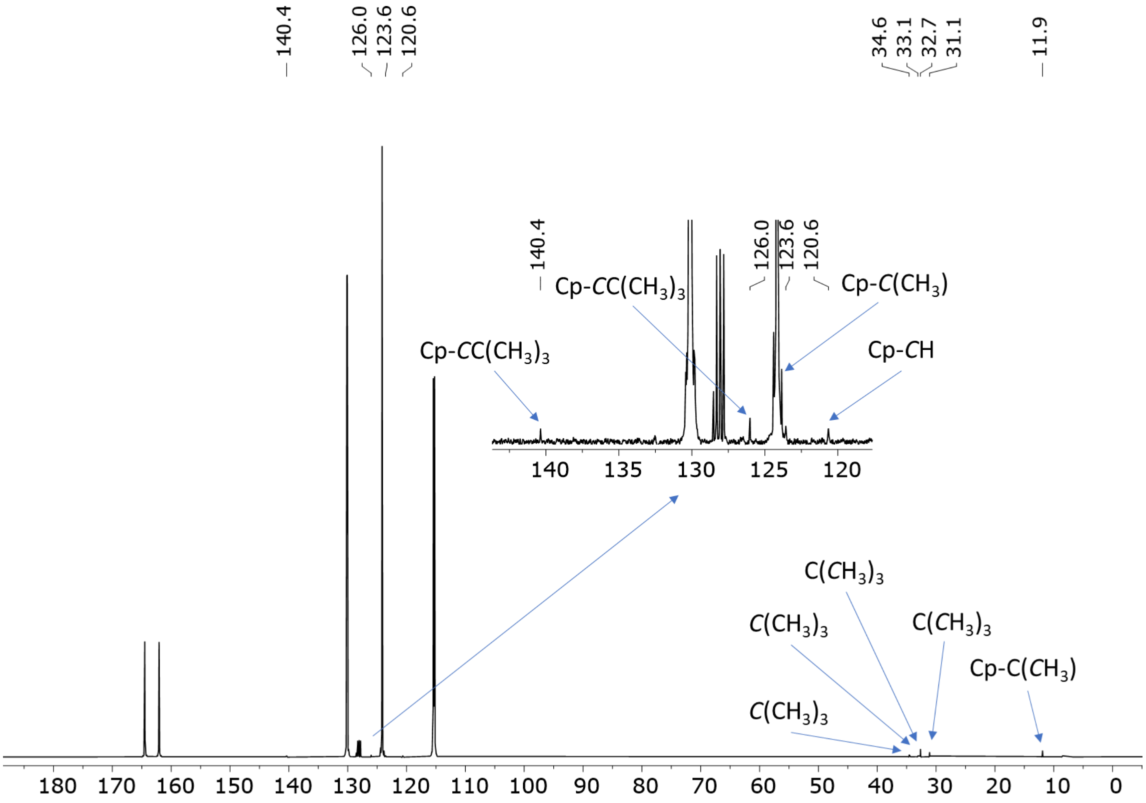


**Figure S50.** $^{13}$C{$^{1}$H} NMR spectrum of **3-Y·C$_6$H$_6$** (100 MHz) in $C_6H_5F$ with a $C_6D_6$ insert.

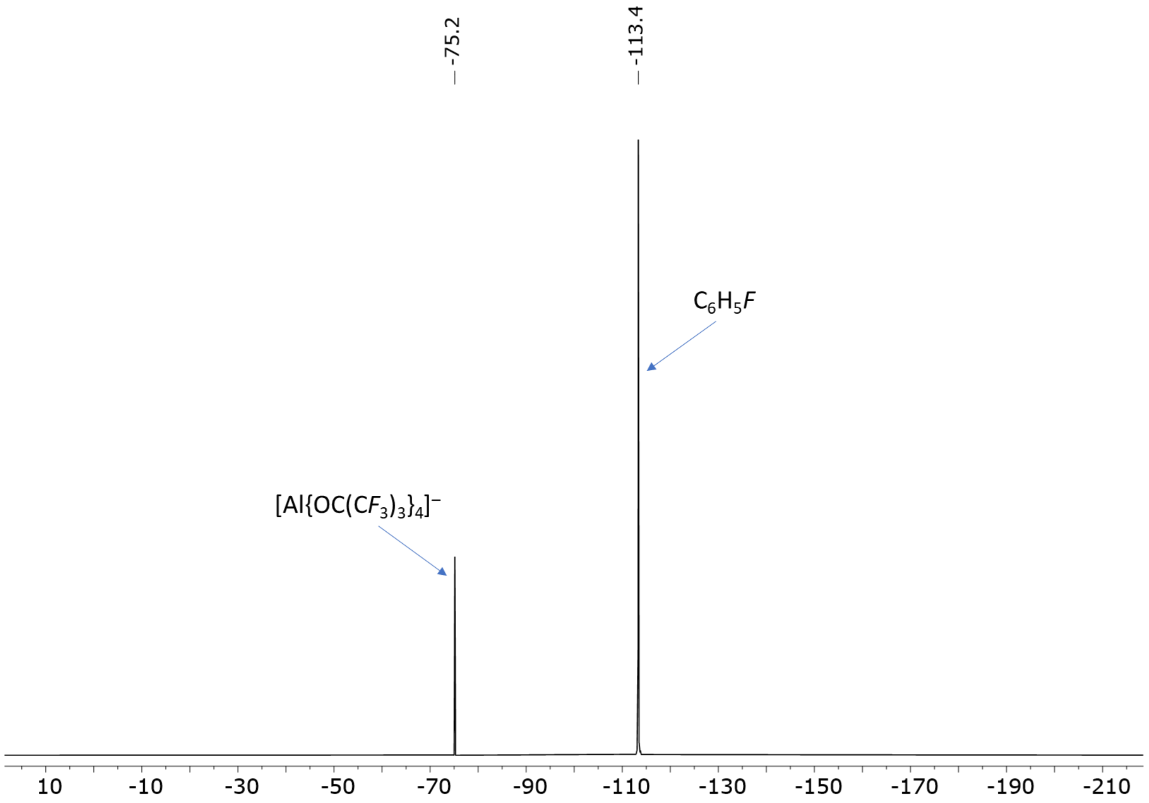


**Figure S51.** $^{19}F\{^1H\}$ NMR spectrum of **3-Y·C6H6** (376 MHz) in $C_6H_5F$ with a $C_6D_6$ insert.

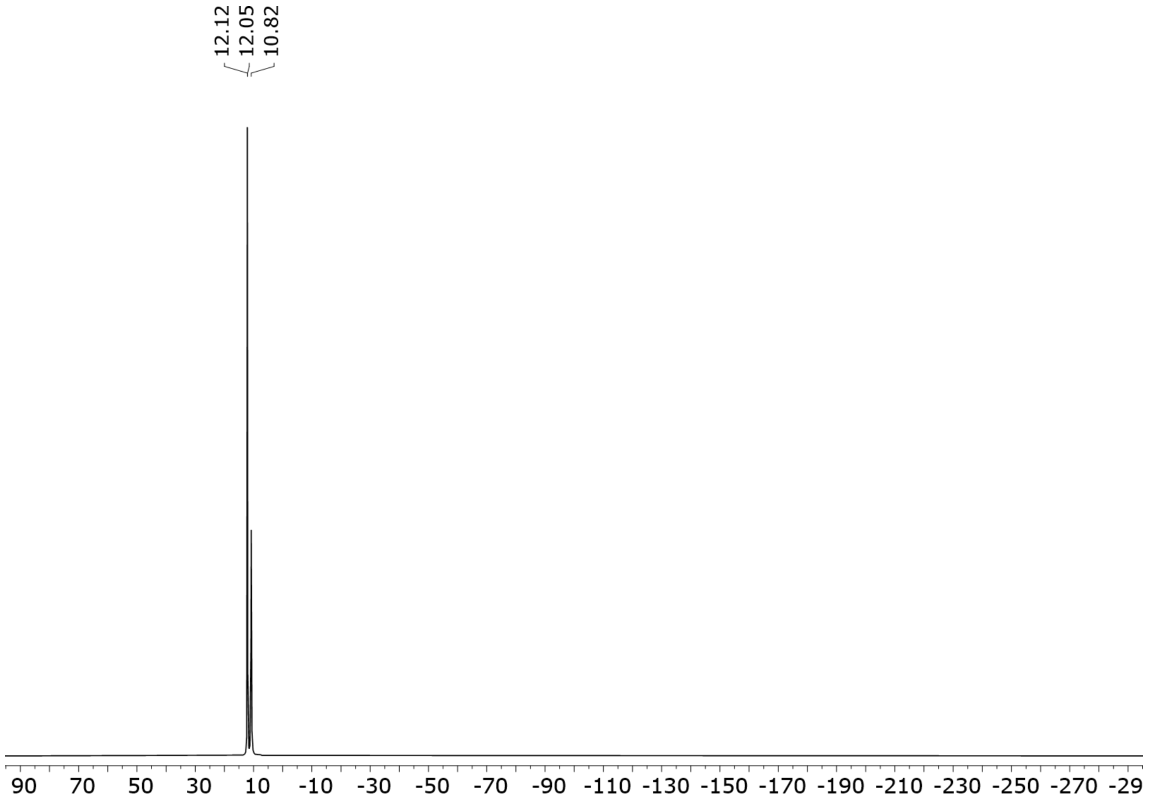


**Figure S52.** $^1H$ NMR spectrum of "[{Dy(Cp$^{ttt}$)(Cp*)}{Al[OC($CF_3$)$_3$]$_4$}]" (400 MHz) in $C_6H_5F$.

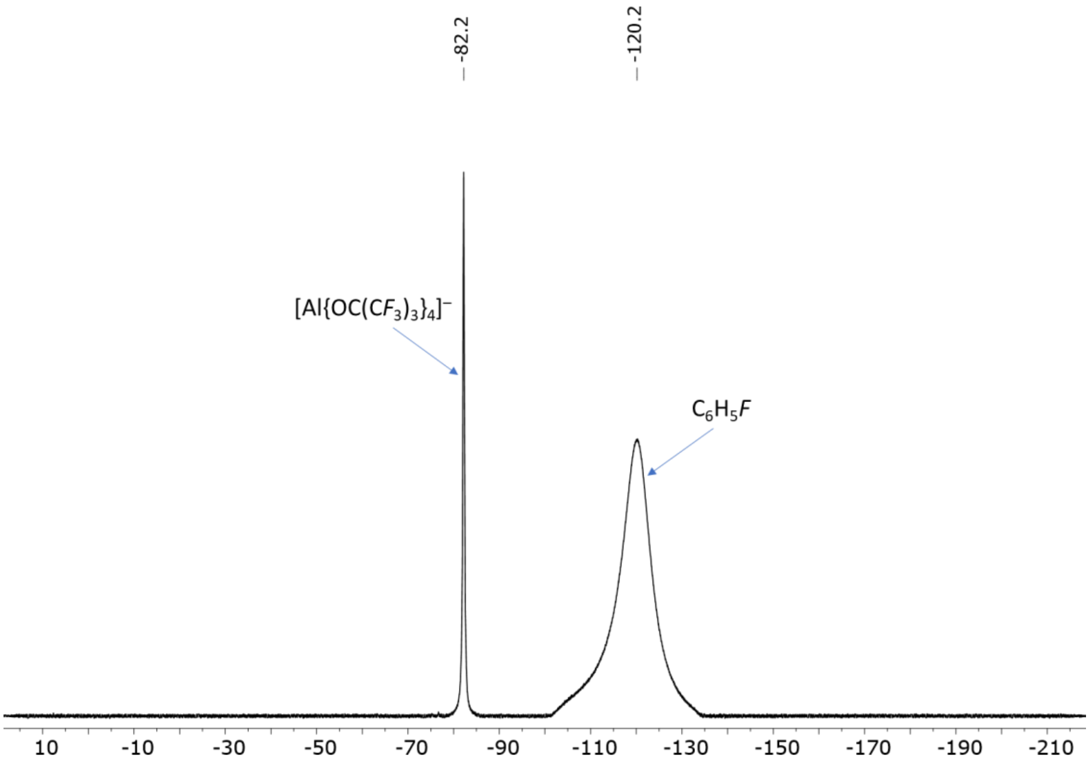


**Figure S53.** $^{19}F\{^{1}H\}$ NMR spectrum of "[{Dy(Cp$^{ttt}$)(Cp*)}{Al[OC($CF_3$)$_3$]$_4$}]" (376 MHz) in $C_6H_5F$.

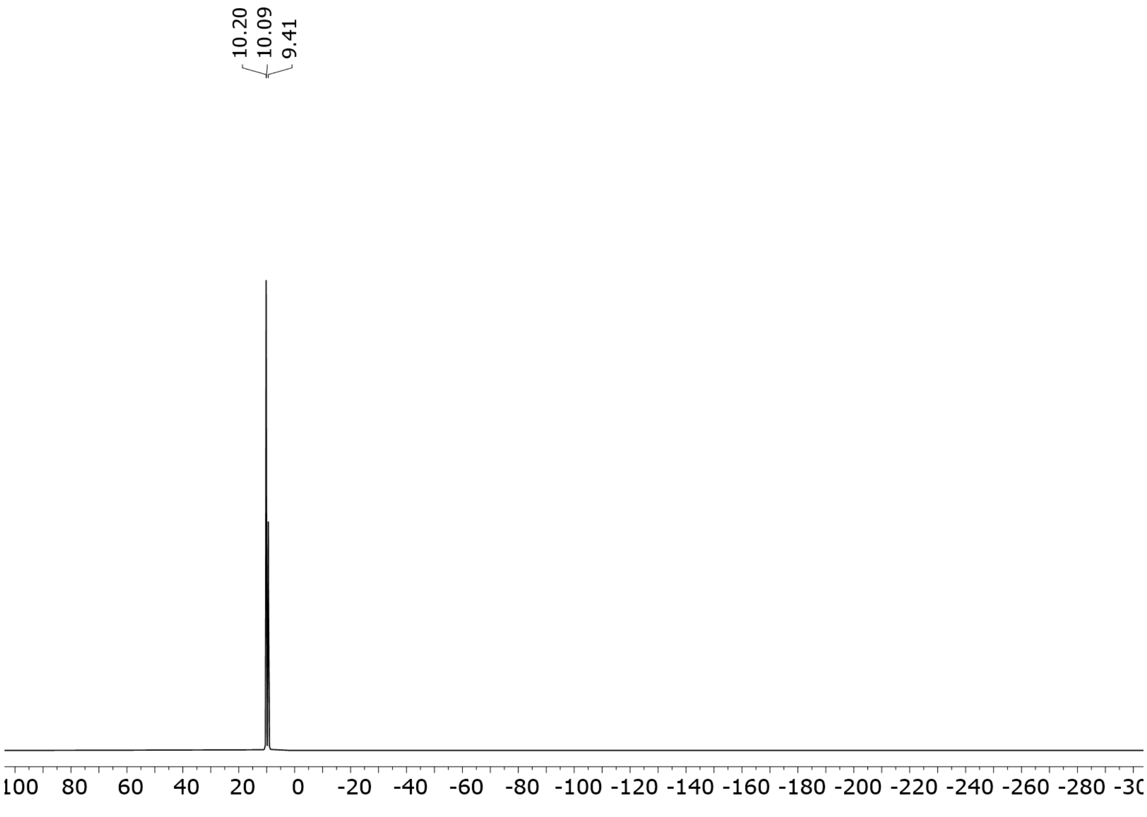


**Figure S54.** $^{1}H$ NMR spectrum of **3-Dy·$C_6H_6$** (400 MHz) in $C_6H_5F$.

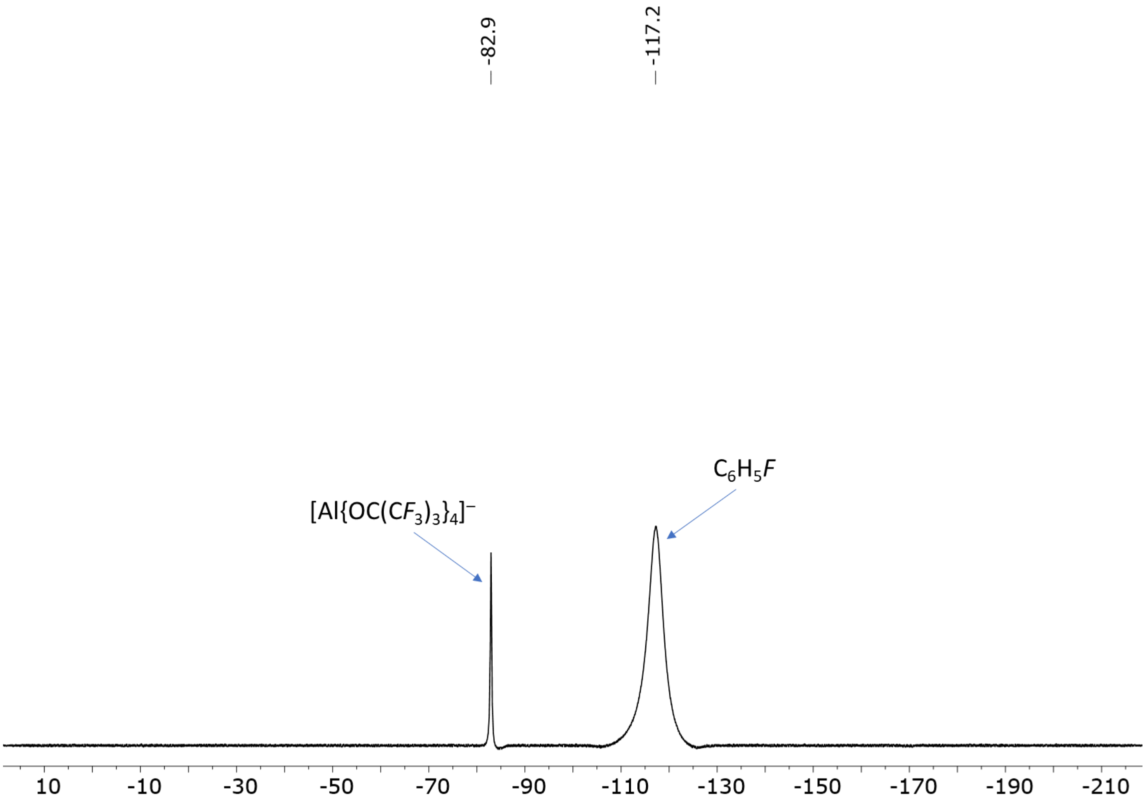


**Figure S55.** $^{19}F\{^{1}H\}$ NMR spectrum of **3-Dy·$C_6H_6$** (376 MHz) in $C_6H_5F$.

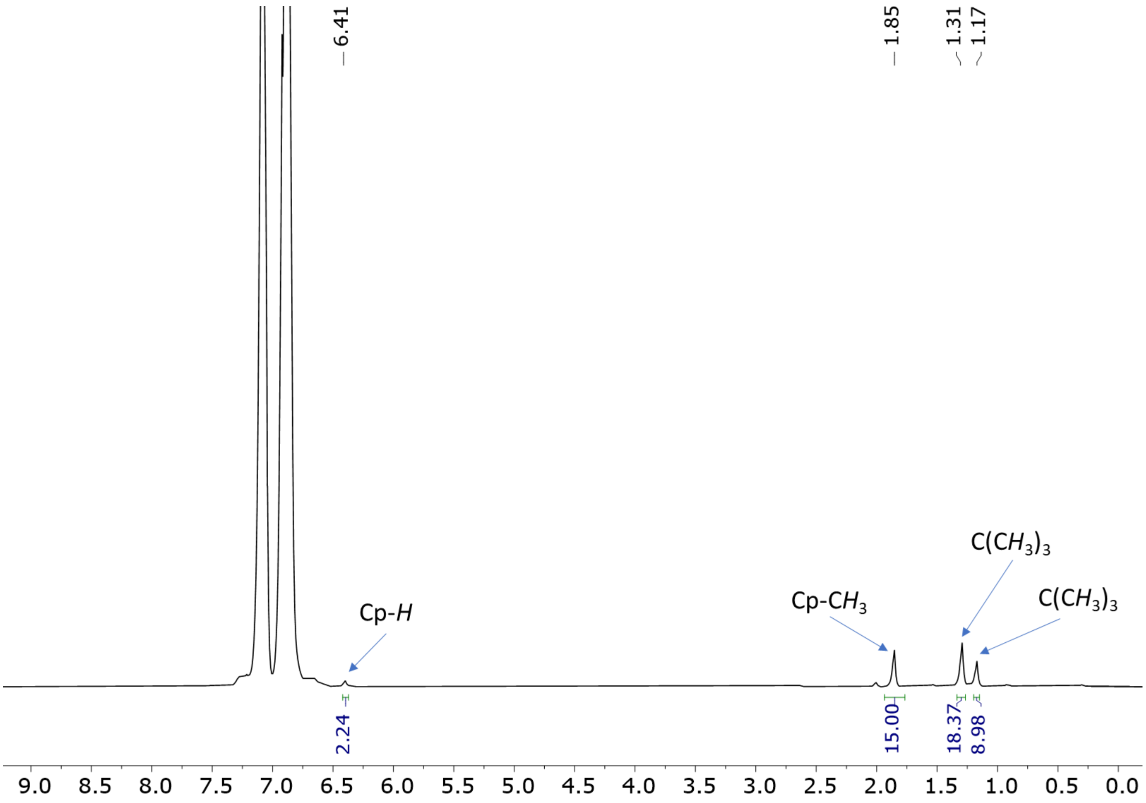


**Figure S56.** $^{1}H$ NMR spectrum of **4-Y** (400 MHz) in $C_6H_5F$ with a $C_6D_6$ insert.

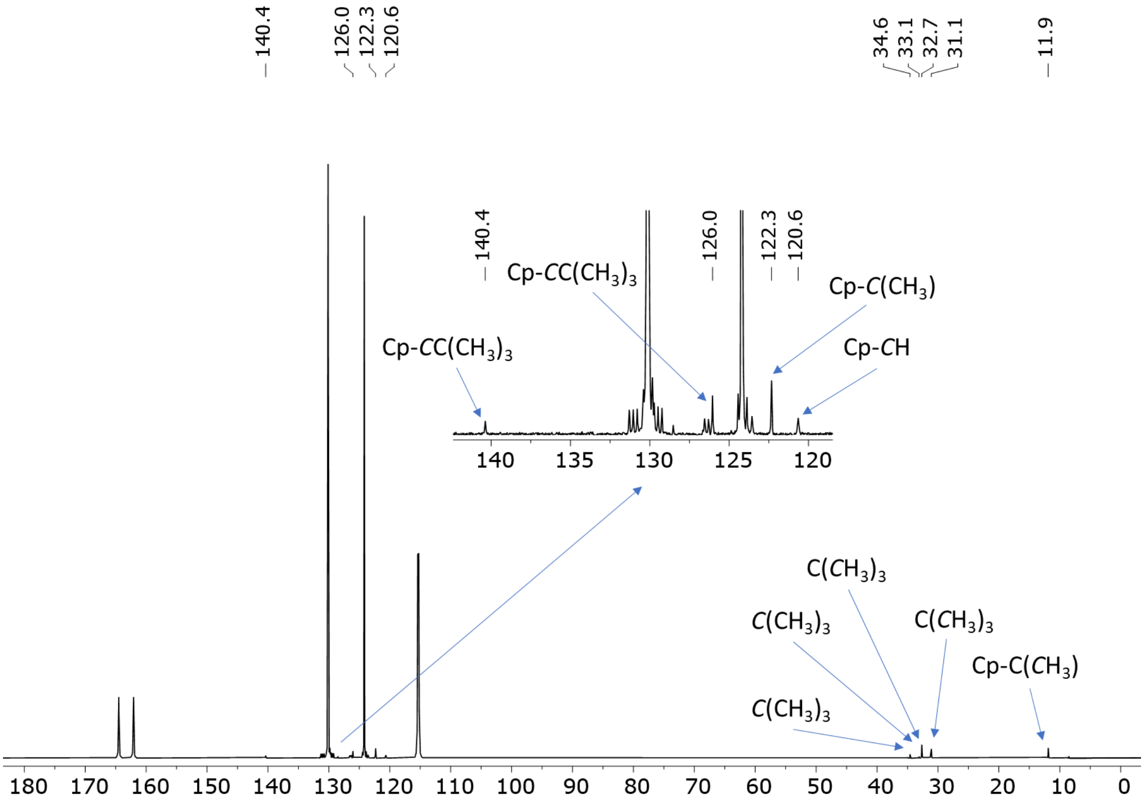


**Figure S57.** $^{13}C\{^{1}H\}$ NMR spectrum of **4-Y** (100 MHz) in $C_6H_5F$ with a $C_6D_6$ insert.

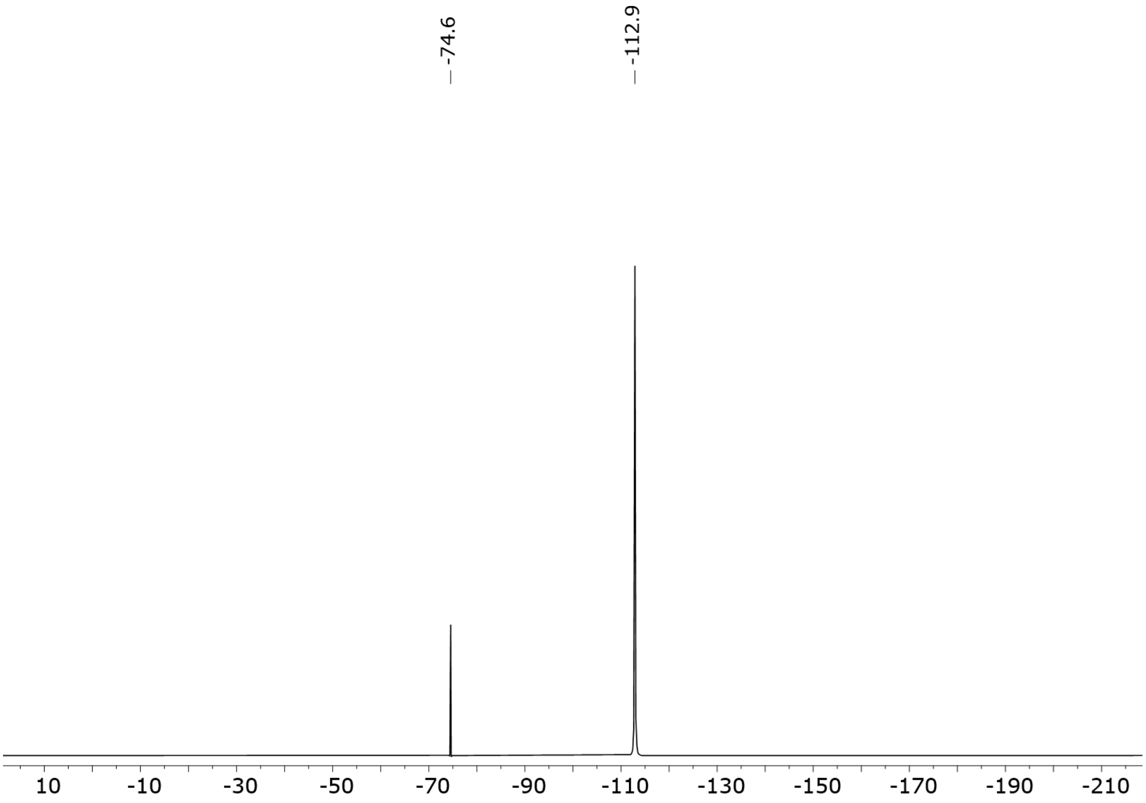


**Figure S58.** $^{19}F\{^{1}H\}$ NMR spectrum of **4-Y** (376 MHz) in $C_6H_5F$ with a $C_6D_6$ insert.

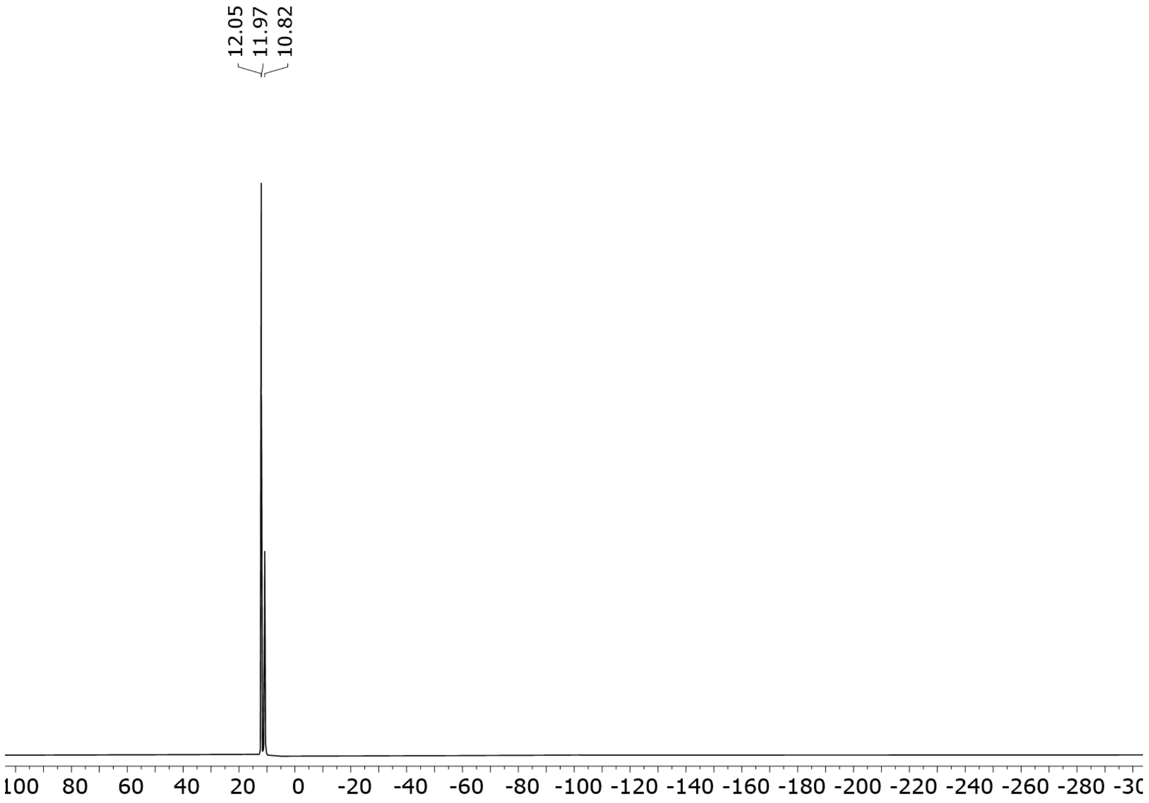


**Figure S59.** $^{1}H$ NMR spectrum of **4-Dy** (400 MHz) in $C_6H_5F$.

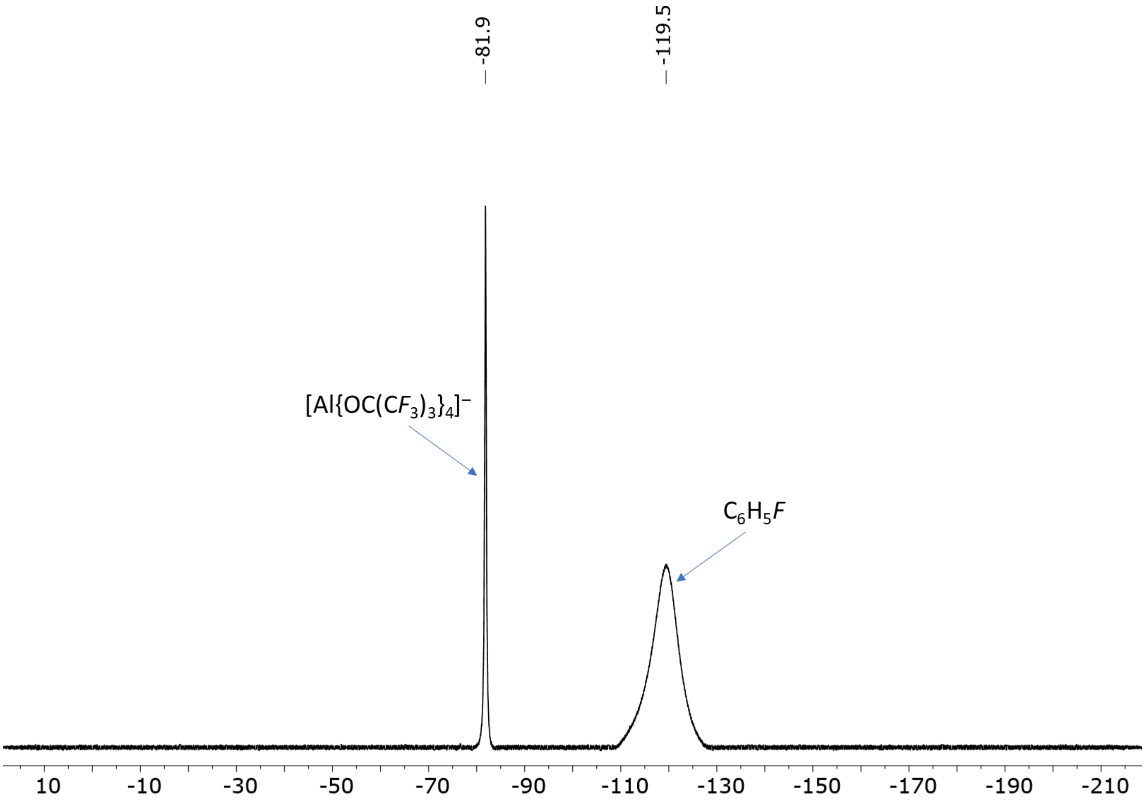


**Figure S60.** $^{19}F\{^{1}H\}$ NMR spectrum of **4-Dy** (376 MHz) in $C_6H_5F$.

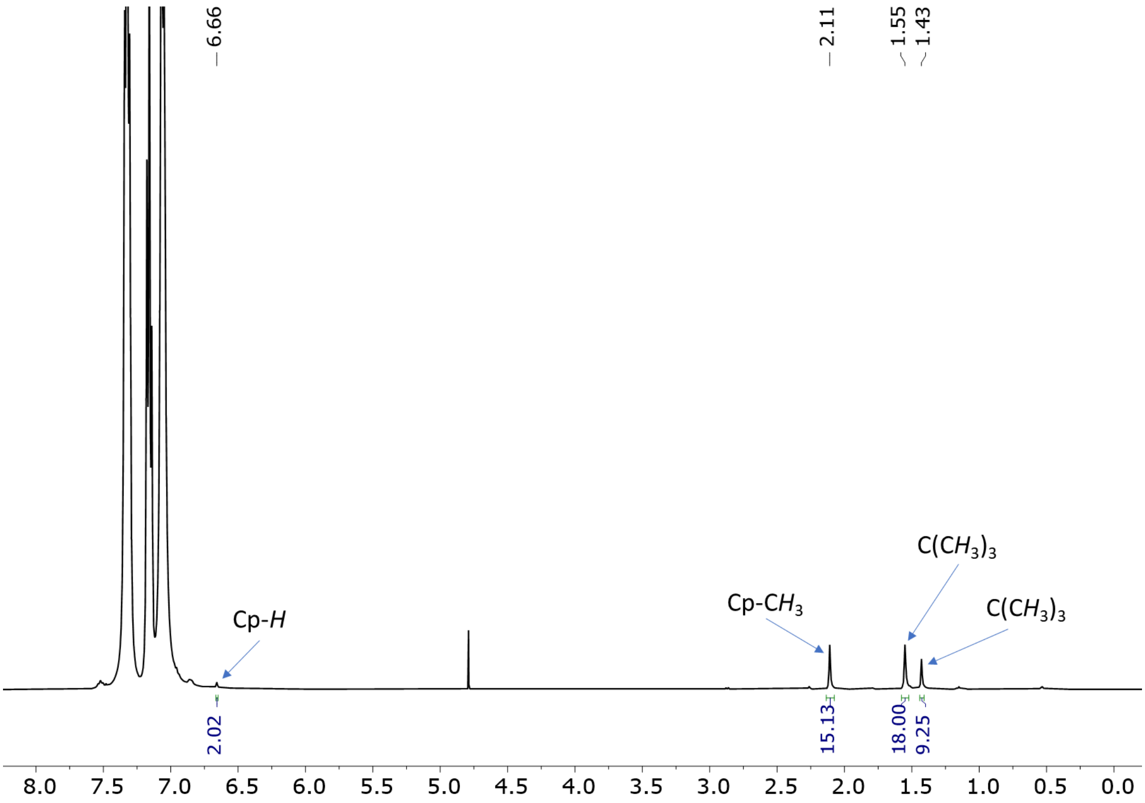


**Figure S61.** $^{1}H$ NMR spectrum of **5%Dy@4-Y** (400 MHz) in $C_6H_5F$ with a $D_2O$ insert.

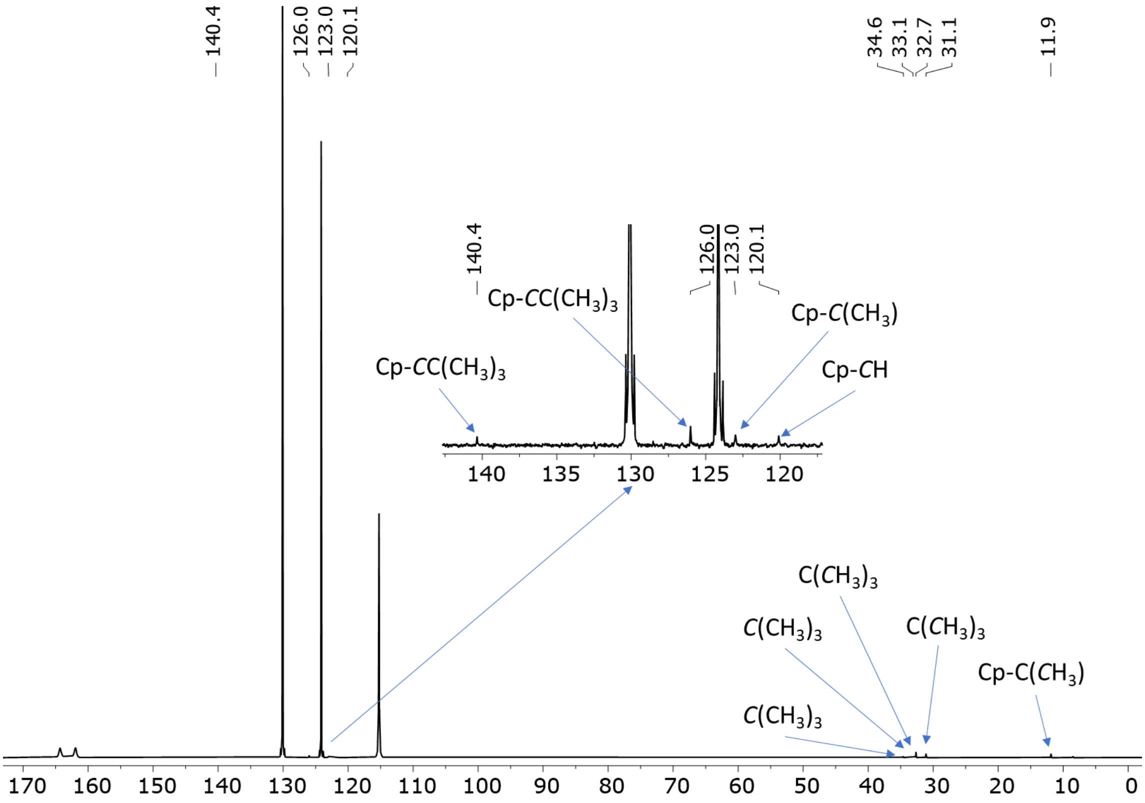


**Figure S62.** $^{13}C\{^{1}H\}$ NMR spectrum of **5%Dy@4-Y** (100 MHz) in $C_6H_5F$ with a $D_2O$ insert.

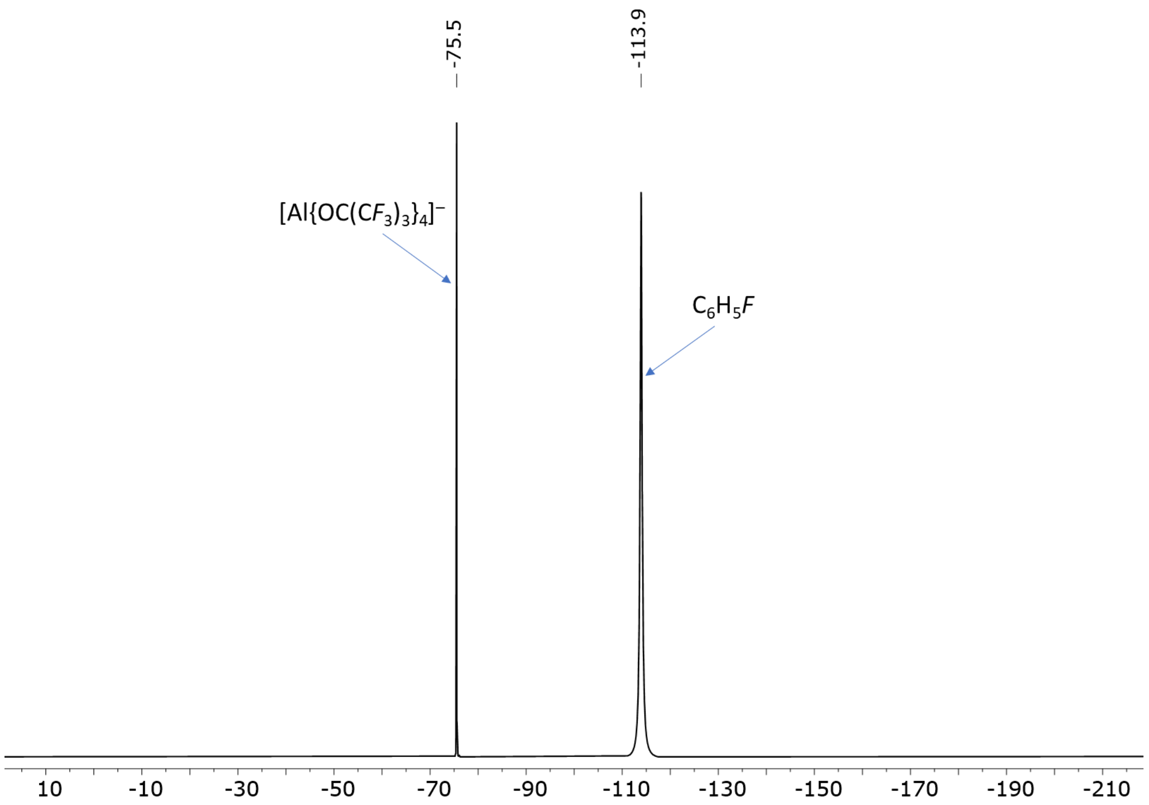


**Figure S63.** $^{19}$F NMR spectrum of **5%Dy@4-Y** (376 MHz) in $C_6H_5F$ with a $D_2O$ insert.

## 7. Single Crystal X-ray Diffraction

Crystals of **1-Ln**, **2-Dy**, **3-Ln·$C_6H_6$**, **4-Y**, **5%Dy@4-Y**, **5·$C_6H_5F$** and **6-Dy·$C_6H_{14}$** were examined using a Rigaku XtalLAB AFC11 diffractometer with a Hybrid Photon Counting area detector and mirror-monochromated Cu Kα (λ = 1.54178 Å) or Mo Kα radiation (λ = 0.71073 Å). Crystals of **2-Y** and **4-Dy** were examined using an Oxford Diffraction Supernova diffractometer, furnished with a CCD area detector and a mirror-monochromated Mo Kα radiation (λ = 0.71073 Å). Intensities were integrated from data recorded on 1° frames by ω or φ rotation. Cell parameters were refined from the observed positions of all strong reflections in each data set. A Gaussian grid face-indexed absorption correction with a beam profile was applied to all structures.[20] The structures were solved by direct and heavy atom methods using SHELXS or dual-space methods using SHELXT;[24] the datasets were refined by full-matrix least-squares on all unique $F^2$ values,[24] with anisotropic displacement parameters for all non-hydrogen atoms, and with constrained riding hydrogen geometries; $U_{iso}$(H) was set at 1.2 (1.5 for methyl groups) times $U_{eq}$ of the parent atom. The largest features in final difference syntheses were close to heavy atoms and were of no chemical significance. CrysAlisPro[20] was used for control and integration, and SHELXL[24,25] was employed through OLEX2[26] for structure solution and refinement. ORTEP-3[27] and POV-Ray[28] were employed for molecular graphics.

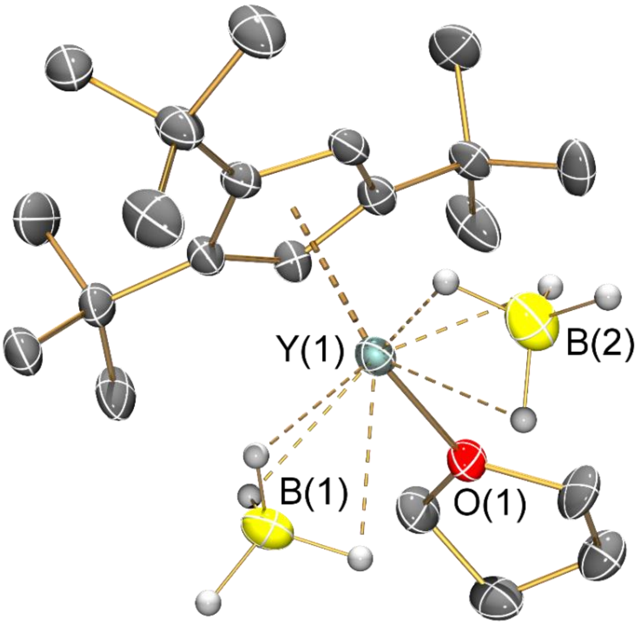


**Figure S64.** SCXRD structure of **1-Y** with select atom labeling (Y: cyan, C: grey, O: red, B: yellow, H: white). Displacement ellipsoids set at 30% probability levels; hydrogen atoms are omitted for clarity, with the exception of those belonging to the $BH_4^-$ groups. Selected bond lengths and angles: Y(1)···$Cp^{ttt}_{centroid}$ 2.345(2) Å, Y(1)···B(1) 2.509(8) Å, Y(1)···B(2) 2.490(8) Å, Y(1)–O(1) 2.32(3) Å, B(1)···Y(1)···(B2) 106.1(3)°, B(1)···Y(1)–O(1) 91.2(11)°, B(2)···Y(1)–O(1) 99.3(7)°, $Cp^{ttt}_{centroid}$···Y(1)···B(1) 123.4(2)°, $Cp^{ttt}_{centroid}$···Y(1)···B(2) 119.6(2)°, $Cp^{ttt}_{centroid}$···Y(1)–O(1) 111.0(10).°

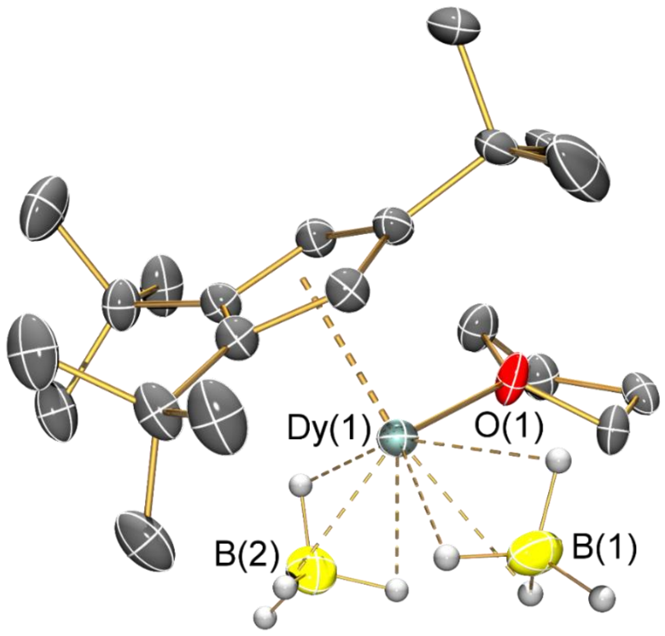


**Figure S65.** SCXRD structure of **1-Dy** with select atom labeling (Dy: cyan, C: grey, O: red, B: yellow, H: white). Displacement ellipsoids set at 30% probability levels; hydrogen atoms are omitted for clarity, with the exception of those belonging to the $BH_4^-$ groups. Selected bond lengths and angles: Dy(1)···$Cp^{ttt}_{centroid}$ 2.351(2) Å, Dy(1)···B(1) 2.510(6) Å, Dy(1)···B(2) 2.511(9) Å, Dy(1)–O(1) 2.34(2) Å, B(1)···Dy(1)···(B2) 105.8(2)°, B(1)···Dy(1)–O(1) 99.5(6)°, B(2)···Dy(1)–O(1) 91.7(10)°, $Cp^{ttt}_{centroid}$···Dy(1)···B(1) 119.9(2)°, $Cp^{ttt}_{centroid}$···Dy(1)···B(2) 123.7(2)°, $Cp^{ttt}_{centroid}$···Dy(1)–O(1) 110.1(9)°.

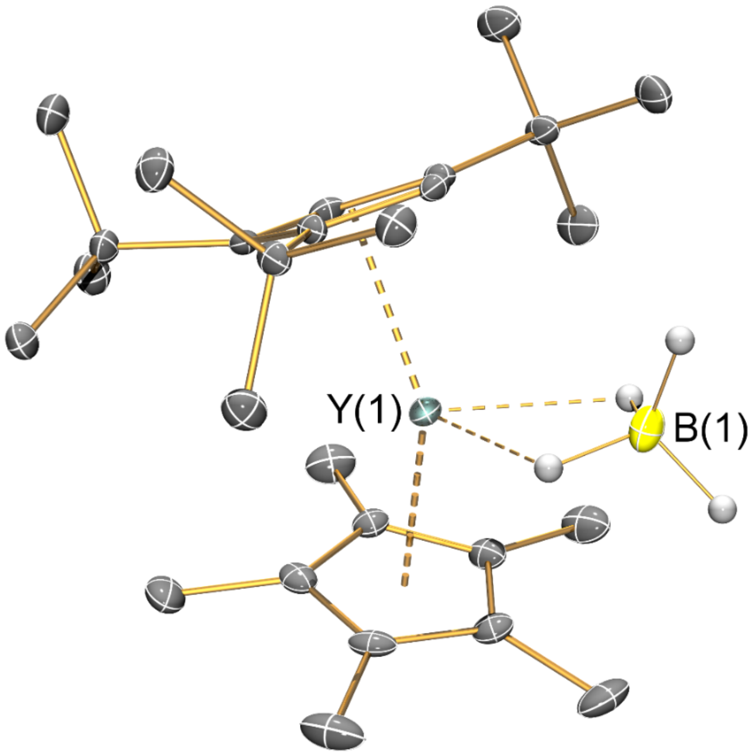


**Figure S66.** SCXRD structure of **2-Y** with select atom labeling (Y: cyan, C: grey, B: yellow, H: white). Displacement ellipsoids set at 30% probability levels; hydrogen atoms are omitted for clarity, with the exception of those belonging to the $BH_4^-$ group. Selected bond lengths and angles: Y(1)···$Cp^{ttt}_{centroid}$ 2.353(2) Å, Y(1)···$Cp^*_{centroid}$ 2.350(2) Å, Y(1)···B(1) 2.723(6) Å, $Cp^{ttt}_{centroid}$···Y(1)···$Cp^*_{centroid}$ 144.80(7)°, $Cp^{ttt}_{centroid}$···Y(1)···B(1) 106.23(13)°, $Cp^*_{centroid}$···Y(1)···B(1) 108.13(13)°.

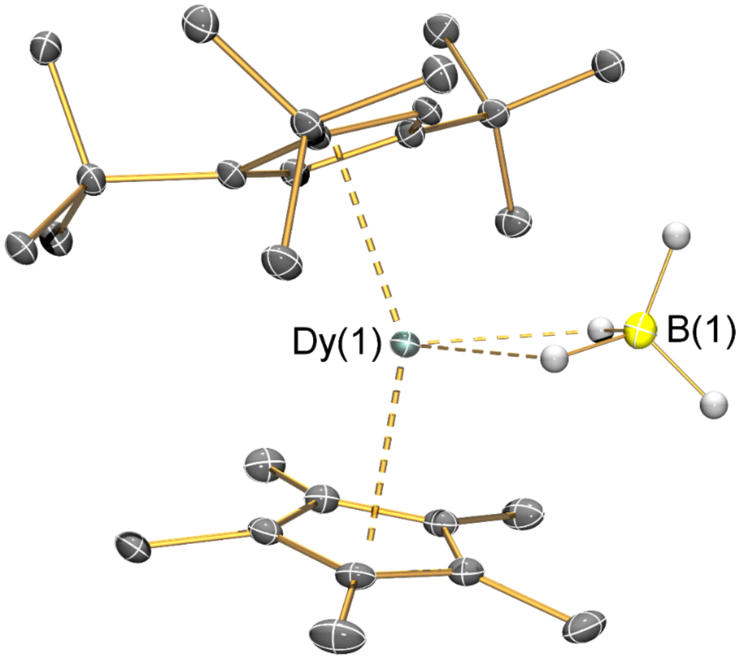


**Figure S67.** SCXRD structure of **2-Dy** with select atom labeling (Dy: cyan, C: grey, B: yellow, H: white). Displacement ellipsoids set at 30% probability levels; hydrogen atoms are omitted for clarity, with the exception of those belonging to the $BH_4^-$ group. Selected bond lengths and angles: Dy(1)···$Cp^{ttt}_{centroid}$ 2.35450(8) Å, Dy(1)···$Cp^*_{centroid}$ 2.35253(8) Å, Dy(1)···B(1) 2.726(3) Å, $Cp^{ttt}_{centroid}$···Dy(1)···$Cp^*_{centroid}$ 145.030(4)°, $Cp^{ttt}_{centroid}$···Dy(1)···B(1) 105.86(6)°, $Cp^*_{centroid}$···Dy(1)···B(1) 108.09(6)°.

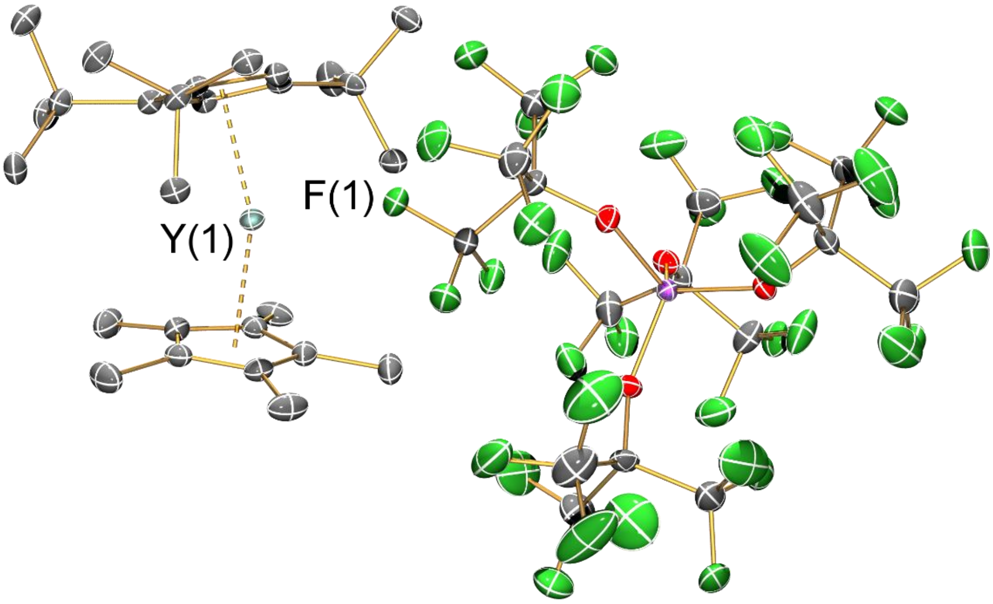


**Figure S68.** SCXRD structure of **3-Y·$C_6H_6$** with select atom labeling (Y: cyan, C: grey, O: red, F: green, Al: purple). Displacement ellipsoids set at 30% probability levels; hydrogen atoms and the lattice solvent (benzene) are omitted for clarity. Selected bond lengths and angles: Y(1)···$Cp^{ttt}_{centroid}$ 2.2848(4) Å, Y(1)···$Cp^{*}_{centroid}$ 2.3058(4) Å, Y(1)···F(1) 3.103(4) Å, Y(1)···C(9) 2.945(5) Å, Y(1)···H 2.5079 Å, $Cp^{ttt}_{centroid}$···Y(1)···$Cp^{*}_{centroid}$ 148.77(2)°, $Cp^{ttt}_{centroid}$···Y(1)···F(1) 98.63(6)°, $Cp^{*}_{centroid}$···Y(1)···F(1) 112.18(6)°, Y(1)···F(1)–C(29) 141.9(3)°.

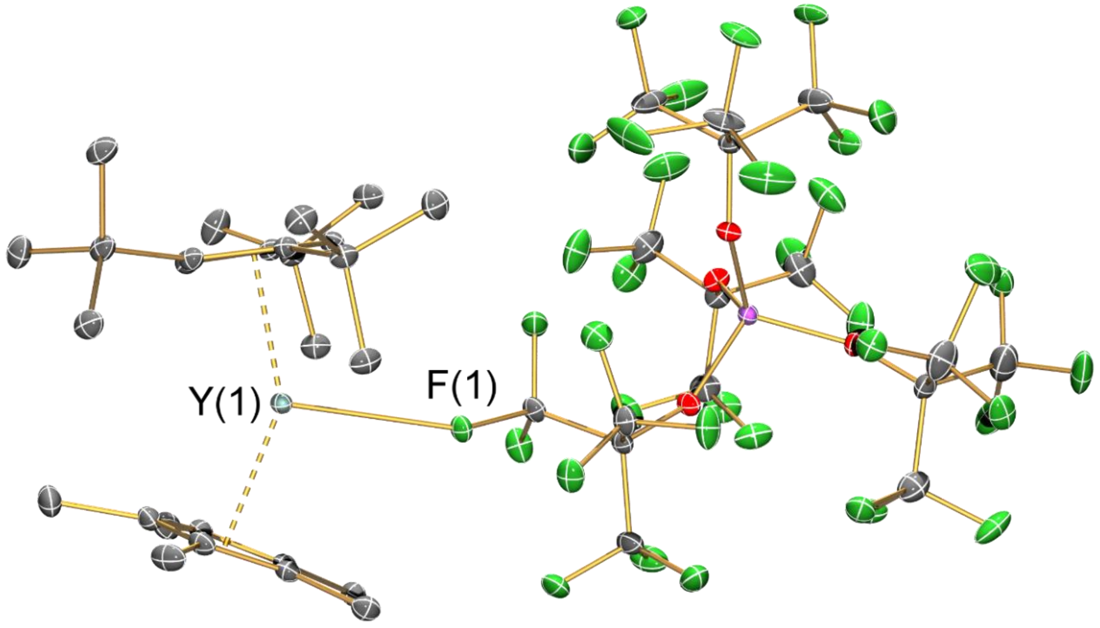


**Figure S69.** SCXRD structure of **4-Y** with select atom labeling (Y: cyan, C: grey, O: red, F: green, Al: purple). Displacement ellipsoids set at 30% probability levels; hydrogen atoms are omitted for clarity. Selected bond lengths and angles: Y(1)···$Cp^{ttt}_{centroid}$ 2.3077(2) Å, Y(1)···$Cp^{*}_{centroid}$ 2.2991(2) Å, Y(1)–F(1) 2.783(2) Å, Y(1)···C(9) 2.975(3) Å, Y(1)···H 2.5624 Å, $Cp^{ttt}_{centroid}$···Y(1)···$Cp^{*}_{centroid}$ 147.987(11)°, $Cp^{ttt}_{centroid}$···Y(1)–F(1) 108.76(4)°, $Cp^{*}_{centroid}$···Y(1)–F(1) 103.04(4)°, Y(1)–F(1)–C(29) 149.4(2)°.

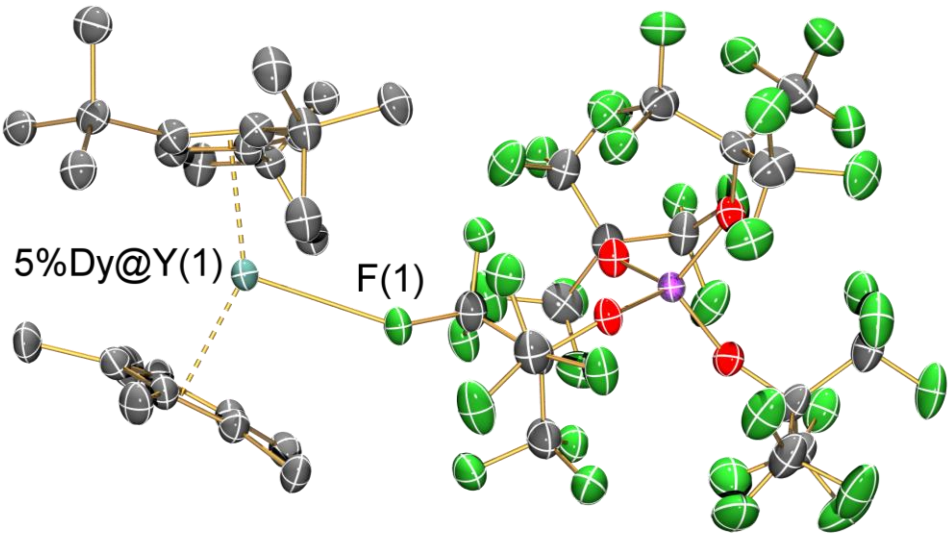


**Figure S70.** SCXRD structure of **5%Dy@4-Y** with select atom labeling (Y: cyan, C: grey, O: red, F: green, Al: purple). Displacement ellipsoids set at 30% probability levels; hydrogen atoms are omitted for clarity. Selected bond lengths and angles: 5%Dy@Y(1)···$Cp^{ttt}_{centroid}$ 2.298(4) Å, 5%Dy@Y(1)···$Cp^{*}_{centroid}$ 2.300(4) Å, 5%Dy@Y(1)–F(1) 2.710(4) Å, C(29)–F(1) 1.421(9) Å, 5%Dy@Y(1)···C(7) 3.003(9) Å, 5%Dy@Y(1)···H 2.5781 Å, shortest 5%Dy@Y(1)···5%Dy@Y(1) 9.2206(13) Å, $Cp^{ttt}_{centroid}$···5%Dy@Y(1)···$Cp^{*}_{centroid}$ 144.52(12)°, $Cp^{ttt}_{centroid}$···5%Dy@Y(1)–F(1) 114.60(14)°, $Cp^{*}_{centroid}$···5%Dy@Y(1)–F(1) 99.39(14)°, 5%Dy@Y(1)–F(1)–C(29) 142.8(5)°.

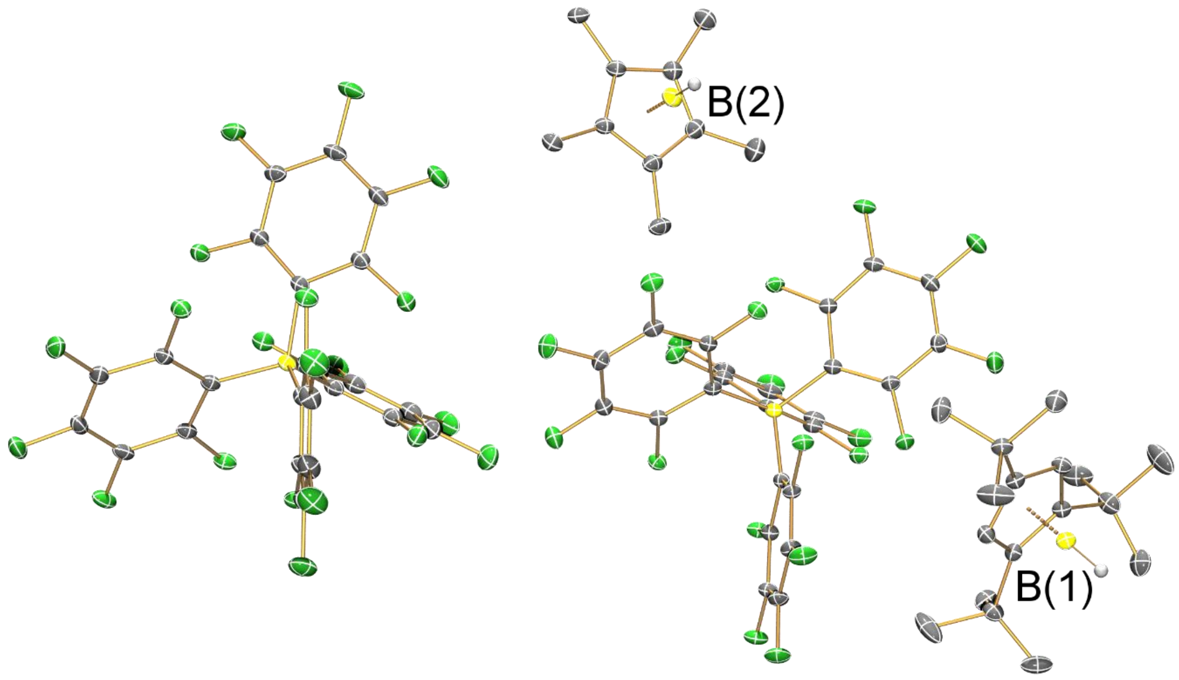


**Figure S71.** SCXRD structure of **5·$C_6H_5F$** with select atom labeling (C: grey, B: yellow, H: white, F: green). Displacement ellipsoids set at 30% probability levels; hydrogen atoms, with the exception of those belonging to the borinium cations, and the fluorobenzene lattice solvent have been omitted for clarity. Selected bond lengths and angles: $Cp^{ttt}_{centroid}$···B(1) 1.240(2) Å, $Cp^{*}_{centroid}$···B(2) 1.235(2) Å.

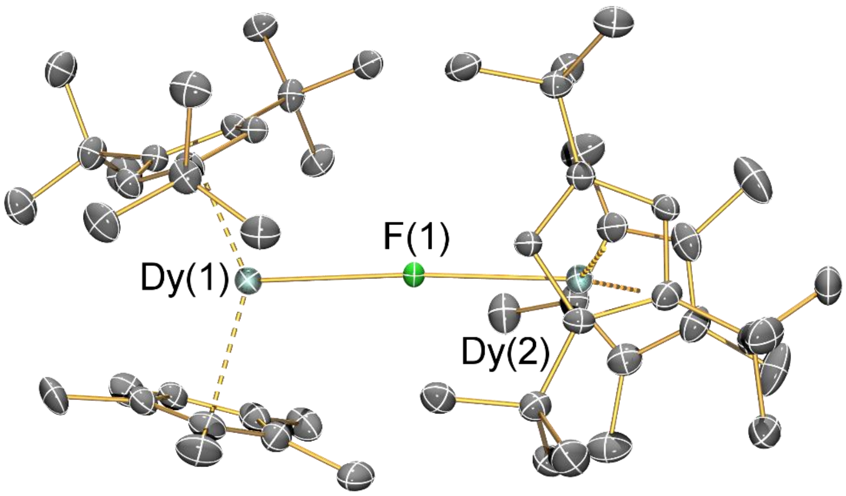


**Figure S72.** SCXRD structure of **6-Dy·$C_6H_{14}$** with select atom labeling (Dy: cyan, C: grey, F: green). Displacement ellipsoids set at 30% probability levels; hydrogen atoms, the $[Al\{OC(CF_3)_3\}_4]^-$ anion and the *n*-hexane lattice solvent have been omitted for clarity. Selected averaged bond lengths and angles: Dy···$Cp^{ttt}_{centroid}$ 2.3311(6) Å, Dy···$Cp^{*}_{centroid}$ 2.3369(6) Å, Dy–F(1) 2.771(4) Å, $Cp^{ttt}_{centroid}$···Dy···$Cp^{*}_{centroid}$ 142.92(3)°, $Cp^{ttt}_{centroid}$···Dy–F(1) 107.72(10)°, $Cp^{*}_{centroid}$···Dy–F(1) 108.02(11)°.

**Table S8.** Crystallographic data for **1-Y** and **1-Dy**.

| | **1-Y** | **1-Dy** |
|---|---|---|
| formula | $C_{21}H_{45}B_2OY$ | $C_{21}H_{45}B_2DyO$ |
| molecular mass, g $mol^{-1}$ | 424.10 | 497.69 |
| cryst size, mm | 0.446 × 0.103 × 0.076 | 0.361 × 0.204 × 0.082 |
| cryst syst | monoclinic | monoclinic |
| space group | $P2_1/c$ | $P2_1/c$ |
| collection temperature, K | 150(2) | 150(2) |
| a, Å | 18.1859(6) | 18.1792(3) |
| b, Å | 9.0270(2) | 9.04686(11) |
| c, Å | 16.4933(4) | 16.4942(3) |
| α, ° | 90 | 90 |
| β, ° | 113.879(3) | 113.863(2) |
| γ, ° | 90 | 90 |
| V, $Å^3$ | 2475.84(13) | 2480.80(8) |
| Z | 4 | 4 |
| $\rho_{calcd}$, g $cm^{-3}$ | 1.138 | 1.333 |
| μ, $mm^{-1}$ | 3.329 | 16.125 |
| no. of reflections made | 11579 | 23670 |
| no. of unique reflns, $R_{int}$ | 4913, 0.0387 | 5067, 0.0786 |
| no. of reflns with $F^2 > 2\sigma(F^2)$ | 4002 | 4568 |
| transmn coeff range | 0.472–1.000 | 0.056–0.848 |
| R, $R_w$[a] ($F^2 > 2\sigma(F^2)$) | 0.0559, 0.1537 | 0.0569, 0.1483 |
| R, $R_w$[a] (all data) | 0.0658, 0.1610 | 0.0598, 0.1560 |
| S[a] | 1.092 | 1.010 |
| parameters, restraints | 335, 528 | 335, 568 |
| max., min. diff map, e $Å^{-3}$ | 1.227, –1.446 | 2.664, –2.139 |

[a] Conventional $R = \Sigma||F_o| - |F_c||/\Sigma|F_o|$; $R_w = [\Sigma w(F_o^2 - F_c^2)^2/\Sigma w(F_o^2)^2]^{1/2}$; $S = [\Sigma w(F_o^2 - F_c^2)^2/\text{no. data} - \text{no. params})]^{1/2}$ for all data.

**Table S9.** Crystallographic data for **2-Y** and **2-Dy**.

| | **2-Y** | **2-Dy** |
|---|---|---|
| formula | $C_{27}H_{48}BY$ | $C_{27}H_{48}BDy$ |
| molecular mass, g mol$^{-1}$ | 472.37 | 545.96 |
| cryst size, mm | 0.257 × 0.091 × 0.073 | 0.117 × 0.097 × 0.058 |
| cryst syst | monoclinic | monoclinic |
| space group | $P2_1/c$ | $P2_1/c$ |
| collection temperature, K | 150(2) | 100(2) |
| a, Å | 8.8052(8) | 8.79620(9) |
| b, Å | 11.6029(7) | 11.55732(11) |
| c, Å | 26.1332(17) | 26.0045(2) |
| α, ° | 90 | 90 |
| β, ° | 91.747(6) | 91.4976(8) |
| γ, ° | 90 | 90 |
| V, Å$^3$ | 2668.7(3) | 2642.73(4) |
| Z | 4 | 4 |
| $\rho_{calcd}$, g cm$^{-3}$ | 1.176 | 1.372 |
| μ, mm$^{-1}$ | 2.195 | 15.166 |
| no. of reflections made | 17886 | 30558 |
| no. of unique reflns, $R_{int}$ | 6231, 0.0968 | 5362, 0.0520 |
| no. of reflns with $F^2 > 2\sigma(F^2)$ | 3564 | 5039 |
| transmn coeff range | 0.514–1.000 | 0.754–0.948 |
| R, $R_w$[a] ($F^2 > 2\sigma(F^2)$) | 0.0628, 0.0979 | 0.0265, 0.0704 |
| R, $R_w$[a] (all data) | 0.1367, 0.1203 | 0.0280, 0.0713 |
| S[a] | 0.997 | 1.085 |
| parameters, restraints | 288, 6 | 288, 6 |
| max., min. diff map, e Å$^{-3}$ | 0.605, –0.676 | 0.702, –0.642 |

[a] Conventional $R = \Sigma||F_o| - |F_c||/\Sigma|F_o|$; $R_w = [\Sigma w(F_o^2 - F_c^2)^2/\Sigma w(F_o^2)^2]^{1/2}$; $S = [\Sigma w(F_o^2 - F_c^2)^2/\text{no. data} - \text{no. params})]^{1/2}$ for all data.

**Table S10.** Crystallographic data for **3-Y·$C_6H_6$** and **3-Dy·$C_6H_6$**.

| | **3-Y·$C_6H_6$** | **3-Dy·$C_6H_6$** |
|---|---|---|
| formula | $C_{49}H_{50}AlF_{36}O_4Y$ | $C_{49}H_{50}AlDyF_{36}O_4$ |
| molecular mass, g mol$^{-1}$ | 1502.78 | 1576.37 |
| cryst size, mm | 0.129 × 0.065 × 0.034 | 0.127 × 0.114 × 0.087 |
| cryst syst | orthorhombic | orthorhombic |
| space group | *Pbca* | *Pbca* |
| collection temperature, K | 100(2) | 100(2) |
| a, Å | 19.3388(4) | 19.3225(5) |
| b, Å | 17.6094(3) | 17.6470(5) |
| c, Å | 34.4160(7) | 34.4828(11) |
| α, ° | 90 | 90 |
| β, ° | 90 | 90 |
| γ, ° | 90 | 90 |
| V, Å$^3$ | 11720.2(4) | 11758.1(6) |
| Z | 8 | 8 |
| $\rho_{calcd}$, g cm$^{-3}$ | 1.703 | 1.781 |
| μ, mm$^{-1}$ | 3.010 | 1.443 |
| no. of reflections made | 45045 | 78418 |
| no. of unique reflns, $R_{int}$ | 10722, 0.0749 | 14664, 0.0602 |
| no. of reflns with $F^2 > 2\sigma(F^2)$ | 8029 | 10037 |
| transmn coeff range | 0.863–1.000 | 0.863–1.000 |
| R, $R_w$[a] ($F^2 > 2\sigma(F^2)$) | 0.0753, 0.2072 | 0.0611, 0.1446 |
| R, $R_w$[a] (all data) | 0.0962, 0.2244 | 0.0980, 0.1631 |
| S[a] | 1.036 | 1.029 |
| parameters, restraints | 1213, 3123 | 1204, 3531 |
| max., min. diff map, e Å$^{-3}$ | 1.118, –1.825 | 2.173, –1.517 |

[a] Conventional $R = \Sigma||F_o| - |F_c||/\Sigma|F_o|$; $R_w = [\Sigma w(F_o^2 - F_c^2)^2/\Sigma w(F_o^2)^2]^{1/2}$; $S = [\Sigma w(F_o^2 - F_c^2)^2/\text{no. data} - \text{no. params})]^{1/2}$ for all data.

**Table S11.** Crystallographic data for **4-Y** and **4-Dy**.

| | **4-Y** | **4-Dy** |
|---|---|---|
| formula | $C_{43}H_{44}AlF_{36}O_4Y$ | $C_{43}H_{44}AlDyF_{36}O_4$ |
| molecular mass, g $mol^{-1}$ | 1424.67 | 1498.26 |
| cryst size, mm | 0.253 × 0.172 × 0.094 | 0.384 × 0.196 × 0.166 |
| cryst syst | monoclinic | monoclinic |
| space group | $P2_1/c$ | $P2_1/c$ |
| collection temperature, K | 100(2) | 100(2) |
| a, Å | 19.8930(2) | 19.901(2) |
| b, Å | 15.05390(10) | 15.0732(7) |
| c, Å | 20.2976(2) | 20.318(2) |
| α, ° | 90 | 90 |
| β, ° | 117.4260(10) | 117.337(14) |
| γ, ° | 90 | 90 |
| V, $Å^3$ | 5395.28(9) | 5414.3(11) |
| Z | 4 | 4 |
| $\rho_{calcd}$, g $cm^{-3}$ | 1.754 | 1.838 |
| μ, $mm^{-1}$ | 3.229 | 1.561 |
| no. of reflections made | 67076 | 18340 |
| no. of unique reflns, $R_{int}$ | 9777, 0.0371 | 9819, 0.0626 |
| no. of reflns with $F^2 > 2\sigma(F^2)$ | 9195 | 6668 |
| transmn coeff range | 0.608–1.000 | 0.560–1.000 |
| R, $R_w$[a] ($F^2 > 2\sigma(F^2)$) | 0.0483, 0.1294 | 0.0577, 0.1027 |
| R, $R_w$[a] (all data) | 0.0503, 0.1310 | 0.1008, 0.1231 |
| S[a] | 1.114 | 0.957 |
| parameters, restraints | 1033, 2537 | 1027, 2358 |
| max., min. diff map, e $Å^{-3}$ | 2.193, –1.013 | 1.847, –1.882 |

[a] Conventional $R = \Sigma||F_o| - |F_c||/\Sigma|F_o|$; $R_w = [\Sigma w(F_o^2 - F_c^2)^2/\Sigma w(F_o^2)^2]^{1/2}$; $S = [\Sigma w(F_o^2 - F_c^2)^2$/no. data – no. params)]$^{1/2}$ for all data.

**Table S12.** Crystallographic data for **5%Dy@4-Y** and **5·$C_6H_5F$**.

| | **5%Dy@4-Y** | **5·$C_6H_5F$** |
|---|---|---|
| formula | $C_{43}H_{44}AlDy_{0.05}F_{36}O_4Y_{0.95}$ | $C_{156}H_{97}B_8F_{81}$ |
| molecular mass, g $mol^{-1}$ | 1428.35 | 3596.81 |
| cryst size, mm | 0.125 x 0.1 x 0.051 | 0.144 x 0.129 x 0.081 |
| cryst syst | triclinic | triclinic |
| space group | $P\bar{1}$ | $P\bar{1}$ |
| collection temperature, K | 100(2) | 100(2) |
| a, Å | 11.2267(9) | 10.14100(10) |
| b, Å | 14.9495(10) | 17.56690(10) |
| c, Å | 16.7987(8) | 21.52500(10) |
| α, ° | 82.650(5) | 105.8600(10) |
| β, ° | 86.390(6) | 95.7760(10) |
| γ, ° | 77.441(6) | 92.5560(10) |
| V, $Å^3$ | 2727.5(3) | 3659.46(5) |
| Z | 2 | 1 |
| $\rho_{calcd}$, g $cm^{-3}$ | 1.739 | 1.632 |
| μ, $mm^{-1}$ | 3.485 | 1.496 |
| no. of reflections made | 29148 | 73365 |
| no. of unique reflns, $R_{int}$ | 9756, 0.1422 | 14899, 0.0315 |
| no. of reflns with $F^2 > 2\sigma(F^2)$ | 6141 | 12904 |
| transmn coeff range | 0.888-1.000 | 0.866–1.000 |
| R, $R_w^a$ ($F^2 > 2\sigma(F^2)$) | 0.0852, 0.2249 | 0.0366, 0.0940 |
| R, $R_w^a$ (all data) | 0.1282, 0.2695 | 0.0426, 0.0977 |
| $S^a$ | 0.987 | 1.004 |
| parameters, restraints | 907, 1918 | 1137, 1151 |
| max., min. diff map, e $Å^{-3}$ | 1.246, -1.906 | 0.609, –0.285 |

[a] Conventional $R = \Sigma||F_o| - |F_c||/\Sigma|F_o|$; $R_w = [\Sigma w(F_o^2 - F_c^2)^2/\Sigma w(F_o^2)^2]^{1/2}$; $S = [\Sigma w(F_o^2 - F_c^2)^2$/no. data – no. params)$]^{1/2}$ for all data.

**Table S13.** Crystallographic data for **6-Dy·$C_6H_{14}$**.

| | **6-Dy·$C_6H_{14}$** |
|---|---|
| formula | $C_{76}H_{102}AlDy_2F_{37}O_4$ |
| molecular mass, g $mol^{-1}$ | 2134.55 |
| cryst size, mm | 0.081 x 0.061 x 0.018 |
| cryst syst | monoclinic |
| space group | $P2_1/m$ |
| collection temperature, K | 100(2) |
| a, Å | 10.7706(3) |
| b, Å | 26.2037(8) |
| c, Å | 31.1339(8) |
| α, ° | 90 |
| β, ° | 94.345(2) |
| γ, ° | 90 |
| V, $Å^3$ | 8761.6(4) |
| Z | 4 |
| $\rho_{calcd}$, g $cm^{-3}$ | 1.618 |
| μ, $mm^{-1}$ | 10.234 |
| no. of reflections made | 46401 |
| no. of unique reflns, $R_{int}$ | 17472, 0.0664 |
| no. of reflns with $F^2 > 2\sigma(F^2)$ | 11420 |
| transmn coeff range | 0.593–0.887 |
| R, $R_w$[a] ($F^2 > 2\sigma(F^2)$) | 0.0786, 0.2091 |
| R, $R_w$[a] (all data) | 0.1160, 0.2392 |
| S[a] | 1.089 |
| parameters, restraints | 1663, 2821 |
| max., min. diff map, e $Å^{-3}$ | 2.396, –2.521 |

[a] Conventional $R = \Sigma||F_o| - |F_c||/\Sigma|F_o|$; $R_w = [\Sigma w(F_o^2 - F_c^2)^2/\Sigma w(F_o^2)^2]^{1/2}$; $S = [\Sigma w(F_o^2 - F_c^2)^2/\text{no. data} - \text{no. params})]^{1/2}$ for all data.

## 8. DFT Calculations

DFT calculations were performed on the molecular geometries of **3-Dy** and **4-Dy**, taking the largest disorder component only from the single crystal XRD structures and substituting the Dy atom for Y. Both cation and anion were included for **3-Dy** and **4-Dy**, the benzene co-crystallized solvent was not included for **3-Dy**. Single-point DFT calculations were performed at the PBE0[29,30]-D3[31] level of theory with the cc-pVDZ[32] basis set for the light atoms and the Stuttgart RSC 1997 effective core potential and corresponding valence basis set for Y.[33,34] The default Gaussian 16 Rev C.01[35] integration grids, convergence method, and convergence thresholds (for both SCF and geometry iterations) were used throughout. Mulliken population analysis was used to compute atomic charges. Topological analysis was performed on the DFT electronic structure with Multiwfn[36] to quantify the strength of the Dy-F interactions in both XRD and DFT optimized structures, where the value of the density at the bond critical point, ρ(BCP), is indicative of the strength of the interaction, where a larger density and smaller distance between atoms results in a stronger interaction.

**Table S14.** Results from Mulliken population analysis

| | **3-Y (3-Dy geometry)** | **4-Y (4-Dy geometry)** |
|---|---|---|
| F(1) charge / *e* | –0.096 | –0.069 |
| Average F charge in other $C(CF_3)$ groups (standard deviation) / *e* | –0.117(0.011) | –0.116(0.011) |
| Δ(C(29)–F(1)) charge / *e* | 0.420 | 0.401 |
| C–F bonds in other $C(CF_3)$ groups, Δ(C–F) charge (standard deviation) / *e* | 0.438(0.012) | 0.435(0.012) |
| Δ(Y(1)–F(1)) charge / *e* | 0.418 | 0.359 |

**Table S15.** Results from topological analysis.

| | R(Dy-F) / Å | ρ(BCP) / a.u. |
|---|---|---|
| **3-Y (3-Dy geometry)** | 3.144 | 0.002 |
| **4-Y (4-Dy geometry)** | 2.813 | 0.018 |
| **3-Y (Opt. geometry)** | 2.312 | 0.025 |
| | 2.292 | 0.024 |
| **4-Y (Opt. geometry)** | 2.334 | 0.022 |
| | 2.440 | 0.016 |
| | 2.183 | 0.035 |

## 9. Magnetic Measurements

### *9.1 Temperature- and Field-Swept Magnetic Measurements*

Magnetic measurements were performed using a Quantum Design MPMS3 superconducting quantum interference device (SQUID) magnetometer. All samples were crushed with a mortar and pestle under an inert atmosphere, and then loaded into a borosilicate glass NMR tube along with eicosane, which was then evacuated and flame-sealed to give an ampoule with a length of *ca.* 3 cm. The eicosane was melted by heating the tube gently with a low-power heat gun to immobilize the crystallites. The ampoule was then mounted in the center of a drinking straw using friction by wrapping it with Kapton tape, and the straw was then fixed to the end of the sample rod. Samples of "[{Dy($Cp^{ttt}$)(Cp*)}{Al[OC($CF_3$)$_3$]$_4$}]" (19.6 mg), **3-Dy·$C_6H_6$** (18.9 mg), **4-Dy** (21.3 mg) and **5%Dy@4-Y** (33.3 mg) were prepared with 14.7 mg, 13.1 mg, 14.0 mg and 19.2 mg of eicosane respectively. We attempted a solution measurement of **4-Dy** (27.9 mg) in benzene (50 μL), which was biphasic, forming a yellow oil layer and supernatant colorless solution; other solvents could not be used as they coordinate to Dy (e.g. halobenzenes) or cause decomposition (e.g. $CH_2Cl_2$). The measurements were corrected for the diamagnetism of the straw, borosilicate tube and eicosane using calibrated blanks, for the shape of the sample using Quantum Design Geometry Simulator, and for the intrinsic diamagnetism of the sample estimated as the molecular weight (g $mol^{-1}$) multiplied by $-0.5 \times 10^{-6}$ $cm^3$ K $mol^{-1}$. In the case of **4-Dy** in benzene the diamagnetic contribution of benzene was estimated with Pascal's constants.[37]

Dc magnetic measurements (susceptibility, ZFC/FC and hysteresis) were performed in dc scan mode with a scan length of 40 mm and a scan time of 6 s, unless specified otherwise. Susceptibility measurements were measured under 0.1 T field on cooling. For **3-Dy·$C_6H_6$**, **4-Dy** and "[{Dy($Cp^{ttt}$)(Cp*)}{Al[OC($CF_3$)$_3$]$_4$}]" measurements were performed in settle mode, at 5 K $min^{-1}$ from 300–100 K and 1 K $min^{-1}$ from 100–1.8 K. For **4-Dy** in benzene,

measurements were performed from 100–2 K in continuous sweep mode with a sweep rate of –0.5 K $min^{-1}$, measuring every 1 K. For **5%Dy@4-Y**, measurements were performed from 100–2 K in continuous sweep mode with a sweep rate of –0.5 K $min^{-1}$, measuring continuously in VSM mode with a vibration amplitude of 5 mm and averaging time of 2 s.

Zero-field cooled and field-cooled measurements were performed on **3-Dy·$C_6H_6$**, **4-Dy** and "[{Dy($Cp^{ttt}$)(Cp*)}{Al[OC($CF_3$)$_3$]$_4$}]" under 0.1 T field in continuous temperature sweep mode with a constant sweep rate of 0.5 K $min^{-1}$. The zero-field cooled sample was prepared by holding the sample at 100 K for 10 min, cooling at 5 K $min^{-1}$ to 20 K, holding for 5 min, cooling to 2 K at 0.5 K $min^{-1}$ and holding for 2 hours before switching on the field. The field-cooled sample measured on warming was prepared by stabilizing in 0.1 T field at 2 K for 2 h, after approaching from above at 0.5 K $min^{-1}$.

All hysteresis measurements were performed in continuous sweep mode with a sweep rate of 22 Oe $s^{-1}$ across the entire field range. Hysteresis measurements were performed between ± 7 T at temperatures of 2–12 K (**3-Dy·$C_6H_6$** and **4-Dy**), 2–32 K ("[{Dy($Cp^{ttt}$)(Cp*)}{Al[OC($CF_3$)$_3$]$_4$}]") or 2–50 K (**4-Dy** in benzene) on a sample that had been magnetized at 7 T. Above these temperatures until closing of the hysteresis loop, hysteresis measurements were performed between ± 5 T on a sample that had been magnetized at 5 T. All hysteresis measurements on **5%Dy@4-Y** were performed between ± 5 T; these are noisy because of the weak signal of the diluted sample. Hysteresis measurements on **4-Dy** in benzene were performed in VSM mode with 1 mm vibration amplitude and 0.5 s averaging time, we display the lower temperature datasets (2–30 K) with adjacent points averaged in 10 point windows.

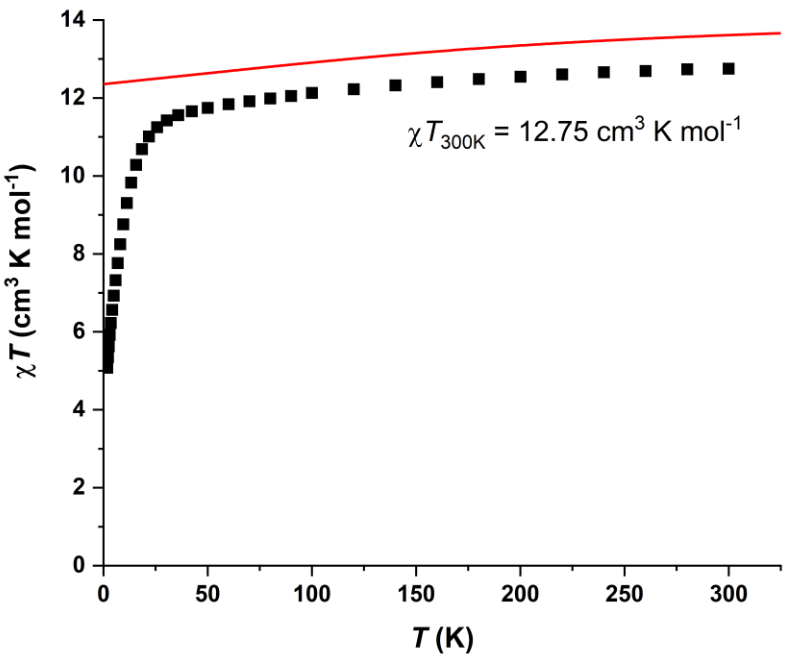


**Figure S73.** Temperature dependence of the molar magnetic susceptibility χT product for powdered **3-Dy·$C_6H_6$** measured under a 0.1 T applied magnetic field (black squares) and predicted values from CASSCF (red line).

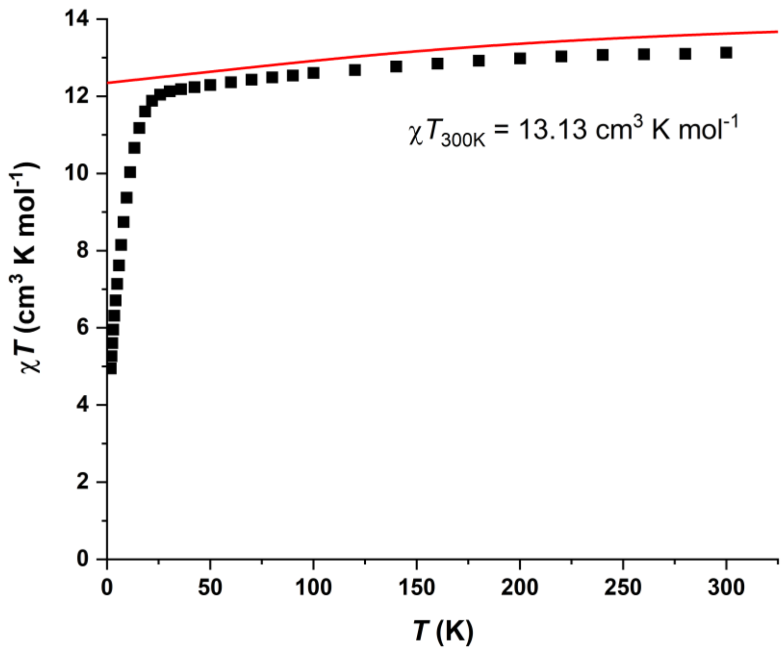


**Figure S74.** Temperature dependence of the molar magnetic susceptibility χT product for powdered **4-Dy** measured under a 0.1 T applied magnetic field (black squares) and predicted values from CASSCF (red line).

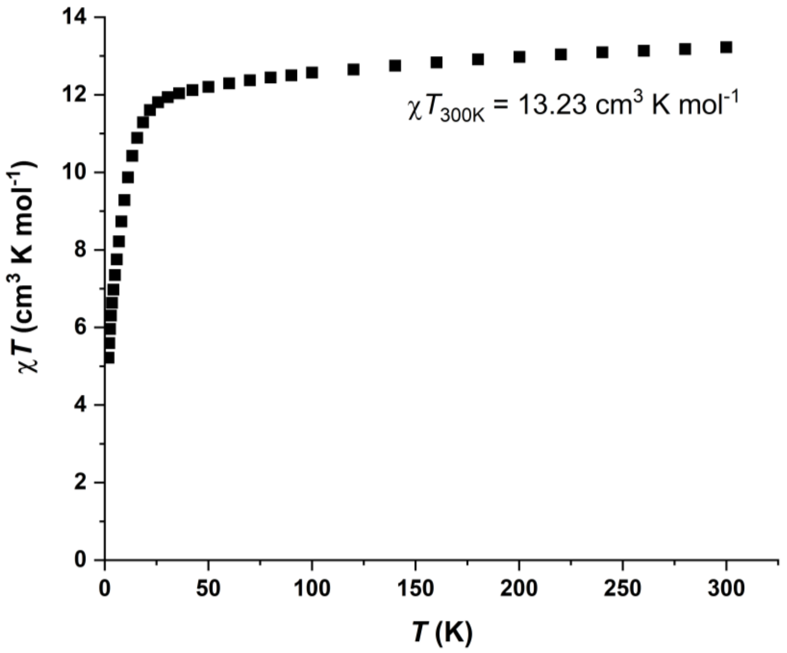


**Figure S75.** Temperature dependence of the molar magnetic susceptibility $\chi T$ product for powdered "[{Dy(Cp$^{ttt}$)(Cp*)}{Al[OC($CF_3$)$_3$]$_4$}]" measured under a 0.1 T applied magnetic field.

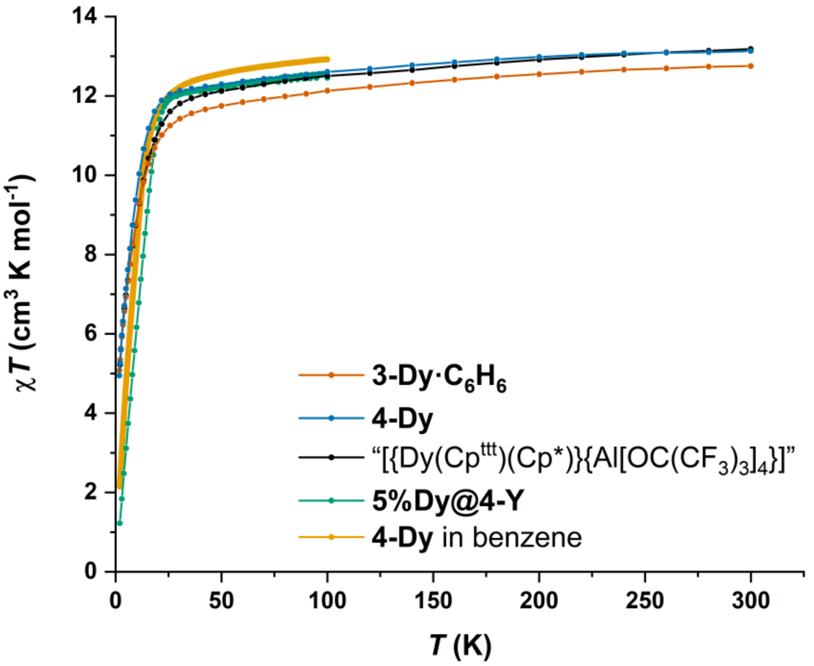


**Figure S76.** Temperature dependence of the molar magnetic susceptibility χT products of **3-Dy·$C_6H_6$**, **4-Dy**, "[{Dy(Cp$^{ttt}$)(Cp*)}{Al[OC($CF_3$)$_3$]$_4$}]", **5%Dy@4-Y** and **4-Dy** in benzene measured under a 0.1 T DC field. Lines are guides for the eye.

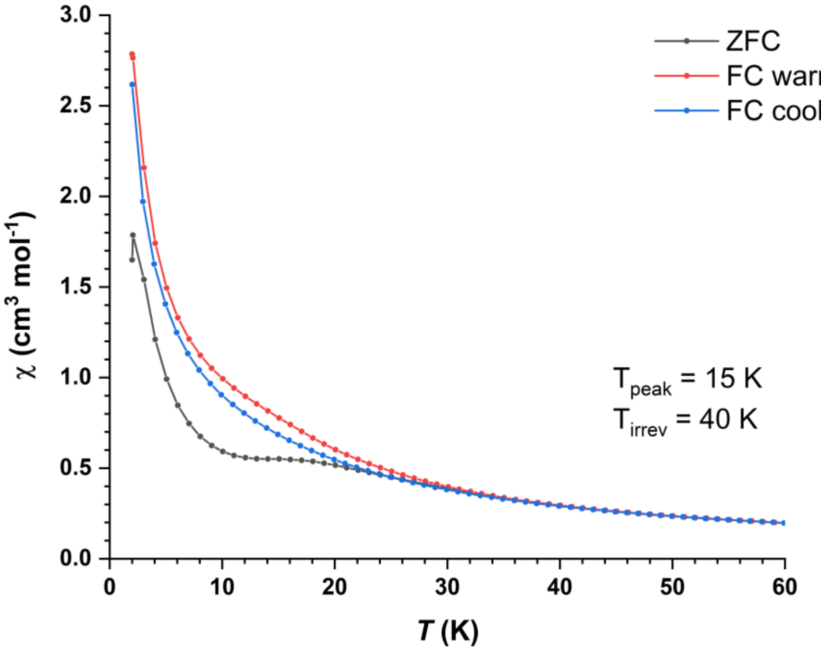


**Figure S77.** Magnetic susceptibility (χ) vs. temperature (K) for **3-Dy·C$_6$H$_6$** measured on warming after cooling in zero field (ZFC, grey) and measured on cooling in field (FC cool, blue) and warming in field (FC warm, red).

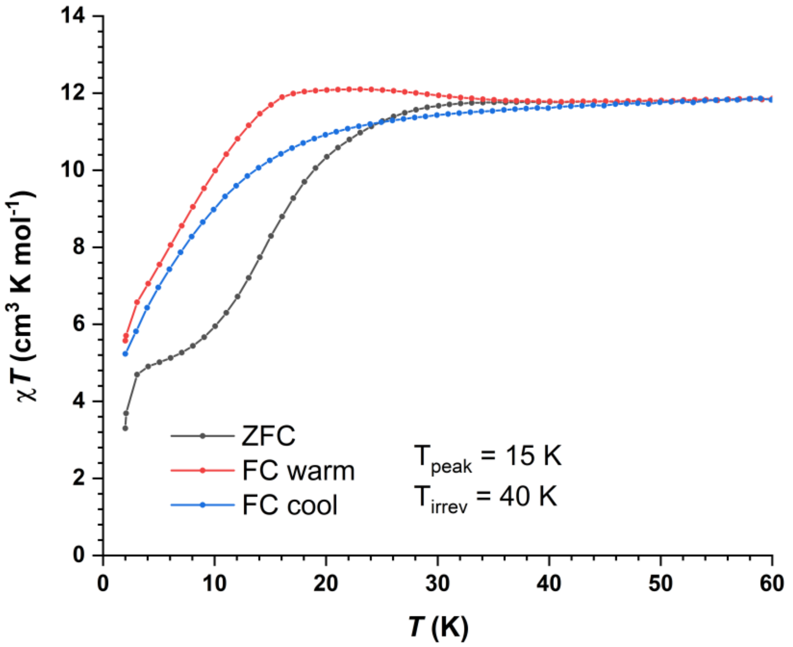


**Figure S78.** Magnetic susceptibility temperature product (χT) vs. temperature (K) for **3-Dy·C$_6$H$_6$** measured on warming after cooling in zero field (ZFC, grey) and measured on cooling in field (FC cool, blue) and warming in field (FC warm, red).

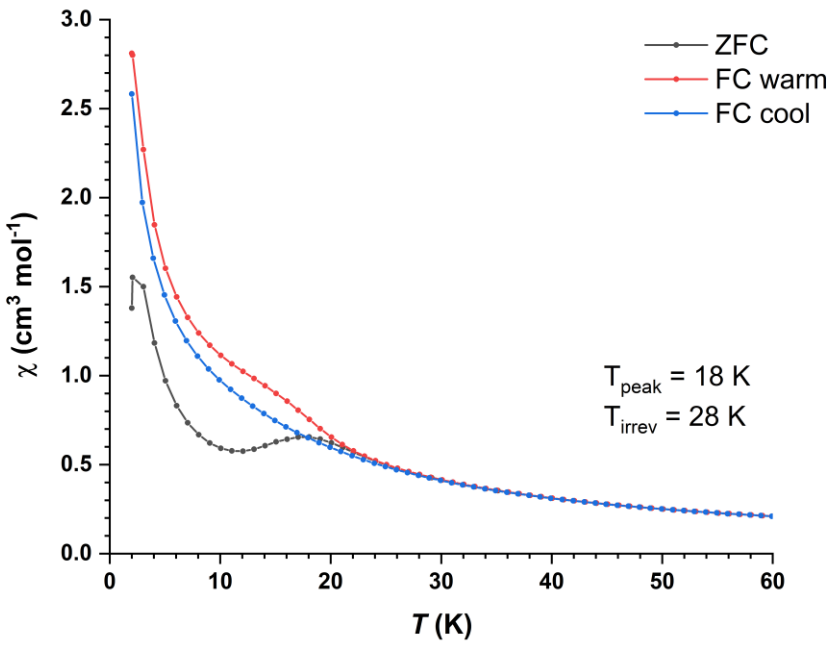


**Figure S79.** Magnetic susceptibility (χ) vs. temperature (K) for **4-Dy** measured on warming after cooling in zero field (ZFC, grey) and measured on cooling in field (FC cool, blue) and warming in field (FC warm, red).

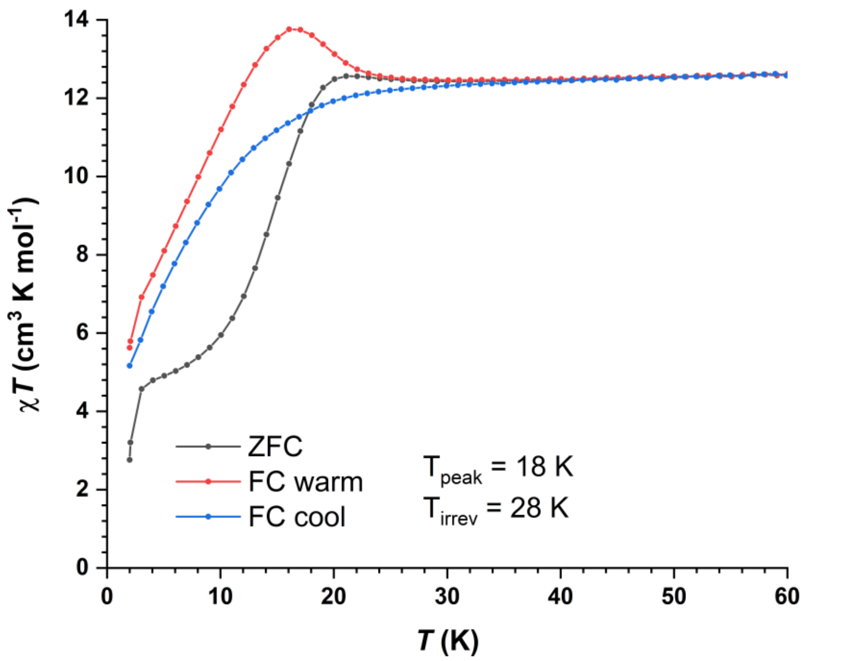


**Figure S80.** Magnetic susceptibility temperature product (χT) vs. temperature (K) for **4-Dy** measured on warming after cooling in zero field (ZFC, grey) and measured on cooling in field (FC cool, blue) and warming in field (FC warm, red).

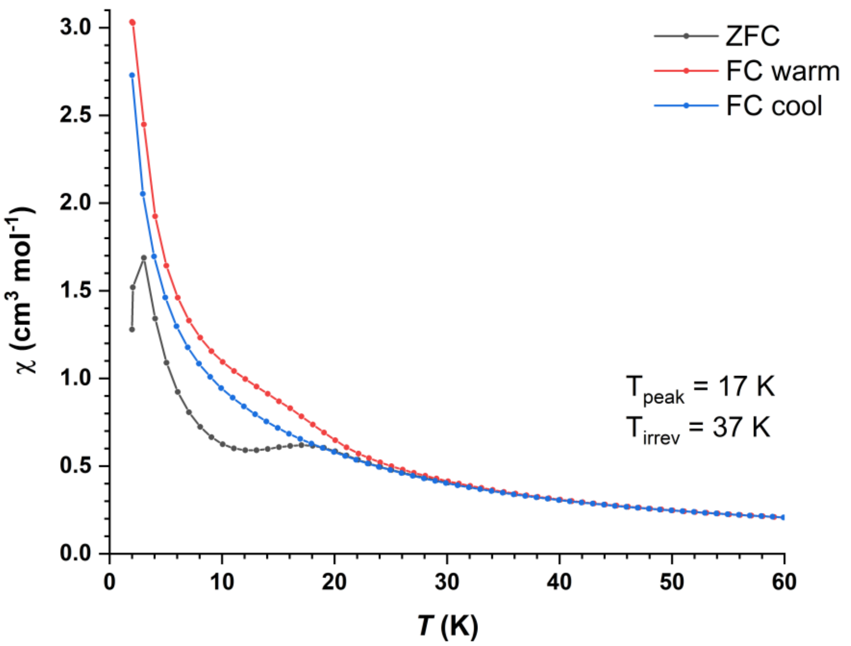


**Figure S81.** Magnetic susceptibility (χ) vs. temperature (K) for "[{Dy(Cp$^{ttt}$)(Cp*)}{Al[OC(CF$_3$)$_3$]$_4$}]" measured on warming after cooling in zero field (ZFC, grey) and measured on cooling in field (FC cool, blue) and warming in field (FC warm, red).

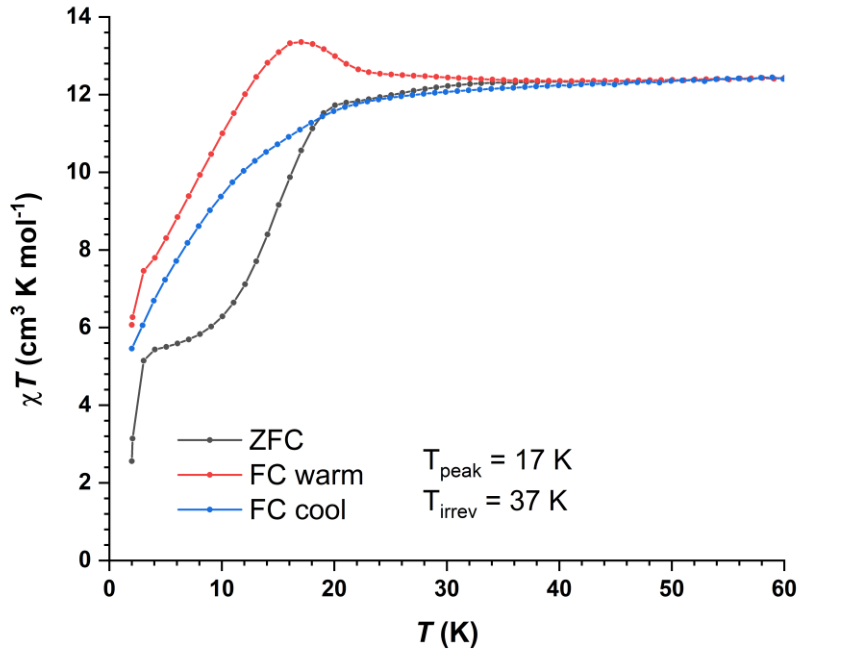


**Figure S82.** Magnetic susceptibility temperature product (χT) vs. temperature (K) for "[{Dy(Cp$^{ttt}$)(Cp*)}{Al[OC(CF$_3$)$_3$]$_4$}]" measured on warming after cooling in zero field (ZFC, grey) and measured on cooling in field (FC cool, blue) and warming in field (FC warm, red).

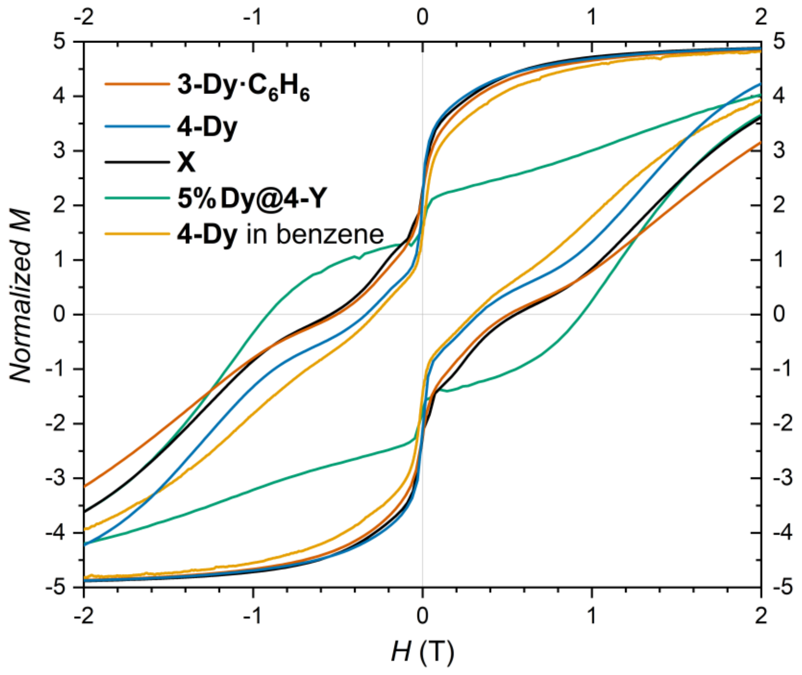


**Figure S83.** Hysteresis loops of "[{Dy(Cp$^{ttt}$)(Cp*)}{Al[OC($CF_3$)$_3$]$_4$}]" (**X**) at 2 K and **3-Dy·$C_6H_6$**, **4-Dy**, **5%Dy@4-Y** and **4-Dy** in benzene at 1.8 K, zoomed in between –2 and 2 T and normalized so value at 7 T (5 T for **5%Dy@4-Y**) is 5.00 N$\mu_B$.

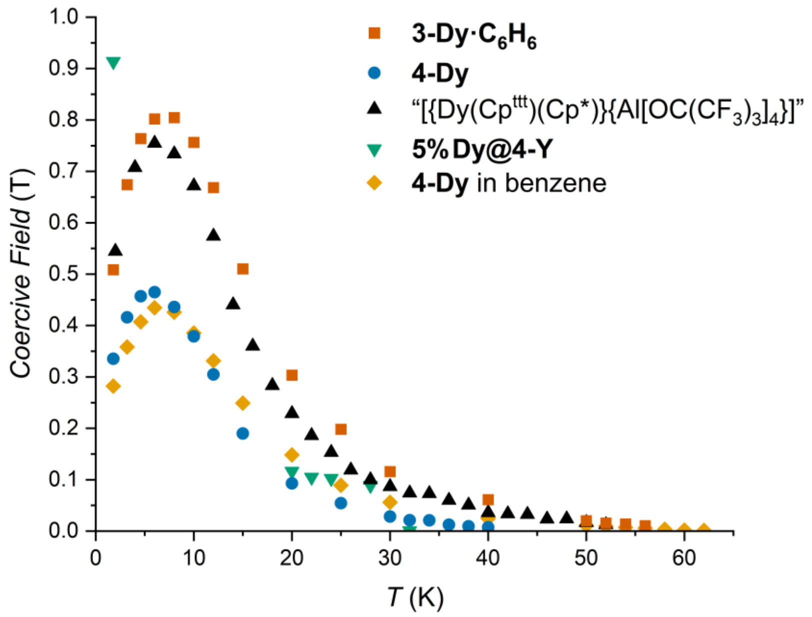


**Figure S84.** Temperature dependence of coercive fields, from interpolation between points on sweep from positive to negative field.

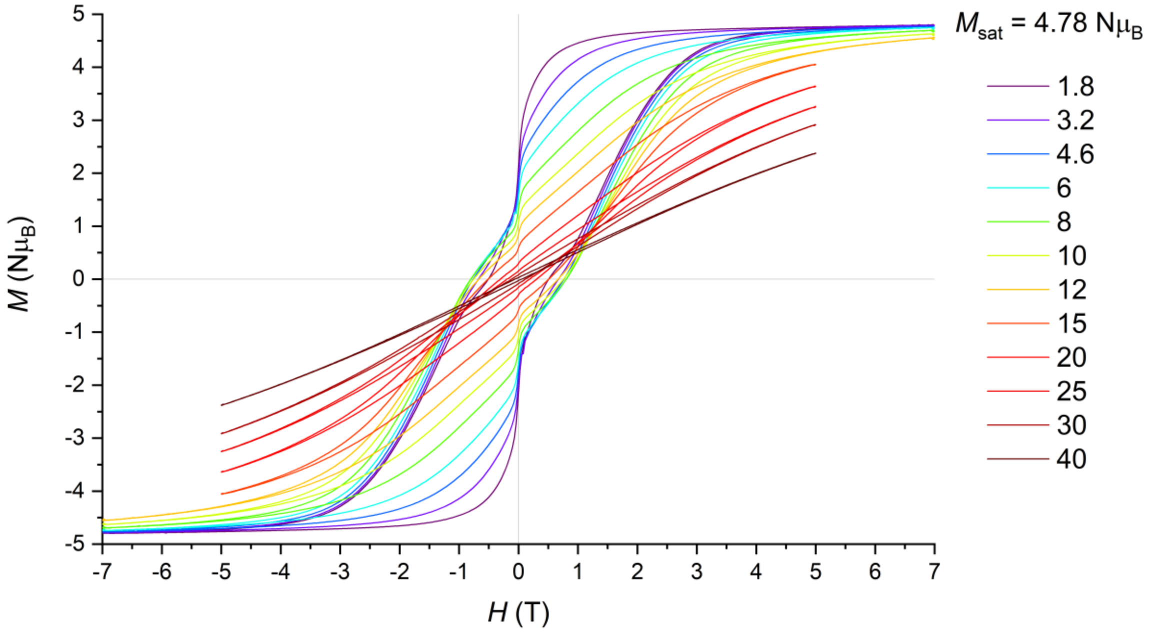


**Figure S85.** Hysteresis loops of **3-Dy·$C_6H_6$** from 1.8 to 40 K and –7 T to +7 T or –5 T to +5 T. Sweep rate is 22 Oe $s^{-1}$.

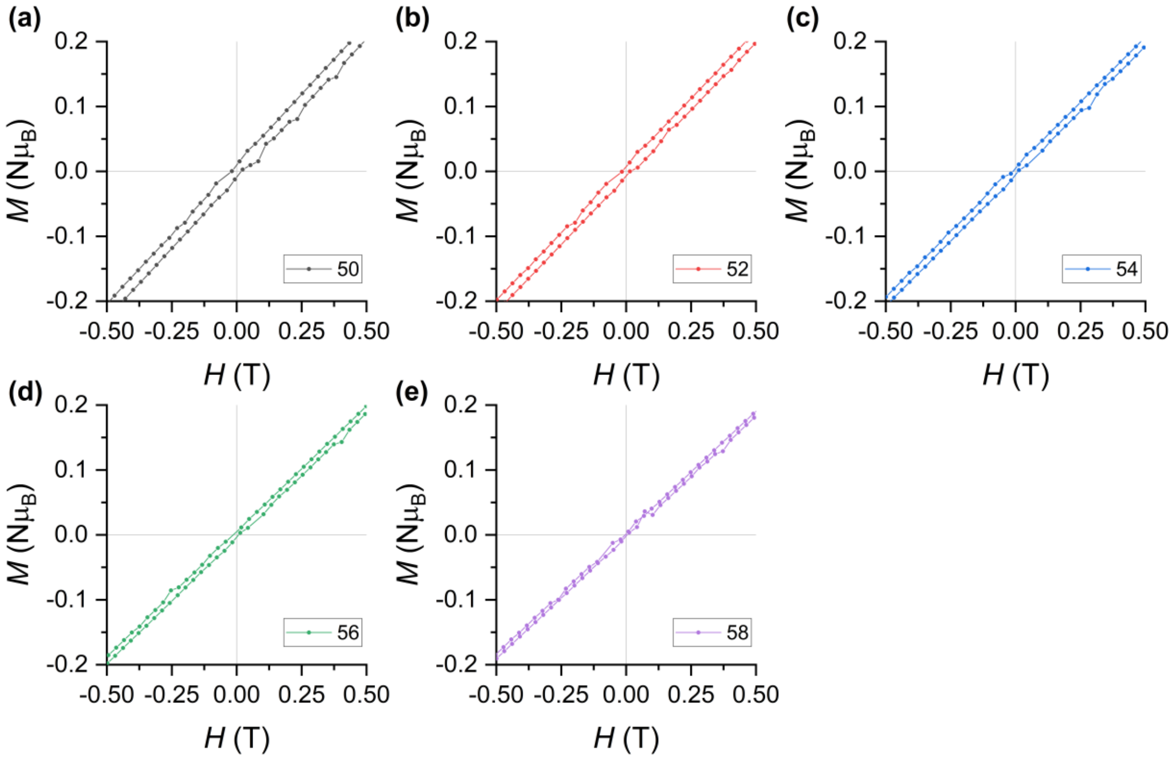


**Figure S86.** Hysteresis loops of **3-Dy·$C_6H_6$** at (a) 50 K, (b) 52 K, (c) 54 K, (d) 56 K and (e) 58 K between –5 T and +5 T, zoomed in between –0.5 and +0.5 T. Sweep rate is 22 Oe $s^{-1}$. Hysteresis is considered open until $T_H$ = 52 K.

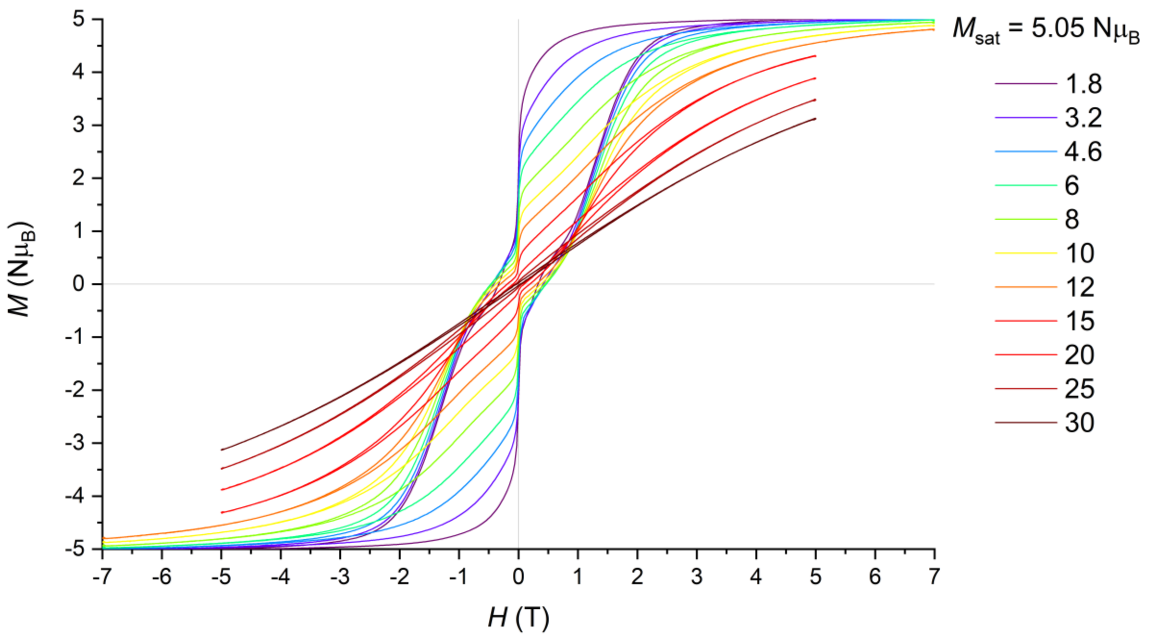


**Figure S87.** Hysteresis loops of **4-Dy** from 1.8 to 30 K and –7 T to +7 T or –5 T to +5 T. Sweep rate is 22 Oe s$^{-1}$.

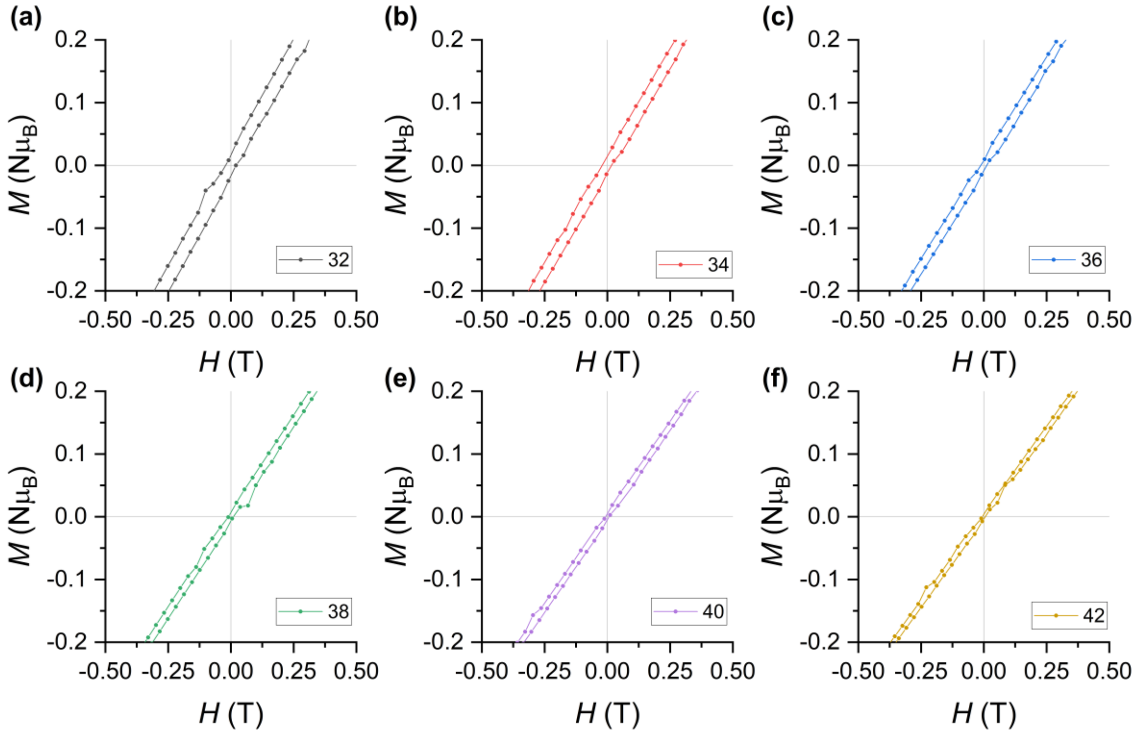


**Figure S88.** Hysteresis loops of **4-Dy** at (a) 32, (b) 34 K, (c) 36 K, (d) 38 K, (e) 40 K and (f) 42 K between –5 T and +5 T, zoomed in between –0.5 and +0.5 T. Sweep rate is 22 Oe s$^{-1}$. Hysteresis is considered open until $T_H$ = 36 K.

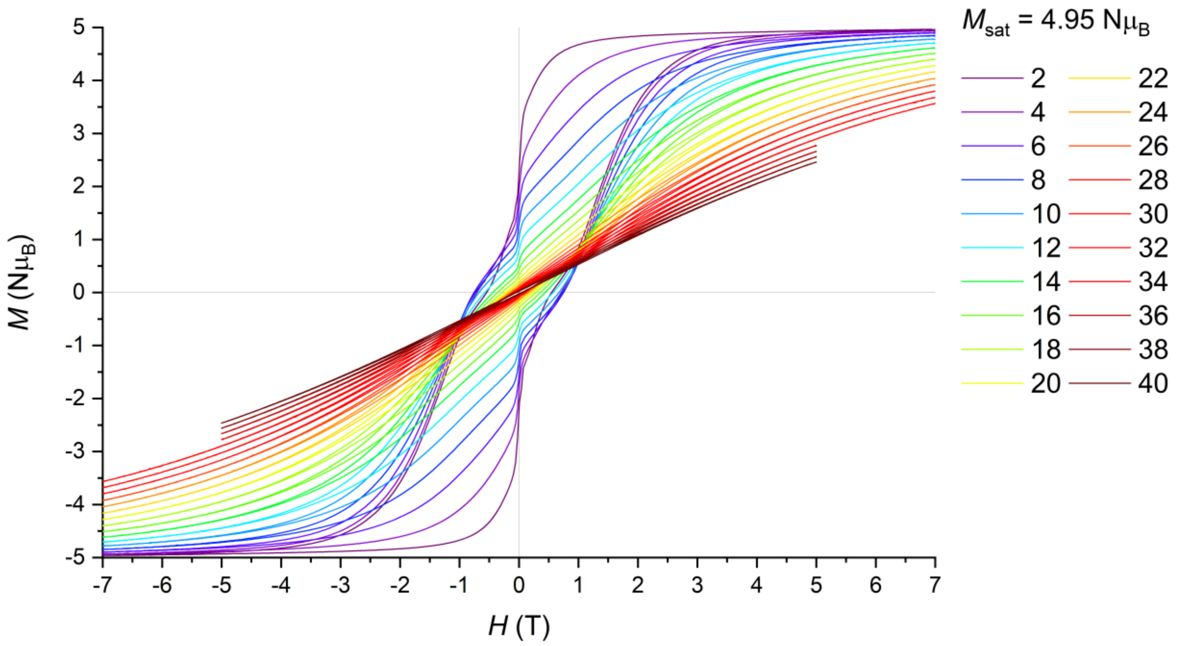


**Figure S89.** Hysteresis loops of “[{Dy(Cp$^{ttt}$)(Cp*)}{Al[OC($CF_3$)$_3$]$_4$}]” from 2 to 40 K and –7 T to +7 T or –5 T to +5 T. Sweep rate is 22 Oe s$^{-1}$.

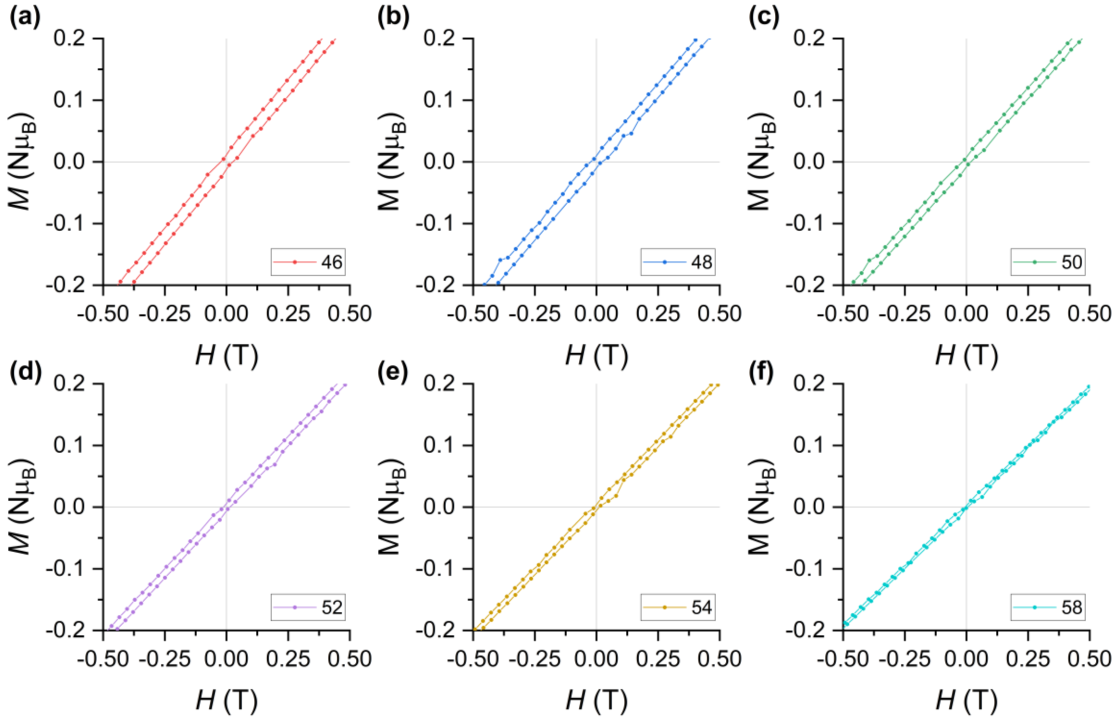


**Figure S90.** Hysteresis loops of “[{Dy(Cp$^{ttt}$)(Cp*)}{Al[OC($CF_3$)$_3$]$_4$}]” at (a) 46 K, (b) 48 K, (c) 50 K, (d) 52 K, (e) 54 K and (f) 58 K between –5 T and +5 T, zoomed in between –0.5 and +0.5 T. Sweep rate is 22 Oe s$^{-1}$. Hysteresis is considered open until $T_H$ = 50 K.

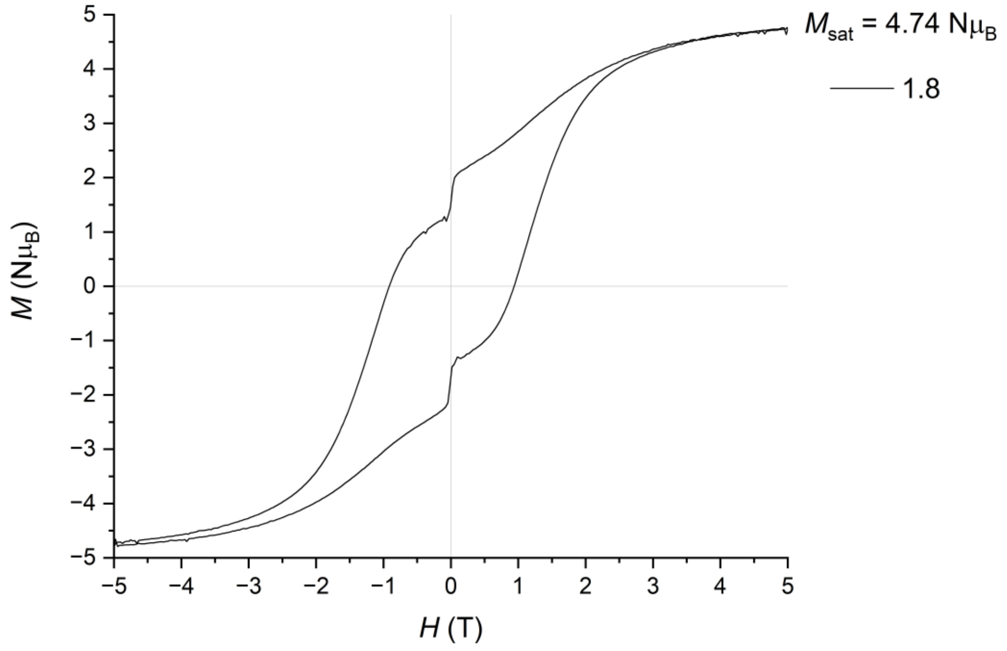


**Figure S91.** Hysteresis loops of **5%Dy@4-Y** at 1.8 K from –5 T to +5 T. Sweep rate is 22 Oe $s^{-1}$. Calculated per mole of Dy.

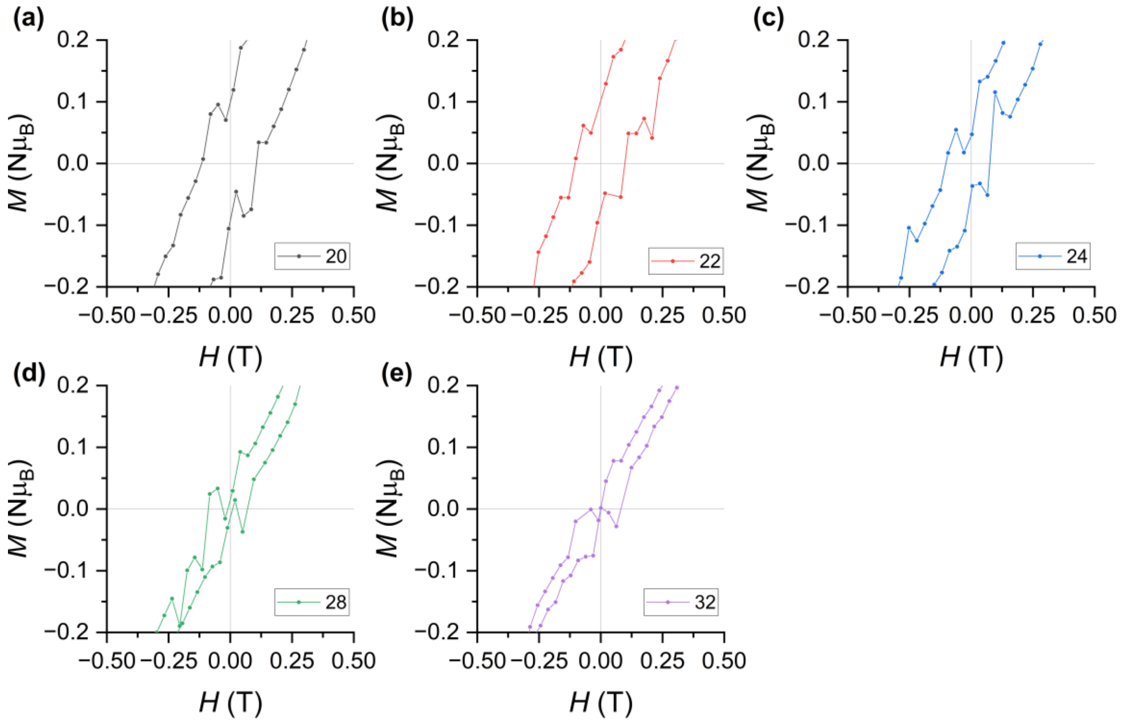


**Figure S92.** Hysteresis loops of **5%Dy@4-Y** at (a) 20 K, (b) 22 K, (c) 24 K, (d) 28 K and (e) 32 K zoomed in between –0.5 and +0.5 T. Sweep rate is 22 Oe $s^{-1}$. Hysteresis is considered open until $T_H$ = 28 K. Calculated per mole of Dy.

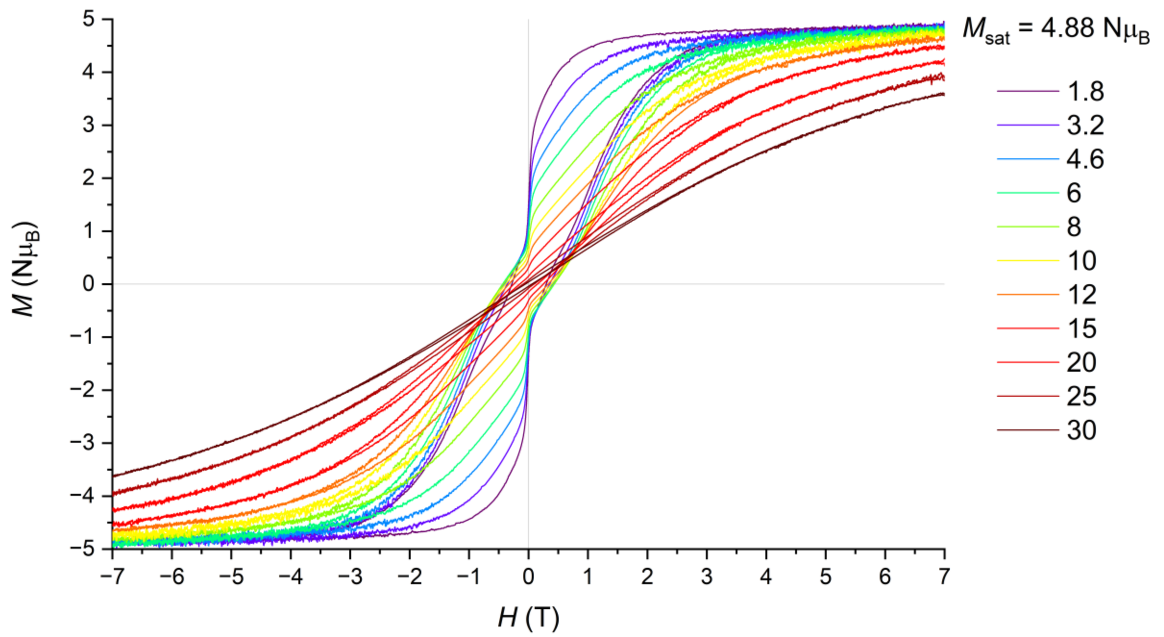


**Figure S93.** Hysteresis loops of **4-Dy** in benzene from 2 to 30 K and –7 T to +7 T. Sweep rate is 22 Oe s$^{-1}$. Data has been smoothed using adjacent averaging over 10 point windows.

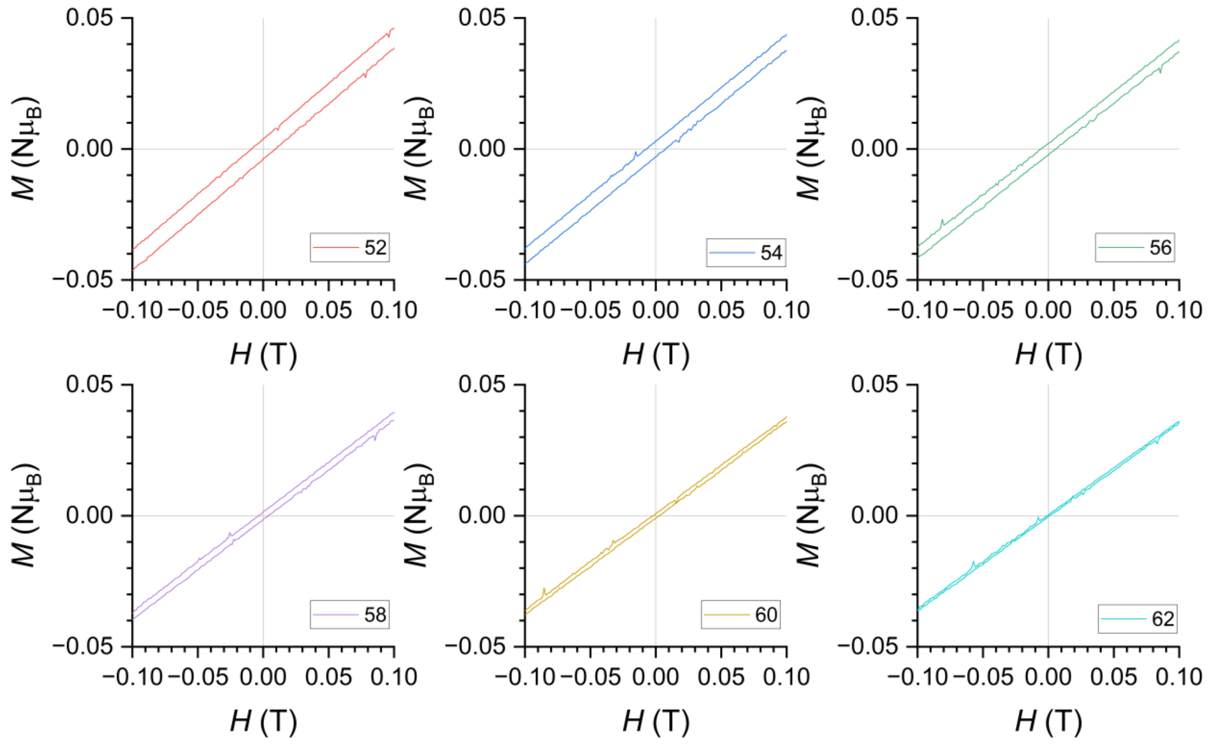


**Figure S94.** Hysteresis loops of **4-Dy** in benzene at (a) 52 K, (b) 54 K, (c) 56 K, (d) 58 K, (e) 60 K and (f) 62 K between –5 T and +5 T, zoomed in between –0.1 and +0.1 T, measured in VSM mode. Sweep rate is 22 Oe s$^{-1}$. Hysteresis is considered open until $T_H$ = 60 K.

*9.2 Ac Magnetic Measurements*

Alternating current (ac) susceptibility measurements were performed on a Quantum Design MPMS3 on samples as described in §9.1. Measurements were recorded for 8 frequencies per decade between 0.1–1000 Hz with a 5 Oe oscillating field. Due to instrumental limitations, 750 and 1000 Hz frequencies were measured with a 2 Oe oscillating field. Averages were generally performed for 2 s or for 10 cycles, whichever was longer; for the weaker **5%Dy@4-Y** sample some measurements were performed with 10 s or 50 cycles, and for **4-Dy** in benzene, measurements were performed with 5 s or 25 cycles. Measurements were performed for 2–105 K for **3-Dy·$C_6H_6$**, 2–108 K for **4-Dy**, 63–108 K for "[{Dy($Cp^{ttt}$)(Cp*)}{Al[OC($CF_3$)$_3$]$_4$}]", 66–102 K for **5%Dy@4-Y** and 63–105 K for **4-Dy** in benzene.

Two relaxation channels were observed for **3-Dy·$C_6H_6$** and **4-Dy**, associated with vastly different temperatures and relaxation timescales (Figures S95–S102). The minor relaxation channel appeared as a broad peak between 10–45 K for **3-Dy·$C_6H_6$** and 10–35 K for **4-Dy** and as a shoulder at 2 and 5 K. Ac data for **4-Dy** in benzene has a second and potentially third high frequency shoulder (Figures S104 and S105). For all samples, peaks in the out-of-phase susceptibility were observed for the major relaxation channel (majority) at 66–102 K. Data for 60–72 K (**3-Dy·$C_6H_6$**, **4-Dy**, "[{Dy($Cp^{ttt}$)(Cp*)}{Al[OC($CF_3$)$_3$]$_4$}]") were trimmed to show only the major relaxation channel. Data for **5%Dy@4-Y** was noisy; obviously incorrect points were removed. Data from 60 K and above for the solid samples were fit to the Generalised Debye (GD) model (Equation S1) in CC-FIT2 (version 5.2.1) to extract relaxation rates and distributions (Figures S106–S113, Tables S16–S19): [38,39]

$$\chi(\omega) = \chi_S + (\chi_T - \chi_S)\frac{1}{1 + \left(i\omega\tau_{\text{debye}}\right)^{1-\alpha}}$$

$$\chi'(\omega) = \chi_S + (\chi_T - \chi_S)\frac{1 + (\omega\tau_{\text{debye}})^{1-\alpha}\sin(\pi\alpha/2)}{1 + 2(\omega\tau_{\text{debye}})^{1-\alpha}\sin(\pi\alpha/2) + (\omega\tau_{\text{debye}})^{2-2\alpha}}$$

$$\chi''(\omega) = (\chi_T - \chi_S)\frac{(\omega\tau_{\text{debye}})^{1-\alpha}\cos(\pi\alpha/2)}{1 + 2(\omega\tau_{\text{debye}})^{1-\alpha}\sin(\pi\alpha/2) + (\omega\tau_{\text{debye}})^{2-2\alpha}}$$

Equation S1

At higher temperatures (78–105 K) for **4-Dy** in benzene we were unable to satisfactorily model the high frequency shoulder and so the high frequency tail has been trimmed and data fit to the generalized Debye model in CC-FIT2 (Figure S114 and S115, Table S20).[38,39] At lower temperatures (63–76.5 K), trimming the highest frequencies allowed the main peak and first high frequency shoulder to be fit using the double generalized Debye model (DGD, Equation S2) in CC-FIT2 to extract relaxation rates and distributions (Figures S116 and S117, Table S21).[38,39]

$$\chi(\omega) = \chi_{\text{Total}} + \frac{\Delta\chi_1}{1 + (i\omega\tau_{\text{debye,1}})^{1-\alpha_1}} + \frac{\Delta\chi_2}{1 + (i\omega\tau_{\text{debye,2}})^{1-\alpha_2}}$$

$$\chi'(\omega) = \chi_{\text{Total}} + \Delta\chi_1\frac{1 + (\omega\tau_{\text{debye,1}})^{1-\alpha_1}\sin(\pi\alpha_1/2)}{1 + 2(\omega\tau_{\text{debye,1}})^{1-\alpha_1}\sin(\pi\alpha_1/2) + (\omega\tau_{\text{debye,1}})^{2-2\alpha_1}}$$

$$+ \Delta\chi_2\frac{1 + (\omega\tau_{\text{debye,2}})^{1-\alpha_2}\sin(\pi\alpha_2/2)}{1 + 2(\omega\tau_{\text{debye,2}})^{1-\alpha_2}\sin(\pi\alpha_2/2) + (\omega\tau_{\text{debye,2}})^{2-2\alpha_2}}$$

$$\chi''(\omega) = \Delta\chi_1\frac{1 + (\omega\tau_{\text{debye,1}})^{1-\alpha_1}\cos(\pi\alpha_1/2)}{1 + 2(\omega\tau_{\text{debye,1}})^{1-\alpha_1}\sin(\pi\alpha_1/2) + (\omega\tau_{\text{debye,1}})^{2-2\alpha_1}}$$

$$+ \Delta\chi_2\frac{1 + (\omega\tau_{\text{debye,2}})^{1-\alpha_2}\cos(\pi\alpha_2/2)}{1 + 2(\omega\tau_{\text{debye,2}})^{1-\alpha_2}\sin(\pi\alpha_2/2) + (\omega\tau_{\text{debye,2}})^{2-2\alpha_2}}$$

Equation S2

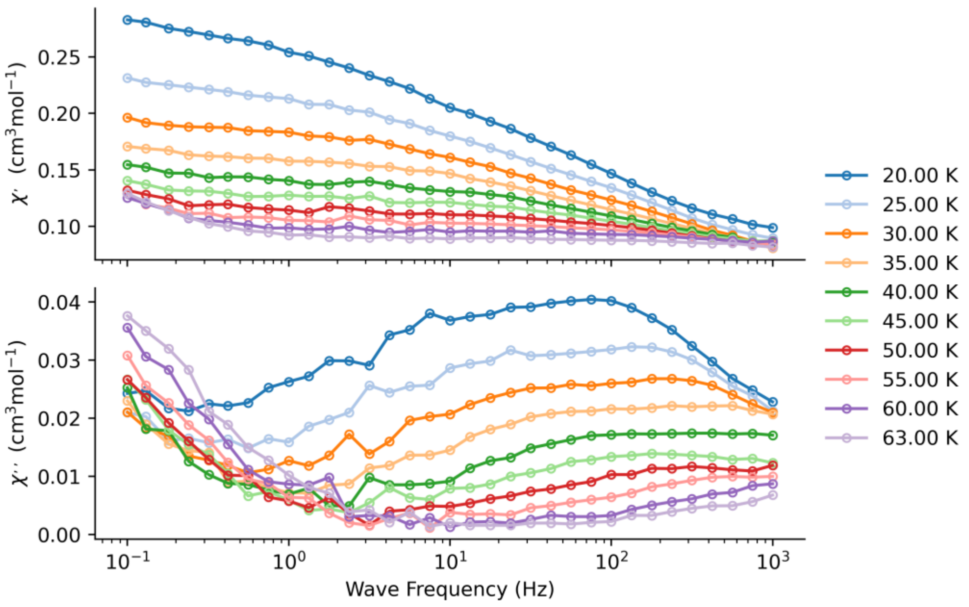


**Figure S95.** Ac susceptibility data on **3-Dy·$C_6H_6$** for 20–63 K showing the appearance of a second minor relaxation channel at low temperature. Lines connect datapoints.

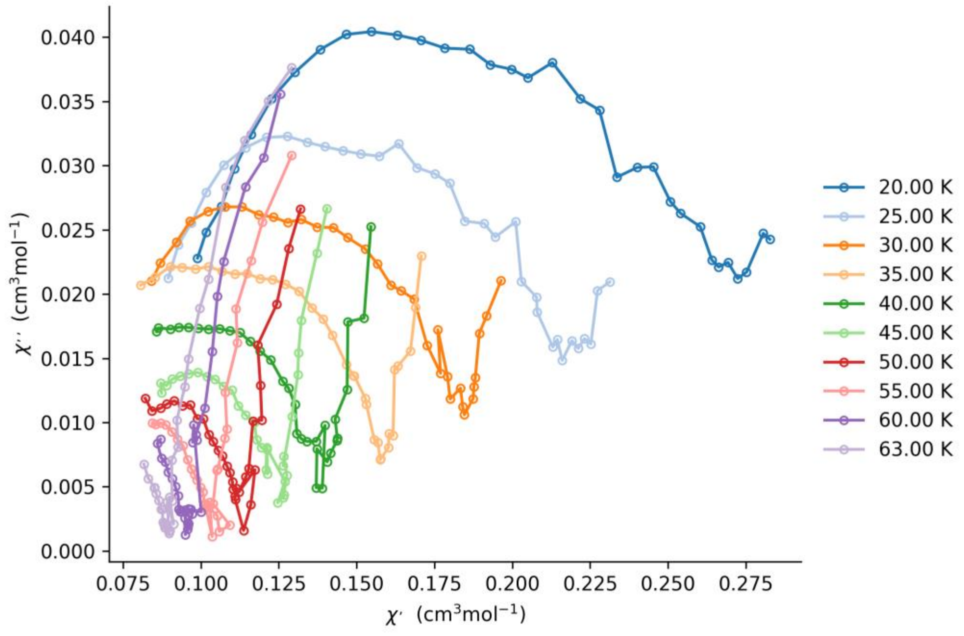


**Figure S96.** Cole-Cole plot of ac susceptibility data on **3-Dy·$C_6H_6$** for 20–63 K showing the appearance of a minor relaxation channel at low temperature. Lines connect datapoints.

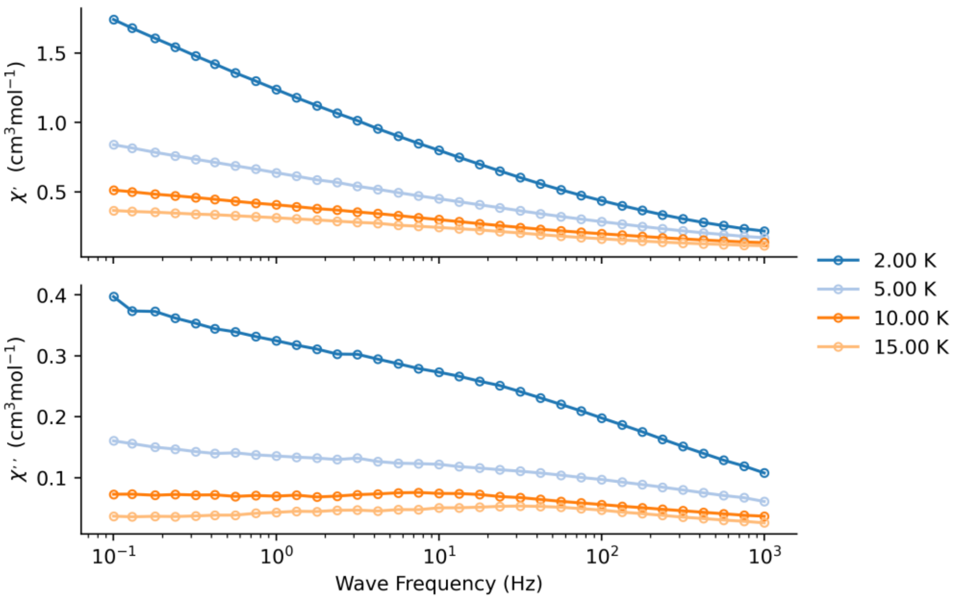


**Figure S97.** Ac susceptibility data on **3-Dy·$C_6H_6$** for 2–15 K showing the minor relaxation channel appearing as a shoulder. Lines connect datapoints.

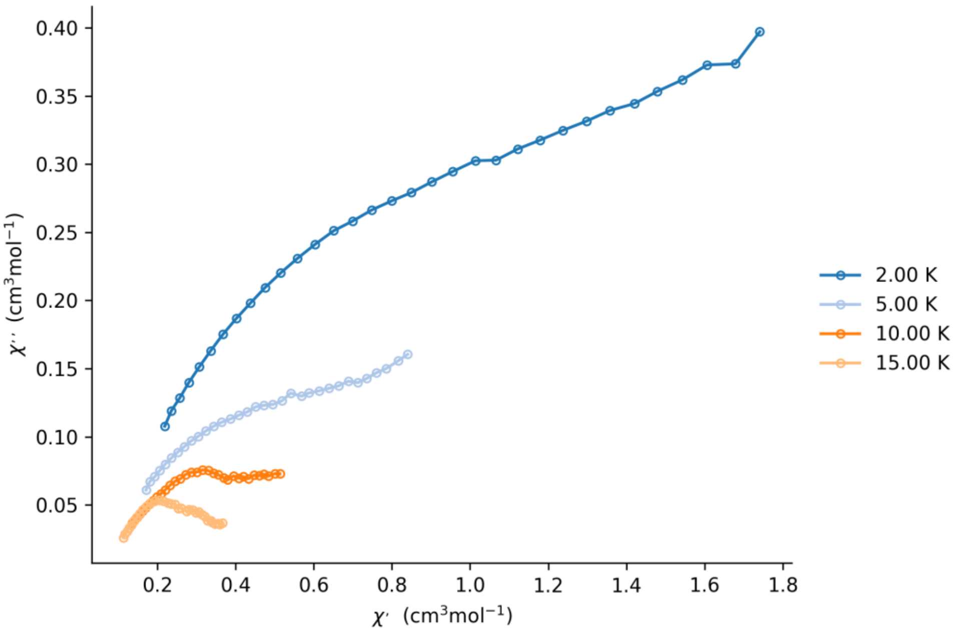


**Figure S98.** Cole-Cole plot of ac susceptibility data on **3-Dy·$C_6H_6$** for 2–15 K showing the minor relaxation channel appearing as a shoulder. Lines connect datapoints.

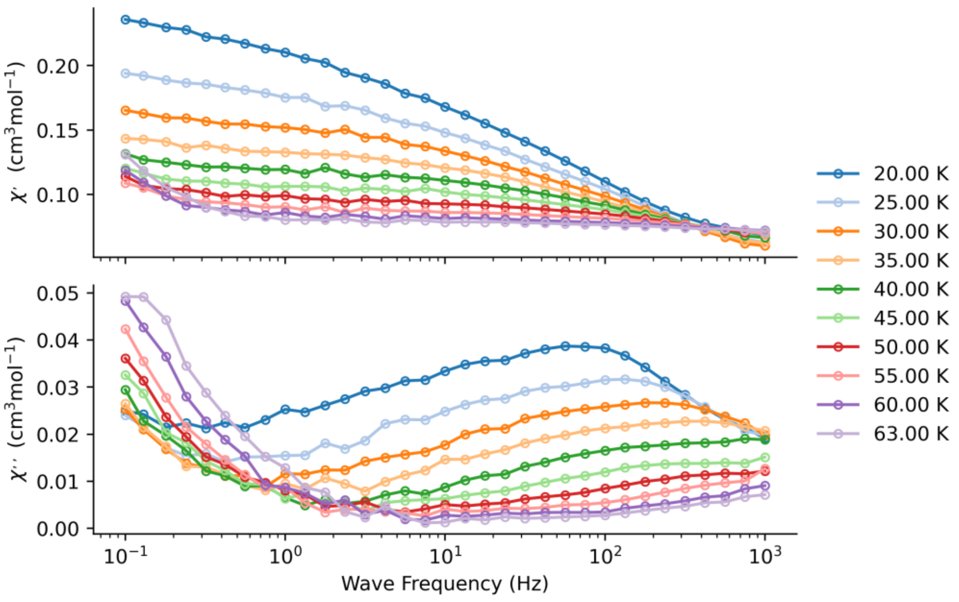


**Figure S99.** Ac susceptibility data on **4-Dy** for 20–63 K showing the appearance of a minor relaxation channel at low temperature. Lines connect datapoints.

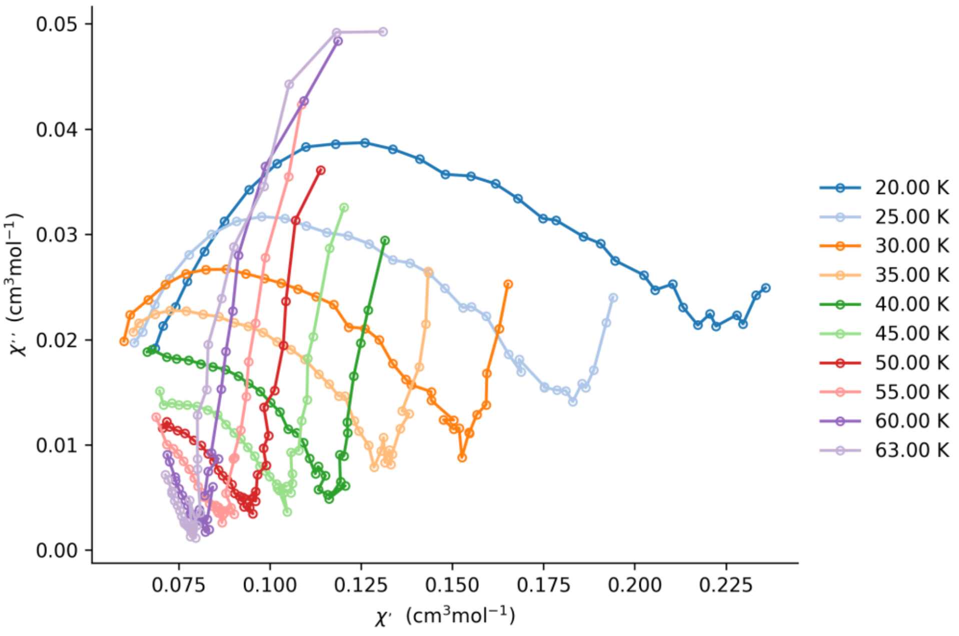


**Figure S100.** Cole-Cole plot of ac susceptibility data on **4-Dy** for 20–63 K showing the appearance of a minor relaxation channel at low temperature. Lines connect datapoints.

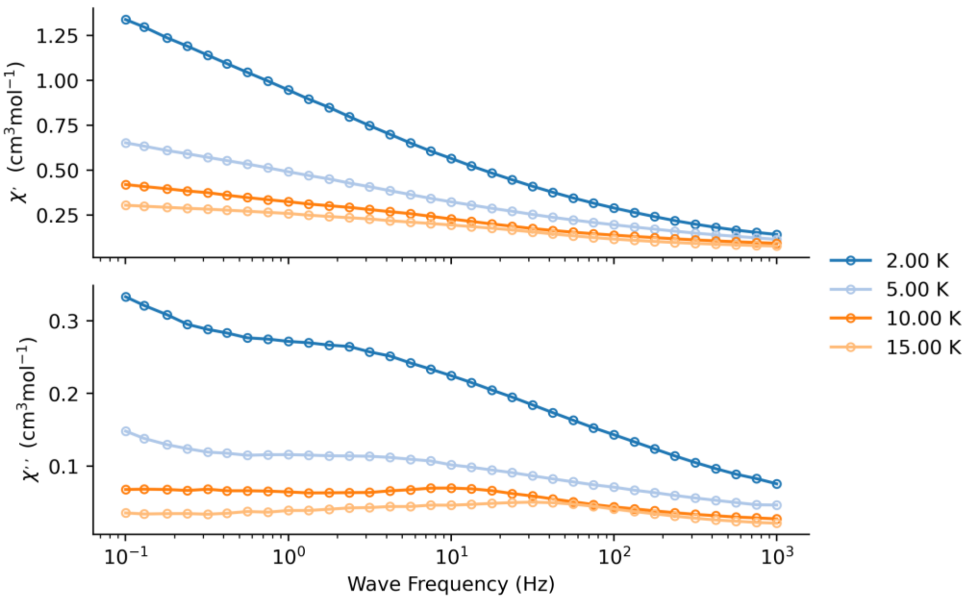


**Figure S101.** Ac susceptibility data on **4-Dy** for 2–15 K showing the minor relaxation channel appearing as a shoulder. Lines connect datapoints.

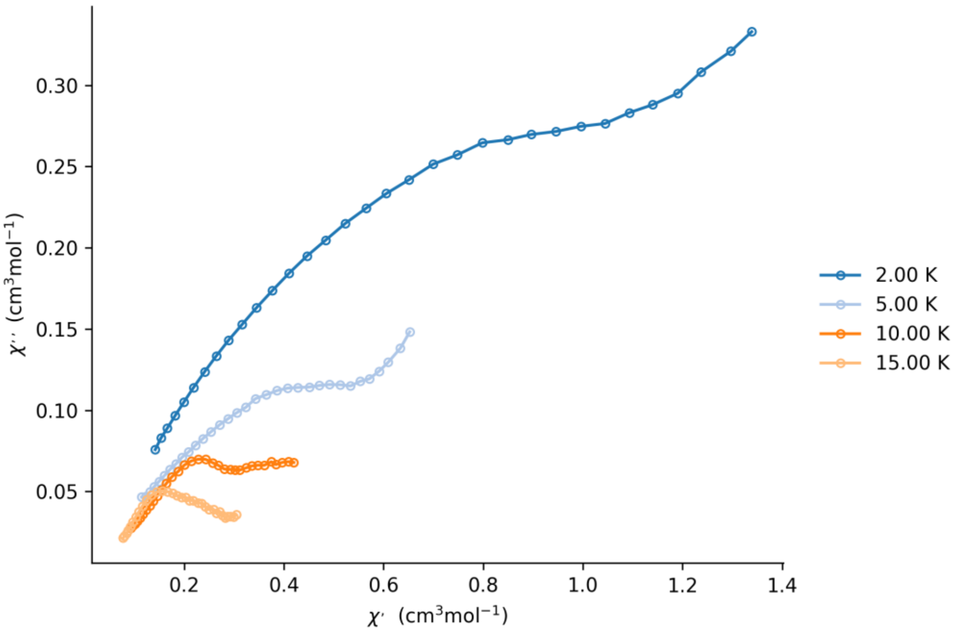


**Figure S102.** Cole-Cole plot of ac susceptibility data on **4-Dy** for 2–15 K showing the minor relaxation channel appearing as a shoulder. Lines connect datapoints.

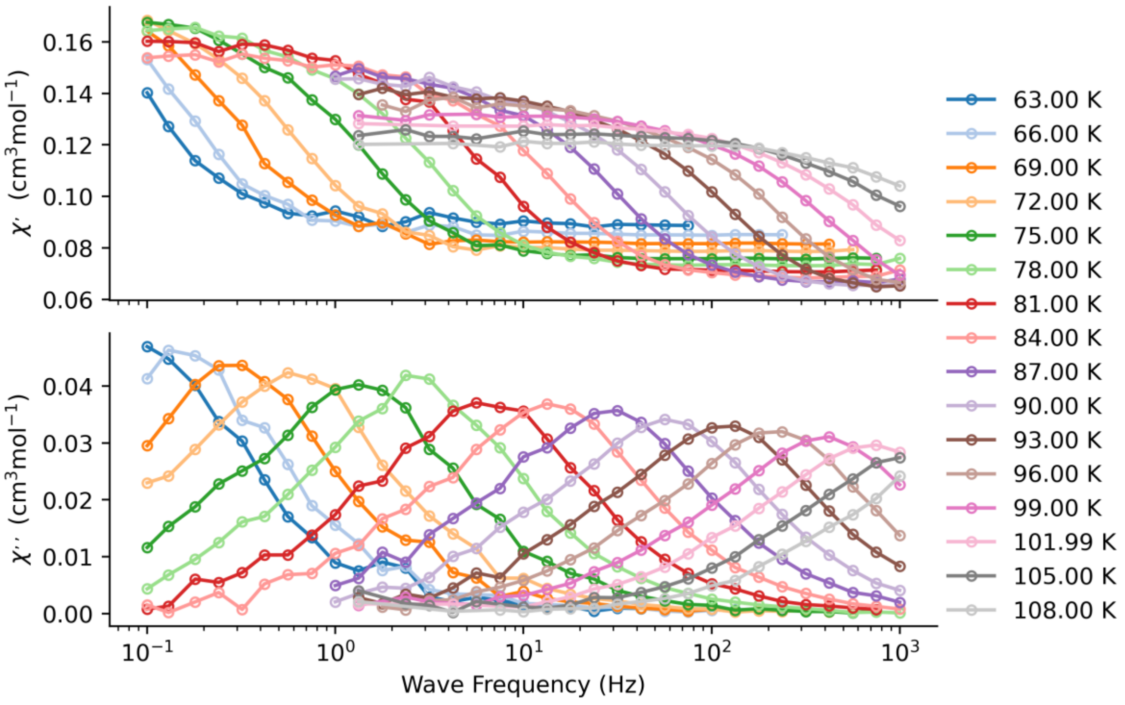


**Figure S103.** Ac data for "[{Dy(Cp$^{ttt}$)(Cp*)}{Al[OC($CF_3$)$_3$]$_4$}]" in zero field.

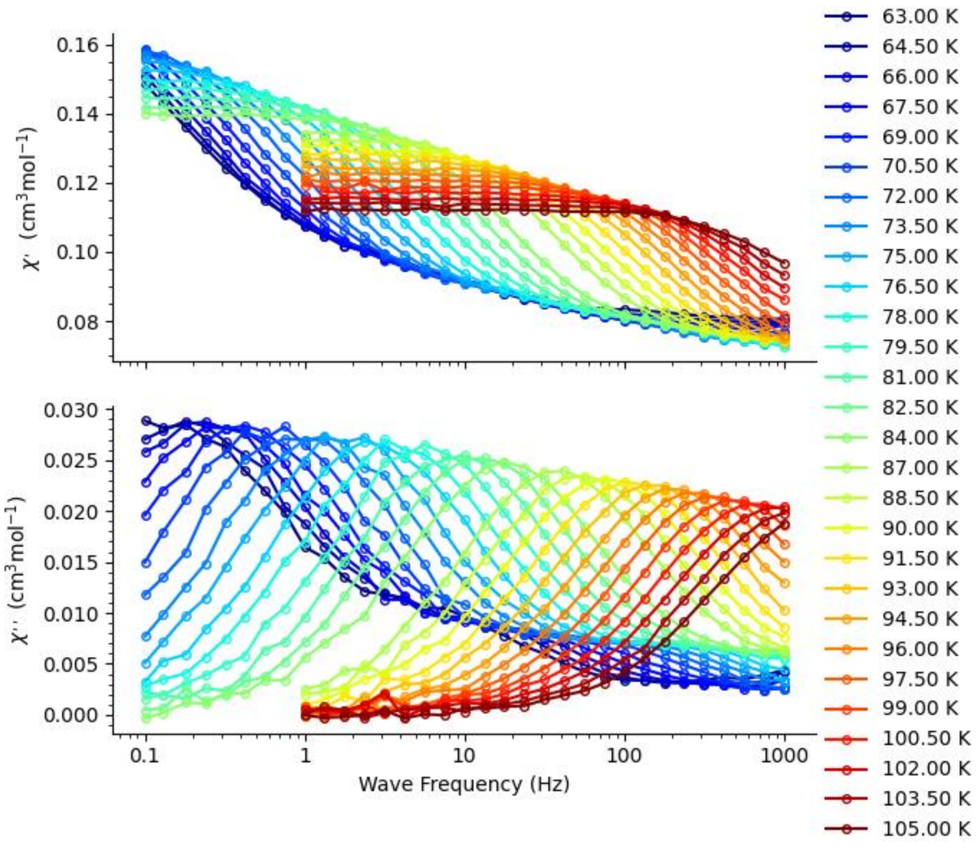


**Figure S104.** Ac susceptibility data on **4-Dy** in benzene for 63–105 K showing a high frequency shoulder. Lines connect datapoints.

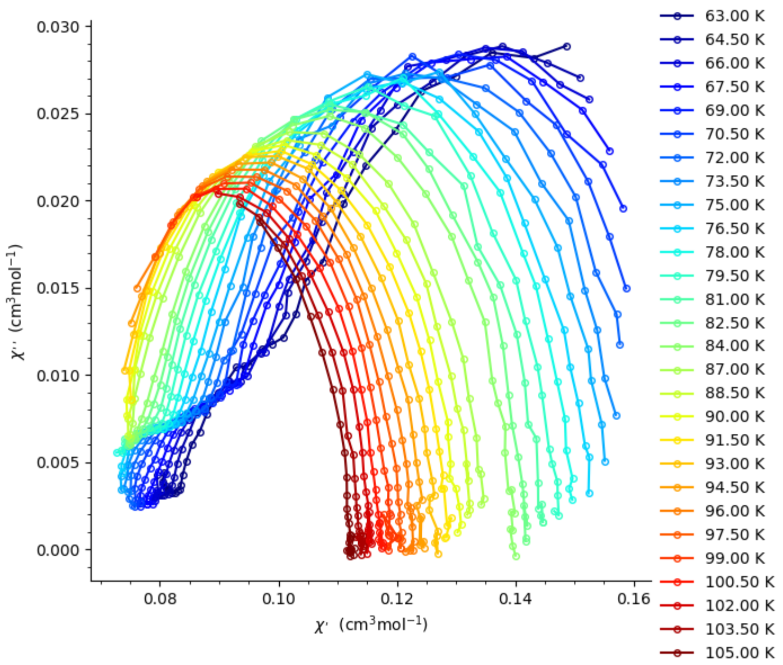


**Figure S105.** Cole-Cole plot of ac susceptibility data on **4-Dy** in benzene for 63–105 K showing a high frequency shoulder. Lines connect datapoints.

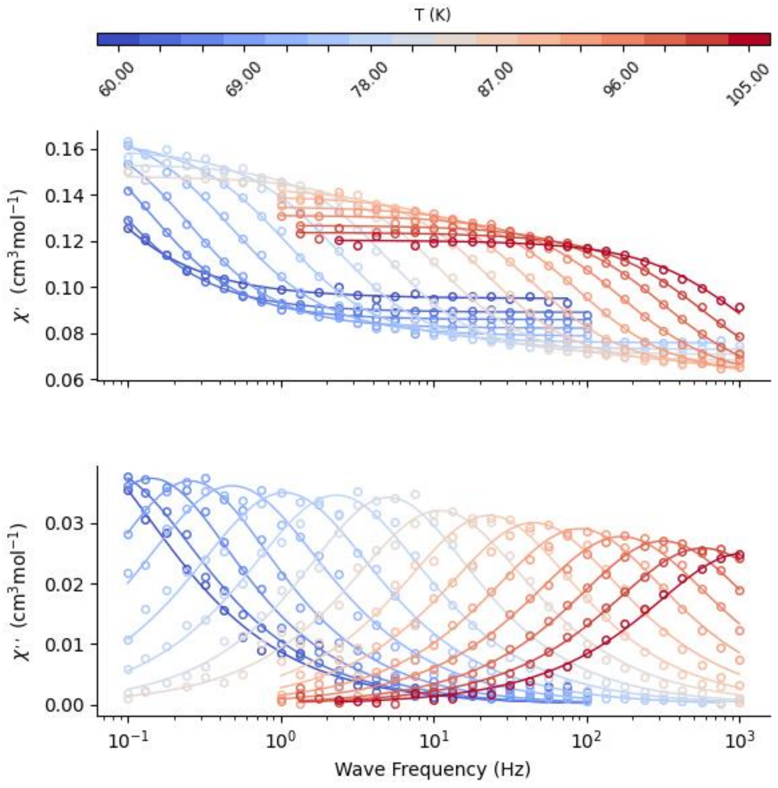


**Figure S106.** Fitting of ac data for **3-Dy·$C_6H_6$** in zero field to generalized Debye model in CC-FIT2.[38,39]

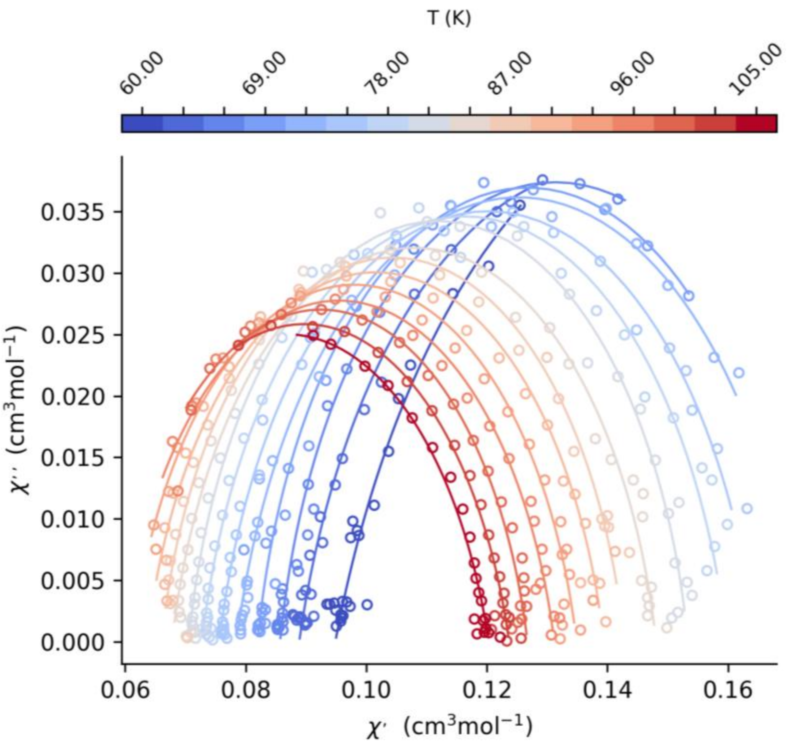


**Figure S107.** Cole-Cole plot showing fitting of ac data for **3-Dy·$C_6H_6$** in zero dc field to generalized Debye model in CC-FIT2.[38,39]

**Table S16.** Best fit parameters to the generalized Debye model for **3-Dy·$C_6H_6$** in zero dc field.

| T | $\tau_{debye}$ | $\tau_{debye}^{err}$ | $\chi_S$ | $\chi_S^{err}$ | $\chi_T$ | $\chi_T^{err}$ | α | $\alpha^{err}$ |
|---|---|---|---|---|---|---|---|---|
| (K) | (s) | | (emu/mol) | | (emu/mol) | | | |
| 60 | 4.5 | 1.3 | 9.487E-2 | 4.4E-4 | 0.223 | 2.0E-2 | 0.232 | 3.2E-2 |
| 63 | 2.22 | 0.18 | 8.888E-2 | 2.5E-4 | 0.1913 | 4.8E-3 | 0.179 | 1.5E-2 |
| 66 | 1.115 | 3.8E-2 | 8.570E-2 | 2.3E-4 | 0.1770 | 1.9E-3 | 0.126 | 1.1E-2 |
| 69 | 0.620 | 1.5E-2 | 8.196E-2 | 2.9E-4 | 0.1716 | 1.3E-3 | 0.122 | 1.0E-2 |
| 72 | 0.3292 | 7.7E-3 | 7.846E-2 | 3.9E-4 | 0.1721 | 1.1E-3 | 0.163 | 1.1E-2 |
| 75 | 0.1500 | 2.5E-3 | 7.562E-2 | 2.8E-4 | 0.16468 | 6.5E-4 | 0.1512 | 7.9E-3 |
| 78 | 6.87E-2 | 1.2E-3 | 7.275E-2 | 3.3E-4 | 0.15984 | 5.6E-4 | 0.1447 | 8.5E-3 |
| 81 | 3.139E-2 | 4.5E-4 | 7.054E-2 | 3.1E-4 | 0.15334 | 3.9E-4 | 0.1202 | 7.5E-3 |
| 84 | 1.453E-2 | 2.6E-4 | 6.780E-2 | 4.2E-4 | 0.14808 | 4.2E-4 | 0.1418 | 9.1E-3 |
| 87 | 6.91E-3 | 1.6E-4 | 6.605E-2 | 5.8E-4 | 0.14279 | 6.8E-4 | 0.130 | 1.3E-2 |
| 90 | 3.531E-3 | 6.8E-5 | 6.365E-2 | 5.5E-4 | 0.13940 | 4.6E-4 | 0.146 | 1.0E-2 |
| 93 | 1.773E-3 | 3.5E-5 | 6.117E-2 | 6.4E-4 | 0.13488 | 3.7E-4 | 0.150 | 1.0E-2 |
| 96 | 9.41E-4 | 1.8E-5 | 5.971E-2 | 6.9E-4 | 0.13119 | 3.1E-4 | 0.1591 | 9.5E-3 |
| 99 | 5.03E-4 | 1.2E-5 | 5.821E-2 | 9.1E-4 | 0.12673 | 2.8E-4 | 0.150 | 1.0E-2 |
| 102 | 2.75E-4 | 1.1E-5 | 5.58E-2 | 1.6E-3 | 0.12368 | 2.6E-4 | 0.171 | 1.4E-2 |
| 105 | 1.47E-4 | 1.3E-5 | 5.32E-2 | 3.5E-3 | 0.12029 | 2.9E-4 | 0.184 | 2.1E-2 |

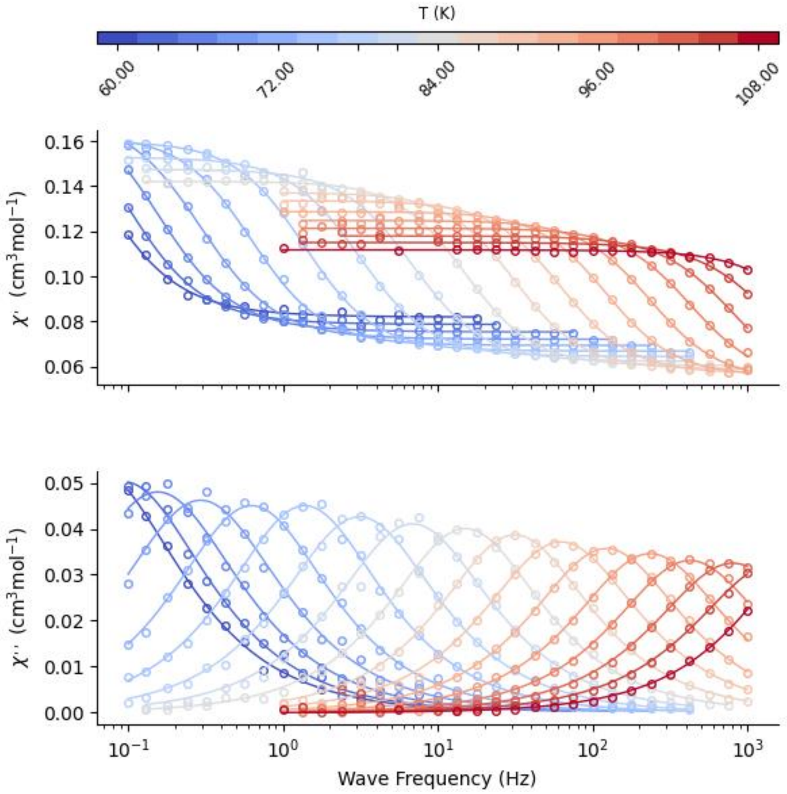


**Figure S108.** Fitting of ac data for **4-Dy** in zero field to generalized Debye model in CC-FIT2.[38,39]

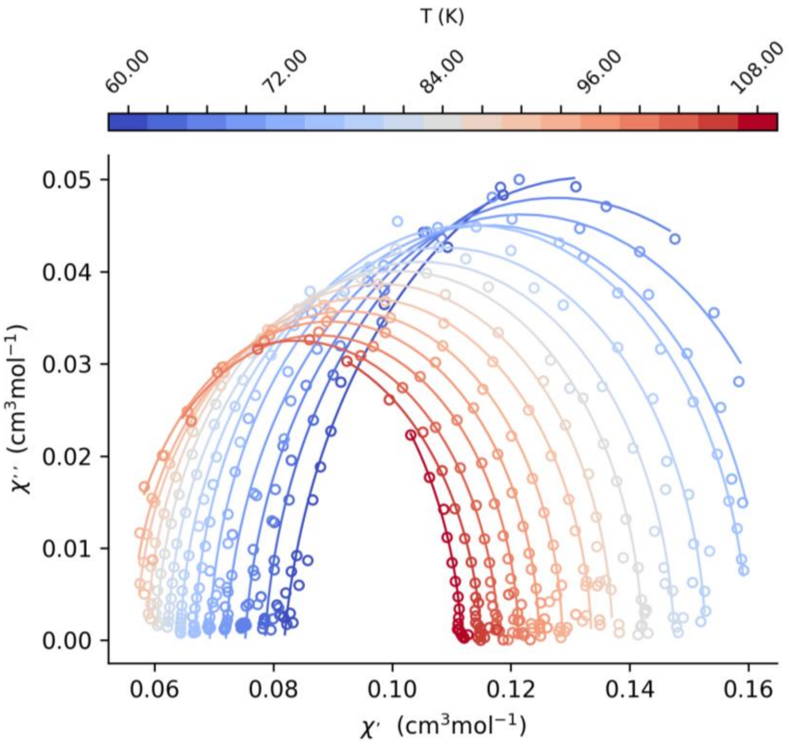


**Figure S109.** Cole-Cole plot showing fitting of ac data for **4-Dy** in zero dc field to generalized Debye model in CC-FIT2.[38,39]

**Table S17.** Best fit parameters to the generalized Debye model for **4-Dy** ac data in zero dc field.

| T | $\tau_{debye}$ | $\tau_{debye}^{err}$ | $\chi_S$ | $\chi_S^{err}$ | $\chi_T$ | $\chi_T^{err}$ | $\alpha$ | $\alpha^{err}$ |
|---|---|---|---|---|---|---|---|---|
| (K) | (s) | | (emu/mol) | | (emu/mol) | | | |
| 60 | 2.73 | 0.26 | 8.879E-2 | 4.1E-4 | 0.2219 | 8.8E-3 | 8.5E-2 | 2.0E-2 |
| 63 | 1.691 | 7.4E-2 | 8.526E-2 | 3.3E-4 | 0.2045 | 3.7E-3 | 5.7E-2 | 1.3E-2 |
| 66 | 1.025 | 2.1E-2 | 8.164E-2 | 2.4E-4 | 0.1953 | 1.6E-3 | 5.57E-2 | 8.4E-3 |
| 69 | 0.5408 | 8.4E-3 | 7.811E-2 | 2.9E-4 | 0.1863 | 1.1E-3 | 4.85E-2 | 8.2E-3 |
| 72 | 0.2533 | 2.2E-3 | 7.518E-2 | 2.2E-4 | 0.17645 | 5.3E-4 | 2.28E-2 | 5.5E-3 |
| 75 | 0.11509 | 6.7E-4 | 7.244E-2 | 1.6E-4 | 0.17336 | 2.8E-4 | 2.04E-2 | 3.6E-3 |
| 78 | 5.087E-2 | 3.3E-4 | 6.984E-2 | 2.0E-4 | 0.16571 | 2.5E-4 | 2.30E-2 | 4.0E-3 |
| 81 | 2.311E-2 | 2.6E-4 | 6.750E-2 | 3.7E-4 | 0.15995 | 3.8E-4 | 2.35E-2 | 7.0E-3 |
| 84 | 1.0563E-2 | 7.0E-5 | 6.550E-2 | 2.2E-4 | 0.15411 | 2.0E-4 | 1.24E-2 | 4.1E-3 |
| 87 | 5.052E-3 | 3.9E-5 | 6.383E-2 | 2.7E-4 | 0.14869 | 2.7E-4 | 7.2E-3 | 5.0E-3 |
| 90 | 2.545E-3 | 2.3E-5 | 6.174E-2 | 3.5E-4 | 0.14485 | 2.6E-4 | 1.96E-2 | 5.7E-3 |
| 93 | 1.296E-3 | 1.3E-5 | 6.082E-2 | 4.3E-4 | 0.13954 | 2.3E-4 | 1.03E-2 | 6.3E-3 |
| 96 | 6.759E-4 | 6.6E-6 | 5.871E-2 | 4.9E-4 | 0.13531 | 1.9E-4 | 1.31E-2 | 6.1E-3 |
| 99 | 3.741E-4 | 5.6E-6 | 5.846E-2 | 8.2E-4 | 0.13153 | 1.9E-4 | 1.12E-2 | 8.3E-3 |
| 102 | 1.975E-4 | 8.1E-6 | 5.38E-2 | 2.3E-3 | 0.12791 | 3.1E-4 | 3.2E-2 | 1.6E-2 |
| 105 | 1.04E-4 | 1.0E-5 | 4.90E-2 | 5.4E-3 | 0.12473 | 2.6E-4 | 4.6E-2 | 2.3E-2 |
| 108 | 4.71E-5 | 6.1E-6 | 3.21E-2 | 8.7E-3 | 0.12116 | 1.2E-4 | 4.5E-2 | 1.6E-2 |

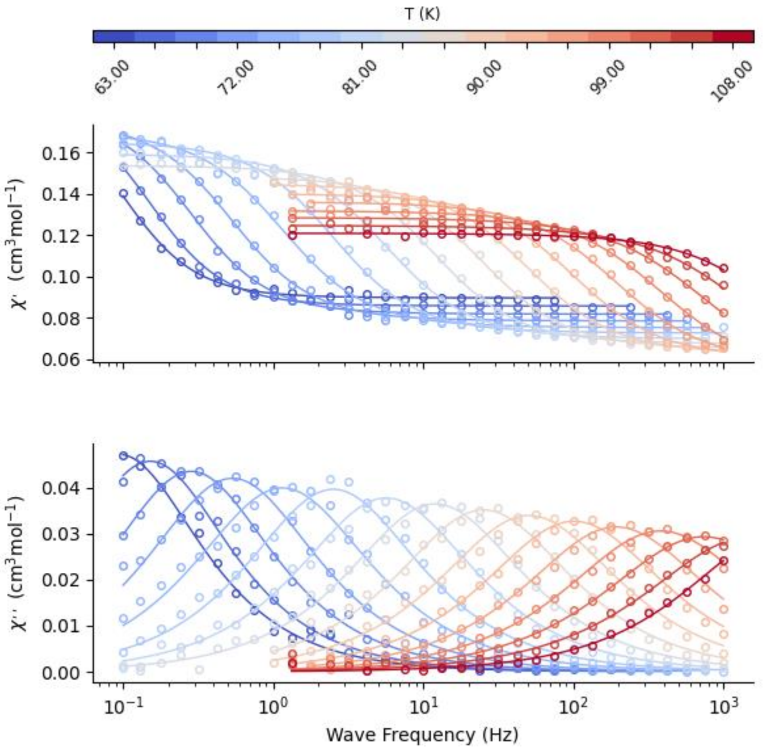


**Figure S110.** Fitting of ac data for "[{Dy(Cp$^{ttt}$)(Cp*)}{Al[OC($CF_3$)$_3$]$_4$}]" in zero field to generalized Debye model in CC-FIT2.[38,39]

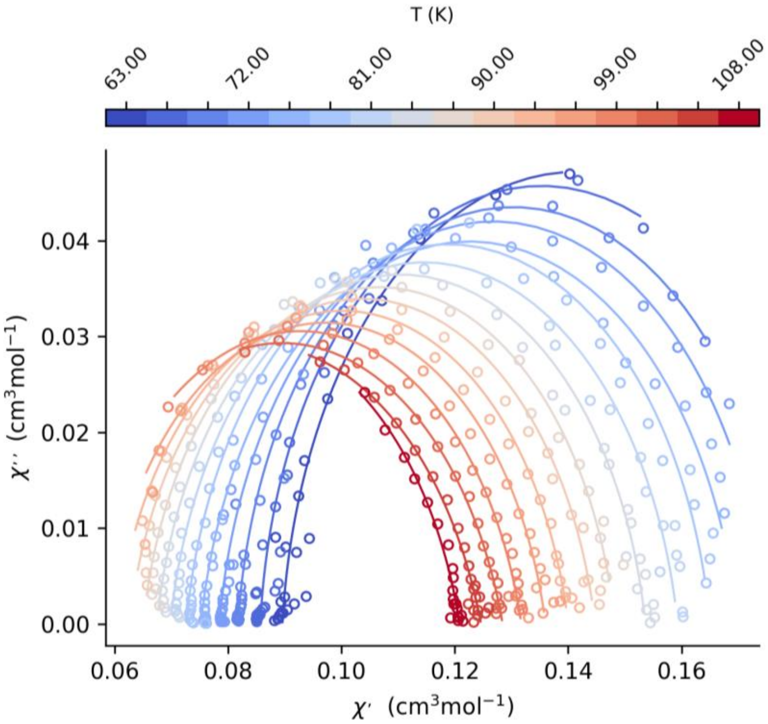


**Figure S111.** Cole-Cole plot showing fitting of ac data for "[{Dy(Cp$^{ttt}$)(Cp*)}-{Al[OC($CF_3$)$_3$]$_4$}]" in zero dc field to generalized Debye model in CC-FIT2.[38,39]

**Table S18.** Best fit parameters to the generalized Debye model for "[{Dy(Cp$^{ttt}$)(Cp*)}{Al[OC($CF_3$)$_3$]$_4$}]" in zero dc field.

| T | $\tau_{debye}$ | $\tau_{debye}^{err}$ | $\chi_S$ | $\chi_S^{err}$ | $\chi_T$ | $\chi_T^{err}$ | α | $\alpha^{err}$ |
|---|---|---|---|---|---|---|---|---|
| (K) | (s) | | (emu/mol) | | (emu/mol) | | | |
| 63.00 | 1.675 | 9.5E-2 | 8.960E-2 | 3.3E-4 | 0.1929 | 4.2E-3 | 5.8E-2 | 1.7E-2 |
| 66.00 | 1.046 | 2.8E-2 | 8.570E-2 | 2.4E-4 | 0.1849 | 1.9E-3 | 5.2E-2 | 1.1E-2 |
| 69.00 | 0.5585 | 8.5E-3 | 8.176E-2 | 2.1E-4 | 0.17882 | 9.6E-4 | 6.95E-2 | 7.5E-3 |
| 72.00 | 0.2971 | 4.3E-3 | 7.847E-2 | 2.5E-4 | 0.17524 | 7.8E-4 | 8.98E-2 | 7.6E-3 |
| 75.00 | 0.1378 | 2.3E-3 | 7.522E-2 | 3.3E-4 | 0.16997 | 7.1E-4 | 0.1085 | 8.7E-3 |
| 78.00 | 6.309E-2 | 9.9E-4 | 7.263E-2 | 3.5E-4 | 0.16506 | 5.7E-4 | 9.76E-2 | 8.5E-3 |
| 81.00 | 2.808E-2 | 4.7E-4 | 6.981E-2 | 4.2E-4 | 0.15932 | 4.9E-4 | 0.1080 | 8.9E-3 |
| 84.00 | 1.324E-2 | 2.3E-4 | 6.742E-2 | 4.7E-4 | 0.15362 | 4.5E-4 | 0.1059 | 9.5E-3 |
| 87.00 | 6.31E-3 | 1.2E-4 | 6.502E-2 | 5.2E-4 | 0.14825 | 5.8E-4 | 0.107 | 1.0E-2 |
| 90.00 | 3.143E-3 | 5.8E-5 | 6.258E-2 | 6.0E-4 | 0.14475 | 4.8E-4 | 0.121 | 1.0E-2 |
| 93.00 | 1.575E-3 | 2.8E-5 | 6.034E-2 | 6.6E-4 | 0.14001 | 3.9E-4 | 0.1252 | 9.7E-3 |
| 96.00 | 8.23E-4 | 1.9E-5 | 5.830E-2 | 9.5E-4 | 0.13601 | 3.9E-4 | 0.134 | 1.2E-2 |
| 99.00 | 4.171E-4 | 9.0E-6 | 5.392E-2 | 9.7E-4 | 0.13174 | 2.4E-4 | 0.1523 | 8.9E-3 |
| 101.99 | 2.152E-4 | 6.2E-6 | 5.06E-2 | 1.3E-3 | 0.12844 | 1.8E-4 | 0.1777 | 8.4E-3 |
| 105.00 | 1.08E-4 | 1.5E-5 | 4.53E-2 | 6.0E-3 | 0.12463 | 3.2E-4 | 0.194 | 2.4E-2 |
| 108.00 | 6.2E-5 | 1.0E-0 | 4.22E-2 | 8.1E-3 | 0.12086 | 2.1E-4 | 0.162 | 2.3E-2 |

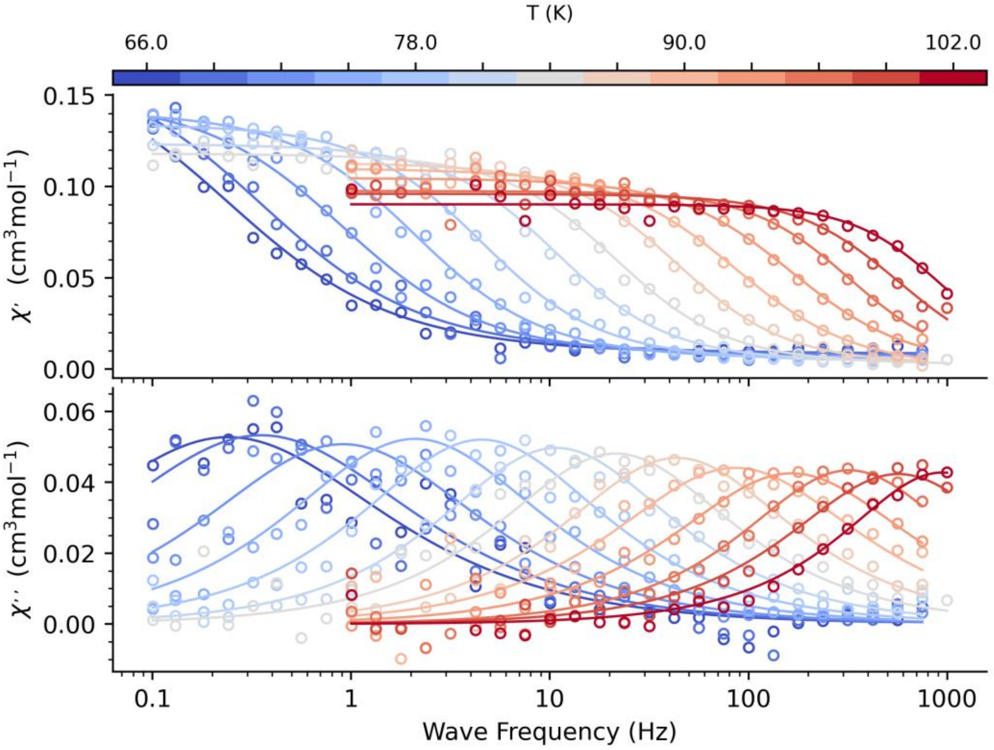


**Figure S112.** Fitting of ac data for **5%Dy@4-Y** in zero dc field to generalized Debye model in CC-FIT2.[38,39] Calculated per mole of Dy.

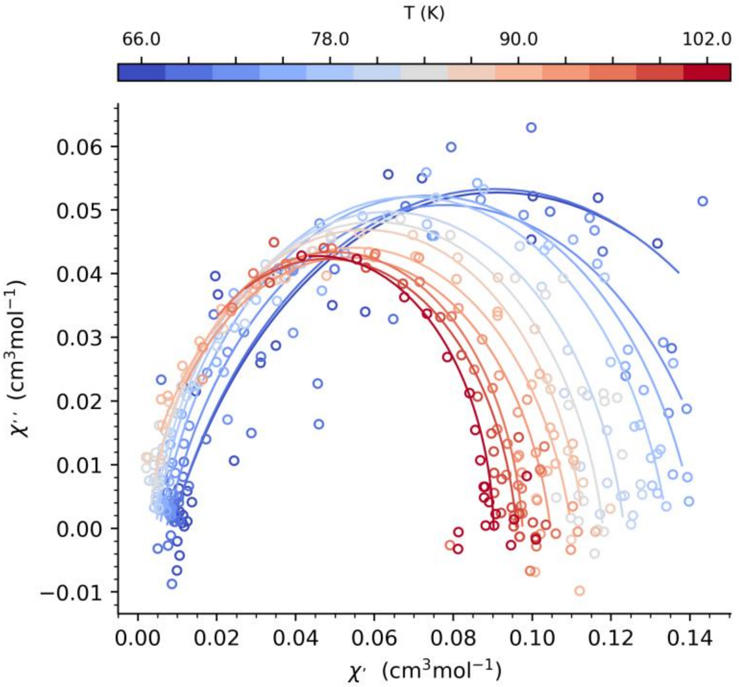


**Figure S113.** Cole-Cole plot showing fitting of ac data for **5%Dy@4-Y** in zero dc field to generalized Debye model in CC-FIT2.[38,39] Calculated per mole of Dy.

**Table S19.** Best fit parameters to the generalized Debye model for **5%Dy@4-Y** ac data in zero dc field.

| T | $\tau_{debye}$ | $\tau_{debye}^{err}$ | $\chi_S$ | $\chi_S^{err}$ | $\chi_T$ | $\chi_T^{err}$ | α | $\alpha^{err}$ |
|---|---|---|---|---|---|---|---|---|
| (K) | (s) | | (emu/mol) | | (emu/mol) | | | |
| 66 | 0.662 | 9.2E-2 | 4.19E-4 | 7.5E-5 | 8.71E-3 | 5.2E-4 | 0.278 | 3.5E-2 |
| 69 | 0.452 | 5.4E-2 | 4.26E-4 | 8.4E-5 | 8.62E-3 | 4.4E-4 | 0.266 | 3.5E-2 |
| 72 | 0.1752 | 8.0E-3 | 3.63E-4 | 5.1E-5 | 7.42E-3 | 1.4E-4 | 0.206 | 1.9E-2 |
| 75 | 7.58E-2 | 2.5E-3 | 2.75E-4 | 5.2E-5 | 7.071E-3 | 8.6E-5 | 0.165 | 1.6E-2 |
| 78 | 3.50E-2 | 1.0E-3 | 2.25E-4 | 5.1E-5 | 6.719E-3 | 6.4E-5 | 0.140 | 1.5E-2 |
| 81 | 1.476E-2 | 4.4E-4 | 1.91E-4 | 5.7E-5 | 6.167E-3 | 5.2E-5 | 0.116 | 1.6E-2 |
| 84 | 7.43E-3 | 3.4E-4 | 1.26E-4 | 9.0E-5 | 5.900E-3 | 7.0E-5 | 0.115 | 2.4E-2 |
| 87 | 3.65E-3 | 1.2E-4 | 1.17E-4 | 7.8E-5 | 5.655E-3 | 6.5E-5 | 0.105 | 2.0E-2 |
| 90 | 1.85E-3 | 1.3E-4 | 0.0E-4 | 1.8E-4 | 5.497E-3 | 9.5E-5 | 0.139 | 3.6E-2 |
| 93 | 9.65E-4 | 5.0E-5 | 8E-5 | 1.5E-4 | 5.232E-3 | 5.5E-5 | 0.121 | 2.6E-2 |
| 96 | 4.95E-4 | 4.1E-5 | 9E-5 | 2.7E-4 | 4.878E-3 | 6.0E-5 | 6.2E-2 | 4.0E-2 |
| 99 | 2.80E-4 | 2.6E-5 | 0.0E-4 | 3.0E-4 | 4.801E-3 | 5.3E-5 | 8.0E-2 | 3.9E-2 |
| 102 | 1.68E-4 | 2.6E-5 | 7E-5 | 5.3E-4 | 4.511E-3 | 5.4E-5 | 2.4E-2 | 5.5E-2 |

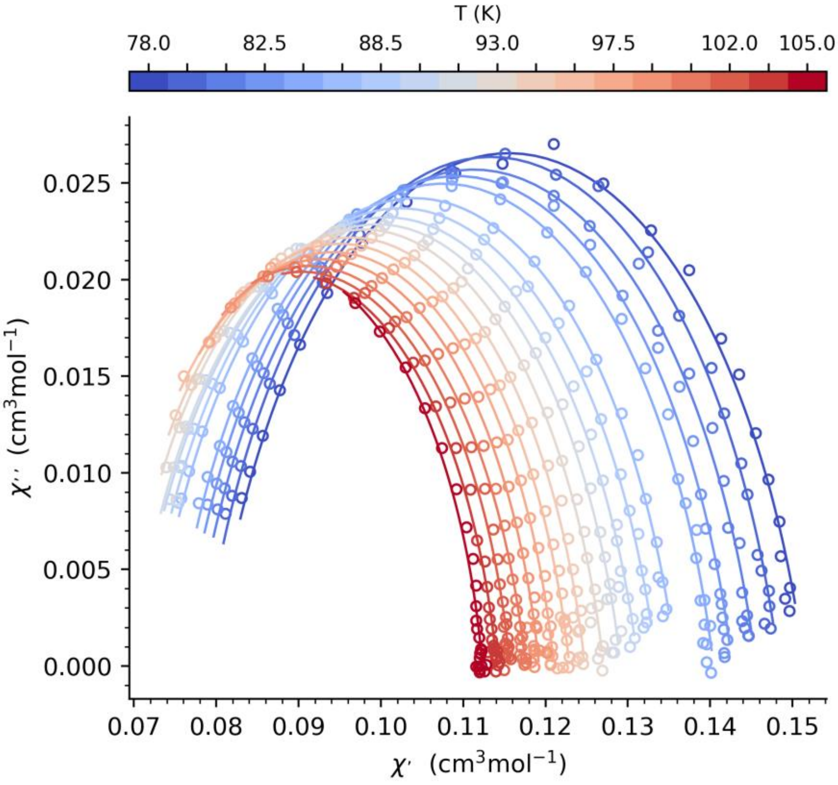


**Figure S114.** Fitting of ac data for **4-Dy** in benzene in zero dc field (78–105 K) to generalized Debye model in CC-FIT2.[38,39]

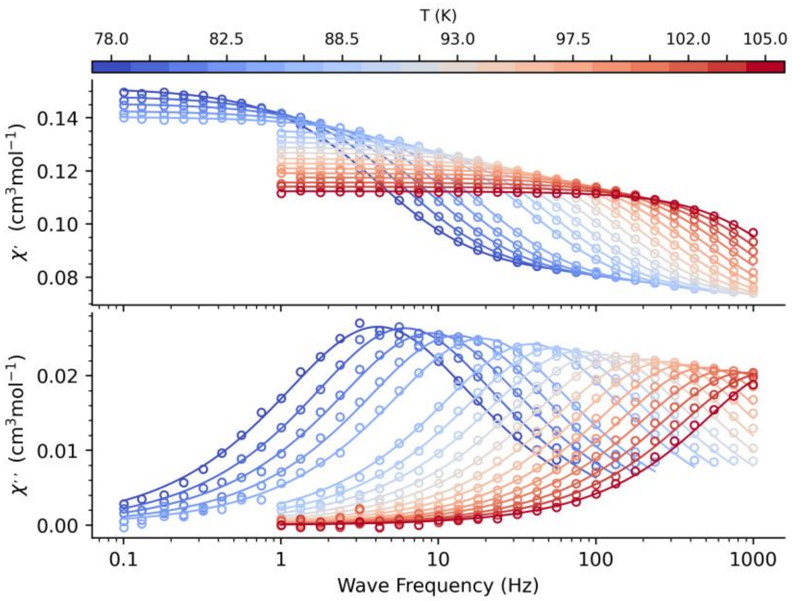


**Figure S115.** Cole-Cole plot showing fitting of trimmed ac data for **4-Dy** in benzene in zero dc field (78–105 K) to generalized Debye model in CC-FIT2.[38,39]

**Table S20.** Best fit parameters to the generalized Debye model for **4-Dy** in benzene ac data in zero dc field (78–105 K).

| T | $\tau_{\text{debye}}$ | $\tau_{\text{debye}}^{\text{err}}$ | $\chi_S$ | $\chi_S^{\text{err}}$ | $\chi_T$ | $\chi_T^{\text{err}}$ | α | $\alpha^{\text{err}}$ |
|---|---|---|---|---|---|---|---|---|
| (K) | (s) | | (emu/mol) | | (emu/mol) | | | |
| 78.0 | 3.851E-2 | 4.1E-4 | 7.963E-2 | 3.1E-4 | 0.15148 | 2.3E-4 | 0.1899 | 5.4E-3 |
| 79.5 | 2.597E-2 | 2.8E-4 | 7.839E-2 | 3.0E-4 | 0.14836 | 2.1E-4 | 0.1782 | 5.4E-3 |
| 81.0 | 1.785E-2 | 2.3E-4 | 7.694E-2 | 3.5E-4 | 0.14558 | 2.3E-4 | 0.1818 | 6.3E-3 |
| 82.5 | 1.220E-2 | 1.4E-4 | 7.581E-2 | 3.2E-4 | 0.14271 | 1.9E-4 | 0.1740 | 5.8E-3 |
| 84.0 | 8.478E-3 | 8.7E-5 | 7.474E-2 | 2.8E-4 | 0.14042 | 1.5E-4 | 0.1723 | 5.0E-3 |
| 87.0 | 4.115E-3 | 3.9E-5 | 7.222E-2 | 2.6E-4 | 0.13589 | 1.8E-4 | 0.1721 | 4.9E-3 |
| 88.5 | 2.909E-3 | 3.2E-5 | 7.116E-2 | 3.0E-4 | 0.13350 | 1.8E-4 | 0.1725 | 5.6E-3 |
| 90.0 | 2.079E-3 | 2.0E-5 | 7.018E-2 | 2.6E-4 | 0.13126 | 1.4E-4 | 0.1723 | 4.7E-3 |
| 91.5 | 1.509E-3 | 1.9E-5 | 6.995E-2 | 3.4E-4 | 0.12916 | 1.8E-4 | 0.1627 | 6.4E-3 |
| 93.0 | 1.080E-3 | 1.2E-5 | 6.874E-2 | 3.2E-4 | 0.12706 | 1.4E-4 | 0.1606 | 5.5E-3 |
| 94.5 | 7.86E-4 | 1.0E-5 | 6.805E-2 | 3.9E-4 | 0.12478 | 1.4E-4 | 0.1552 | 6.2E-3 |
| 96.0 | 5.782E-4 | 8.6E-6 | 6.716E-2 | 4.6E-4 | 0.12291 | 1.3E-4 | 0.1535 | 6.5E-3 |
| 97.5 | 4.366E-4 | 6.9E-6 | 6.727E-2 | 4.9E-4 | 0.12101 | 1.1E-4 | 0.1431 | 6.5E-3 |
| 99.0 | 3.248E-4 | 8.3E-6 | 6.651E-2 | 8.0E-4 | 0.11922 | 1.4E-4 | 0.1416 | 9.5E-3 |
| 100.5 | 2.353E-4 | 7.7E-6 | 6.49E-2 | 1.0E-3 | 0.11758 | 1.3E-4 | 0.152 | 1.0E-2 |
| 102.0 | 1.819E-4 | 8.9E-6 | 6.51E-2 | 1.5E-3 | 0.11568 | 1.4E-4 | 0.135 | 1.4E-2 |
| 103.5 | 1.352E-4 | 7.5E-6 | 6.39E-2 | 1.7E-3 | 0.11408 | 1.1E-4 | 0.135 | 1.3E-2 |
| 105.0 | 9.89E-5 | 8.2E-6 | 6.17E-2 | 2.7E-3 | 0.11234 | 1.1E-4 | 0.125 | 1.6E-2 |

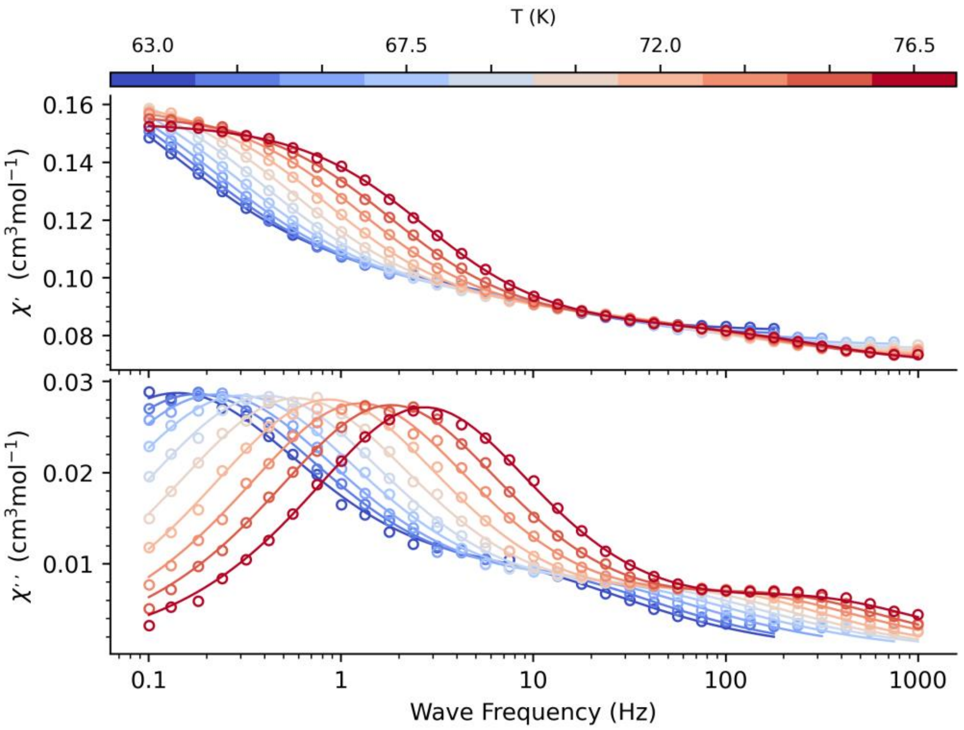


**Figure S116.** Fitting of ac data for **4-Dy** in benzene in zero dc field (63–76.5 K) to double generalized Debye model in CC-FIT2.[38,39]

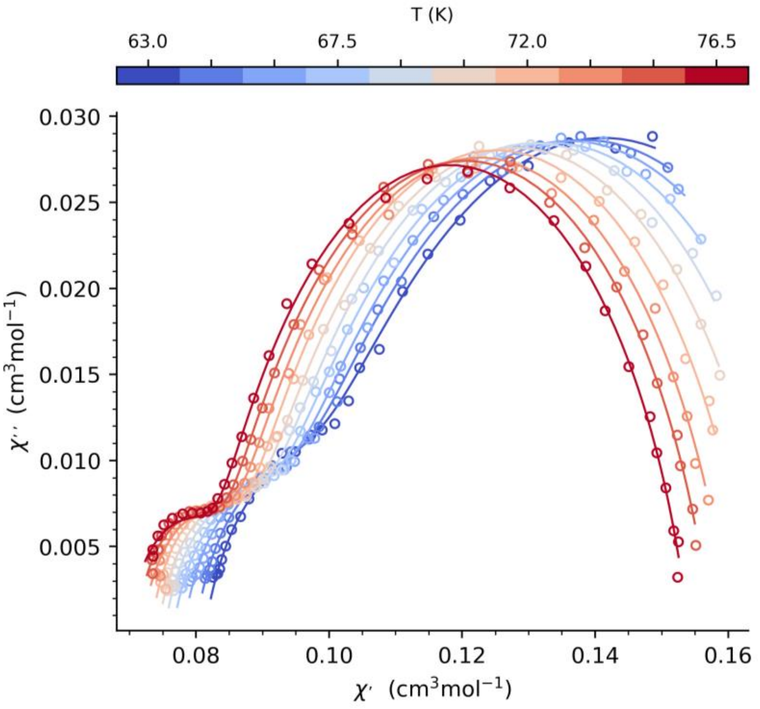


**Figure S117.** Cole-Cole plot showing fitting of ac data for **4-Dy** in benzene in zero dc field (63–76.5 K) to double generalized Debye model in CC-FIT2.[38,39]

**Table S21.** Best fit parameters to the double generalized Debye model for **4-Dy** in benzene ac data in zero dc field (63–76.5 K).

| T | $\tau_{\mathrm{debye},1}$ | $\tau_{\mathrm{debye},1}^{\mathrm{err}}$ | $\tau_{\mathrm{debye},2}$ | $\tau_{\mathrm{debye},2}^{\mathrm{err}}$ | $\chi_{\mathrm{Total}}$ | $\chi_{\mathrm{Total}}^{\mathrm{err}}$ | $\Delta\chi_1$ | $\Delta\chi_1^{\mathrm{err}}$ | $\Delta\chi_2$ | $\Delta\chi_2^{\mathrm{err}}$ | $\alpha_1$ | $\alpha_1^{\mathrm{err}}$ | $\alpha_2$ | $\alpha_2^{\mathrm{err}}$ | $\eta_1$[a] |
|---|---|---|---|---|---|---|---|---|---|---|---|---|---|---|---|
| (K) | (s) | | (s) | | (emu/mol) | | (emu/mol) | | (emu/mol) | | | | | | |
| 63.0 | 1.53E-2 | 1.7E-3 | 1.173 | 4.6E-2 | 8.132E-2 | 3.1E-4 | 1.62E-2 | 1.8E-3 | 8.96E-2 | 3.9E-3 | 0.214 | 4.2E-2 | 0.284 | 2.1E-2 | 0.153 |
| 64.5 | 1.059E-2 | 8.3E-4 | 9.21E-1 | 2.3E-2 | 7.990E-2 | 2.9E-4 | 1.53E-2 | 1.2E-3 | 8.80E-2 | 2.4E-3 | 0.195 | 3.5E-2 | 0.274 | 1.4E-2 | 0.148 |
| 66.0 | 6.97E-3 | 5.9E-4 | 7.32E-1 | 1.7E-2 | 7.808E-2 | 3.3E-4 | 1.55E-2 | 1.2E-3 | 8.67E-2 | 2.1E-3 | 0.211 | 3.8E-2 | 0.266 | 1.3E-2 | 0.152 |
| 67.5 | 4.77E-3 | 3.3E-4 | 5.472E-1 | 8.2E-3 | 7.648E-2 | 2.4E-4 | 1.585E-2 | 8.6E-4 | 8.40E-2 | 1.4E-3 | 0.235 | 2.8E-2 | 0.2499 | 9.0E-3 | 0.159 |
| 69.0 | 3.29E-3 | 1.9E-4 | 3.947E-1 | 4.8E-3 | 7.525E-2 | 2.3E-4 | 1.553E-2 | 7.2E-4 | 8.15E-2 | 1.0E-3 | 0.219 | 2.6E-2 | 0.2315 | 7.4E-3 | 0.160 |
| 70.5 | 2.25E-3 | 1.4E-4 | 2.758E-1 | 3.6E-3 | 7.432E-2 | 2.9E-4 | 1.449E-2 | 7.6E-4 | 7.962E-2 | 9.6E-4 | 0.186 | 3.3E-2 | 0.2217 | 7.6E-3 | 0.154 |
| 72.0 | 1.515E-3 | 8.8E-5 | 1.872E-1 | 2.2E-3 | 7.330E-2 | 3.2E-4 | 1.374E-2 | 7.0E-4 | 7.701E-2 | 7.7E-4 | 0.152 | 3.4E-2 | 0.2037 | 6.8E-3 | 0.151 |
| 73.5 | 1.071E-3 | 6.5E-5 | 1.286E-1 | 1.5E-3 | 7.186E-2 | 3.9E-4 | 1.408E-2 | 7.8E-4 | 7.447E-2 | 7.0E-4 | 0.186 | 3.6E-2 | 0.1938 | 6.1E-3 | 0.159 |
| 75.0 | 7.33E-4 | 3.9E-5 | 8.859E-2 | 8.7E-4 | 7.094E-2 | 4.0E-4 | 1.324E-2 | 7.0E-4 | 7.338E-2 | 5.4E-4 | 0.156 | 3.5E-2 | 0.1879 | 5.1E-3 | 0.153 |
| 76.5 | 4.95E-4 | 3.5E-5 | 5.932E-2 | 6.4E-4 | 6.975E-2 | 5.9E-4 | 1.313E-2 | 9.3E-4 | 7.122E-2 | 5.7E-4 | 0.172 | 4.5E-2 | 0.1756 | 5.3E-3 | 0.156 |

[a] fraction of component 1, $\eta_1 = \Delta\chi_1/(\Delta\chi_1 + \Delta\chi_2)$

*9.3 Waveform Measurements*

Waveform measurements were performed as described by Hilgar *et al.* to characterize the relaxation rate in the intermediate temperature range.[40] Measurements were performed using a Quantum Design MPMS3 superconducting quantum interference device (SQUID) magnetometer. Samples of **3-Dy·$C_6H_6$** (16.8 mg) and **4-Dy** (17.5 mg) were prepared as described in §9.1 with 13.5 mg and 14.3 mg of eicosane respectively. Before the first measurement, the samples were centered in 1 kOe field (60 K for **3-Dy·$C_6H_6$**, 84 K for **4-Dy**), then the magnet was reset at 300 K and the sample cooled in zero field. Measurements were performed at fixed temperatures between 32–56 K for **3-Dy·$C_6H_6$** (Figures S118–S123) and 20–54 K for **4-Dy** (Figures S124–S133). Continuous measurements were performed in VSM mode with 1 mm amplitude and 0.5 s averaging time. A square wave was applied, by sweeping the field to +8 Oe at 700 Oe $s^{-1}$ (linear sweep), holding for time *t* (14–2776 s), then sweeping the field to –8 Oe at 700 Oe $s^{-1}$ (linear sweep) and holding for time *t*. The square wave field cycle was repeated for a total of 3–20 times and then the field was returned to 0 Oe at 700 Oe $s^{-1}$ (linear) and measurements ceased.

Waveform data was processed in CC-FIT2[38,39], according to the method developed by Hilgar *et al.* to extract the complex susceptibility, $\chi$.[40] A discrete Fourier transform was performed on the time dependent field and magnetic moment data and $\chi$ was defined as the ratio of absolute values of the complex moment and field spectra at the fundamental drive field frequency. The in-phase ($\chi'$) and out-of-phase ($\chi''$) susceptibilities were calculated as $\chi' = \chi\cos\phi$ and $\chi'' = \chi\sin\phi$ with $\phi$ as the phase angle between the field and moment spectra at the fundamental frequency.[40] Extracted values are reported in Tables S22 and S23. The in-phase and out-of-phase susceptibilities were fit to the Generalised Debye (GD) model in CC-FIT2 (Equation S1) to extract relaxation rates and distributions (Figures S134 and S135 and Table S24 for **3-Dy·$C_6H_6$** and Figures S136 and S137 and Table S25 for **4-Dy**). Cole-Cole fits are

good for **4-Dy** but poorer for **3-Dy·$C_6H_6$**, suggesting the distribution is asymmetric for **3-Dy·$C_6H_6$**. The position of the peak for **3-Dy·$C_6H_6$** suggests rates are slightly underestimated by the GD model. Fits were not improved by using the Havriliak-Negami model which includes a skew parameter (skewed towards faster rates) in addition to α.[41]

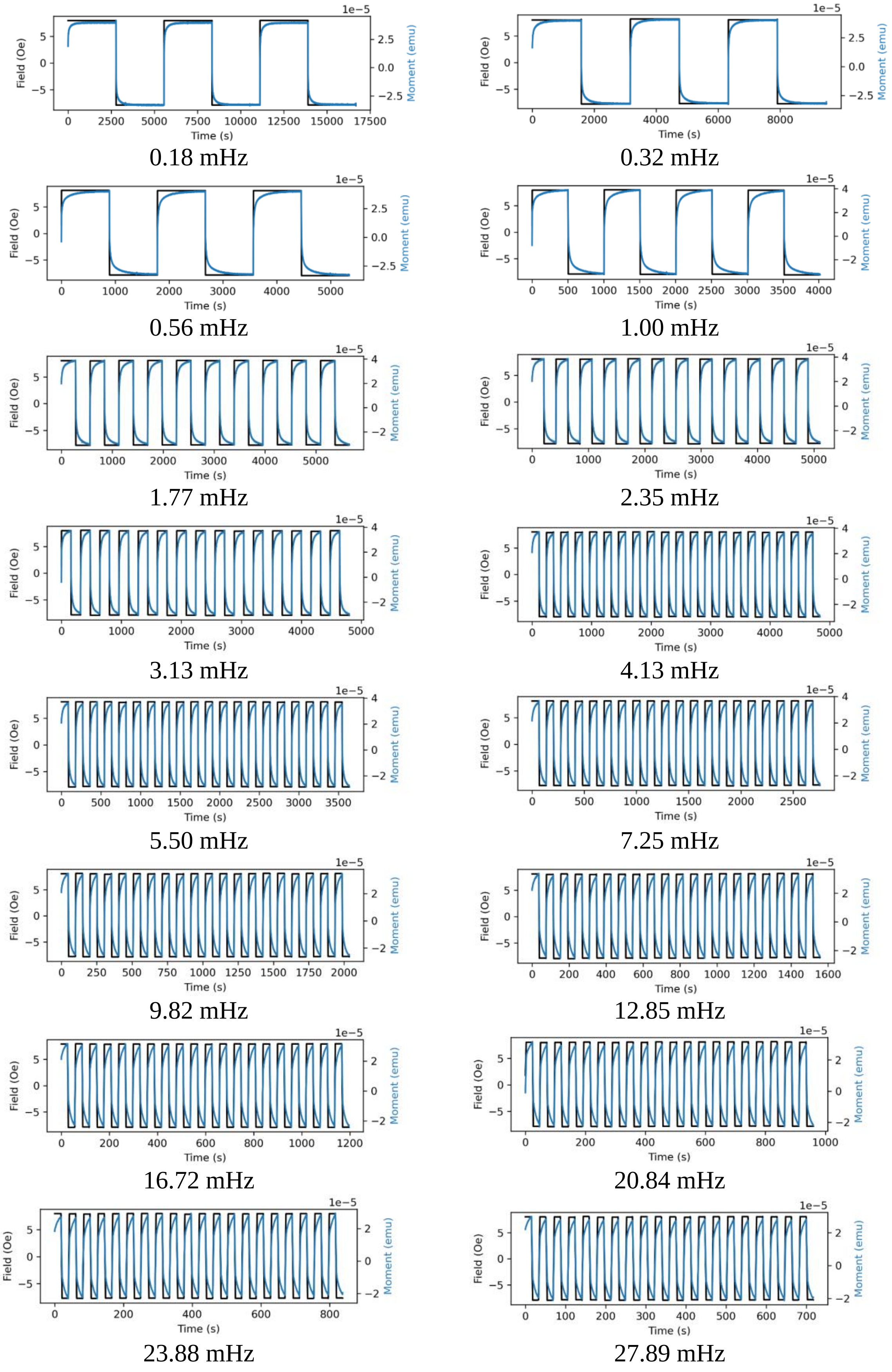


**Figure S118.** Raw waveform data and corresponding frequencies for **3-Dy·$C_6H_6$** at 32 K.

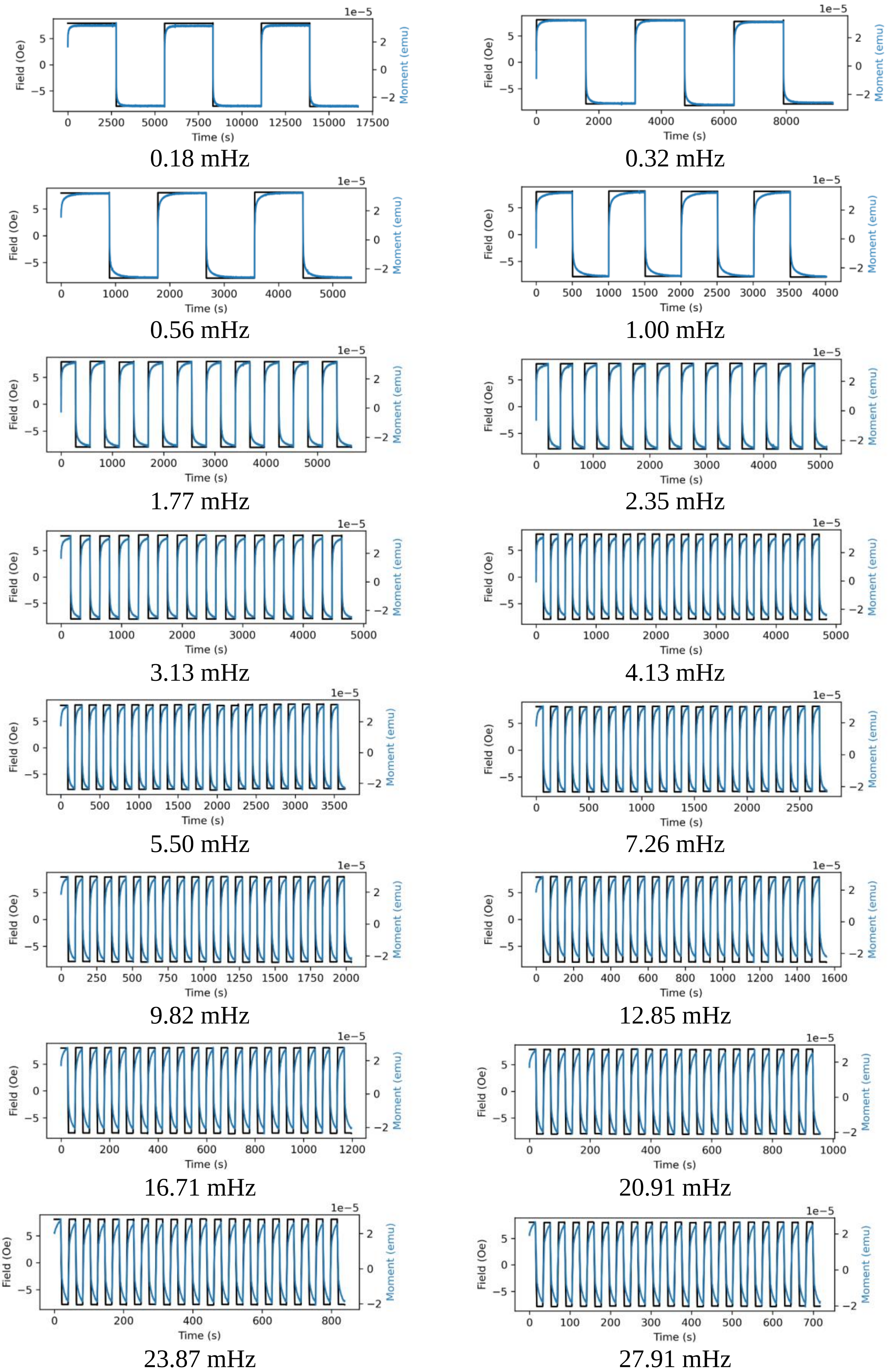


**Figure S119.** Raw waveform data and corresponding frequencies for **3-Dy·$C_6H_6$** at 40 K.

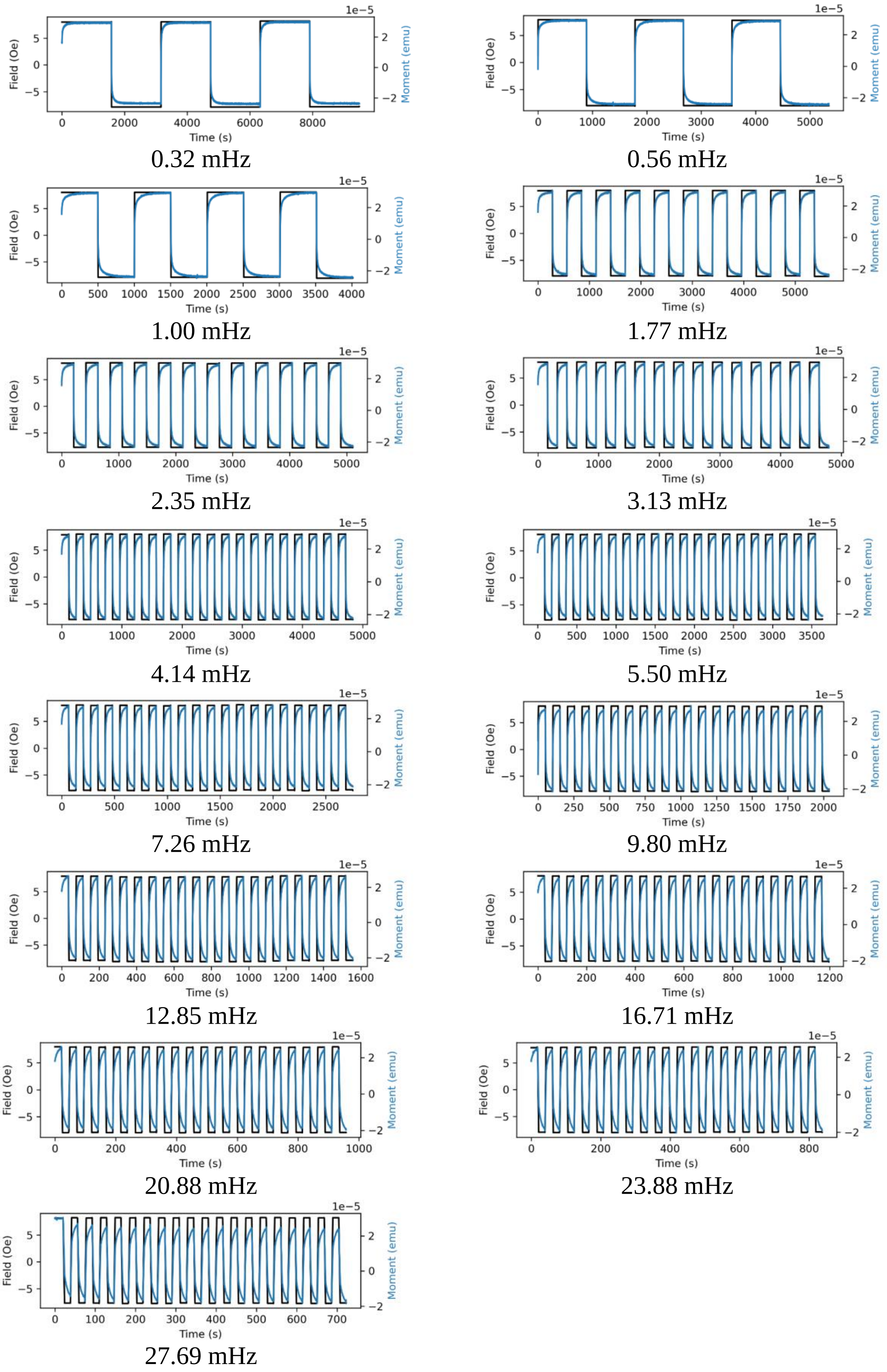


**Figure S120.** Raw waveform data and corresponding frequencies for **3-Dy·$C_6H_6$** at 44 K.

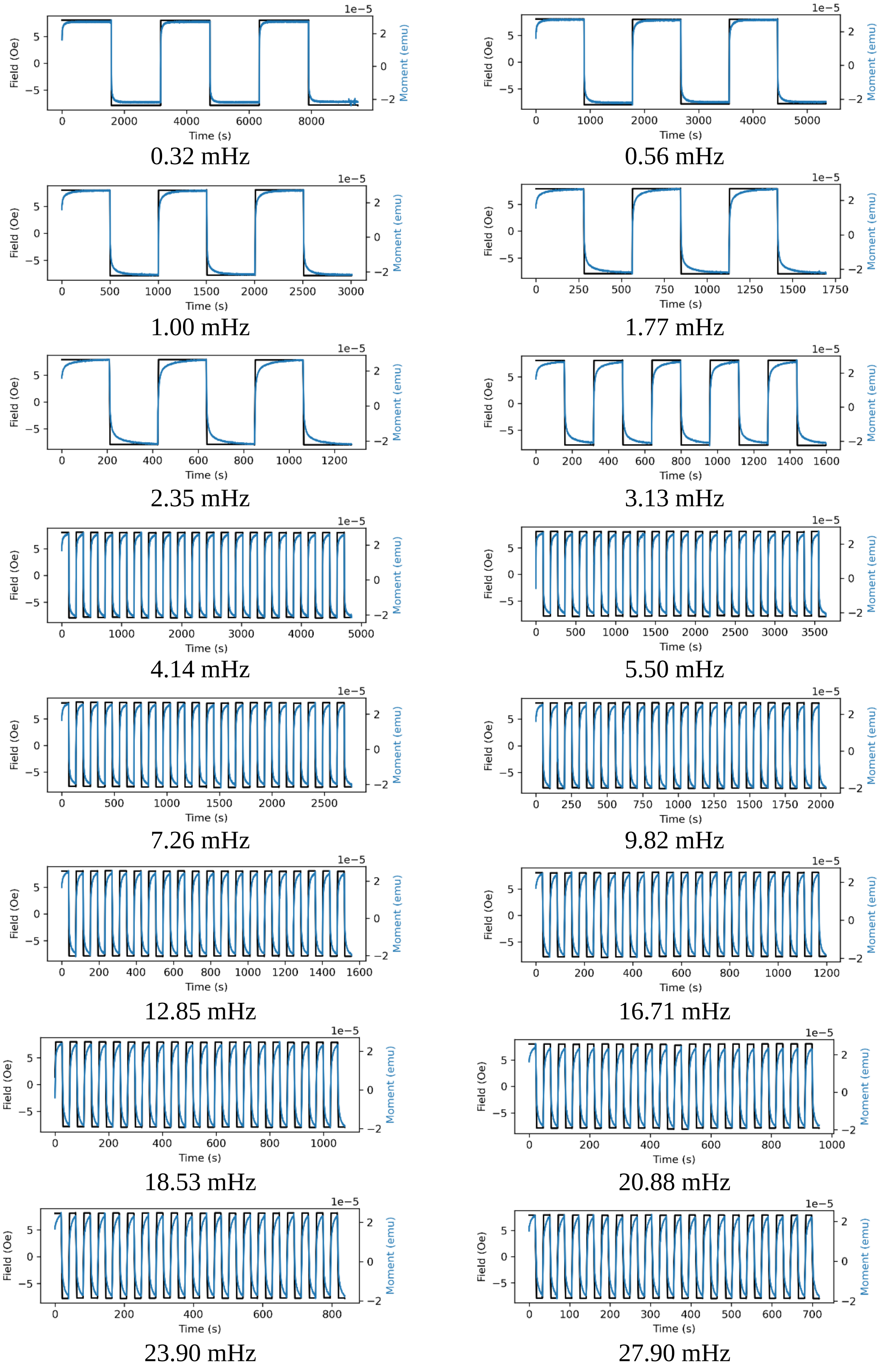


**Figure S121.** Raw waveform data and corresponding frequencies for **3-Dy·$C_6H_6$** at 48 K.

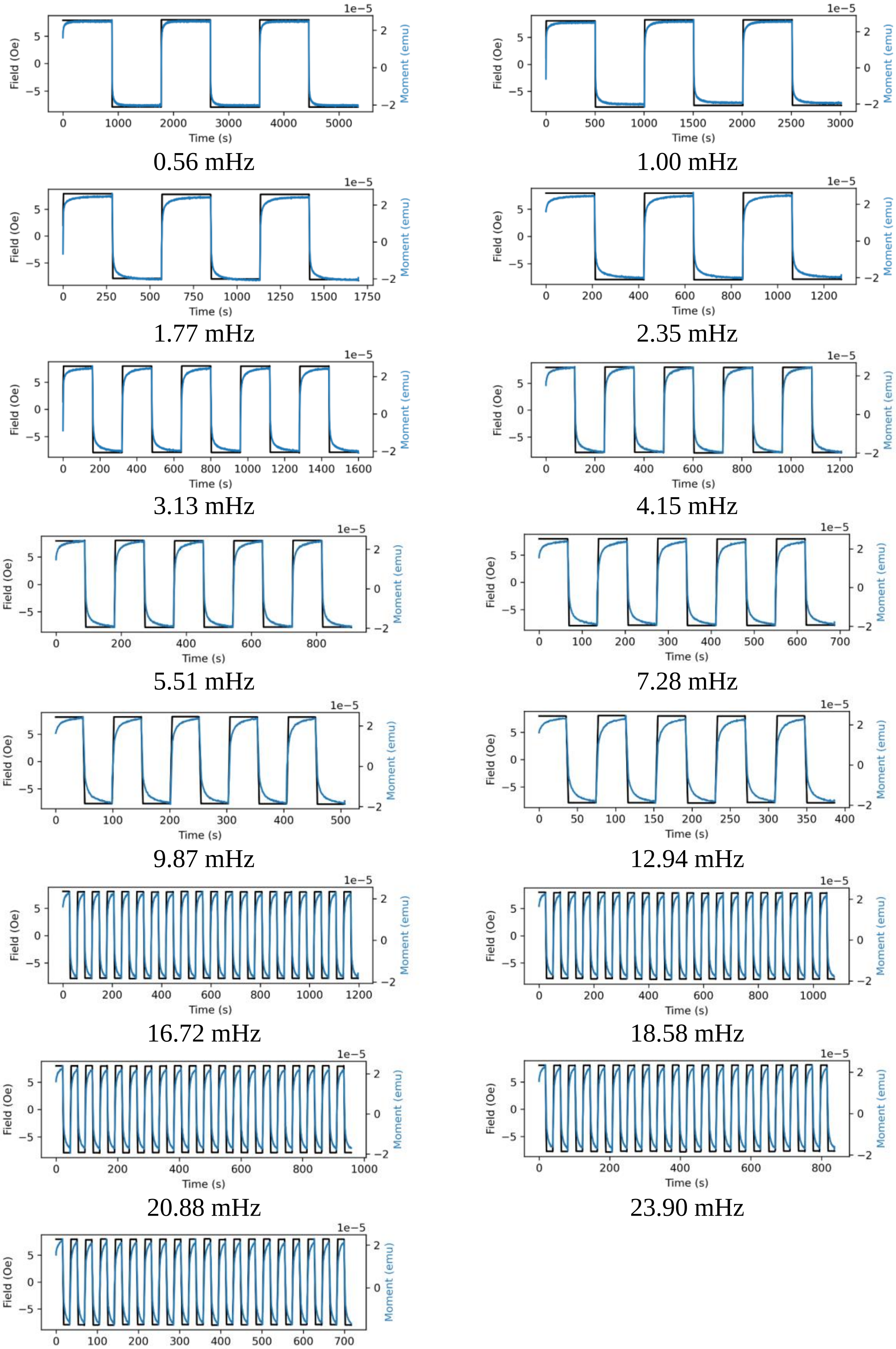


0.56 mHz | 1.00 mHz | 1.77 mHz | 2.35 mHz | 3.13 mHz | 4.15 mHz | 5.51 mHz | 7.28 mHz | 9.87 mHz | 12.94 mHz | 16.72 mHz | 18.58 mHz | 20.88 mHz | 23.90 mHz | 27.91 mHz

**Figure S122.** Raw waveform data and corresponding frequencies for **3-Dy·$C_6H_6$** at 52 K.

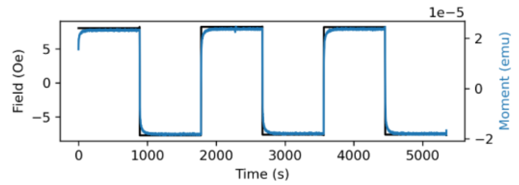


0.56 mHz

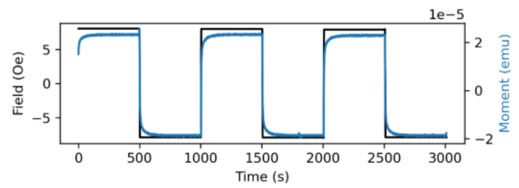


1.00 mHz

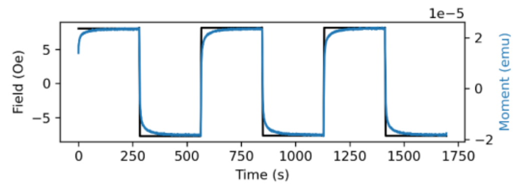


1.77 mHz

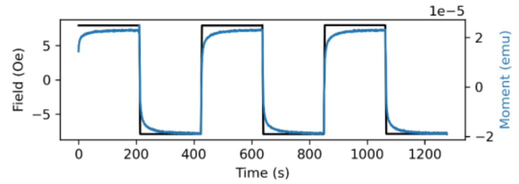


2.35 mHz

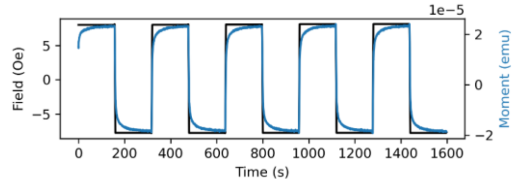


3.13 mHz

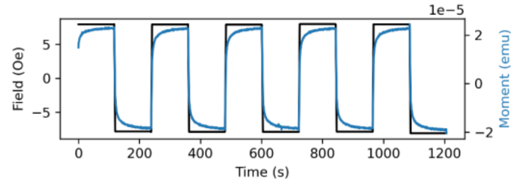


4.14 mHz

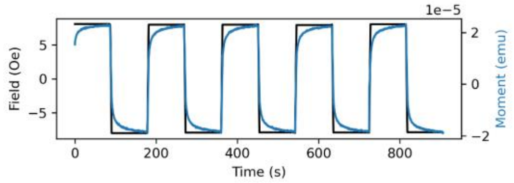


5.52 mHz

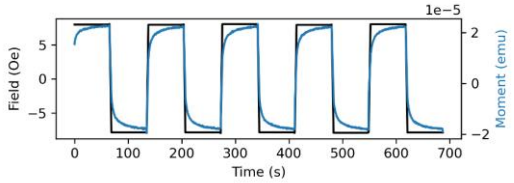


7.28 mHz

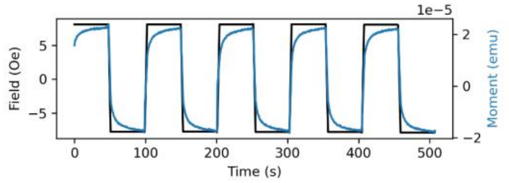


9.86 mHz

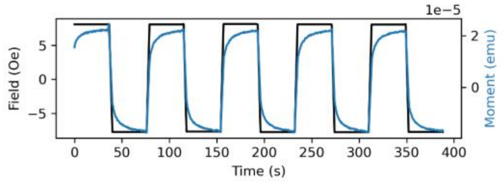


12.88 mHz

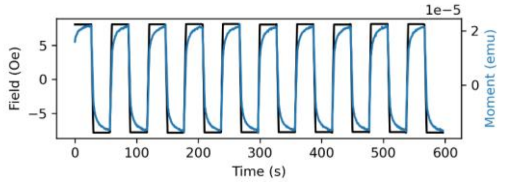


16.77 mHz

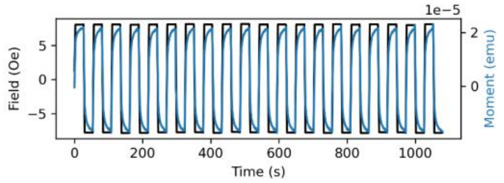


18.52 mHz

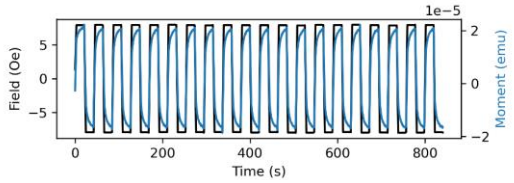


23.80 mHz

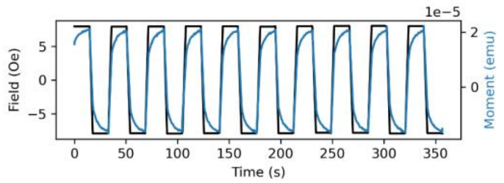


28.04 mHz

**Figure S123.** Raw waveform data and corresponding frequencies for **3-Dy·$C_6H_6$** at 56 K.

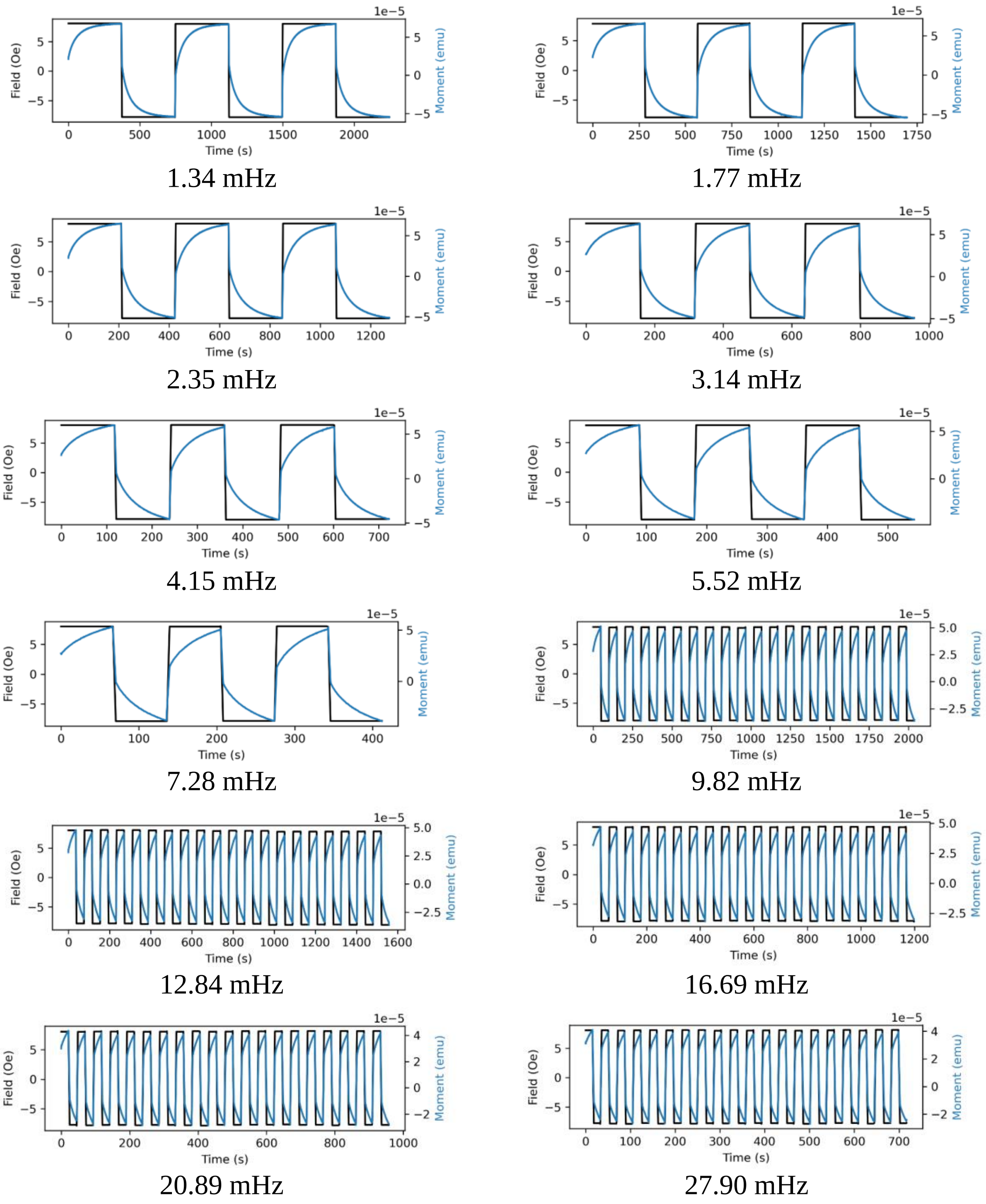


**Figure S124.** Raw waveform data and corresponding frequencies for **4-Dy** at 20 K.

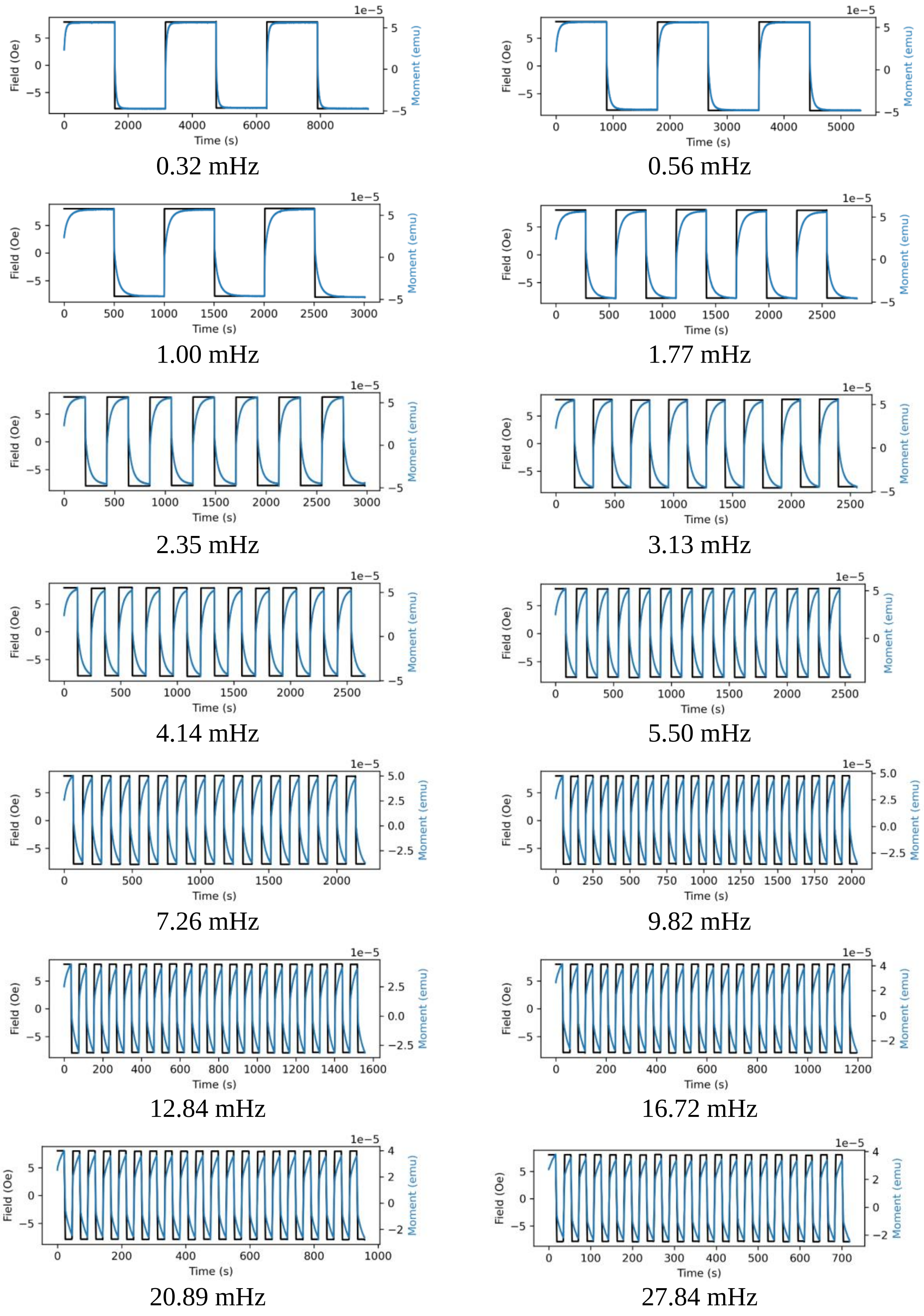


**Figure S125.** Raw waveform data and corresponding frequencies for **4-Dy** at 24 K.

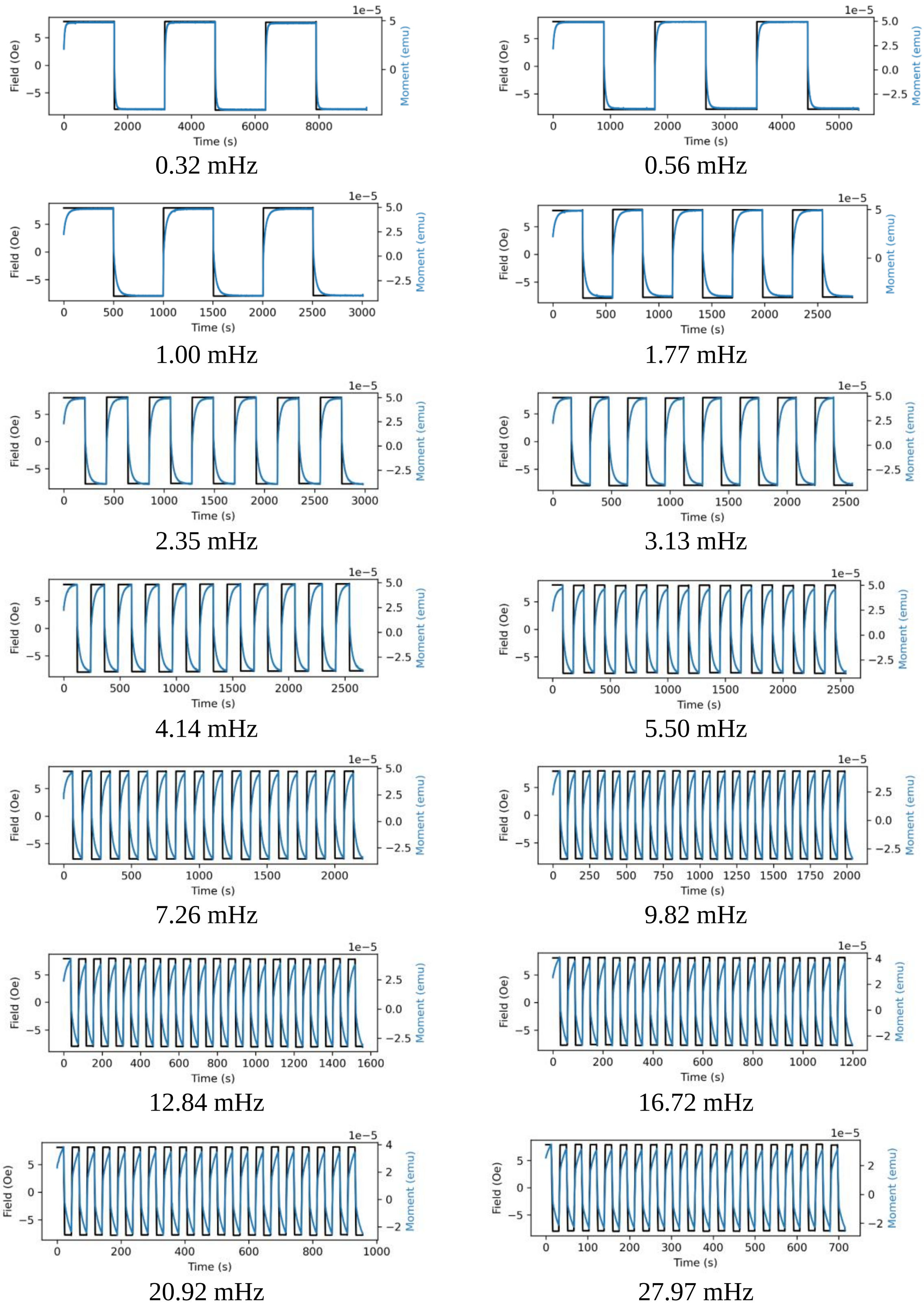


**Figure S126.** Raw waveform data and corresponding frequencies for **4-Dy** at 28 K.

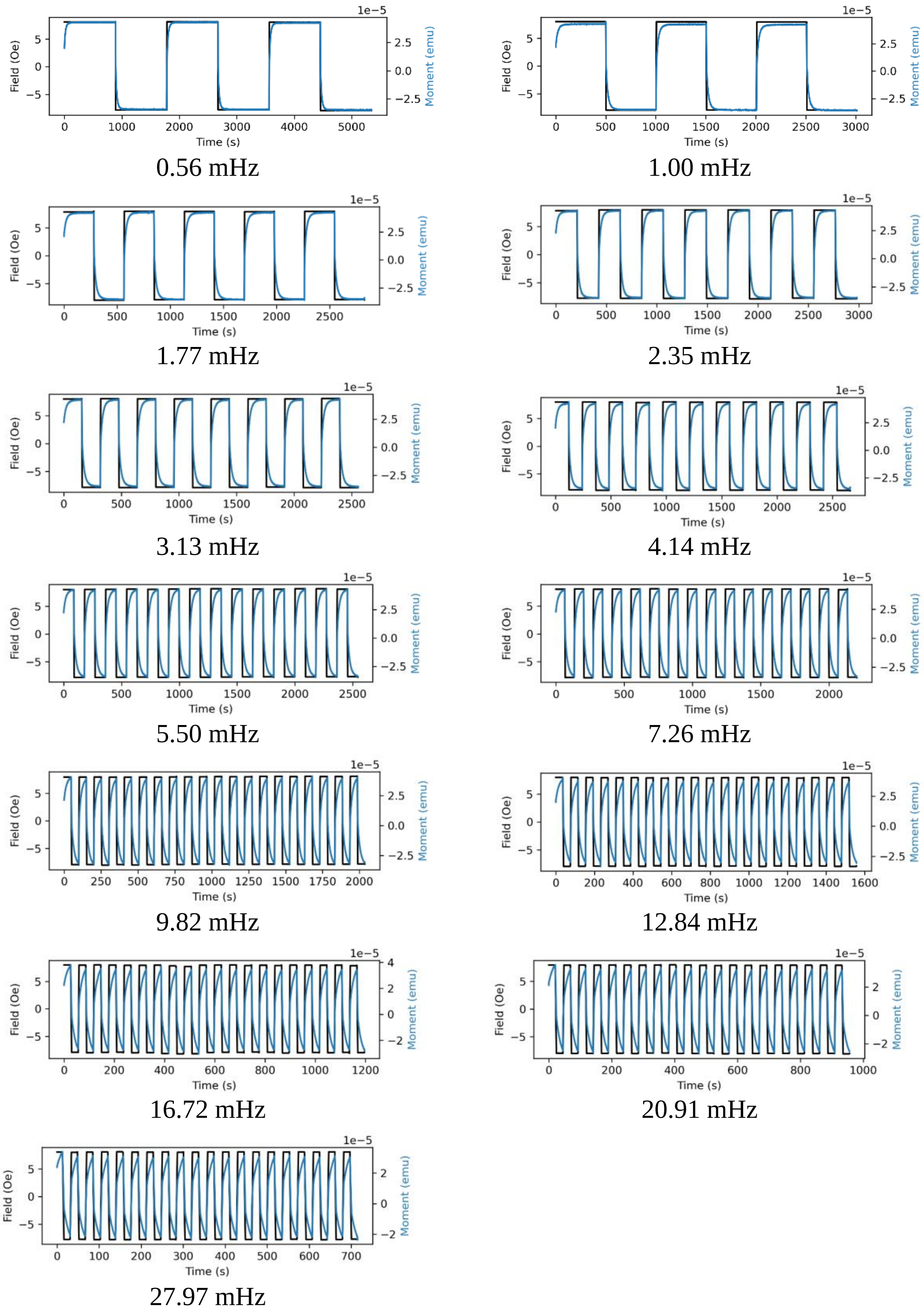


**Figure S127.** Raw waveform data and corresponding frequencies for **4-Dy** at 32 K.

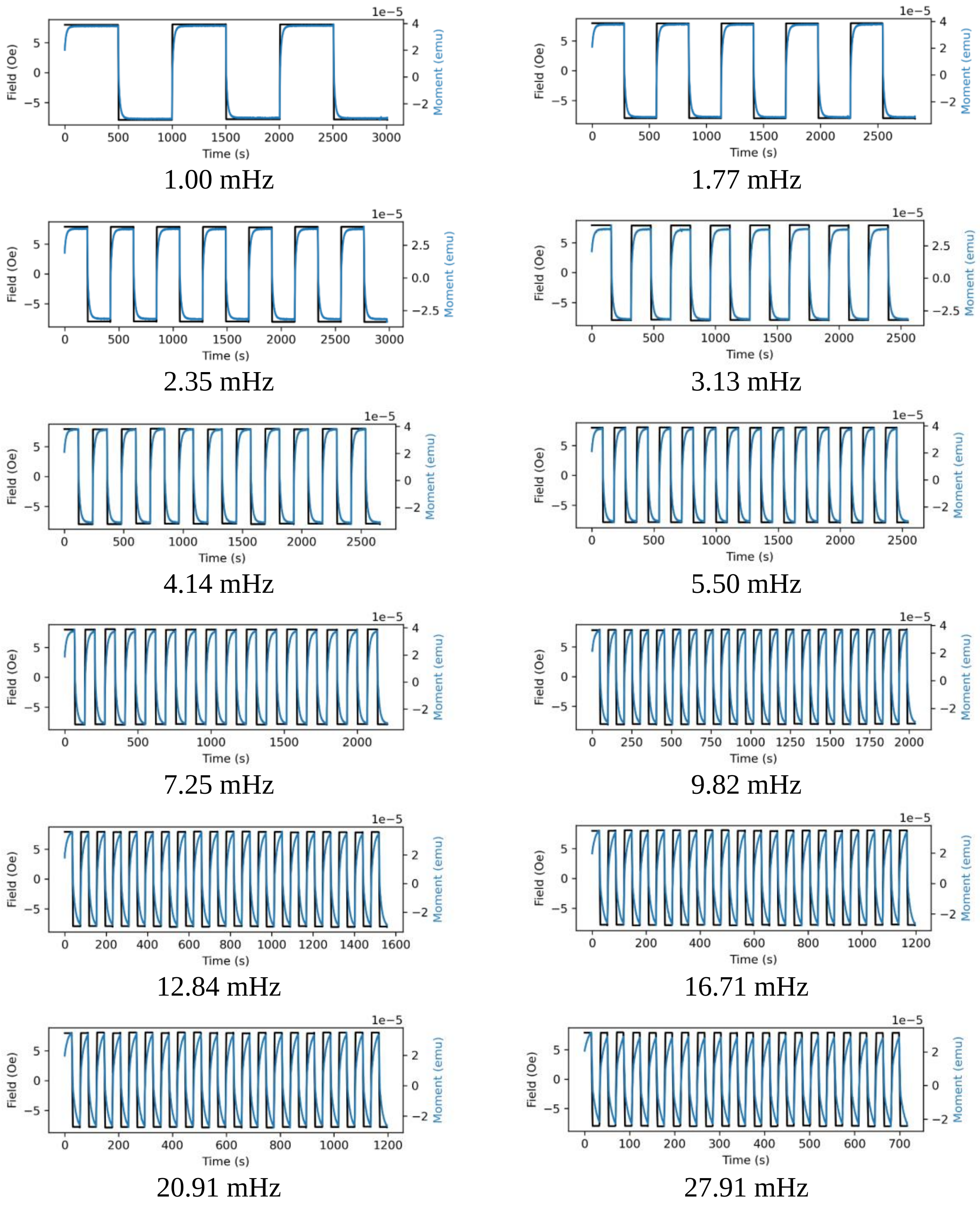


**Figure S128.** Raw waveform data and corresponding frequencies for **4-Dy** at 36 K.

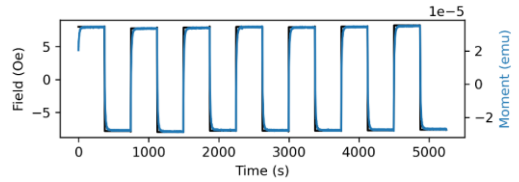


1.33 mHz

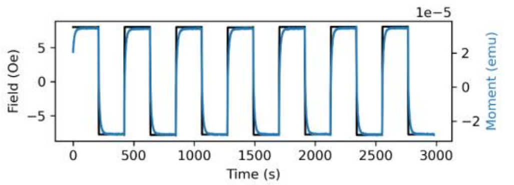


2.35 mHz

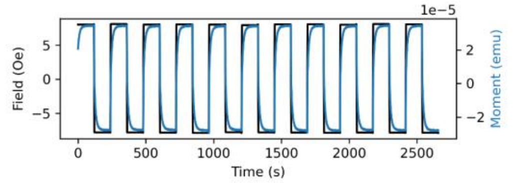


4.14 mHz

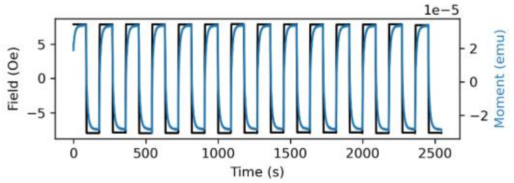


5.50 mHz

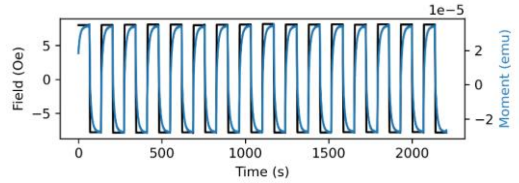


7.26 mHz

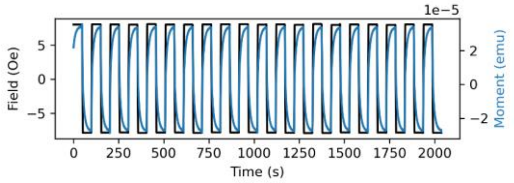


9.82 mHz

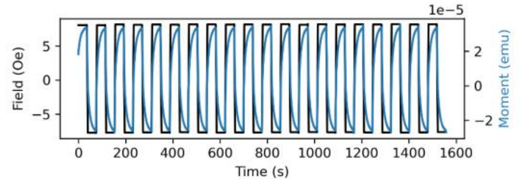


12.84 mHz

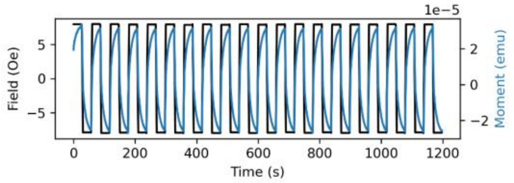


16.71 mHz

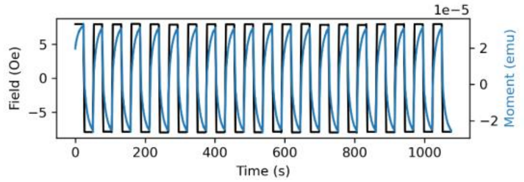


18.59 mHz

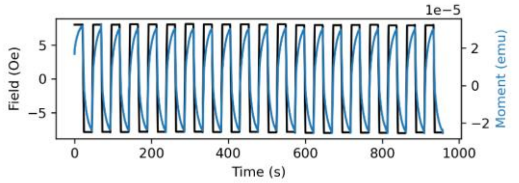


20.89 mHz

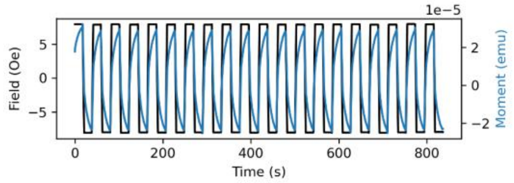


23.91 mHz

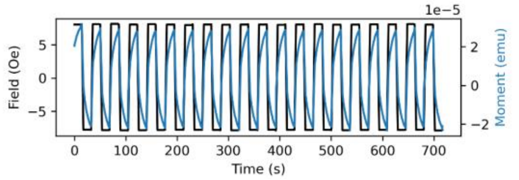


27.91 mHz

**Figure S129.** Raw waveform data and corresponding frequencies for **4-Dy** at 40 K.

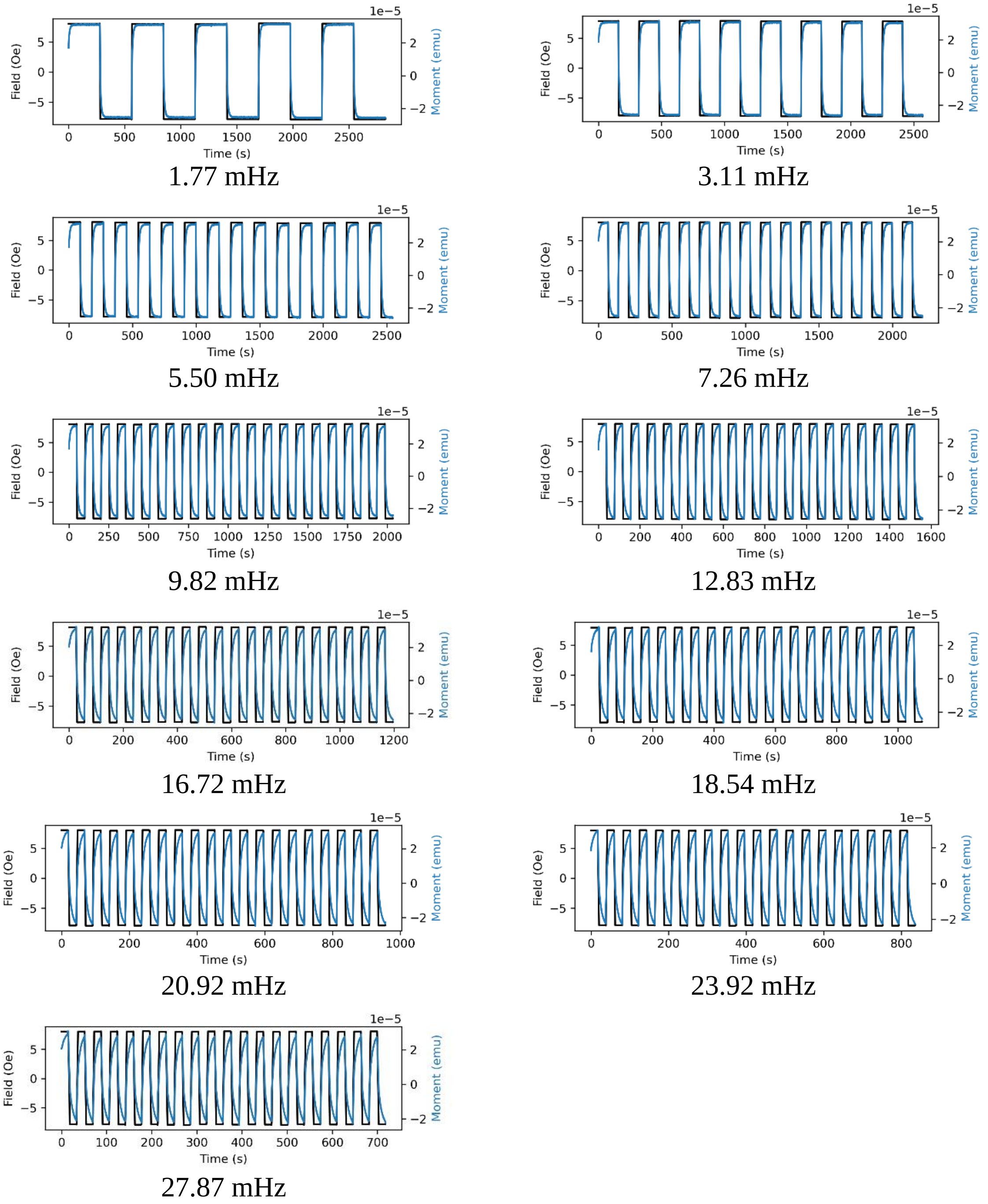


**Figure S130.** Raw waveform data and corresponding frequencies for **4-Dy** at 44 K.

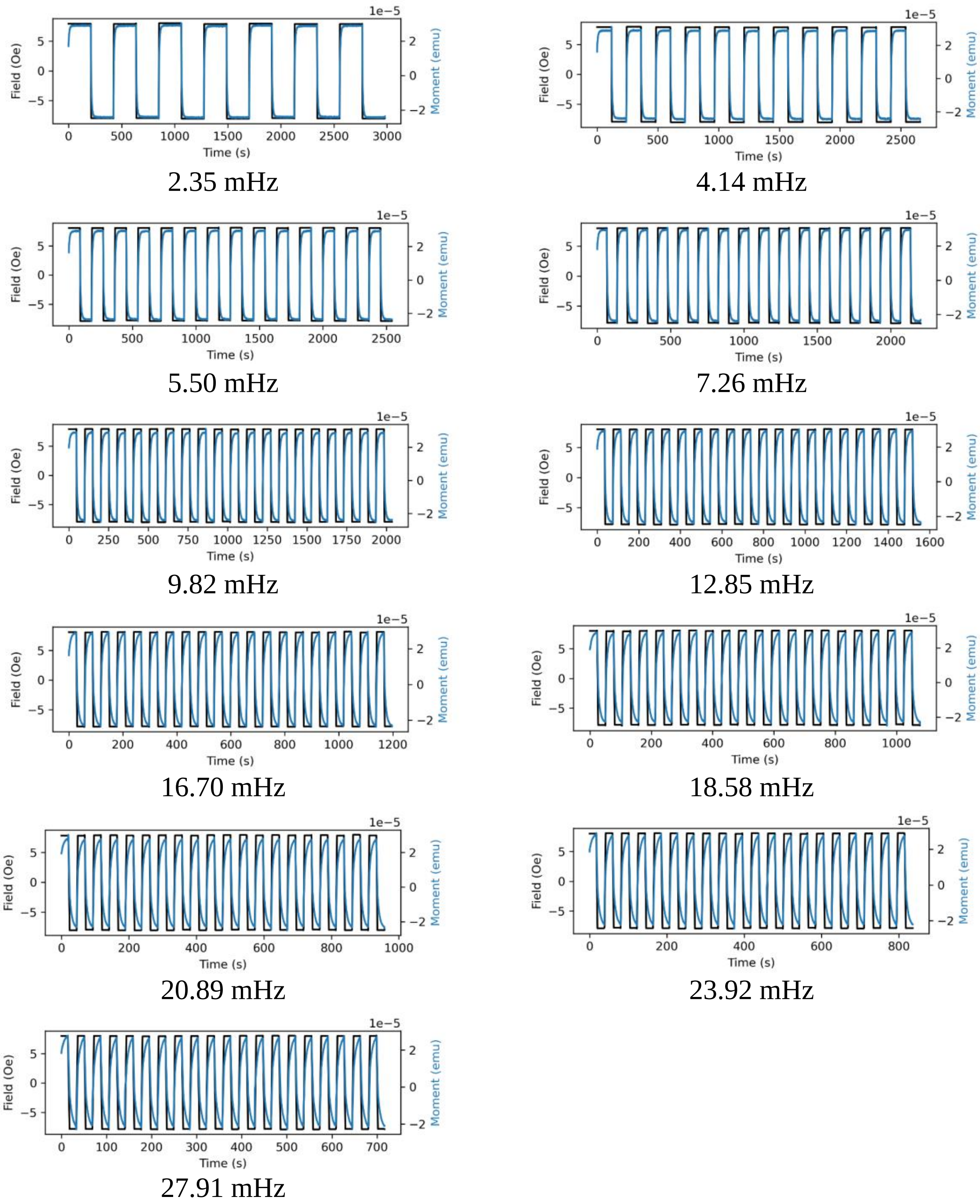


**Figure S131.** Raw waveform data and corresponding frequencies for **4-Dy** at 47 K.

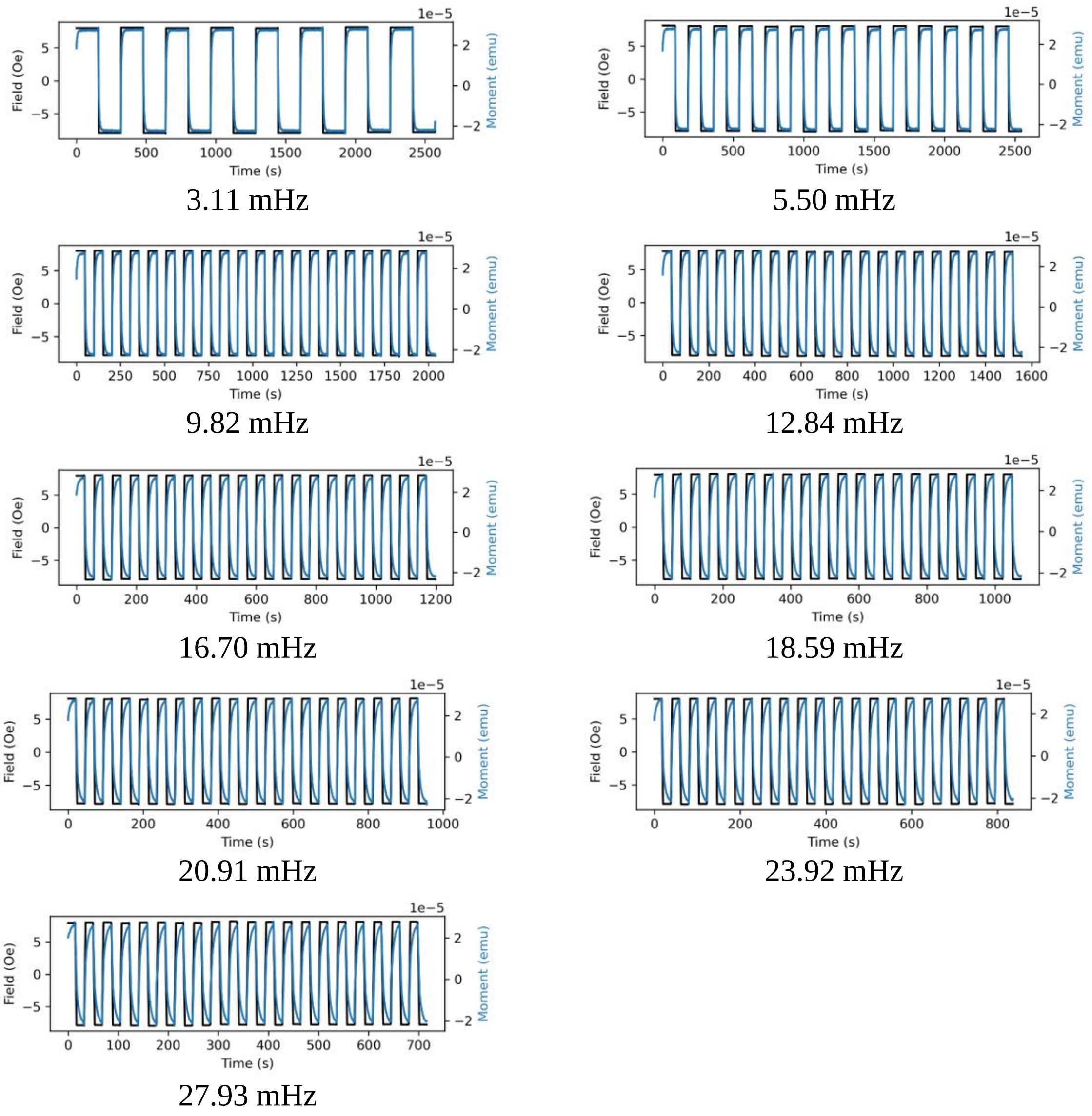


**Figure S132.** Raw waveform data and corresponding frequencies for **4-Dy** at 50 K.

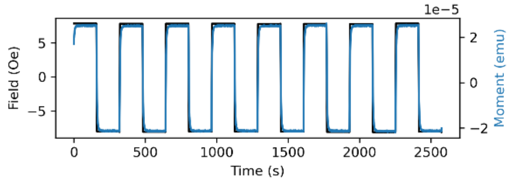


3.11 mHz

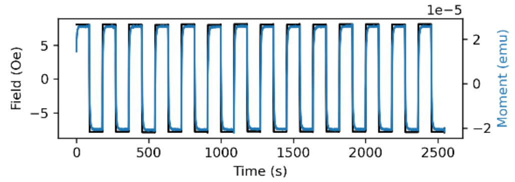


5.50 mHz

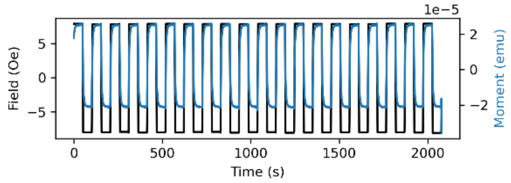


9.62 mHz

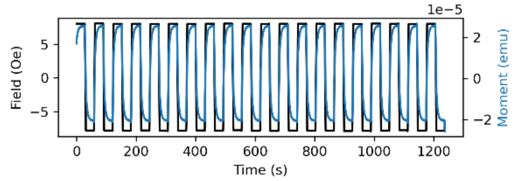


16.16 mHz

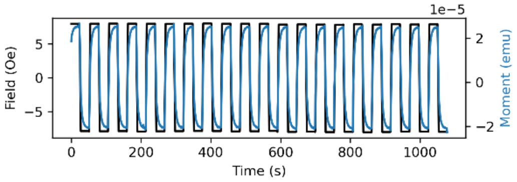


18.57 mHz

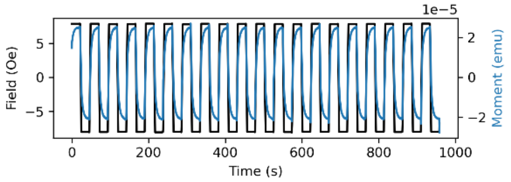


20.87 mHz

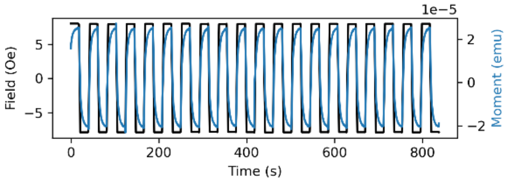


23.88 mHz

**Figure S133.** Raw waveform data and corresponding frequencies for **4-Dy** at 54 K.

**Table S22.** Extracted ac susceptibilities from waveform data for **3-Dy·$C_6H_6$**

| T | Frequency | χ' | χ" | ϕ | Drive Amplitude |
|---|---|---|---|---|---|
| (K) | (Hz) | (emu/Oe) | (emu/Oe) | (rad) | (Oe) |
| 32 | 0.00017988 | 4.53925039E–6 | 1.72097788E–7 | 0.0379 | 7.9297 |
| 32 | 0.00031596 | 4.46489554E–6 | 2.76257018E–7 | 0.0618 | 7.9058 |
| 32 | 0.00056120 | 4.34264809E–6 | 3.86758121E–7 | 0.0888 | 7.9576 |
| 32 | 0.00099610 | 4.15464233E–6 | 4.81340206E–7 | 0.1153 | 7.9234 |
| 32 | 0.00176779 | 3.93867379E–6 | 5.63465978E–7 | 0.1421 | 7.9258 |
| 32 | 0.00234889 | 3.82879659E–6 | 5.95755446E–7 | 0.1544 | 7.9428 |
| 32 | 0.00312523 | 3.70541594E–6 | 6.42155042E–7 | 0.1716 | 7.9228 |
| 32 | 0.00413451 | 3.57138311E–6 | 6.80476979E–7 | 0.1883 | 7.9317 |
| 32 | 0.00549960 | 3.41824541E–6 | 7.10136281E–7 | 0.2048 | 7.9235 |
| 32 | 0.00725221 | 3.25245353E–6 | 7.22776430E–7 | 0.2187 | 7.9100 |
| 32 | 0.00981976 | 3.06138367E–6 | 7.04827826E–7 | 0.2263 | 7.9099 |
| 32 | 0.01284936 | 2.88623717E–6 | 6.66163360E–7 | 0.2268 | 7.9290 |
| 32 | 0.01672213 | 2.74811906E–6 | 5.93185493E–7 | 0.2126 | 7.9109 |
| 32 | 0.02084303 | 2.63322620E–6 | 5.34066369E–7 | 0.2001 | 7.8794 |
| 32 | 0.02388112 | 2.57328108E–6 | 4.87141285E–7 | 0.1871 | 7.8122 |
| 32 | 0.02789211 | 2.51070142E–6 | 4.42028388E–7 | 0.1743 | 7.9104 |
| 40 | 0.00017988 | 3.66278915E–6 | 7.93206146E–8 | 0.0217 | 7.9078 |
| 40 | 0.00031588 | 3.63654279E–6 | 1.34263678E–7 | 0.0369 | 7.9654 |
| 40 | 0.00056151 | 3.58773836E–6 | 2.10448022E–7 | 0.0586 | 7.9326 |
| 40 | 0.00099620 | 3.49687947E–6 | 3.01798945E–7 | 0.0861 | 7.9174 |
| 40 | 0.00176631 | 3.34823750E–6 | 3.81612947E–7 | 0.1135 | 7.9206 |
| 40 | 0.00234786 | 3.26175246E–6 | 4.11225235E–7 | 0.1254 | 7.9360 |
| 40 | 0.00312646 | 3.17944819E–6 | 4.38831832E–7 | 0.1372 | 7.9305 |
| 40 | 0.00413221 | 3.09094834E–6 | 4.65239150E–7 | 0.1494 | 7.9152 |
| 40 | 0.00549978 | 2.99544750E–6 | 4.94919840E–7 | 0.1637 | 7.9237 |
| 40 | 0.00725645 | 2.89037986E–6 | 5.22898264E–7 | 0.1790 | 7.9089 |
| 40 | 0.00982056 | 2.76109744E–6 | 5.45724022E–7 | 0.1951 | 7.9195 |
| 40 | 0.01284767 | 2.63937411E–6 | 5.44484789E–7 | 0.2034 | 7.8831 |
| 40 | 0.01671004 | 2.49992565E–6 | 5.33163710E–7 | 0.2101 | 7.9050 |
| 40 | 0.02090647 | 2.38346350E–6 | 5.13135846E–7 | 0.2121 | 7.8906 |
| 40 | 0.02386784 | 2.31387452E–6 | 4.84783905E–7 | 0.2065 | 7.8915 |
| 40 | 0.02790579 | 2.25022114E–6 | 4.42539754E–7 | 0.1942 | 7.8316 |
| 44 | 0.00031598 | 3.33074844E–6 | 9.59290121E–8 | 0.0288 | 7.9609 |
| 44 | 0.00056119 | 3.29510276E–6 | 1.55640243E–7 | 0.0472 | 7.9341 |
| 44 | 0.00099680 | 3.22293450E–6 | 2.32949064E–7 | 0.0722 | 7.9673 |
| 44 | 0.00176778 | 3.11386255E–6 | 3.12506128E–7 | 0.1000 | 7.9295 |
| 44 | 0.00234898 | 3.04152627E–6 | 3.44638889E–7 | 0.1128 | 7.9256 |
| 44 | 0.00312685 | 2.97072269E–6 | 3.70946572E–7 | 0.1242 | 7.9162 |
| 44 | 0.00413514 | 2.89448352E–6 | 3.93659791E–7 | 0.1352 | 7.9292 |
| 44 | 0.00549986 | 2.81020294E–6 | 4.15452876E–7 | 0.1468 | 7.9317 |

| | | | | | |
|---|---|---|---|---|---|
| 44 | 0.00725553 | 2.73148707E–6 | 4.35140449E–7 | 0.1580 | 7.9078 |
| 44 | 0.00980481 | 2.62639795E–6 | 4.65981273E–7 | 0.1756 | 7.9007 |
| 44 | 0.01284803 | 2.53065815E–6 | 4.61906808E–7 | 0.1805 | 7.8619 |
| 44 | 0.01671433 | 2.40512429E–6 | 4.80892377E–7 | 0.1973 | 7.9008 |
| 44 | 0.02088335 | 2.30189684E–6 | 4.65274172E–7 | 0.1994 | 7.8986 |
| 44 | 0.02388048 | 2.23617858E–6 | 4.53443906E–7 | 0.2001 | 7.8915 |
| 44 | 0.02768634 | 2.18371234E–6 | 4.28162669E–7 | 0.1936 | 7.8767 |
| 48 | 0.00031597 | 3.05990911E–6 | 6.78487733E–8 | 0.0222 | 7.9398 |
| 48 | 0.00056151 | 3.03686136E–6 | 1.12667036E–7 | 0.0371 | 7.9498 |
| 48 | 0.00099704 | 2.99445132E–6 | 1.75835419E–7 | 0.0587 | 7.9179 |
| 48 | 0.00177063 | 2.91898040E–6 | 2.43160345E–7 | 0.0831 | 7.9247 |
| 48 | 0.00235385 | 2.85750264E–6 | 2.71716562E–7 | 0.0948 | 7.9242 |
| 48 | 0.00313041 | 2.79571145E–6 | 3.07028334E–7 | 0.1094 | 7.9137 |
| 48 | 0.00413504 | 2.72008831E–6 | 3.32662987E–7 | 0.1217 | 7.9195 |
| 48 | 0.00549509 | 2.65166830E–6 | 3.52365481E–7 | 0.1321 | 7.9222 |
| 48 | 0.00725500 | 2.58008606E–6 | 3.71769848E–7 | 0.1431 | 7.9131 |
| 48 | 0.00982210 | 2.49861271E–6 | 3.92955960E–7 | 0.1560 | 7.9134 |
| 48 | 0.01284782 | 2.41128630E–6 | 4.04103000E–7 | 0.1660 | 7.8850 |
| 48 | 0.01670994 | 2.31742477E–6 | 4.17636387E–7 | 0.1783 | 7.9130 |
| 48 | 0.01853091 | 2.27447518E–6 | 4.24222710E–7 | 0.1844 | 7.8978 |
| 48 | 0.02088275 | 2.22849713E–6 | 4.17208128E–7 | 0.1851 | 7.9054 |
| 48 | 0.02389518 | 2.17764656E–6 | 4.16416968E–7 | 0.1889 | 7.8958 |
| 48 | 0.02790458 | 2.10463623E–6 | 4.08427221E–7 | 0.1917 | 7.8612 |
| 52 | 0.00056141 | 2.82577697E–6 | 7.90297924E–8 | 0.0280 | 7.9311 |
| 52 | 0.00099612 | 2.79841221E–6 | 1.29563496E–7 | 0.0463 | 7.9246 |
| 52 | 0.00176631 | 2.73795593E–6 | 1.87564611E–7 | 0.0684 | 7.9209 |
| 52 | 0.00235266 | 2.69390914E–6 | 2.16312200E–7 | 0.0801 | 7.9127 |
| 52 | 0.00312544 | 2.64452460E–6 | 2.49029071E–7 | 0.0939 | 7.9221 |
| 52 | 0.00414559 | 2.58767675E–6 | 2.68346359E–7 | 0.1033 | 7.9069 |
| 52 | 0.00551294 | 2.52910615E–6 | 2.86828003E–7 | 0.1129 | 7.9004 |
| 52 | 0.00728023 | 2.46823937E–6 | 3.03289210E–7 | 0.1223 | 7.9302 |
| 52 | 0.00986631 | 2.40239113E–6 | 3.18050674E–7 | 0.1316 | 7.8615 |
| 52 | 0.01294027 | 2.33057926E–6 | 3.41763950E–7 | 0.1456 | 7.9172 |
| 52 | 0.01671545 | 2.24440125E–6 | 3.53675935E–7 | 0.1563 | 7.9130 |
| 52 | 0.01857546 | 2.21237940E–6 | 3.59990877E–7 | 0.1613 | 7.9149 |
| 52 | 0.02088412 | 2.16642183E–6 | 3.66481744E–7 | 0.1676 | 7.8779 |
| 52 | 0.02389668 | 2.12597266E–6 | 3.67579079E–7 | 0.1712 | 7.8910 |
| 52 | 0.02791031 | 2.06296445E–6 | 3.62862763E–7 | 0.1741 | 7.8810 |
| 56 | 0.00056146 | 2.62741524E–6 | 5.05104672E–8 | 0.0192 | 7.8892 |
| 56 | 0.00099670 | 2.62051549E–6 | 8.75626573E–8 | 0.0334 | 7.9180 |
| 56 | 0.00176988 | 2.58338985E–6 | 1.37512304E–7 | 0.0532 | 7.9099 |
| 56 | 0.00235079 | 2.55794579E–6 | 1.59799471E–7 | 0.0624 | 7.9223 |
| 56 | 0.00313018 | 2.51555337E–6 | 1.93758966E–7 | 0.0769 | 7.9232 |
| 56 | 0.00414441 | 2.47599750E–6 | 2.16112418E–7 | 0.0871 | 7.9362 |

| | | | | | |
|---|---|---|---|---|---|
| 56 | 0.00551923 | 2.42665043E–6 | 2.31928765E–7 | 0.0953 | 7.9109 |
| 56 | 0.00728025 | 2.37661698E–6 | 2.51222761E–7 | 0.1053 | 7.8918 |
| 56 | 0.00985959 | 2.31338075E–6 | 2.66820203E–7 | 0.1148 | 7.9019 |
| 56 | 0.01288273 | 2.26113861E–6 | 2.88978162E–7 | 0.1271 | 7.8960 |
| 56 | 0.01676778 | 2.18738068E–6 | 3.00174898E–7 | 0.1364 | 7.9106 |
| 56 | 0.01852284 | 2.16207213E–6 | 3.04145690E–7 | 0.1398 | 7.9254 |
| 56 | 0.02379658 | 2.09197300E–6 | 3.15016523E–7 | 0.1495 | 7.8999 |
| 56 | 0.02804043 | 2.04245371E–6 | 3.19791484E–7 | 0.1553 | 7.9162 |

**Table S23.** Extracted ac susceptibilities from waveform data for **4-Dy**

| T | Frequency | χ' | χ'' | ϕ | Drive Amplitude |
|---|---|---|---|---|---|
| (K) | (Hz) | (emu/Oe) | (emu/Oe) | (rad) | (Oe) |
| 20 | 0.00133534 | 6.72236765E–6 | 1.56171128E–6 | 0.2283 | 7.9294 |
| 20 | 0.00176958 | 6.32412831E–6 | 1.70846337E–6 | 0.2639 | 7.9025 |
| 20 | 0.00235415 | 5.86752816E–6 | 1.77304791E–6 | 0.2935 | 7.9227 |
| 20 | 0.00313690 | 5.39348881E–6 | 1.73615988E–6 | 0.3114 | 7.9249 |
| 20 | 0.00415265 | 4.96564344E–6 | 1.63427049E–6 | 0.3179 | 7.9530 |
| 20 | 0.00552051 | 4.58155567E–6 | 1.46900414E–6 | 0.3103 | 7.8746 |
| 20 | 0.00728498 | 4.22935992E–6 | 1.30949554E–6 | 0.3003 | 7.9074 |
| 20 | 0.00982068 | 3.89070358E–6 | 1.13566255E–6 | 0.2840 | 7.8969 |
| 20 | 0.01284063 | 3.70260595E–6 | 9.57478942E–7 | 0.2531 | 7.9279 |
| 20 | 0.01669423 | 3.57346099E–6 | 7.74526633E–7 | 0.2134 | 7.9121 |
| 20 | 0.02089429 | 3.47444402E–6 | 6.45036026E–7 | 0.1836 | 7.8913 |
| 20 | 0.02790327 | 3.37737974E–6 | 4.94914841E–7 | 0.1455 | 7.8777 |
| 24 | 0.00031600 | 6.46521507E–6 | 2.91323419E–7 | 0.0450 | 7.9438 |
| 24 | 0.00056155 | 6.38361214E–6 | 4.96287957E–7 | 0.0776 | 7.9750 |
| 24 | 0.00099702 | 6.21630478E–6 | 8.04384144E–7 | 0.1287 | 7.9597 |
| 24 | 0.00176909 | 5.82295655E–6 | 1.21674837E–6 | 0.2060 | 7.9215 |
| 24 | 0.00235021 | 5.50588434E–6 | 1.40312567E–6 | 0.2495 | 7.9521 |
| 24 | 0.00312976 | 5.11420840E–6 | 1.52200319E–6 | 0.2893 | 7.9333 |
| 24 | 0.00413787 | 4.70807693E–6 | 1.55694765E–6 | 0.3194 | 7.9264 |
| 24 | 0.00550303 | 4.27095257E–6 | 1.51636952E–6 | 0.3412 | 7.9175 |
| 24 | 0.00725603 | 3.90711602E–6 | 1.38560770E–6 | 0.3408 | 7.9008 |
| 24 | 0.00982449 | 3.54728849E–6 | 1.21539640E–6 | 0.3301 | 7.9204 |
| 24 | 0.01284398 | 3.30812208E–6 | 1.02107967E–6 | 0.2994 | 7.8942 |
| 24 | 0.01672458 | 3.13931130E–6 | 8.53858691E–7 | 0.2656 | 7.8956 |
| 24 | 0.02088611 | 3.02267506E–6 | 7.09325067E–7 | 0.2305 | 7.8704 |
| 24 | 0.02784151 | 2.92039100E–6 | 5.39840530E–7 | 0.1828 | 7.8523 |
| 28 | 0.00031594 | 5.59364133E–6 | 1.65456790E–7 | 0.0296 | 7.9398 |
| 28 | 0.00056158 | 5.56936619E–6 | 2.84913627E–7 | 0.0511 | 7.9244 |
| 28 | 0.00099722 | 5.48959876E–6 | 4.78906175E–7 | 0.0870 | 7.9219 |
| 28 | 0.00176859 | 5.30295628E–6 | 7.89517901E–7 | 0.1478 | 7.9156 |

| | | | | | |
|---|---|---|---|---|---|
| 28 | 0.00234894 | 5.12824270E–6 | 9.70962452E–7 | 0.1871 | 7.9308 |
| 28 | 0.00312912 | 4.88201299E–6 | 1.14032024E–6 | 0.2295 | 7.8980 |
| 28 | 0.00413667 | 4.57806314E–6 | 1.27553686E–6 | 0.2717 | 7.9031 |
| 28 | 0.00550011 | 4.20859161E–6 | 1.33261920E–6 | 0.3067 | 7.9275 |
| 28 | 0.00725636 | 3.84039918E–6 | 1.32461768E–6 | 0.3321 | 7.9210 |
| 28 | 0.00982157 | 3.46313718E–6 | 1.21376144E–6 | 0.3371 | 7.8826 |
| 28 | 0.01284167 | 3.16539245E–6 | 1.08865902E–6 | 0.3313 | 7.8897 |
| 28 | 0.01670236 | 2.92204376E–6 | 9.42011703E–7 | 0.3119 | 7.8937 |
| 28 | 0.02091744 | 2.76948264E–6 | 7.93685407E–7 | 0.2791 | 7.8698 |
| 28 | 0.02797224 | 2.62844304E–6 | 6.19502970E–7 | 0.2315 | 7.8754 |
| 32 | 0.00056148 | 4.88048815E–6 | 1.72578143E–7 | 0.0353 | 7.9261 |
| 32 | 0.00099671 | 4.85500306E–6 | 2.90272343E–7 | 0.0597 | 7.9578 |
| 32 | 0.00176829 | 4.76293857E–6 | 4.97676874E–7 | 0.1041 | 7.9139 |
| 32 | 0.00234906 | 4.68282900E–6 | 6.39473945E–7 | 0.1357 | 7.8629 |
| 32 | 0.00312906 | 4.52464522E–6 | 7.95922756E–7 | 0.1741 | 7.9098 |
| 32 | 0.00413526 | 4.34656378E–6 | 9.41083078E–7 | 0.2132 | 7.9166 |
| 32 | 0.00550263 | 4.09710430E–6 | 1.08160595E–6 | 0.2581 | 7.9090 |
| 32 | 0.00725865 | 3.79310530E–6 | 1.14414640E–6 | 0.2930 | 7.9242 |
| 32 | 0.00982065 | 3.43610054E–6 | 1.14126730E–6 | 0.3207 | 7.9027 |
| 32 | 0.01284043 | 3.11173183E–6 | 1.09254092E–6 | 0.3377 | 7.8891 |
| 32 | 0.01671617 | 2.83326051E–6 | 9.82507051E–7 | 0.3338 | 7.8955 |
| 32 | 0.02091013 | 2.64877593E–6 | 8.54769851E–7 | 0.3122 | 7.8941 |
| 32 | 0.02797323 | 2.46994323E–6 | 6.83320848E–7 | 0.2699 | 7.8575 |
| 36 | 0.00099732 | 4.32731290E–6 | 1.85285281E–7 | 0.0428 | 7.9395 |
| 36 | 0.00176926 | 4.28753971E–6 | 3.19100951E–7 | 0.0743 | 7.9485 |
| 36 | 0.00235043 | 4.25662845E–6 | 4.20507646E–7 | 0.0985 | 7.9205 |
| 36 | 0.00312871 | 4.17548738E–6 | 5.29588855E–7 | 0.1262 | 7.9226 |
| 36 | 0.00413813 | 4.06649486E–6 | 6.68429857E–7 | 0.1629 | 7.9273 |
| 36 | 0.00550361 | 3.91011569E–6 | 7.96505853E–7 | 0.2010 | 7.9360 |
| 36 | 0.00725295 | 3.70057958E–6 | 9.07093044E–7 | 0.2404 | 7.9229 |
| 36 | 0.00982085 | 3.40716052E–6 | 9.80560520E–7 | 0.2802 | 7.9199 |
| 36 | 0.01284069 | 3.11295933E–6 | 9.95935086E–7 | 0.3096 | 7.9217 |
| 36 | 0.01671436 | 2.82511232E–6 | 9.46644735E–7 | 0.3233 | 7.9121 |
| 36 | 0.02090895 | 2.60308922E–6 | 8.79529447E–7 | 0.3258 | 7.8704 |
| 36 | 0.02790694 | 2.38142587E–6 | 7.20520263E–7 | 0.2938 | 7.8635 |
| 40 | 0.00133433 | 3.90933180E–6 | 1.64617936E–7 | 0.0421 | 7.8945 |
| 40 | 0.00235036 | 3.87020559E–6 | 2.75288971E–7 | 0.0710 | 7.9203 |
| 40 | 0.00413897 | 3.76813349E–6 | 4.62019988E–7 | 0.1220 | 7.9268 |
| 40 | 0.00550275 | 3.66796433E–6 | 5.78061620E–7 | 0.1563 | 7.9213 |
| 40 | 0.00725578 | 3.52929189E–6 | 6.88870076E–7 | 0.1928 | 7.9067 |
| 40 | 0.00981805 | 3.32393590E–6 | 7.93775929E–7 | 0.2344 | 7.9246 |
| 40 | 0.01284418 | 3.08710064E–6 | 8.50188840E–7 | 0.2687 | 7.9143 |
| 40 | 0.01670879 | 2.82652039E–6 | 8.73387070E–7 | 0.2997 | 7.8756 |
| 40 | 0.01858725 | 2.71660869E–6 | 8.70391841E–7 | 0.3101 | 7.9007 |

| | | | | | |
|---|---|---|---|---|---|
| 40 | 0.02089122 | 2.59775485E–6 | 8.53312078E–7 | 0.3174 | 7.8498 |
| 40 | 0.02391048 | 2.48324294E–6 | 8.03040112E–7 | 0.3128 | 7.8905 |
| 40 | 0.02790930 | 2.35804476E–6 | 7.34872831E–7 | 0.3021 | 7.8393 |
| 44 | 0.00176910 | 3.55729420E–6 | 1.49001392E–7 | 0.0419 | 7.9196 |
| 44 | 0.00310992 | 3.51409072E–6 | 2.53371448E–7 | 0.0720 | 7.9241 |
| 44 | 0.00550070 | 3.42348761E–6 | 4.23004584E–7 | 0.1229 | 7.9196 |
| 44 | 0.00725869 | 3.33457719E–6 | 5.17873057E–7 | 0.1541 | 7.9081 |
| 44 | 0.00982234 | 3.19553291E–6 | 6.31506695E–7 | 0.1951 | 7.9195 |
| 44 | 0.01283382 | 3.02688622E–6 | 7.16379218E–7 | 0.2324 | 7.8889 |
| 44 | 0.01672176 | 2.81811814E–6 | 7.56843166E–7 | 0.2624 | 7.9244 |
| 44 | 0.01854498 | 2.71935797E–6 | 7.76030723E–7 | 0.2780 | 7.8886 |
| 44 | 0.02092364 | 2.61579385E–6 | 7.73520369E–7 | 0.2875 | 7.8461 |
| 44 | 0.02392008 | 2.50133502E–6 | 7.45981217E–7 | 0.2898 | 7.8175 |
| 44 | 0.02787364 | 2.37319234E–6 | 7.11614838E–7 | 0.2913 | 7.8672 |
| 47 | 0.00234946 | 3.31859999E–6 | 1.50003246E–7 | 0.0452 | 7.9444 |
| 47 | 0.00413588 | 3.28548659E–6 | 2.57716081E–7 | 0.0783 | 7.9219 |
| 47 | 0.00550245 | 3.24823184E–6 | 3.29446552E–7 | 0.1011 | 7.9295 |
| 47 | 0.00725843 | 3.19003112E–6 | 4.16359779E–7 | 0.1298 | 7.8987 |
| 47 | 0.00982394 | 3.08571906E–6 | 5.16795105E–7 | 0.1659 | 7.9014 |
| 47 | 0.01285085 | 2.95589073E–6 | 5.92852385E–7 | 0.1979 | 7.861 |
| 47 | 0.01670427 | 2.78448587E–6 | 6.66123445E–7 | 0.2348 | 7.8824 |
| 47 | 0.01858195 | 2.71475844E–6 | 6.96992827E–7 | 0.2513 | 7.8424 |
| 47 | 0.02088733 | 2.62290658E–6 | 6.96299088E–7 | 0.2595 | 7.8432 |
| 47 | 0.02391521 | 2.51163131E–6 | 6.95480688E–7 | 0.2701 | 7.8506 |
| 47 | 0.02790764 | 2.37833066E–6 | 6.84283689E–7 | 0.2801 | 7.8266 |
| 50 | 0.00311013 | 3.12736044E–6 | 1.47558155E–7 | 0.0471 | 7.9217 |
| 50 | 0.00550108 | 3.08242505E–6 | 2.49586505E–7 | 0.0808 | 7.9117 |
| 50 | 0.00981794 | 2.97634722E–6 | 4.13005451E–7 | 0.1379 | 7.9114 |
| 50 | 0.01284368 | 2.88140910E–6 | 4.80570576E–7 | 0.1653 | 7.8892 |
| 50 | 0.0167038 | 2.75927652E–6 | 5.63284507E–7 | 0.2014 | 7.8926 |
| 50 | 0.01858556 | 2.69120116E–6 | 5.72365069E–7 | 0.2096 | 7.8525 |
| 50 | 0.02091244 | 2.61684329E–6 | 6.11128045E–7 | 0.2294 | 7.8391 |
| 50 | 0.02391784 | 2.51900188E–6 | 6.20952135E–7 | 0.2417 | 7.8596 |
| 50 | 0.02793494 | 2.40150991E–6 | 6.36025206E–7 | 0.2589 | 7.8562 |
| 54 | 0.00310861 | 2.90632742E–6 | 9.64719368E–8 | 0.0332 | 7.9407 |
| 54 | 0.0055003 | 2.89127522E–6 | 1.70314405E–7 | 0.0588 | 7.9167 |
| 54 | 0.00962297 | 2.82854107E–6 | 2.80125749E–7 | 0.0987 | 7.9103 |
| 54 | 0.01616173 | 2.70688162E–6 | 4.11317779E–7 | 0.1508 | 7.8855 |
| 54 | 0.01856758 | 2.64882246E–6 | 4.37476780E–7 | 0.1637 | 7.8988 |
| 54 | 0.02086828 | 2.59790693E–6 | 4.81077820E–7 | 0.1831 | 7.8764 |
| 54 | 0.02388425 | 2.53599381E–6 | 4.96393386E–7 | 0.1933 | 7.8359 |

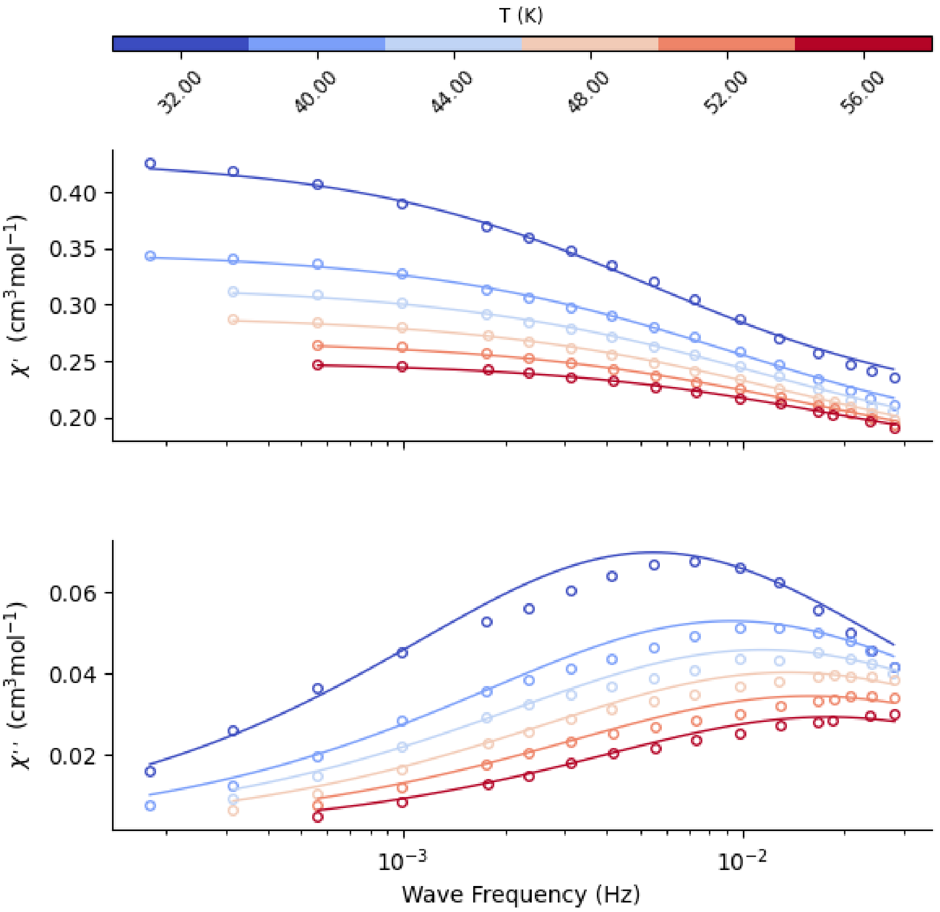


**Figure S134.** Fitting of ac susceptibilities extracted from waveform data for **3-Dy·$C_6H_6$** to generalized Debye model in CC-FIT2.[38,39]

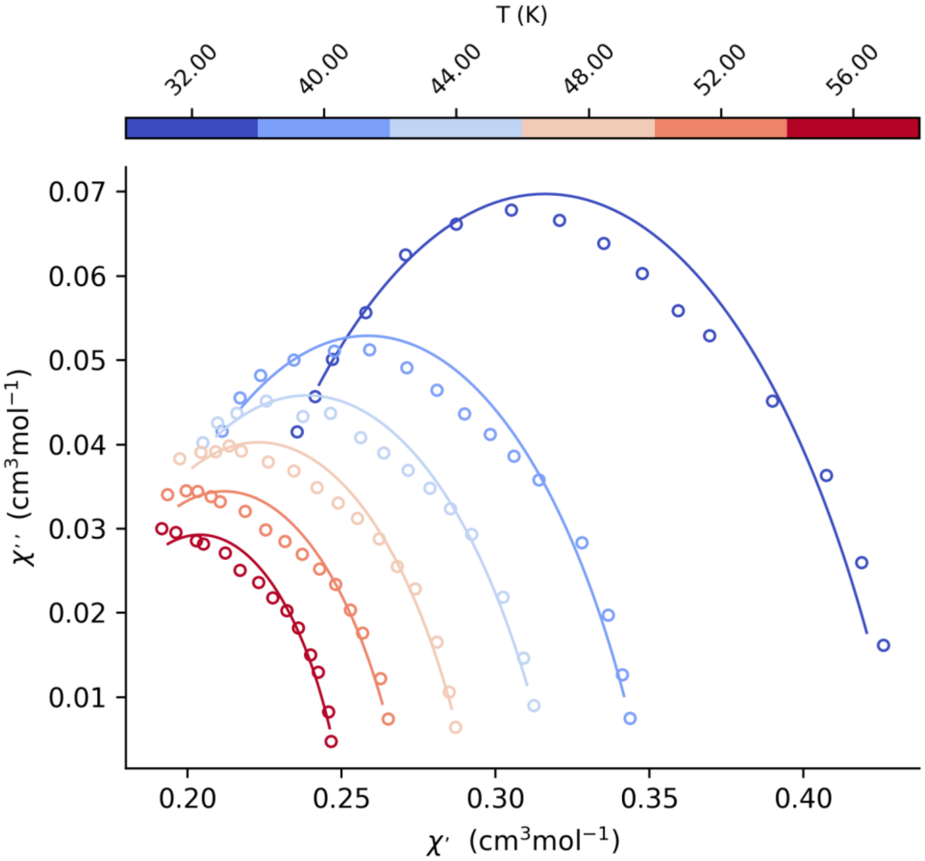


**Figure S135.** Cole-Cole plot showing fitting of ac susceptibilities extracted from waveform data for **3-Dy·$C_6H_6$** to generalized Debye model in CC-FIT2.[38,39]

**Table S24.** Best fit parameters to the generalized Debye model for **3-Dy·$C_6H_6$** waveform data in zero dc field.

| T | $\tau_{debye}$ | $\tau_{debye}^{err}$ | $\chi_S$ | $\chi_S^{err}$ | $\chi_T$ | $\chi_T^{err}$ | α | $\alpha^{err}$ |
|---|---|---|---|---|---|---|---|---|
| (K) | (s) | | (emu/mol) | | (emu/mol) | | | |
| 32 | 29.2 | 1.5 | 0.2008 | 5.3E–3 | 0.4318 | 3.5E–3 | 0.309 | 2.3E–2 |
| 40 | 17.1 | 1.1 | 0.1688 | 5.2E–3 | 0.3482 | 2.2E–3 | 0.322 | 2.2E–2 |
| 44 | 13.88 | 0.97 | 0.1596 | 5.2E–3 | 0.3181 | 2.0E–3 | 0.333 | 2.4E–2 |
| 48 | 11.64 | 0.76 | 0.1549 | 4.3E–3 | 0.2914 | 1.5E–3 | 0.322 | 2.1E–2 |
| 52 | 10.05 | 0.83 | 0.1548 | 4.7E–3 | 0.2693 | 1.6E–3 | 0.311 | 2.7E–2 |
| 56 | 8.93 | 0.68 | 0.1585 | 3.6E–3 | 0.2497 | 1.0E–3 | 0.274 | 2.5E–2 |

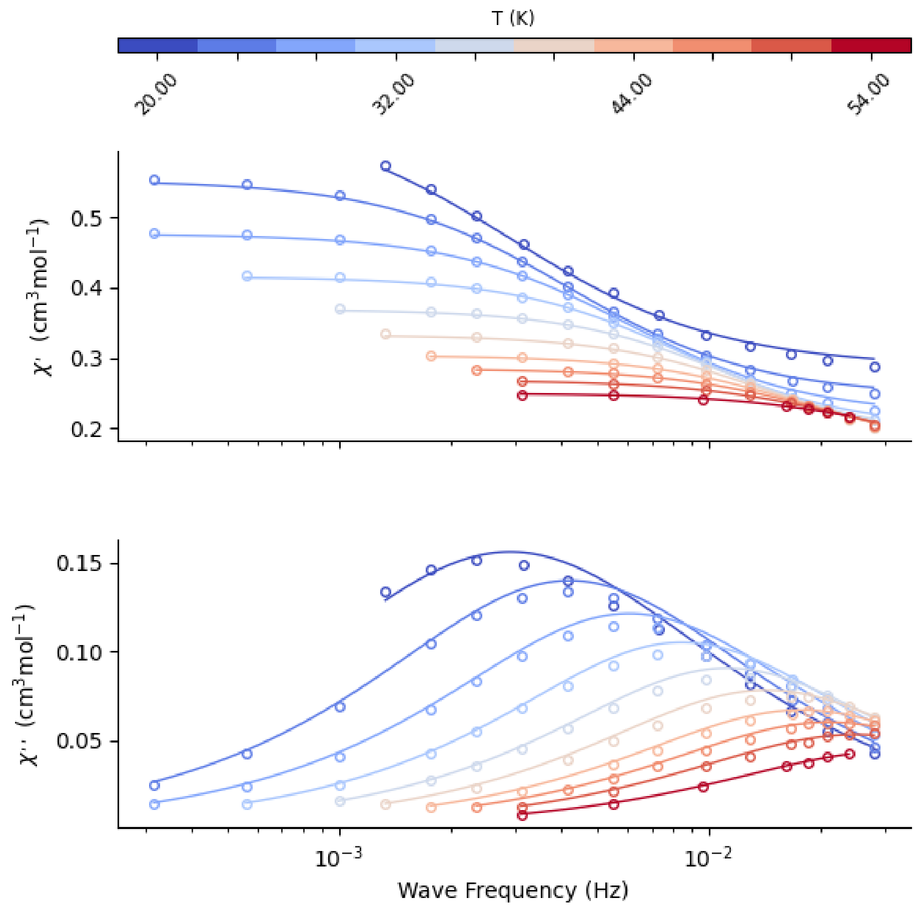


**Figure S136.** Fitting of ac susceptibilities extracted from waveform data for **4-Dy** to generalized Debye model in CC-FIT2.[38,39]

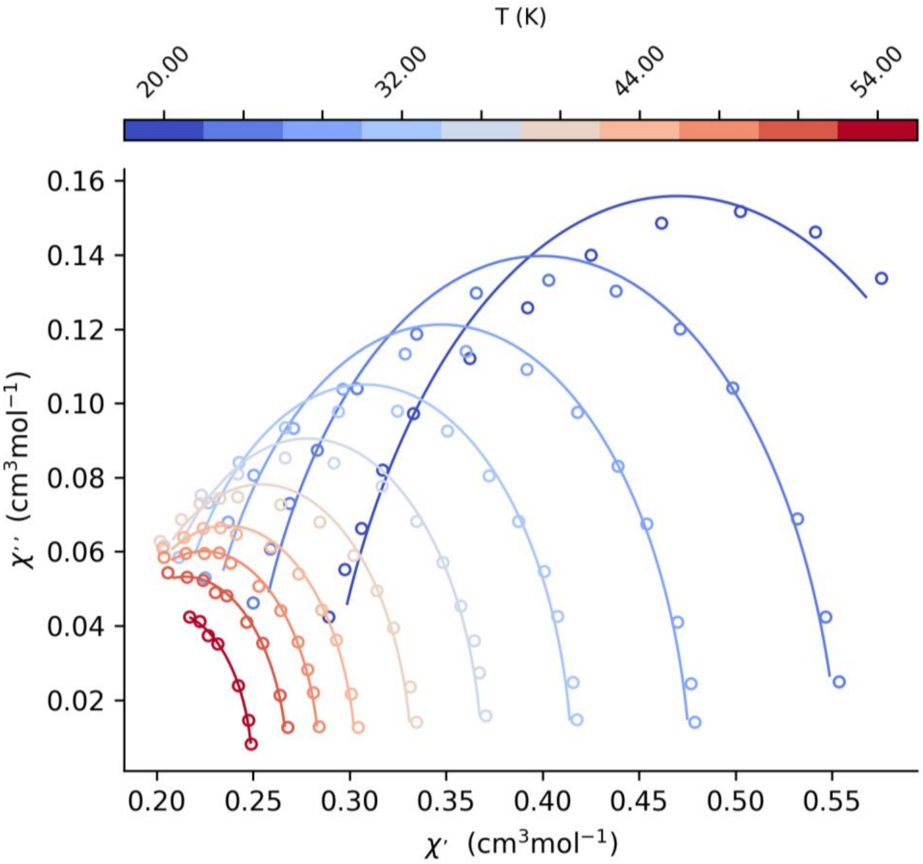


**Figure S137.** Cole-Cole plot showing fitting of ac susceptibilities extracted from waveform data for **4-Dy** to generalized Debye model in CC-FIT2.[38,39]

**Table S25.** Best fit parameters to the generalized Debye model for **4-Dy** waveform data in zero dc field.

| T | $\tau_{debye}$ | $\tau_{debye}^{err}$ | $\chi_S$ | $\chi_S^{err}$ | $\chi_T$ | $\chi_T^{err}$ | α | $\alpha^{err}$ |
|---|---|---|---|---|---|---|---|---|
| (K) | (s) | | (emu/mol) | | (emu/mol) | | | |
| 20 | 54.8 | 2.2 | 0.2844 | 4.3E–3 | 0.6558 | 9.9E–3 | 0.111 | 2.2E–2 |
| 24 | 37.85 | 0.65 | 0.2448 | 2.8E–3 | 0.5539 | 2.7E–3 | 6.4E–2 | 1.2E–2 |
| 28 | 26.15 | 0.53 | 0.2183 | 3.3E–3 | 0.4769 | 2.4E–3 | 4.1E–2 | 1.4E–2 |
| 32 | 18.86 | 0.40 | 0.2002 | 3.3E–3 | 0.4155 | 2.0E–3 | 1.6E–2 | 1.5E–2 |
| 36 | 14.21 | 0.39 | 0.1878 | 3.9E–3 | 0.3688 | 2.0E–3 | 3.23E–12 | 2.0E–2 |
| 40 | 11.12 | 0.33 | 0.1761 | 4.0E–3 | 0.3323 | 2.1E–3 | 3.76E–10 [a] | 2.3E–2 |
| 44 | 8.96 | 0.26 | 0.1694 | 3.5E–3 | 0.3035 | 1.5E–3 | 2.59E–12 [a] | 2.0E–2 |
| 47 | 7.54 | 0.23 | 0.1643 | 3.3E–3 | 0.2846 | 1.1E–3 | 2.20E–10 [a] | 1.9E–2 |
| 50 | 6.35 | 0.29 | 0.1615 | 4.3E–3 | 0.2680 | 1.3E–3 | 7.06E–15 [a] | 2.6E–2 |
| 54 | 5.06 | 0.21 | 0.1620 | 3.0E–3 | 0.24960 | 4.8E–4 | 2.19E–9 [a] | 1.6E–2 |

*a* Value of α fixed to 0.05 so as to not over-bias points when fitting relaxation profile

*9.4 Magnetization Decays*

Dc magnetisation decay measurements on **3-Dy·$C_6H_6$**, **4-Dy** and "[{Dy($Cp^{ttt}$)(Cp*)}{Al[OC($CF_3$)$_3$]$_4$}]" were performed at constant temperature by applying a 30 kOe magnetic field for a minimum of 5 minutes to saturate the sample ($M_{sat}$) and then rapidly removing the field (700 Oe $s^{-1}$) and measuring the magnetic moment as a function of time as soon as the field stabilized at zero. Measurements were performed with dc scan mode with 40 mm scan length and 6 s scan time, this corresponded to points measured every ~14.7 s. Dc magnetisation decay measurements on **5%Dy@4-Y** and **4-Dy** frozen solution were performed at constant temperature with an updated measurement protocol[38,39] using VSM mode with 3 mm vibration amplitude and 0.5 s averaging time, measuring continuously through the field change (700 Oe $s^{-1}$): 30 kOe to calibrated zero field (+24.2 Oe for **5%Dy@4-Y**, +26.4 Oe for **4-Y** in benzene) at 700 Oe $s^{-1}$ for 2–24 K and 70 kOe to calibrated zero field (+24.2 Oe for **5%Dy@4-Y**, +25.9 Oe for **4-Y** in benzene) at 700 Oe $s^{-1}$ for $T \geq 28$ K.

The first point measured in zero field (within 0.5 Oe of target) was used to define $t = 0$, and the corresponding magnetic moment, $M_0$. Decay curves were measured until the magnetisation had decayed to 1% of $M_0$ unless specified otherwise or as indicated in Table S26.[39] At the higher temperature limit of magnetization decays, significant relaxation of the sample occurred as the field was swept to zero. Datasets with $M_0/M_{sat} < 0.07$ were discarded, this value was chosen based on **4-Dy**, where relaxation rates from magnetization decays begin to diverge from waveform-based rates at 40 K, where $M_0/M_{sat} = 0.062$. An artifact the dc auto-ranging function on the instrument is to misreport the timestamp of the datapoint immediately after adjusting the range, these points were removed. The curated magnetization decay datasets covered 2–56 K for **3-Dy·$C_6H_6$**, 2–38 K for **4-Dy**, 2–52 K for "[{Dy($Cp^{ttt}$)(Cp*)}{Al[OC($CF_3$)$_3$]$_4$}]", 2–48 K for **5%Dy@4-Y** and 2–48 K for **4-Dy** in benzene.

**Table S26**. End point for decays not measured to 1% $M_0$ (less than 99% of decay)

| $T$ (K) | **3-Dy·$C_6H_6$** | | "[{Dy(Cp$^{ttt}$)(Cp*)}{Al[OC($CF_3$)$_3$]$_4$}]" | | **4-Dy** in benzene | |
|---|---|---|---|---|---|---|
| | % of $M_0$ | $t$ (h) | % of $M_0$ | $t$ (h) | % of $M_0$ | $t$ (h) |
| 2 | 8.6 | 10 | 2.0 | 3.3 | 5.3 | 10 |
| 3 | 9.4 | 7.5 | 1.8 | 3.0 | 6.3 | 6 |
| 4 | 8.5 | 7.3 | 1.6 | 3.0 | 6.4 | 6 |
| 6 | 5.1 | 7 | 1.9 | 2.5 | 4.8 | 6 |
| 8 | 2.5 | 6 | 1.4 | 2.5 | 2.5 | 6 |
| 10 | 1.1 | 5 | n/a | n/a | 1.3 | 5 |

Dc decay data were fit to a stretched exponential curve (Equation S3) in OriginPro[42]

$$M(t) = M_{\mathrm{eq}} + \left(M_0 - M_{\mathrm{eq}}\right)e^{-\left(\frac{t}{\tau*}\right)^{\beta}}$$

Equation S3

where $\tau^*$ is the characteristic relaxation time, $\beta$ is the stretching factor and $M_{\mathrm{eq}}$ is equilibrium magnetisation at infinite time. $M_0$ was fixed to the value at $t = 0$. Measurements on a Pd standard on the MPMS3 instrument indicated a small negative residual field after sweeping from 30 kOe to a target of 0 Oe (*ca.* –22 Oe) and so only negative values of $M_{\mathrm{eq}}$ are physical for **3-Dy·$C_6H_6$**, **4-Dy** and "[{Dy(Cp$^{ttt}$)(Cp*)}{Al[OC($CF_3$)$_3$]$_4$}]". For these datasets $M_{\mathrm{eq}}$ was initially allowed to refine freely, and datasets with positive $M_{\mathrm{eq}}$ values were re-fit with $M_{\mathrm{eq}}$ fixed to zero. For **5%Dy@4-Y** and **4-Dy** in benzene, the target field was calibrated to very close to zero field, so $M_{\mathrm{eq}}$ was fixed to zero. Fits are shown in Figures S138 and S139 for **3-Dy·$C_6H_6$** and Figure S140 and S141 for **4-Dy**, Figures S142–S144 for "[{Dy(Cp$^{ttt}$)(Cp*)}{Al[OC($CF_3$)$_3$]$_4$}]", Figure S145 for **5%Dy@4-Y** (2–18 K) and Figure S146 for **4-Dy** in benzene (28–52 K) with parameters in Table S27 (**3-Dy·$C_6H_6$**), Table S28 (**4-Dy**), Table S29 ("[{Dy(Cp$^{ttt}$)(Cp*)}{Al[OC($CF_3$)$_3$]$_4$}]"), Table S30 (**5%Dy@4-Y**, 2–18 K) and Table S31 (**4-Dy** in benzene, 28–52 K). Fits to the stretched exponential model are generally good, except for "[{Dy(Cp$^{ttt}$)(Cp*)}{Al[OC($CF_3$)$_3$]$_4$}]" and low temperature data ($T \leq 8$ K) for **3-Dy·$C_6H_6$**. The lower temperature (2–8 K) datasets for **3-Dy·$C_6H_6$** are well reproduced at longer

timescales but the fitted curve decays too rapidly initially (Figure S138b). Therefore **3-Dy·$C_6H_6$** has a distribution of relaxation rates that deviates from the assumed distribution in the stretched exponential function model, and the model includes a too large proportion of fast rates at low temperatures. The slight decrease in $e^{\langle \ln \tau \rangle}$ at low temperatures (Table S35) is likely an artifact from this poor fit.

Fits to the single stretched exponential function are poor at low and intermediate temperatures for "[{Dy($Cp^{ttt}$)(Cp*)}{Al[OC($CF_3$)$_3$]$_4$}]" (Figures S142 and S143). The calculated rates (§9.5, Figure S164b) behave unusually; the rates below 28 K are as expected, but then they plateau for 28–36 K, followed by a decrease in both rates and distributions from 36–48 K. Furthermore, the rates for "[{Dy($Cp^{ttt}$)(Cp*)}{Al[OC($CF_3$)$_3$]$_4$}]" extracted from a single stretched exponential function are similar to **4-Dy** at low temperatures but trend towards the rates for **3-Dy·$C_6H_6$** at higher temperatures (comparison made to rates extracted from magnetization decays with a single stretched exponential function). The "[{Dy($Cp^{ttt}$)(Cp*)}{Al[OC($CF_3$)$_3$]$_4$}]" sample is amorphous and potentially inhomogeneous, so we investigated the possibility of "[{Dy($Cp^{ttt}$)(Cp*)}{Al[OC($CF_3$)$_3$]$_4$}]" being composed of two components with different relaxation times by fitting the magnetization decay data to a sum of two stretched exponential functions:

$$M(t) = M_{\mathrm{eq}} + \left(M_0 - M_{\mathrm{eq}}\right)\eta_1 e^{-\left(\frac{t}{\tau*_1}\right)^{\beta_1}} + \left(M_0 - M_{\mathrm{eq}}\right)(1-\eta_1)e^{-\left(\frac{t}{\tau*_2}\right)^{\beta_2}}$$

Equation S4

where $\tau *_1$ and $\beta_1$ characterize the faster relaxing component, $\tau *_2$ and $\beta_2$ characterize the slower relaxing component and $\eta_1$ is the fraction of the faster relaxing component. Over parametrization can be a problem when fitting so many parameters, so we have fixed parameters wherever reasonable guesses are available, as detailed below. The value of $M_0$ was

fixed to the first point measured in zero field ($t$ = 0) and $M_{eq}$ was fixed to zero. Fits are shown in Figures S147–S149 with parameters in Table S32.

The magnetization decay data for "[{Dy(Cp$^{ttt}$)(Cp*)}{Al[OC($CF_3$)$_3$]$_4$}]" at low and intermediate temperatures are significantly better modelled by Equation S4 than Equation S3. From 2–16 K we resolve two relaxation processes with $\tau^*{}_2/\tau^*{}_1$ = 7.3–8.6 and $\eta_1$ = 0.66–0.74. The consistent value of $\eta_1$, and smooth trend in $\tau^*{}_1$ and $\tau^*{}_2$ values suggest we are resolving relaxation processes originating from two distinct Dy environments in the amorphous sample. Errors in $\eta_1$ (and other parameters) increase at lower temperature: at 16 K, $\eta_1$= 0.696 ± 0.007, while at 3 K, $\eta_1$ = 0.67 ± 0.14. This suggests slight over-parameterization as the distribution of both components become broader, overlap more, and are less easily distinguished. As the free values of $\eta_1$ are approximately constant, we refit the data (2–16 K) with $\eta_1$ fixed to 0.7 to have more confidence in the τ* and β parameters extracted for each component. At 18 K and above for "[{Dy(Cp$^{ttt}$)(Cp*)}{Al[OC($CF_3$)$_3$]$_4$}]", the freely fit value of $\eta_1$ steadily reduces, reaching 0.26 at 44 K. As the ratio of Dy environments in the sample are unchanged with temperature, this can only be explained by fast-relaxing components relaxing preferentially while sweeping the field to zero and stabilising the field (over ~62 s) in the process of setting up the magnetization decay experiment. For 46–52 K with "[{Dy(Cp$^{ttt}$)(Cp*)}{Al[OC($CF_3$)$_3$]$_4$}]", the fit is easily overparameterized as $\eta_1$ < 0.24 and $\tau^*{}_1$ < 8 s, so the fast-relaxing component only contributes significantly to the first two datapoints. To avoid overparameterization we fixed $\beta_1$ to 0.94 for 46–52 K; $\beta_1$ is consistently ~0.94 for 30–44 K. The value of $\eta_1$ continues to reduce with increasing temperature, reaching 0.16 at 52 K.

On increasing temperature for **5%Dy@4-Y**, the stretched exponential function becomes inadequate to model the experimental data, and deviates from experiment at long timescales. For 20–38 K, a sum of two exponential functions (Equation S4) with $M_{eq}$ fixed to zero can account for the decay at all timescales. These fits (Figure S150, Table S33) have a

major component ($\eta_1$ = 0.90–0.96) and a minor component (0.04–0.10 of observed decay). To better fit the minor component, we extended the dataset to include decay until the moment reached 0.1% of $M_0$.

For **4-Dy** in benzene, at lower temperatures the fit to a single stretched exponential function became increasingly inadequate. Decays from 2–24 K were instead fit to a sum of two stretched exponential functions (Figure S151, Table S34). A second fast component is observed that grows in intensity on lowering temperature, from $\eta_1$ = 0.12 at 24 K to 0.80 at 2 K. This behaviour is similar to the "[{Dy(Cp$^{ttt}$)(Cp*)}{Al[OC($CF_3$)$_3$]$_4$}]" and suggests **4-Dy** in benzene is also inhomogeneous, consistent with the appearance of the sample.

The raw magnetization decay curves can be compared to make some broad comparisons about relaxation rates, without relying on assumptions of the fitting model. We select 28 K (Figure S152) as an example intermediate (Raman regime) temperature and 2 K (Figure S153) as an example low temperature (QTM regime). At 28 K the rates of decay follow: **4-Dy** ≈ **5%Dy@4-Y** > "[{Dy(Cp$^{ttt}$)(Cp*)}{Al[OC($CF_3$)$_3$]$_4$}]" ≈ **4-Dy** in benzene > **3-Dy·$C_6H_6$**. This is consistent with similar and fast Raman rates for **4-Dy** and **5%Dy@4-Y**. The SIP has the slowest Raman rates. The inhomogeneous samples have intermediate rates, with the largest distribution of rates for **4-Dy** in benzene, as it has initial decay similar to "[{Dy(Cp$^{ttt}$)(Cp*)}{Al[OC($CF_3$)$_3$]$_4$}]" but at long timescales is even slower than **3-Dy·$C_6H_6$** (Figure S152c). At 2 K **3-Dy·$C_6H_6$** has the slowest decay, **4-Dy** is much faster and both **5%Dy@4-Y** and "[{Dy(Cp$^{ttt}$)(Cp*)}{Al[OC($CF_3$)$_3$]$_4$}]" are intermediate to these. **4-Dy** in benzene at 2 K has a very large distribution of rates, the decay is faster than **4-Dy** at low temperatures and slower than **3-Dy·$C_6H_6$** at long timescales (Figure S153c).

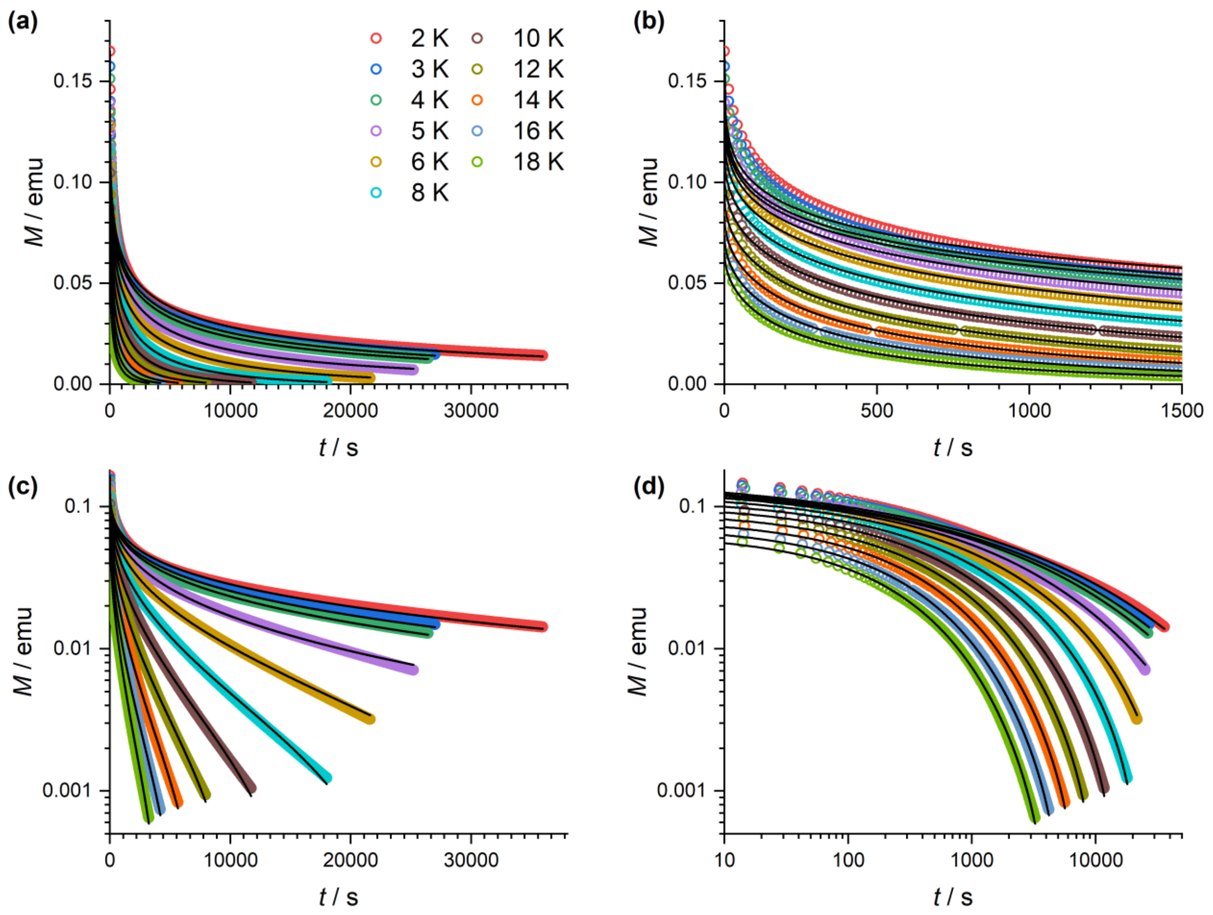


**Figure S138.** Fit of dc magnetisation decay data (2–20 K) for **3-Dy·$C_6H_6$** to a stretched exponential model, with parameters given in Table S27. Represented as (a) linear-linear plot, (b) linear-linear plot at low times, (c) log-linear plot and (d) log-log plot.

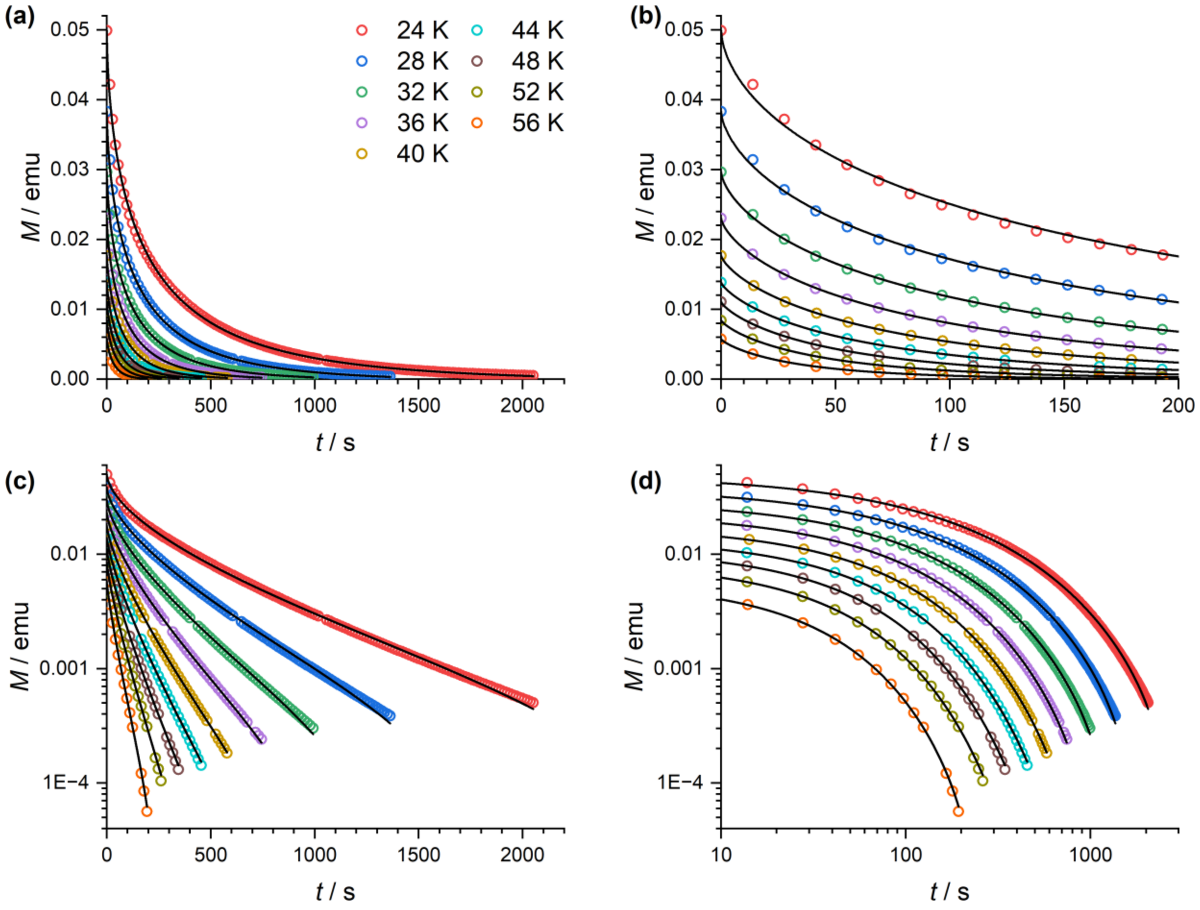


**Figure S139.** Fit of dc magnetisation decay data (24–56 K) for **3-Dy·$C_6H_6$** to a stretched exponential model, with parameters given in Table S27. Represented as (a) linear-linear plot, (b) linear-linear plot at low times, (c) log-linear plot and (d) log-log plot.

**Table S27.** Parameters from fitting magnetization decays of **3-Dy·$C_6H_6$** to stretched exponential function

| $T$ | $M_{sat}$ | $M_0$ | $M_0/M_{sat}$ | $M_{eq}$ | $M_{eq}^{err}$ | $\tau^*$ | $\tau^{*err}$ | $\beta$ | $\beta^{err}$ | $R^2$ |
|---|---|---|---|---|---|---|---|---|---|---|
| (K) | (emu) | (emu) | | (emu) | (emu) | (s) | (s) | | | |
| 2 | 0.32867 | 0.16502 | 0.50208 | 0 | 0 | 1237.4 | 6.0 | 0.26957 | 5.6E–4 | 0.99019 |
| 3 | 0.32570 | 0.15740 | 0.48326 | 0 | 0 | 1204.6 | 6.5 | 0.28231 | 7.2E–4 | 0.98920 |
| 4 | 0.32112 | 0.15130 | 0.47118 | 0 | 0 | 1211.7 | 5.6 | 0.29570 | 6.6E–4 | 0.99203 |
| 6 | 0.30775 | 0.13942 | 0.45304 | 0 | 0 | 1159.5 | 3.5 | 0.34500 | 5.4E–4 | 0.99651 |
| 8 | 0.29025 | 0.12800 | 0.44099 | –2.269E–3 | 9.6E–5 | 1094.9 | 4.1 | 0.38281 | 9.8E–4 | 0.99829 |
| 10 | 0.27106 | 0.11603 | 0.42804 | –1.776E–3 | 4.8E–5 | 870.3 | 1.9 | 0.43239 | 7.8E–4 | 0.99915 |
| 12 | 0.25147 | 0.10463 | 0.41608 | –1.280E–3 | 4.5E–5 | 676.0 | 1.5 | 0.47457 | 9.1E–4 | 0.99937 |
| 14 | 0.23318 | 0.09351 | 0.40101 | –9.38E–4 | 5.0E–5 | 521.6 | 1.4 | 0.5064 | 1.2E–3 | 0.99933 |
| 16 | 0.21584 | 0.08288 | 0.38401 | –7.11E–4 | 5.7E–5 | 407.2 | 1.3 | 0.5314 | 1.6E–3 | 0.99924 |
| 18 | 0.20024 | 0.07331 | 0.36608 | –5.60E–4 | 6.2E–5 | 325.1 | 1.3 | 0.5511 | 2.1E–3 | 0.99918 |
| 20 | 0.18591 | 0.06483 | 0.34869 | –4.69E–4 | 6.4E–5 | 266.9 | 1.2 | 0.5677 | 2.5E–3 | 0.99917 |
| 24 | 0.16229 | 0.04990 | 0.30746 | –3.47E–4 | 5.6E–5 | 190.06 | 0.93 | 0.5983 | 2.9E–3 | 0.99939 |
| 28 | 0.14310 | 0.03833 | 0.26787 | –2.72E–4 | 4.1E–5 | 144.04 | 0.63 | 0.6341 | 2.8E–3 | 0.99968 |
| 32 | 0.12806 | 0.02963 | 0.23135 | –1.65E–4 | 2.5E–5 | 114.33 | 0.38 | 0.6677 | 2.3E–3 | 0.99987 |
| 36 | 0.11564 | 0.02303 | 0.19915 | –9.4E–5 | 1.5E–5 | 93.54 | 0.22 | 0.7018 | 1.8E–3 | 0.99995 |
| 40 | 0.10534 | 0.01770 | 0.16799 | –3.74E–5 | 5.5E–6 | 78.96 | 0.09 | 0.74156 | 9.1E–4 | 0.99999 |
| 44 | 0.09665 | 0.01388 | 0.14360 | –1.41E–5 | 4.0E–6 | 66.10 | 0.07 | 0.77108 | 9.3E–4 | 0.99999 |
| 48 | 0.08925 | 0.01108 | 0.12410 | –1.09E–5 | 6.7E–6 | 54.51 | 0.11 | 0.7890 | 1.7E–3 | 0.99999 |
| 52 | 0.08289 | 0.00843 | 0.10167 | –1.32E–5 | 8.8E–6 | 44.73 | 0.14 | 0.8094 | 2.9E–3 | 0.99998 |
| 56 | 0.07736 | 0.00573 | 0.07408 | –3.54E–5 | 7.4E–6 | 35.56 | 0.13 | 0.8309 | 3.7E–3 | 0.99998 |

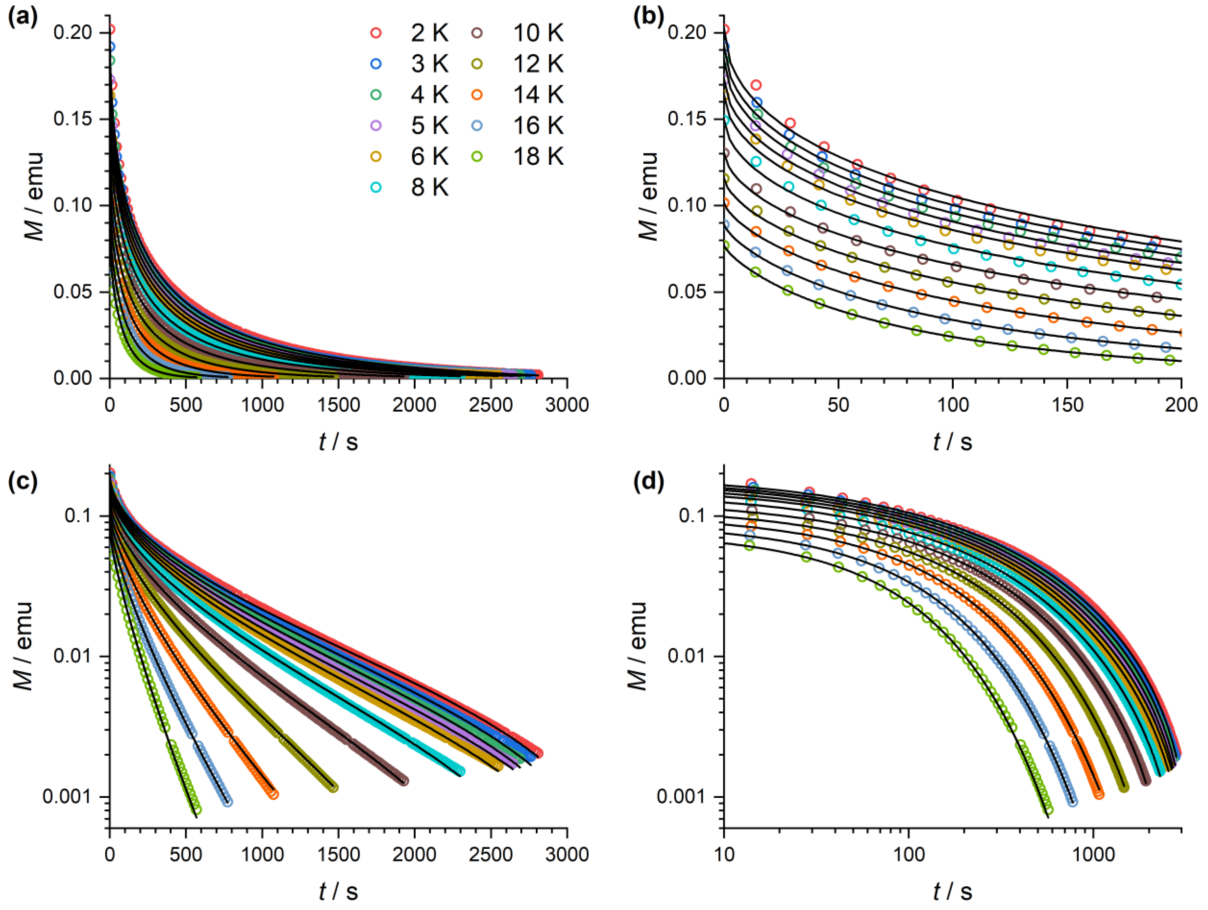


**Figure S140.** Fit of dc magnetisation decay data (2–18 K) for **4-Dy** to stretched exponential model, with parameters given in Table S28. Represented as (a) linear-linear plot, (b) linear-linear plot at low times, (c) log-linear plot and (d) log-log plot.

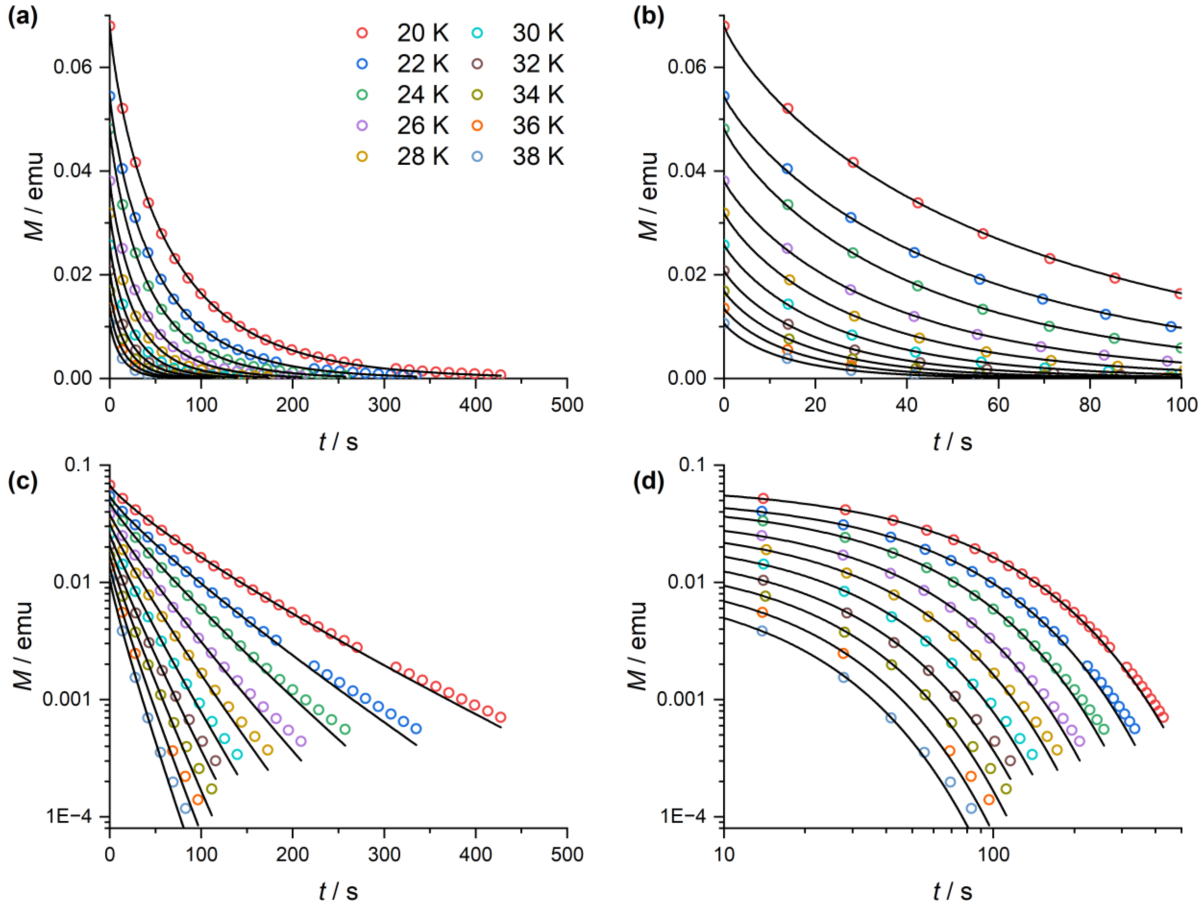


**Figure S141.** Fit of dc magnetisation decay data (20–38 K) for **4-Dy** to stretched exponential model, with parameters given in Table S28. Represented as (a) linear-linear plot, (b) linear-linear plot at low times, (c) log-linear plot and (d) log-log plot.

**Table S28.** Parameters from fitting magnetization decays of **4-Dy** to stretched exponential function

| $T$ | $M_{sat}$ | $M_0$ | $M_0/M_{sat}$ | $M_{eq}$ | $M_{eq}^{err}$ | $\tau^*$ | $\tau^{*err}$ | $\beta$ | $\beta^{err}$ | $R^2$ |
|---|---|---|---|---|---|---|---|---|---|---|
| (K) | (emu) | (emu) | | (emu) | (emu) | (s) | (s) | | | |
| 2 | 0.39632 | 0.20206 | 0.50983 | –3.87E–3 | 3.1E–4 | 241.5 | 1.6 | 0.5187 | 2.8E–3 | 0.99909 |
| 3 | 0.39420 | 0.19190 | 0.48681 | –3.18E–3 | 2.2E–4 | 236.5 | 1.2 | 0.5313 | 2.3E–3 | 0.99941 |
| 4 | 0.38785 | 0.18396 | 0.47430 | –2.55E–3 | 1.8E–4 | 228.1 | 1.0 | 0.5408 | 2.1E–3 | 0.99949 |
| 5 | 0.38111 | 0.17295 | 0.45381 | –1.87E–3 | 1.8E–4 | 226.6 | 1.0 | 0.5566 | 2.3E–3 | 0.99941 |
| 6 | 0.37248 | 0.16362 | 0.43927 | –1.51E–3 | 1.6E–4 | 222.24 | 0.94 | 0.5678 | 2.3E–3 | 0.99948 |
| 8 | 0.35142 | 0.14929 | 0.42481 | –1.21E–3 | 1.5E–4 | 205.41 | 0.89 | 0.5795 | 2.4E–3 | 0.99951 |
| 10 | 0.33001 | 0.13033 | 0.39494 | –7.4E–4 | 1.1E–4 | 188.63 | 0.68 | 0.6161 | 2.2E–3 | 0.99970 |
| 12 | 0.30757 | 0.11571 | 0.37619 | –3.65E–4 | 9.1E–5 | 161.80 | 0.52 | 0.6629 | 2.2E–3 | 0.99980 |
| 14 | 0.28550 | 0.10157 | 0.35575 | –1.5E–5 | 7.6E–5 | 133.90 | 0.39 | 0.7208 | 2.3E–3 | 0.99988 |
| 16 | 0.26519 | 0.08898 | 0.33554 | 0 | 0 | 104.89 | 0.20 | 0.7616 | 1.7E–3 | 0.99991 |
| 18 | 0.24668 | 0.07698 | 0.31205 | 0 | 0 | 83.24 | 0.20 | 0.8032 | 2.4E–3 | 0.99989 |
| 20 | 0.22945 | 0.06799 | 0.29634 | 0 | 0 | 65.65 | 0.18 | 0.8328 | 3.0E–3 | 0.99990 |
| 22 | 0.21492 | 0.05447 | 0.25346 | 0 | 0 | 53.61 | 0.22 | 0.8657 | 4.8E–3 | 0.99983 |
| 24 | 0.20090 | 0.04817 | 0.23977 | 0 | 0 | 43.13 | 0.19 | 0.8754 | 5.2E–3 | 0.99985 |
| 26 | 0.18934 | 0.03806 | 0.20104 | 0 | 0 | 35.70 | 0.20 | 0.8914 | 6.9E–3 | 0.99981 |
| 28 | 0.17792 | 0.03189 | 0.17924 | 0 | 0 | 29.50 | 0.17 | 0.8925 | 7.4E–3 | 0.99986 |
| 30 | 0.16853 | 0.02577 | 0.15290 | 0 | 0 | 24.91 | 0.18 | 0.9008 | 9.4E–3 | 0.99983 |
| 32 | 0.15938 | 0.02080 | 0.13052 | 0 | 0 | 20.97 | 0.16 | 0.893 | 1.0E–2 | 0.99987 |
| 34 | 0.15164 | 0.01689 | 0.11137 | 0 | 0 | 18.17 | 0.17 | 0.898 | 1.2E–2 | 0.99985 |
| 36 | 0.14426 | 0.01356 | 0.09399 | 0 | 0 | 15.62 | 0.19 | 0.892 | 2.1E–2 | 0.99986 |
| 38 | 0.13752 | 0.01066 | 0.07752 | 0 | 0 | 13.53 | 0.17 | 0.888 | 1.6E–2 | 0.99987 |

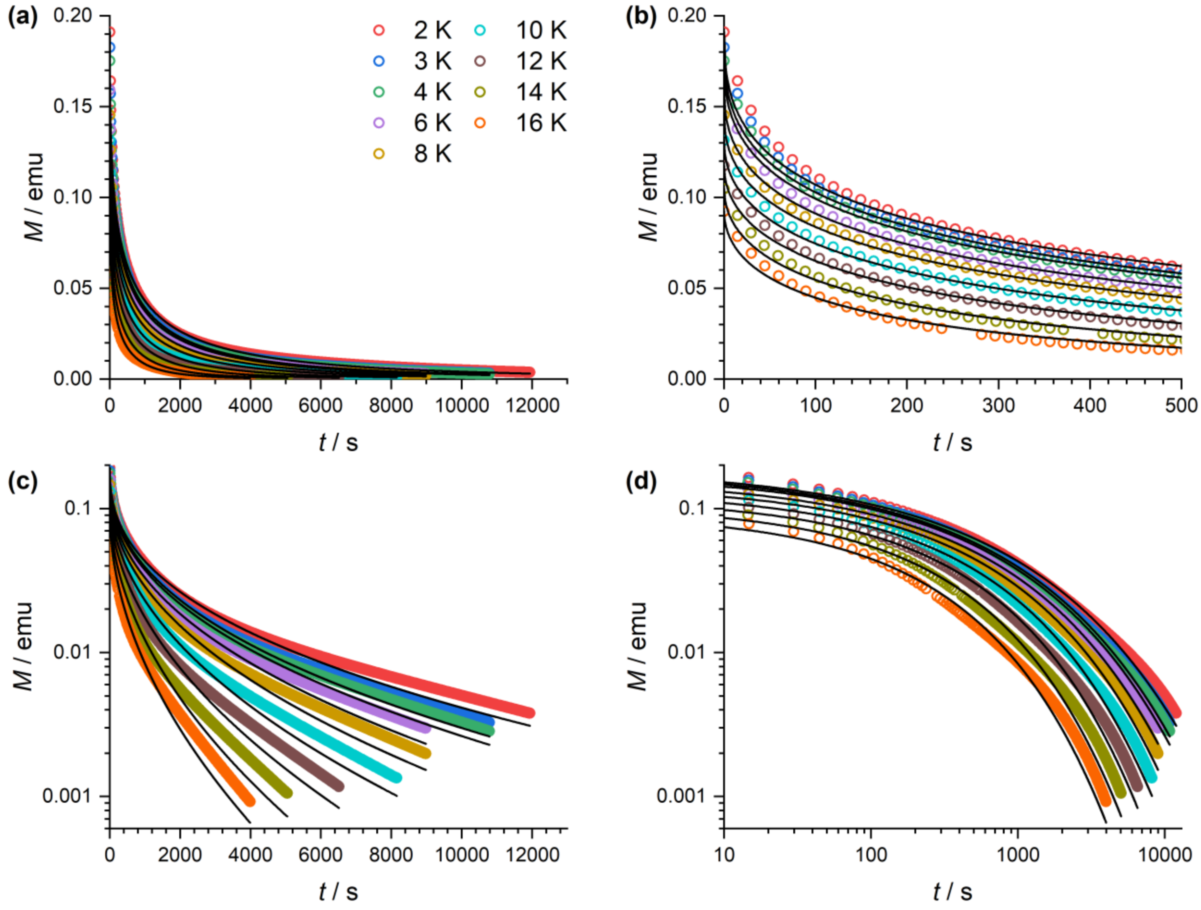


**Figure S142.** Fit of dc magnetisation decay data (2–16 K) for "[{Dy(Cp$^{ttt}$)(Cp*)}{Al[OC($CF_3$)$_3$]$_4$}]" to a single stretched exponential model, with parameters given in Table S29. Represented as (a) linear-linear plot, (b) linear-linear plot at low times, (c) log-linear plot and (d) log-log plot.

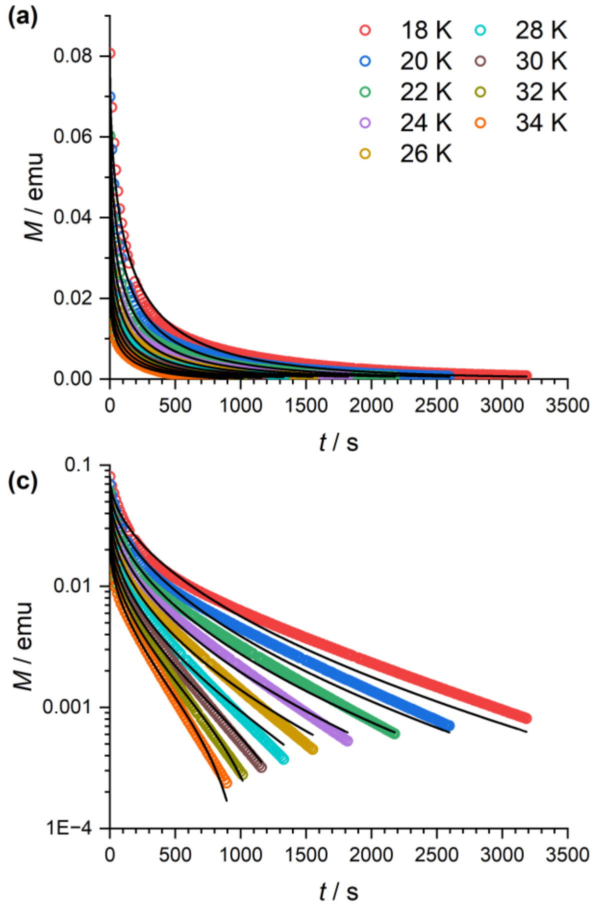


**Figure S143.** Fit of dc magnetisation decay data (18–34 K) for "[{Dy($Cp^{ttt}$)(Cp*)}{Al[OC($CF_3$)$_3$]$_4$}]" to a single stretched exponential model, with parameters given in Table S29. Represented as (a) linear-linear plot, (b) linear-linear plot at low times, (c) log-linear plot and (d) log-log plot.

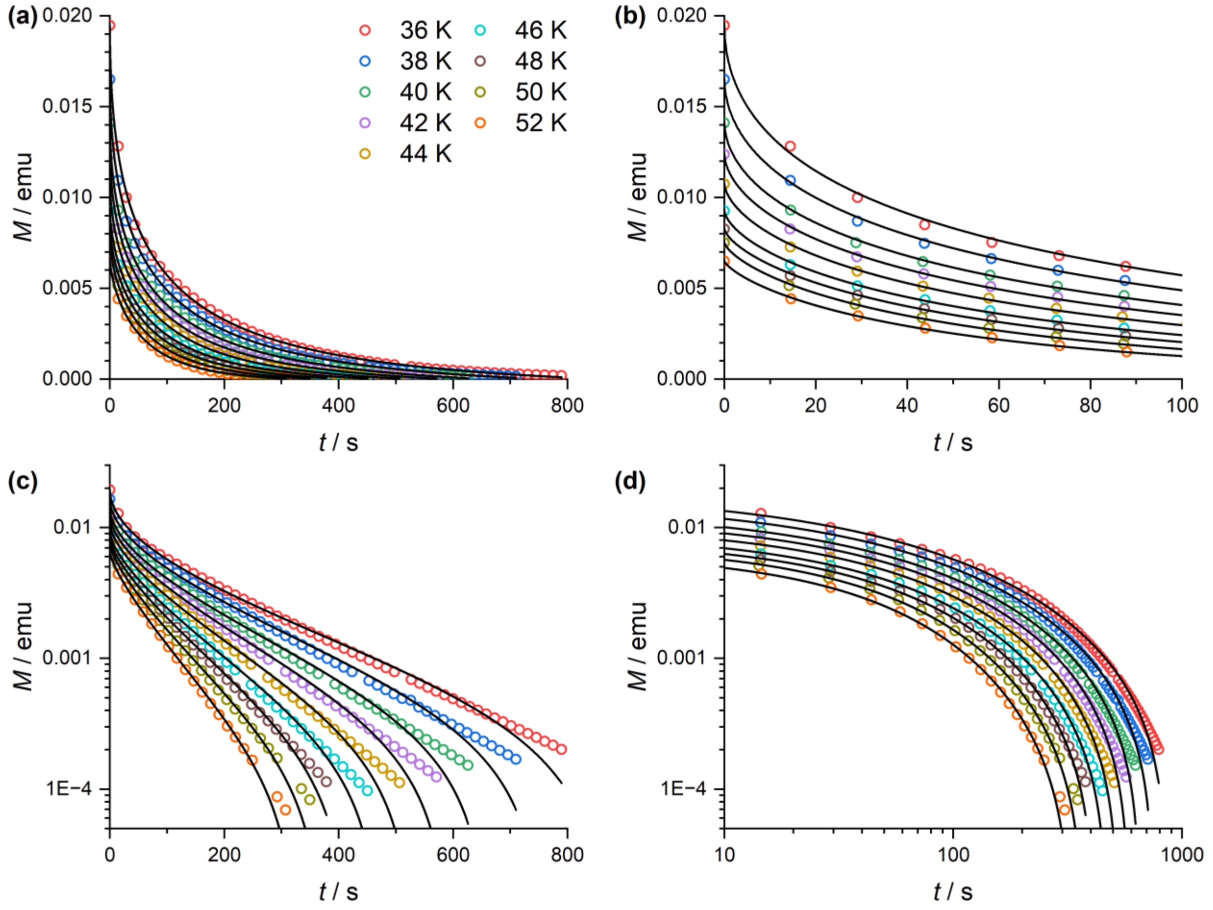


**Figure S144.** Fit of dc magnetisation decay data (36–52 K) for "[{Dy(Cp$^{ttt}$)(Cp*)}{Al[OC($CF_3$)$_3$]$_4$}]" to a single stretched exponential model, with parameters given in Table S29. Represented as (a) linear-linear plot, (b) linear-linear plot at low times, (c) log-linear plot and (d) log-log plot.

**Table S29.** Parameters from fitting magnetization decays of "[{Dy(Cp$^{ttt}$)(Cp*)}{Al[OC($CF_3$)$_3$]$_4$}]" to a single stretched exponential function

| $T$ | $M_{sat}$ | $M_0$ | $M_0/M_{sat}$ | $M_{eq}$ | $M_{eq}^{err}$ | $\tau^*$ | $\tau^{*err}$ | $\beta$ | $\beta^{err}$ | $R^2$ |
|---|---|---|---|---|---|---|---|---|---|---|
| (K) | (emu) | (emu) | | (emu) | (emu) | (s) | (s) | | | |
| 2 | 0.36617 | 0.19114 | 0.52200 | 0 | 0 | 378.0 | 2.1 | 0.4100 | 1.2E–3 | 0.99560 |
| 3 | 0.36269 | 0.18264 | 0.50358 | 0 | 0 | 366.1 | 1.9 | 0.4273 | 1.2E–3 | 0.99617 |
| 4 | 0.35722 | 0.17532 | 0.49078 | 0 | 0 | 367.6 | 1.8 | 0.4343 | 1.2E–3 | 0.99648 |
| 6 | 0.34228 | 0.15932 | 0.46546 | 0 | 0 | 365.3 | 1.8 | 0.4501 | 1.3E–3 | 0.99667 |
| 8 | 0.32302 | 0.14543 | 0.45024 | 0 | 0 | 355.7 | 1.7 | 0.4694 | 1.4E–3 | 0.99685 |
| 10 | 0.30191 | 0.13146 | 0.43542 | 0 | 0 | 320.4 | 1.6 | 0.4888 | 1.6E–3 | 0.99689 |
| 12 | 0.28033 | 0.11744 | 0.41895 | 0 | 0 | 281.8 | 1.8 | 0.5090 | 2.2E–3 | 0.99583 |
| 14 | 0.25894 | 0.10477 | 0.40461 | 0 | 0 | 229.0 | 2.0 | 0.5185 | 3.1E–3 | 0.99414 |
| 16 | 0.24047 | 0.09246 | 0.38449 | 0 | 0 | 186.0 | 2.1 | 0.5214 | 4.1E–3 | 0.99220 |
| 18 | 0.22315 | 0.08071 | 0.36168 | 0 | 0 | 152.1 | 2.1 | 0.5193 | 5.0E–3 | 0.99075 |
| 20 | 0.20768 | 0.06994 | 0.33675 | 0 | 0 | 126.8 | 2.1 | 0.5143 | 5.8E–3 | 0.99024 |
| 22 | 0.19376 | 0.06027 | 0.31108 | 0 | 0 | 108.1 | 1.8 | 0.5063 | 5.8E–3 | 0.99131 |
| 24 | 0.18133 | 0.05156 | 0.28434 | 0 | 0 | 95.1 | 1.6 | 0.5037 | 5.9E–3 | 0.99267 |
| 26 | 0.17020 | 0.04390 | 0.25793 | 0 | 0 | 85.6 | 1.4 | 0.5038 | 5.9E–3 | 0.99395 |
| 28 | 0.16031 | 0.03692 | 0.23032 | –1.2E–4 | 1.4E–4 | 78.5 | 1.5 | 0.4992 | 9.3E–3 | 0.99544 |
| 30 | 0.15137 | 0.03138 | 0.20730 | –3.4E–4 | 1.3E–4 | 76.7 | 1.5 | 0.4940 | 8.7E–3 | 0.99671 |
| 32 | 0.14308 | 0.02667 | 0.18644 | –5.0E–4 | 1.1E–4 | 76.4 | 1.4 | 0.4947 | 7.8E–3 | 0.99791 |
| 34 | 0.13591 | 0.02277 | 0.16756 | –5.95E–4 | 8.9E–5 | 75.6 | 1.2 | 0.4981 | 6.8E–3 | 0.99872 |
| 36 | 0.12931 | 0.01947 | 0.15060 | –6.37E–4 | 7.4E–5 | 75.7 | 1.1 | 0.5083 | 6.1E–3 | 0.99917 |
| 38 | 0.12324 | 0.01650 | 0.13392 | –5.96E–4 | 6.1E–5 | 78.8 | 1.1 | 0.5357 | 5.9E–3 | 0.99937 |
| 40 | 0.11772 | 0.01410 | 0.11979 | –5.45E–4 | 5.9E–5 | 77.6 | 1.2 | 0.5565 | 6.6E–3 | 0.99938 |
| 42 | 0.11288 | 0.01237 | 0.10961 | –4.48E–4 | 5.5E–5 | 76.6 | 1.2 | 0.5918 | 7.6E–3 | 0.99934 |
| 44 | 0.10823 | 0.01075 | 0.09932 | –3.61E–4 | 5.1E–5 | 74.5 | 1.2 | 0.6281 | 8.5E–3 | 0.99933 |
| 46 | 0.10367 | 0.00926 | 0.08928 | –2.83E–4 | 4.4E–5 | 70.7 | 1.1 | 0.6611 | 9.1E–3 | 0.99939 |
| 48 | 0.09995 | 0.00827 | 0.08270 | –2.41E–4 | 4.1E–5 | 67.6 | 1.0 | 0.6985 | 9.4E–3 | 0.99952 |
| 50 | 0.09626 | 0.00752 | 0.07808 | –1.90E–4 | 3.4E–5 | 60.89 | 0.85 | 0.7214 | 9.0E–3 | 0.99964 |
| 52 | 0.09235 | 0.00651 | 0.07044 | –1.50E–4 | 3.2E–5 | 56.30 | 0.82 | 0.755 | 1.0E–2 | 0.99964 |

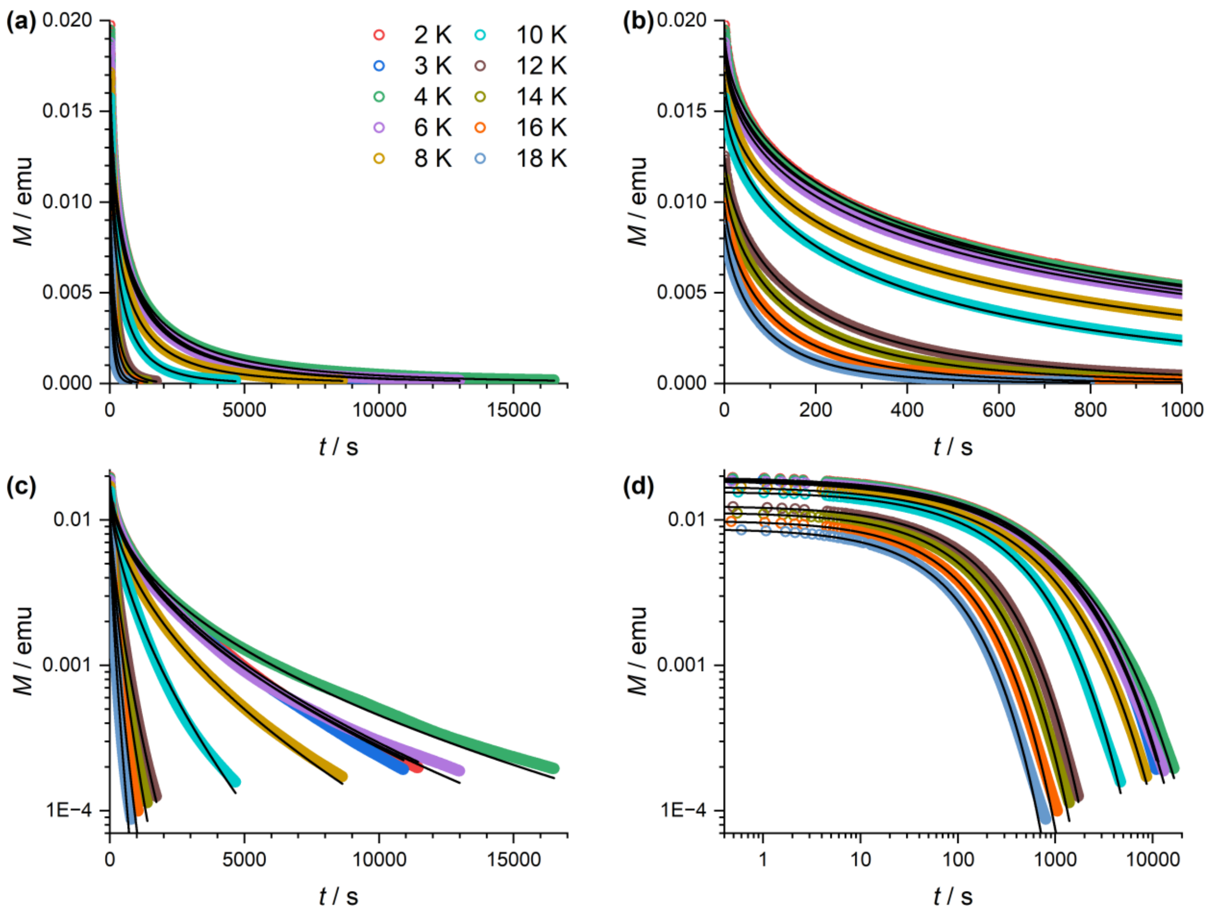


**Figure S145.** Fit of dc magnetisation decay data (2–18 K) for **5%Dy@4-Y** to a single stretched exponential model, with parameters given in Table S30. Represented as (a) linear-linear plot, (b) linear-linear plot at low times, (c) log-linear plot and (d) log-log plot.

**Table S30.** Parameters from fitting magnetization decays of **5%Dy@4-Y** (2–18 K) to stretched exponential function ($M_{eq}$ = 0)

| $T$ (K) | $M_{sat}$ (emu) | $M_0$ (emu) | $M_0/M_{sat}$ | $\tau^*$ (s) | $\tau^{*err}$ (s) | $\beta$ | $\beta^{err}$ | $R^2$ |
|---|---|---|---|---|---|---|---|---|
| 2 | 0.031713 | 0.019759 | 0.62304 | 590.93 | 0.17 | 0.508311 | 9.9E–5 | 0.99953 |
| 3 | 0.031527 | 0.019238 | 0.61021 | 580.05 | 0.14 | 0.509344 | 8.3E–5 | 0.99969 |
| 4 | 0.031521 | 0.019479 | 0.61798 | 594.66 | 0.16 | 0.469151 | 7.9E–5 | 0.99946 |
| 6 | 0.031206 | 0.018790 | 0.60213 | 559.241 | 8.8E–2 | 0.498266 | 5.2E–5 | 0.99985 |
| 8 | 0.030608 | 0.017141 | 0.56001 | 454.035 | 6.0E–2 | 0.526549 | 5.0E–5 | 0.99992 |
| 10 | 0.030096 | 0.015759 | 0.52361 | 337.64 | 0.15 | 0.59565 | 2.2E–4 | 0.99943 |
| 12 | 0.025207 | 0.012538 | 0.49741 | 169.277 | 7.4E–2 | 0.66582 | 2.8E–4 | 0.99973 |
| 14 | 0.027041 | 0.011311 | 0.41830 | 138.643 | 5.0E–2 | 0.68853 | 2.5E–4 | 0.99985 |
| 16 | 0.025410 | 0.009925 | 0.39059 | 106.233 | 4.7E–2 | 0.70933 | 3.3E–4 | 0.99982 |
| 18 | 0.025273 | 0.008727 | 0.34530 | 82.628 | 5.3E–2 | 0.73055 | 5.0E–4 | 0.99972 |

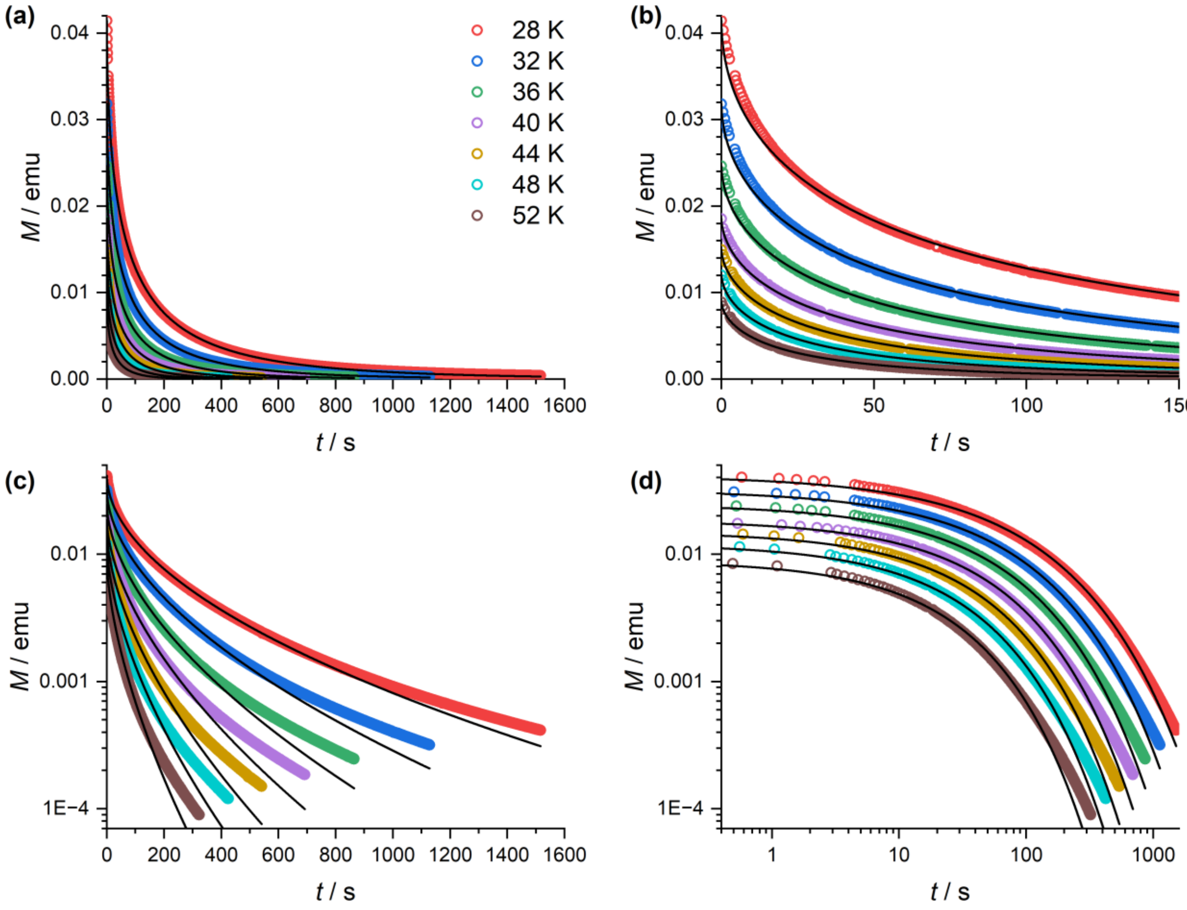


**Figure S146.** Fit of dc magnetisation decay data (28–52 K) for **4-Dy** in benzene to a single stretched exponential model, with parameters given in Table S31. Represented as (a) linear-linear plot, (b) linear-linear plot at low times, (c) log-linear plot and (d) log-log plot.

**Table S31.** Parameters from fitting magnetization decays of **4-Dy** in benzene (28–52 K) to stretched exponential function ($M_{eq}$ = 0)

| $T$ (K) | $M_{sat}$ (emu) | $M_0$ (emu) | $M_0/M_{sat}$ | $\tau^*$ (s) | $\tau^{*err}$ (s) | $\beta$ | $\beta^{err}$ | $R^2$ |
|---|---|---|---|---|---|---|---|---|
| 28 | 0.21637 | 0.04145 | 0.19156 | 73.800 | 9.4E-2 | 0.52532 | 4.7E-4 | 0.99877 |
| 32 | 0.19366 | 0.03180 | 0.16419 | 59.898 | 9.5E-2 | 0.55042 | 6.5E-4 | 0.99851 |
| 36 | 0.17491 | 0.02464 | 0.14085 | 49.123 | 9.5E-2 | 0.57048 | 8.6E-4 | 0.99820 |
| 40 | 0.15934 | 0.01853 | 0.11626 | 42.146 | 8.9E-2 | 0.5908 | 1.0E-3 | 0.99818 |
| 44 | 0.14624 | 0.01497 | 0.10235 | 34.186 | 9.2E-2 | 0.6027 | 1.4E-3 | 0.99759 |
| 48 | 0.13501 | 0.01204 | 0.08919 | 26.946 | 7.9E-2 | 0.6040 | 1.5E-3 | 0.99769 |
| 52 | 0.12546 | 0.00890 | 0.07090 | 22.251 | 8.0E-2 | 0.6250 | 2.0E-3 | 0.99726 |

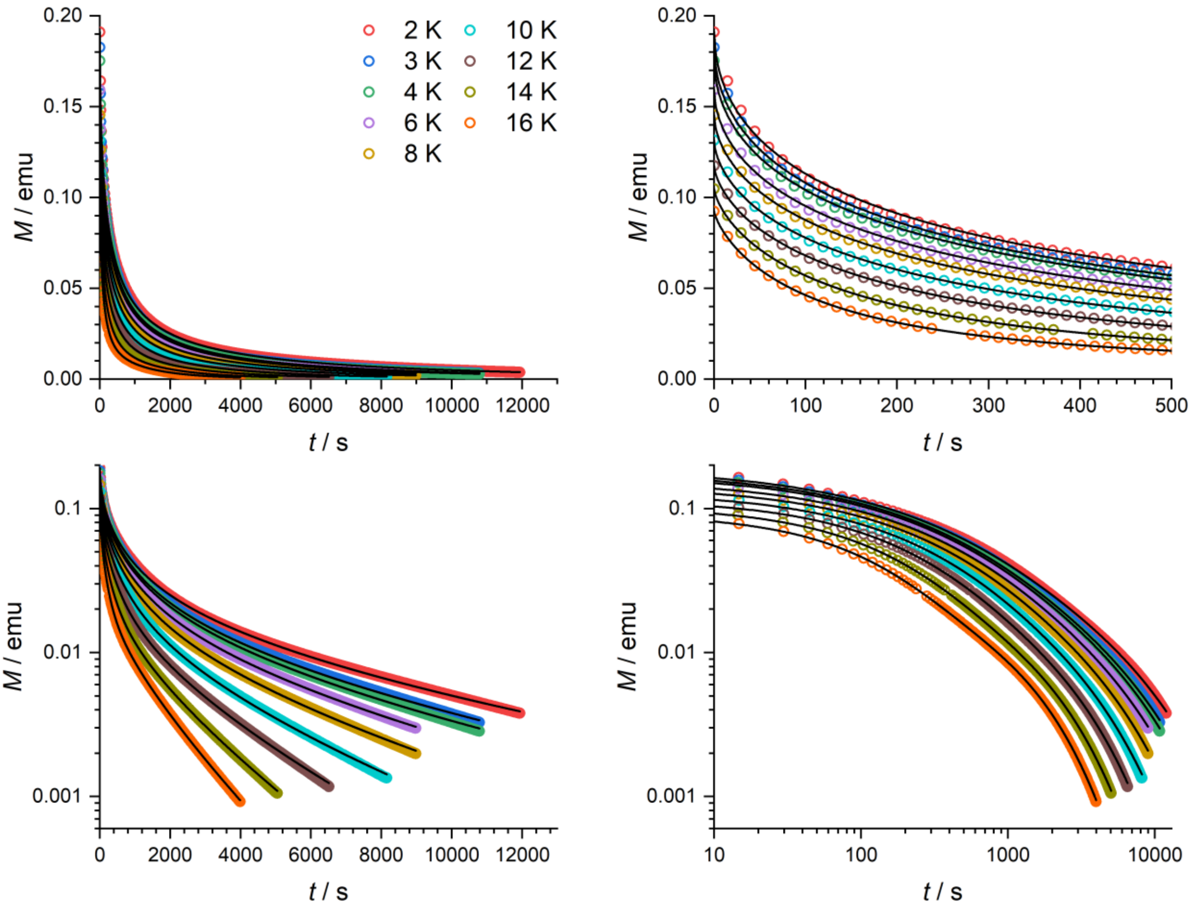


**Figure S147.** Fit of dc magnetisation decay data (2–16 K) for "[{Dy(Cp$^{ttt}$)(Cp*)}{Al[OC($CF_3$)$_3$]$_4$}]" to a sum of two stretched exponentials, with parameters given in Table S32. Represented as (a) linear-linear plot, (b) linear-linear plot at low times, (c) log-linear plot and (d) log-log plot.

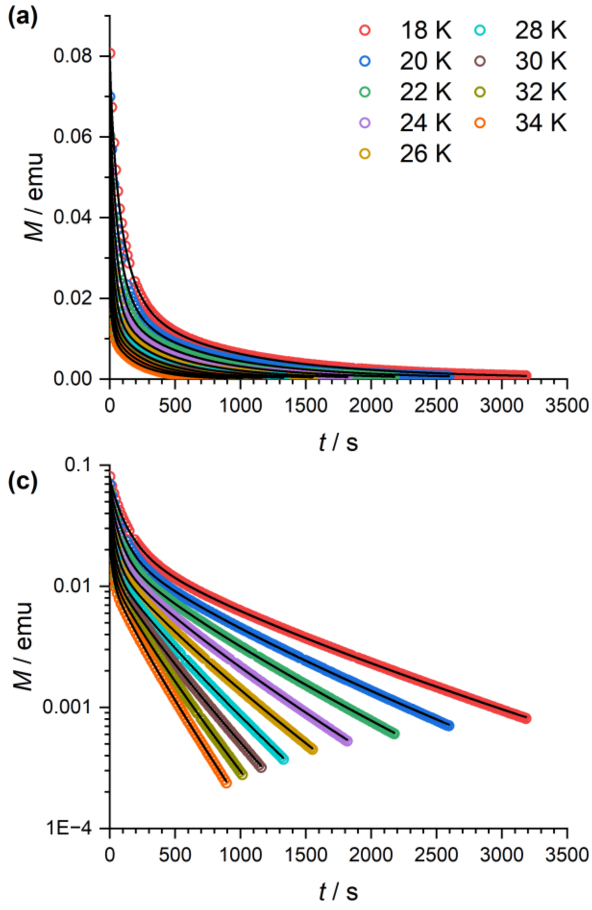


**Figure S148.** Fit of dc magnetisation decay data (18–34 K) for "[{Dy(Cp$^{ttt}$)(Cp*)}{Al[OC($CF_3$)$_3$]$_4$}]" to a sum of two stretched exponentials, with parameters given in Table S32. Represented as (a) linear-linear plot, (b) linear-linear plot at low times, (c) log-linear plot and (d) log-log plot.

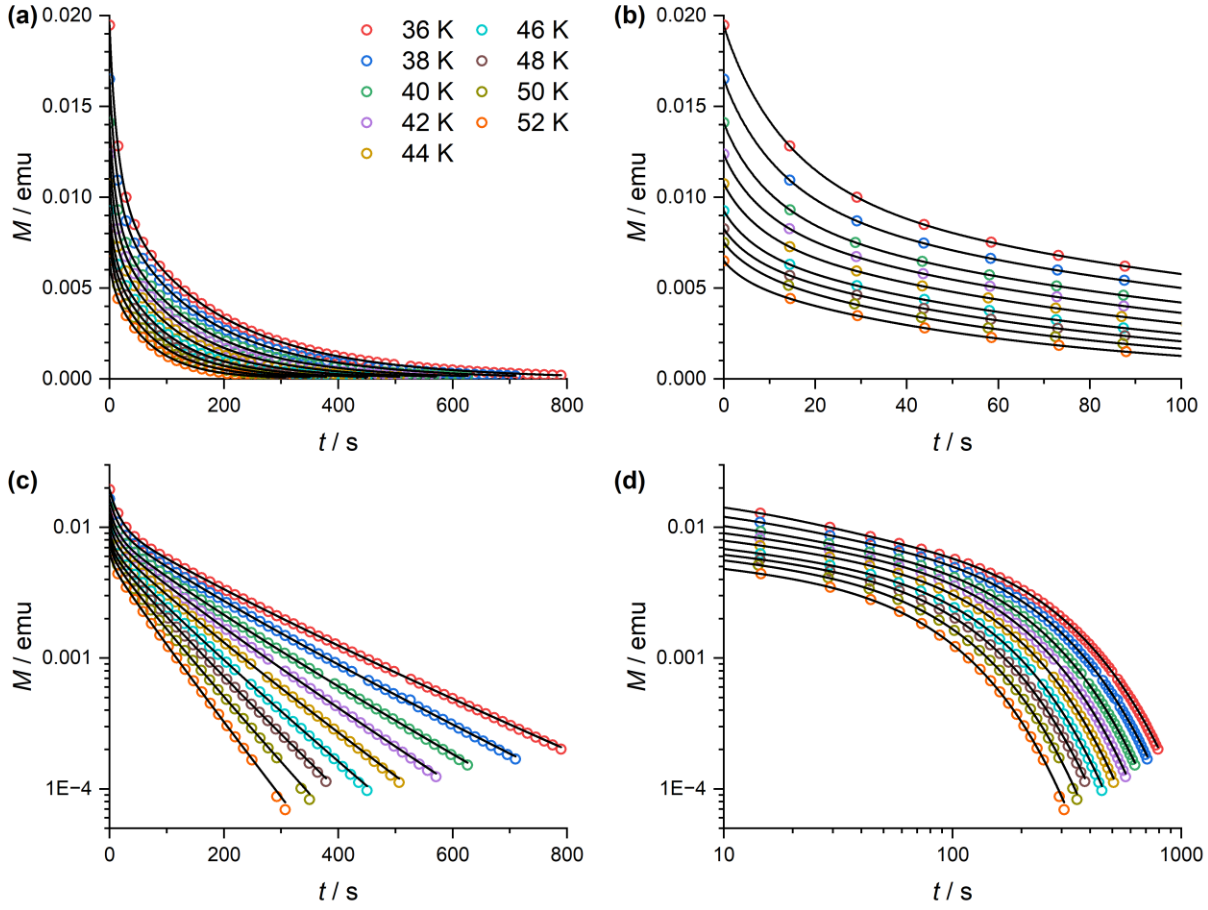


**Figure S149.** Fit of dc magnetisation decay data (36–52 K) for "[{Dy(Cp$^{ttt}$)(Cp*)}{Al[OC($CF_3$)$_3$]$_4$}]" to a sum of two stretched exponentials, with parameters given in Table S32. Represented as (a) linear-linear plot, (b) linear-linear plot at low times, (c) log-linear plot and (d) log-log plot.

**Table S32.** Parameters from fitting magnetization decays of "[{Dy(Cp$^{ttt}$)(Cp*)}{Al[OC($CF_3$)$_3$]$_4$}]" to a sum of two stretched exponential functions

| $T$ | $M_0$ | $M_{eq}$ | $\eta_1$ | $\eta_1^{err}$ | $\tau^*{}_1$ | $\tau^*{}_1^{err}$ | $\beta_1$ | $\beta_1^{err}$ | $\tau^*{}_2$ | $\tau^*{}_2^{err}$ | $\beta_2$ | $\beta_2^{err}$ | $R^2$ |
|---|---|---|---|---|---|---|---|---|---|---|---|---|---|
| (K) | (emu) | (emu) | | | (s) | (s) | | | (s) | (s) | | | |
| 2 | 0.19114 | 0 | 0.7 | 0 | 193.7 | 1.4 | 0.5281 | 1.7E–3 | 1941 | 19 | 0.5447 | 3.8E–3 | 0.99964 |
| 3 | 0.18264 | 0 | 0.7 | 0 | 195.6 | 1.7 | 0.5373 | 1.8E–3 | 1676 | 20 | 0.5508 | 4.5E–3 | 0.99966 |
| 4 | 0.17532 | 0 | 0.7 | 0 | 197.0 | 1.5 | 0.5429 | 1.7E–3 | 1640 | 18 | 0.5613 | 4.1E–3 | 0.99972 |
| 6 | 0.15932 | 0 | 0.7 | 0 | 200.6 | 1.6 | 0.5576 | 1.6E–3 | 1525 | 17 | 0.5723 | 4.6E–3 | 0.99979 |
| 8 | 0.14543 | 0 | 0.7 | 0 | 197.1 | 1.2 | 0.5827 | 1.4E–3 | 1418 | 12 | 0.6034 | 3.6E–3 | 0.99985 |
| 10 | 0.13146 | 0 | 0.7 | 0 | 179.44 | 0.92 | 0.6097 | 1.5E–3 | 1226.9 | 8.8 | 0.6336 | 3.4E–3 | 0.99987 |
| 12 | 0.11744 | 0 | 0.7 | 0 | 158.12 | 0.53 | 0.6607 | 1.3E–3 | 1079.5 | 4.8 | 0.6720 | 2.6E–3 | 0.99993 |
| 14 | 0.10477 | 0 | 0.7 | 0 | 126.65 | 0.26 | 0.7141 | 1.1E–3 | 914.6 | 2.3 | 0.7089 | 1.8E–3 | 0.99996 |
| 16 | 0.09246 | 0 | 0.7 | 0 | 100.17 | 0.13 | 0.76976 | 9.0E–4 | 787.9 | 1.1 | 0.7510 | 1.2E–3 | 0.99998 |
| 18 | 0.08071 | 0 | 0.6787 | 3.8E–3 | 77.80 | 0.23 | 0.8219 | 1.6E–3 | 629.5 | 9.2 | 0.7623 | 5.4E–3 | 0.99999 |
| 20 | 0.06994 | 0 | 0.6645 | 3.0E–3 | 61.30 | 0.16 | 0.8594 | 1.6E–3 | 526.7 | 5.8 | 0.7825 | 4.5E–3 | 0.99999 |
| 22 | 0.06027 | 0 | 0.6474 | 1.9E–3 | 49.180 | 8.5E–2 | 0.8856 | 1.2E–3 | 449.4 | 2.9 | 0.8011 | 2.9E–3 | 1.00000 |
| 24 | 0.05156 | 0 | 0.6238 | 1.5E–3 | 39.736 | 5.9E–2 | 0.9068 | 1.2E–3 | 379.4 | 1.8 | 0.8145 | 2.2E–3 | 1.00000 |
| 26 | 0.04390 | 0 | 0.6051 | 1.3E–3 | 32.703 | 5.1E–2 | 0.9196 | 1.3E–3 | 330.6 | 1.4 | 0.8346 | 2.1E–3 | 1.00000 |
| 28 | 0.03692 | 0 | 0.58723 | 9.3E–4 | 27.221 | 3.3E–2 | 0.9199 | 1.0E–3 | 289.30 | 0.78 | 0.8557 | 1.4E–3 | 1.00000 |
| 30 | 0.03138 | 0 | 0.5500 | 1.3E–3 | 22.909 | 4.1E–2 | 0.9388 | 1.8E–3 | 248.24 | 0.87 | 0.8602 | 1.9E–3 | 1.00000 |
| 32 | 0.02667 | 0 | 0.5139 | 1.1E–3 | 19.474 | 3.4E–2 | 0.9366 | 1.9E–3 | 216.46 | 0.62 | 0.8685 | 1.6E–3 | 1.00000 |
| 34 | 0.02277 | 0 | 0.48005 | 8.0E–4 | 16.624 | 2.2E–2 | 0.9360 | 1.6E–3 | 189.78 | 0.35 | 0.8760 | 1.0E–3 | 1.00000 |
| 36 | 0.01947 | 0 | 0.4448 | 1.0E–3 | 14.300 | 2.7E–2 | 0.9314 | 2.5E–3 | 168.20 | 0.37 | 0.8880 | 1.3E–3 | 1.00000 |
| 38 | 0.01650 | 0 | 0.3875 | 1.7E–3 | 12.387 | 4.4E–2 | 0.9380 | 5.7E–3 | 148.37 | 0.50 | 0.8928 | 2.0E–3 | 1.00000 |
| 40 | 0.01410 | 0 | 0.3515 | 1.3E–3 | 10.725 | 3.0E–2 | 0.9238 | 5.2E–3 | 132.39 | 0.30 | 0.9010 | 1.3E–3 | 1.00000 |
| 42 | 0.01237 | 0 | 0.3079 | 1.8E–3 | 9.405 | 4.4E–2 | 0.9081 | 9.7E–3 | 118.58 | 0.35 | 0.9099 | 1.8E–3 | 1.00000 |
| 44 | 0.01075 | 0 | 0.2627 | 3.0E–3 | 8.329 | 9.2E–2 | 0.939 | 2.6E–2 | 105.56 | 0.49 | 0.9140 | 2.8E–3 | 1.00000 |
| 46 | 0.00926 | 0 | 0.2317 | 2.0E–3 | 7.491 | 9.3E–2 | 0.94 | 0 | 94.68 | 0.30 | 0.9224 | 2.2E–3 | 1.00000 |
| 48 | 0.00827 | 0 | 0.1932 | 2.1E–3 | 6.67 | 0.11 | 0.94 | 0 | 84.51 | 0.27 | 0.9264 | 2.1E–3 | 1.00000 |
| 50 | 0.00752 | 0 | 0.1863 | 3.6E–3 | 6.83 | 0.17 | 0.94 | 0 | 75.51 | 0.38 | 0.9359 | 3.5E–3 | 1.00000 |
| 52 | 0.00651 | 0 | 0.1576 | 4.9E–3 | 5.38 | 0.30 | 0.94 | 0 | 66.95 | 0.45 | 0.9483 | 4.8E–3 | 1.00000 |

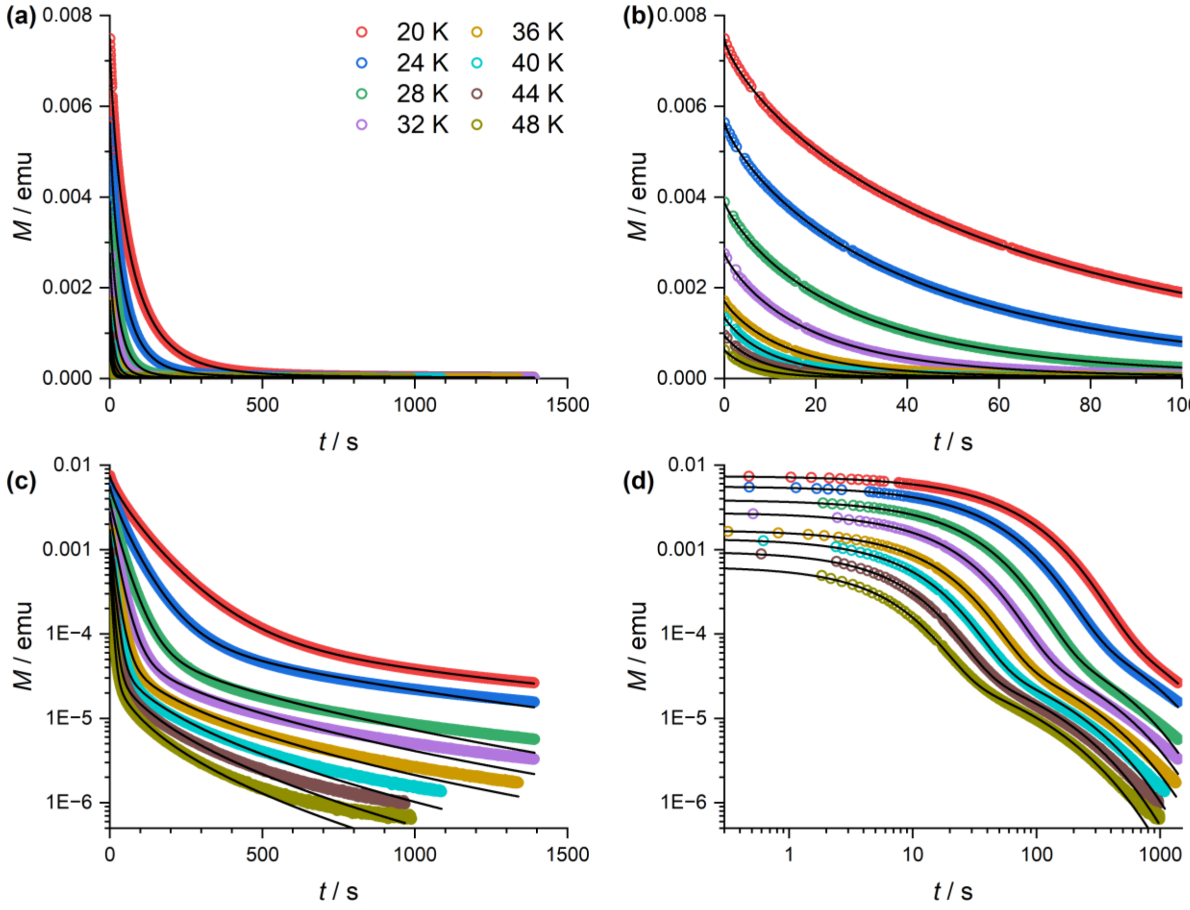


**Figure S150.** Fit of dc magnetisation decay data (20–48 K) for **5%Dy@4-Y** to sum of two stretched exponentials, with parameters given in Table S33. Represented as (a) linear-linear plot, (b) linear-linear plot at low times, (c) log-linear plot and (d) log-log plot.

**Table S33.** Parameters from fitting magnetization decays of **5%Dy@4-Y** (20–48 K) to a sum of two stretched exponential functions ($M_{eq}$ = 0)

| $T$ | $M_{sat}$ | $M_0$ | $M_0/M_{sat}$ | $\eta_1$ | $\eta_1^{err}$ | $\tau^*{}_1$ | $\tau^*{}_1^{err}$ | $\beta_1$ | $\beta_1^{err}$ | $\tau^*{}_2$ | $\tau^*{}_2^{err}$ | $\beta_2$ | $\beta_2^{err}$ | $R^2$ |
|---|---|---|---|---|---|---|---|---|---|---|---|---|---|---|
| (K) | (emu) | (emu) | | | | (s) | (s) | | | (s) | (s) | | | |
| 20 | 0.024186 | 0.007494 | 0.30984 | 0.9412 | 1.3E–3 | 64.531 | 4.1E–2 | 0.79462 | 6.0E–4 | 123.4 | 3.2 | 0.4301 | 2.3E–3 | 0.99998 |
| 24 | 0.023356 | 0.005639 | 0.24144 | 0.9588 | 1.9E–3 | 41.902 | 2.2E–2 | 0.82499 | 7.4E–4 | 204 | 15 | 0.544 | 1.3E–2 | 0.99999 |
| 28 | 0.012196 | 0.003889 | 0.31887 | 0.9583 | 2.7E–3 | 27.588 | 2.0E–2 | 0.8649 | 1.2E–3 | 127 | 12 | 0.548 | 1.6E–2 | 0.99996 |
| 32 | 0.010374 | 0.002754 | 0.26549 | 0.9514 | 2.9E–3 | 19.128 | 2.1E–2 | 0.8845 | 1.5E–3 | 83.6 | 8.2 | 0.503 | 1.4E–2 | 0.99992 |
| 36 | 0.010575 | 0.001713 | 0.16196 | 0.9262 | 1.7E–3 | 14.330 | 1.4E–2 | 0.9184 | 1.2E–3 | 46.5 | 1.9 | 0.4584 | 4.4E–3 | 0.99994 |
| 40 | 0.009226 | 0.001355 | 0.14690 | 0.9353 | 3.5E–3 | 10.487 | 1.3E–2 | 0.9293 | 2.1E–3 | 50.0 | 4.6 | 0.498 | 1.3E–2 | 0.99986 |
| 44 | 0.008352 | 9.57E–4 | 0.11463 | 0.9231 | 4.1E–3 | 8.105 | 1.3E–2 | 0.9484 | 2.7E–3 | 38.3 | 3.6 | 0.489 | 1.2E–2 | 0.99979 |
| 48 | 0.007580 | 6.36E–4 | 0.08396 | 0.8977 | 5.8E–3 | 6.384 | 1.7E–2 | 0.9712 | 4.2E–3 | 26.3 | 2.7 | 0.464 | 1.2E–2 | 0.99965 |

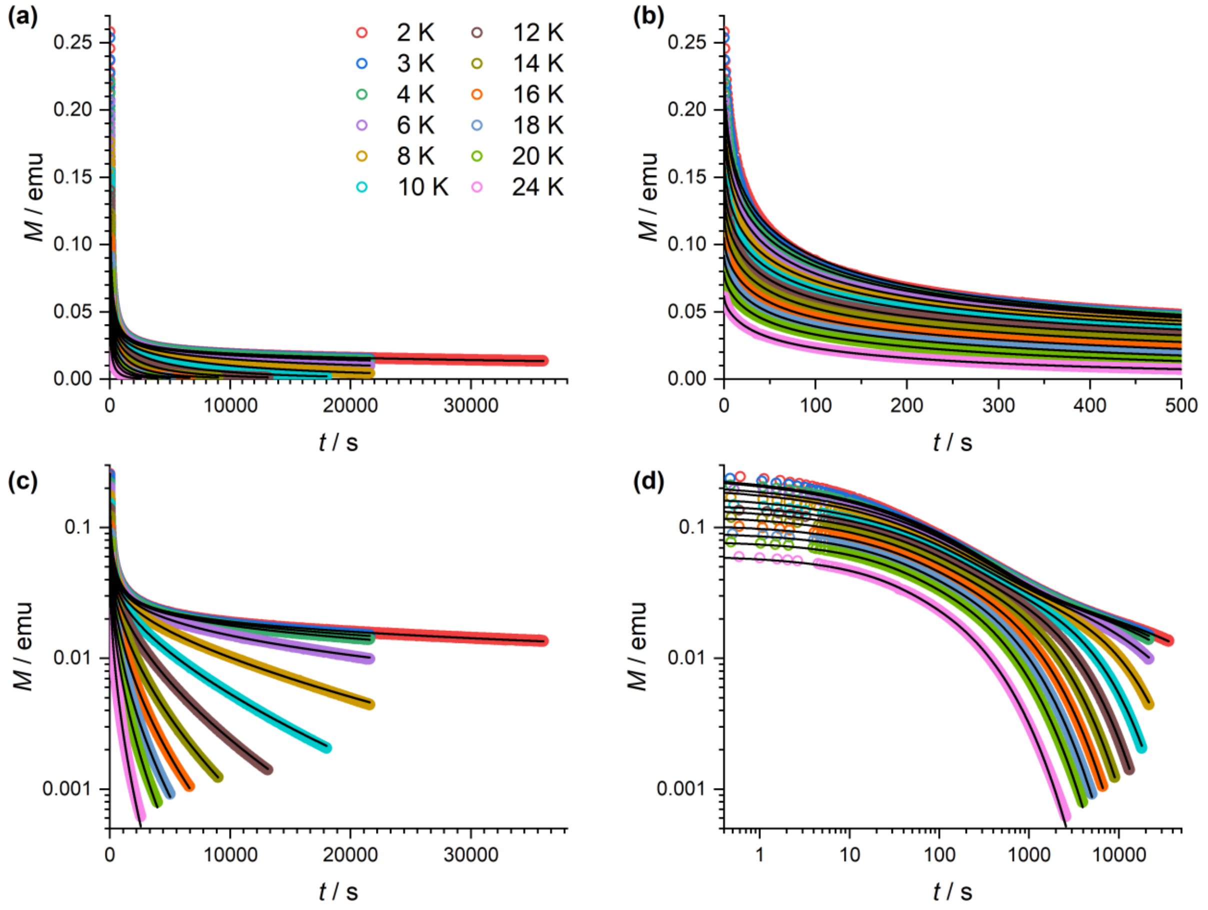


**Figure S151.** Fit of dc magnetisation decay data (2–24 K) for **4-Dy** in benzene to sum of two stretched exponentials, with parameters given in Table S34. Represented as (a) linear-linear plot, (b) linear-linear plot at low times, (c) log-linear plot and (d) log-log plot.

**Table S34.** Parameters from fitting magnetization decays of **4-Dy** in benzene (2–24 K) to a sum of two stretched exponential functions ($M_{eq}$ = 0)

| $T$ | $M_{sat}$ | $M_0$ | $M_0/M_{sat}$ | $\eta_1$ | $\eta_1^{err}$ | $\tau^*{}_1$ | $\tau^*{}_1^{err}$ | $\beta_1$ | $\beta_1^{err}$ | $\tau^*{}_2$ | $\tau^*{}_2^{err}$ | $\beta_2$ | $\beta_2^{err}$ | $R^2$ |
|---|---|---|---|---|---|---|---|---|---|---|---|---|---|---|
| (K) | (emu) | (emu) | | | | (s) | (s) | | | (s) | (s) | | | |
| 2 | 0.49863 | 0.25819 | 0.51781 | 0.7969 | 3.3E-3 | 47.12 | 0.13 | 0.39328 | 8.0E-4 | 9.57E+3 | 6.7E+2 | 0.2296 | 3.3E-3 | 0.99752 |
| 3 | 0.50017 | 0.23749 | 0.47483 | 0.7894 | 5.5E-3 | 41.71 | 0.14 | 0.3896 | 1.2E-3 | 7.96E+3 | 8.5E+2 | 0.2473 | 5.9E-3 | 0.99815 |
| 4 | 0.49931 | 0.22095 | 0.44251 | 0.7578 | 4.9E-3 | 52.53 | 0.14 | 0.4152 | 1.2E-3 | 7.06E+3 | 5.4E+2 | 0.2663 | 4.8E-3 | 0.99826 |
| 6 | 0.49736 | 0.20619 | 0.41456 | 0.7316 | 1.6E-3 | 43.841 | 5.0E-2 | 0.42636 | 4.7E-4 | 4538 | 81 | 0.3384 | 1.6E-3 | 0.99919 |
| 8 | 0.49123 | 0.17647 | 0.35925 | 0.78642 | 4.6E-4 | 55.617 | 8.4E-2 | 0.43919 | 3.0E-4 | 6430 | 23 | 0.6122 | 1.1E-3 | 0.99947 |
| 10 | 0.48560 | 0.15421 | 0.31756 | 0.71643 | 5.2E-4 | 51.720 | 7.9E-2 | 0.48099 | 3.1E-4 | 2988.9 | 8.7 | 0.61438 | 7.6E-4 | 0.99974 |
| 12 | 0.47504 | 0.14037 | 0.29549 | 0.61512 | 6.8E-4 | 38.562 | 6.4E-2 | 0.51594 | 3.9E-4 | 1354.6 | 4.0 | 0.56782 | 6.0E-4 | 0.99985 |
| 14 | 0.46480 | 0.12331 | 0.26529 | 0.49039 | 8.1E-4 | 30.427 | 4.8E-2 | 0.56246 | 5.1E-4 | 696.0 | 1.9 | 0.53514 | 4.7E-4 | 0.99993 |
| 16 | 0.45532 | 0.10494 | 0.23047 | 0.35071 | 8.9E-4 | 28.140 | 3.6E-2 | 0.64892 | 8.7E-4 | 407.53 | 0.99 | 0.51511 | 3.9E-4 | 0.99996 |
| 18 | 0.44535 | 0.09179 | 0.20610 | 0.2474 | 1.1E-3 | 25.662 | 3.4E-2 | 0.7382 | 1.8E-3 | 269.57 | 0.71 | 0.50488 | 4.0E-4 | 0.99996 |
| 20 | 0.43372 | 0.07941 | 0.18308 | 0.1676 | 1.5E-3 | 26.764 | 6.2E-2 | 0.8733 | 4.8E-3 | 199.06 | 0.62 | 0.50424 | 4.7E-4 | 0.99995 |
| 24 | 0.40684 | 0.06158 | 0.15136 | 0.1171 | 3.3E-3 | 26.79 | 0.25 | 0.988 | 1.8E-2 | 133.02 | 0.84 | 0.51908 | 9.7E-4 | 0.99985 |

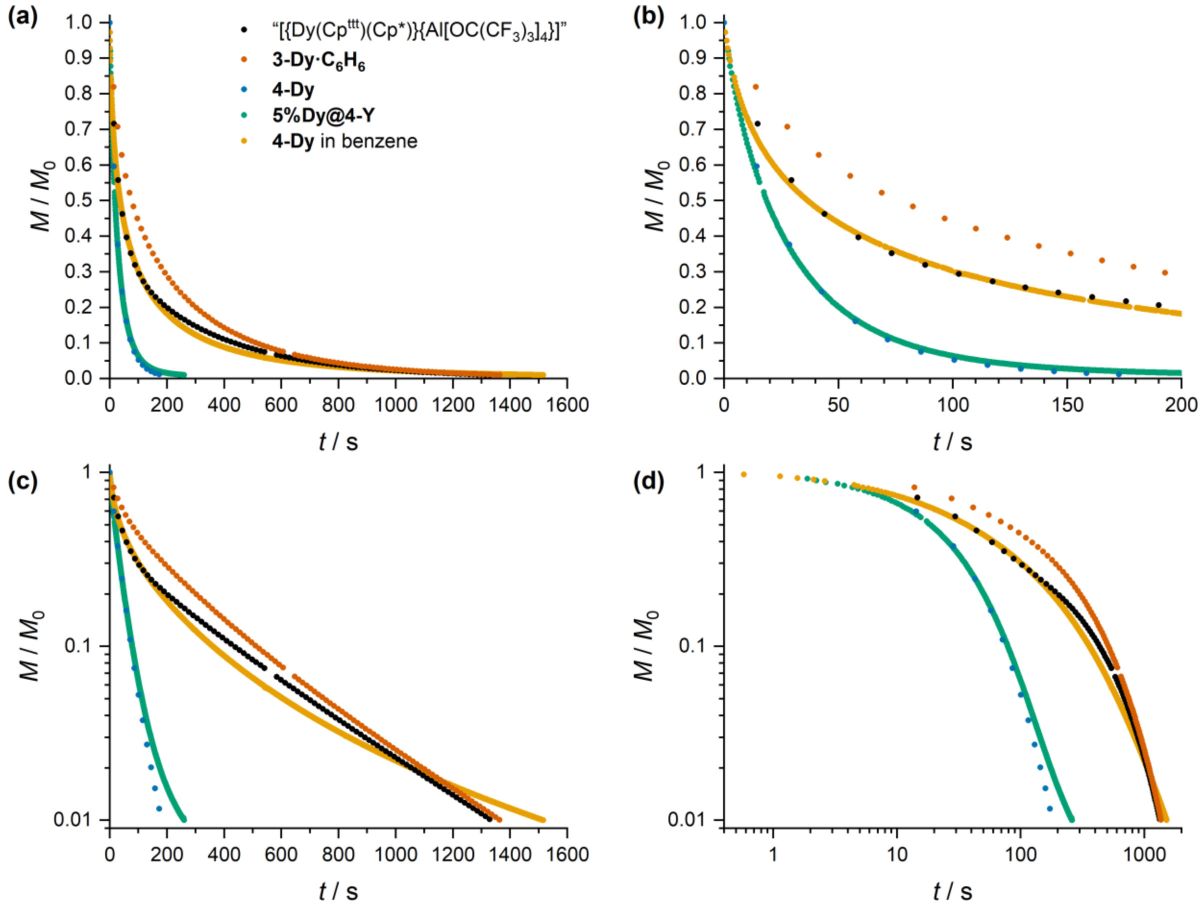


**Figure S152.** Comparison of 28 K magnetization decay curves (Raman region) for “[{Dy(Cp$^{ttt}$)(Cp*)}{Al[OC($CF_3$)$_3$]$_4$}]” (black), **3-Dy·$C_6H_6$** (red), **4-Dy** (blue), **5%Dy@4-Y** (green) and **4-Dy** in benzene (yellow) normalized to $M_0$. Represented as (a) linear-linear plot, (b) linear-linear plot at low times, (c) log-linear plot and (d) log-log plot.

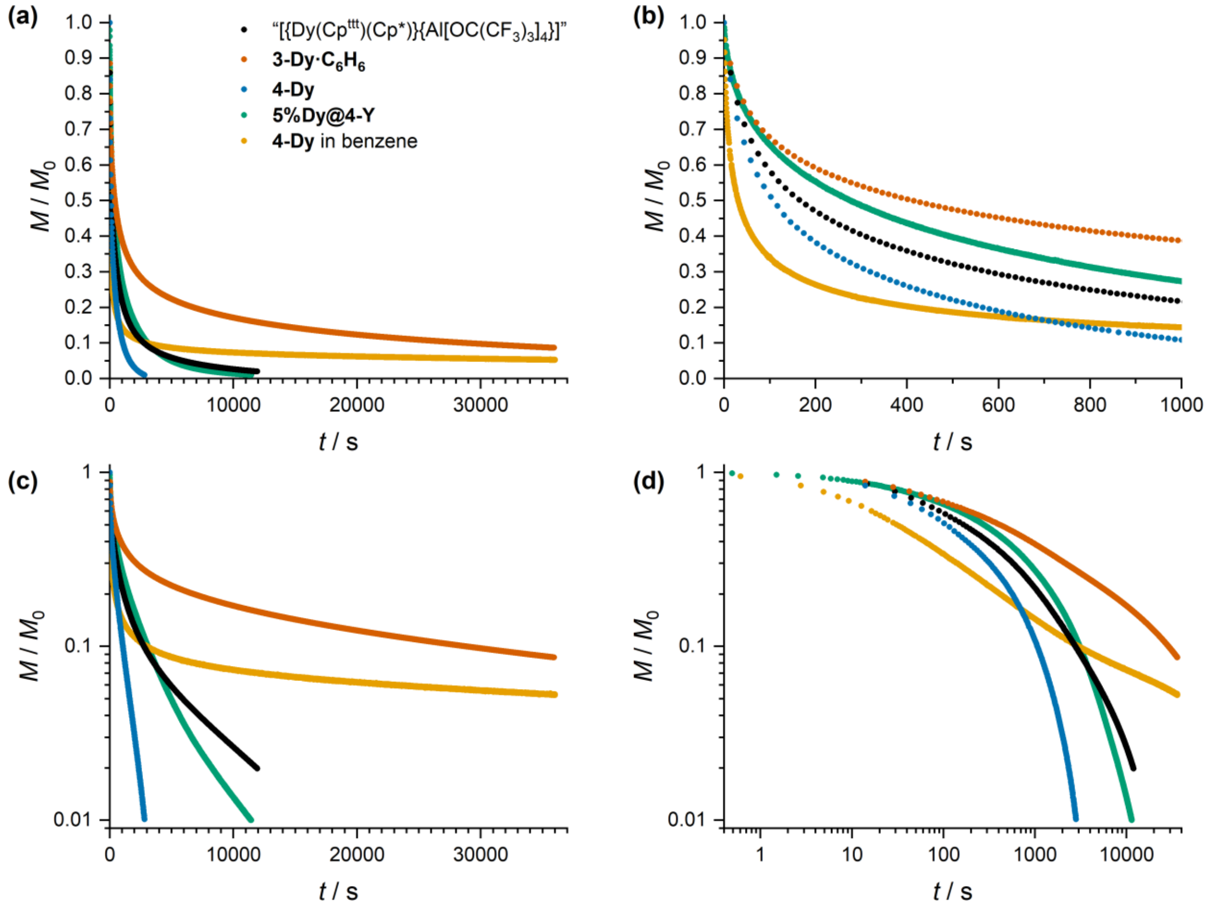


**Figure S153.** Comparison of 2 K magnetization decay curves (QTM region) for “[{Dy(Cp$^{ttt}$)(Cp*)}{Al[OC($CF_3$)$_3$]$_4$}]” (black), **3-Dy·$C_6H_6$** (red), **4-Dy** (blue), **5%Dy@4-Y** (green) and **4-Dy** in benzene (yellow) normalized to $M_0$. Represented as (a) linear-linear plot, (b) linear-linear plot at low times, (c) log-linear plot and (d) log-log plot.

*9.5 Relaxation Profiles*

The representative time and width of the relaxation time distributions are defined using the expectation value ($\langle \ln \tau \rangle$) and variance ($\sigma^2_{\ln \tau}$) of the distribution of logarithmic relaxation times.[39] These values are calculated for relaxation time distributions characterized by different empirical formulae using equations given by Zorn.[43]

For a generalized Debye model:

$$\langle \ln \tau \rangle = \ln \tau_{\mathrm{debye}}$$

$$\sigma^2_{\ln \tau} = \frac{\pi^2}{3}\left(\frac{1}{(1-\alpha)^2} - 1\right)$$

Equation S5

For a stretched exponential function:

$$\langle \ln \tau \rangle = \ln \tau^* + \left(1 - \frac{1}{\beta}\right) \mathrm{Eu}$$

$$\sigma^2_{\ln \tau} = \frac{\pi^2}{6}\left(\frac{1}{\beta^2} - 1\right)$$

Equation S6

where Eu is Euler's constant, 0.5772…. The representative (central) value of τ is given by $e^{\langle \ln \tau \rangle}$ and the one estimated standard deviation (ESD) upper and lower values are given by:[39]

$$\tau_{\pm} = e^{\langle \ln \tau \rangle \pm \sqrt{\sigma^2_{\ln \tau}}}$$

Equation S7

Values of $e^{\langle \ln \tau \rangle}$, $\tau_{\pm}$, $\sigma^2_{\ln \tau}$ and the uncertainty in $e^{\langle \ln \tau \rangle}$ are compared across temperatures and measurement methods in Tables S35–S39. We stress that the one ESD values represent the distributions in relaxation times and are distinct from the uncertainty in the central relaxation time.

The temperature dependence of the relaxation rates ($\tau^{-1}$) are be modelled by:

$$\tau^{-1} = {\tau_0}^{-1} e^{-\left(\frac{U_{\text{eff}}}{k_{\text{B}}T}\right)} + CT^n + {\tau_{\text{QTM}}}^{-1}$$

Equation S8

Where ${\tau_0}^{-1}$ is the Orbach prefactor ($\tau_0$ = $10^A$ s), $U_{\text{eff}}$ is the effective energy barrier for the Orbach process, *C* (*C* = $10^R$ $s^{-1}$ $K^{-n}$) and *n* are phenomenological parameters that describe the Raman process, and ${\tau_{\text{QTM}}}^{-1}$ is the quantum tunnelling of magnetisation (QTM) rate ($\tau_{\text{QTM}}$ = $10^Q$ s). As relaxation rates exist on a logarithmic scale, the logarithmic version of Equation S8 (Equation S9) with *A*, *R*, *n*, *Q* and $U_{\text{eff}}$ as fitting parameters was used to fit the temperature dependence of the average logarithmic relaxation rate (equal to $-\langle \ln \tau \rangle / \ln[10]$).

$$\log_{10}[\tau^{-1}] = \log_{10}\left[10^{-A} e^{-\left(\frac{U_{\text{eff}}}{k_{\text{B}}T}\right)} + 10^R T^n + 10^{-Q}\right]$$

Equation S9

which was performed in CC-FIT2,[38,39] using updated expressions for the distributions in fitted parameters.

There is a discrepancy between waveform- and dc decay-derived rates and their distributions for **3-Dy·$C_6H_6$** in the 32–56 K region (Figure S154). This arises due to the very broad distribution of rates: during removal of the initial magnetic field and wait for a stable field in a dc decay experiment, the faster-relaxing components of the distribution equilibrate, thus biasing the observed decay towards longer relaxation times. The waveform data are not subject to this issue as switching the field between ± 8 Oe is comparatively fast and the Fourier transform captures the fast-relaxing components; hence, these data are more reliable[39] (note the excellent agreement between ac- and waveform-derived rates and distributions for **3-Dy·$C_6H_6$** at ca. 60 K). The magnetization-derived rates at 32–56 K are too slow, and the distributions are too narrow and become even narrower with increasing temperature. In contrast waveform data show consistent values of $\sigma^2_{\ln \tau}$ across the Raman-dominated regime. Therefore, for the relaxation profile of **3-Dy·$C_6H_6$**, relaxation rates were fit to Equation S9 (Figure S154) using

all ac data (60–105 K), all waveform measurements (32–56 K) and low temperature magnetization decays where waveform measurements were not recorded as they were too slow (2–28 K). We recommend using waveform measurements to confirm rates where broad distributions are expected, or where there is an unexpected decrease in distribution width with increasing rates.

For the relaxation profile of **4-Dy**, relaxation rates were fit to Equation S9 (Figure S155) using all ac data (60–108 K), all low temperature magnetization decays (2–38 K) and waveform measurements where magnetization decays could not be recorded (40–54 K). The central rates for decay and waveform data agree very well for **4-Dy**, this is because the distribution of relaxation rates is much narrower than **3-Dy·$C_6H_6$** and the decay measurements are not detrimentally affected by the delay of the field sweeping to zero. Magnetization decays were favoured over waveform measurements in fitting as the wider decay distributions were more consistent with the low temperature ac distributions. The waveform measurements at 40–54 K underestimate the width of the distribution (α values near zero) as only the low-frequency side of the $\chi_M''$ peak is accessible experimentally. Values of $\sigma^2_{\ln\tau}$ for waveform data converge to the values from magnetization decays at lower temperatures as more of the high-frequency tail is observed. To not over-bias waveform datapoints (40–54 K) in the fitting of the relaxation profile, α was fixed to 0.05 for these points (Figure S155), selected to give a similar $\sigma^2_{\ln\tau}$ to the magnetization decays at 26–38 K.

For the relaxation profile of **5%Dy@4-Y**, relaxation rates were fit to Equation S9 (Figure S156) using all ac data (66–102 K), the majority component (≥ 90%) of the magnetisation decays when fit with a sum of two stretched exponentials (20–48 K) and the low temperature magnetisation decays which were fit with a single stretched exponential function (2–18 K). The rates of both components of **5%Dy@4-Y** are compared to the simulated curves for **3-Dy·$C_6H_6$** and **4-Dy** in Figure S157. We did not fit the relaxation profile of **4-Dy** in

benzene as there was not a clear majority component. Instead, the rates of **4-Dy** in benzene are compared to the simulated curves for **3-Dy·$C_6H_6$** and **4-Dy** (Figure S158).

For "[{Dy($Cp^{ttt}$)(Cp*)}{Al[OC($CF_3$)$_3$]$_4$}]", most of the sample appeared to be fast-relaxing, so we fit relaxation data for the fast-relaxing component of magnetization decays (2–52 K) and the ac data (63–108 K) to Equation S9 (Figure S159 and Table S40). The slow-relaxing minor component of "[{Dy($Cp^{ttt}$)(Cp*)}{Al[OC($CF_3$)$_3$]$_4$}]" was discontinuous with ac data and was fit to a relaxation equation for QTM and Raman processes:

$$\log_{10}[\tau^{-1}] = \log_{10}[10^{R}T^{n} + 10^{-Q}]$$

Equation S10

The Orbach parameters for "[{Dy($Cp^{ttt}$)(Cp*)}{Al[OC($CF_3$)$_3$]$_4$}]" ($U_{\mathrm{eff}}$ = 1220(22) $cm^{-1}$, $\tau_0 = 10^{-11.0(2)}$ s) are near identical to those for **3-Dy·$C_6H_6$** ($U_{\mathrm{eff}}$ = 1221(25) $cm^{-1}$, $\tau_0 = 10^{-11.0(2)}$ s) and are similar to those for **4-Dy** ($U_{\mathrm{eff}}$ = 1265(15) $cm^{-1}$, $\tau_0 = 10^{-11.4(1)}$ s). The rates for the fast component of "[{Dy($Cp^{ttt}$)(Cp*)}{Al[OC($CF_3$)$_3$]$_4$}]" and for **4-Dy** agree in the low temperature region (Figure S164c), reflected in Raman and QTM parameters that are within error (fast component of "[{Dy($Cp^{ttt}$)(Cp*)}{Al[OC($CF_3$)$_3$]$_4$}]": $C = 10^{-6.0(2)}$ $s^{-1}$ $K^{-n}$, $n$ = 3.1(2), $\tau_{\mathrm{QTM}} = 10^{2.12(6)}$ s; **4-Dy**: $C = 10^{-6.4(3)}$ $s^{-1}$ $K^{-n}$, $n$ = 3.4(2), $\tau_{\mathrm{QTM}} = 10^{2.13(7)}$ s). This indicates that the CIP is the major component (70% magnetization at low temperature) of "[{Dy($Cp^{ttt}$)(Cp*)}{Al[OC($CF_3$)$_3$]$_4$}]". The Raman rates for the slow component of "[{Dy($Cp^{ttt}$)(Cp*)}{Al[OC($CF_3$)$_3$]$_4$}]" are $C = 10^{-6.2(4)}$ $s^{-1}$ $K^{-n}$, $n$ = 2.54(3) while the tunneling time is $\tau_{\mathrm{QTM}} = 10^{3.040(9)}$ s, compared to $\tau_{\mathrm{QTM}} = 10^{2.54(7)}$ s for **3-Dy·$C_6H_6$**. The rates are similar although slightly slower for the minor component of "[{Dy($Cp^{ttt}$)(Cp*)}{Al[OC($CF_3$)$_3$]$_4$}]" compared to **3-Dy·$C_6H_6$**. We are cautious not to over-interpret the relaxation parameters and apparent slower rates given the co-fitting of a fast component, and potential biasing of magnetization decay measurements. We note that the magnetization decay-derived rates for

both the minor component of "[{Dy(Cp$^{ttt}$)(Cp*)}{Al[OC($CF_3$)$_3$]$_4$}]" and **3-Dy·$C_6H_6$** have a narrowing of distributions on increasing temperature (Figure S164d).

The hysteresis temperature and $T_{irrev}$ blocking temperature for "[{Dy(Cp$^{ttt}$)(Cp*)}{Al[OC($CF_3$)$_3$]$_4$}]" ($T_H$ = 50 K, $T_{irrev}$ = 37 K) lie between **4-Dy** ($T_H$ = 36 K, $T_{irrev}$ = 28 K) and **3-Dy·$C_6H_6$** ($T_H$ = 52 K, $T_{irrev}$ = 40 K). These data support the CIP and SIP geometry both being present in "[{Dy(Cp$^{ttt}$)(Cp*)}{Al[OC($CF_3$)$_3$]$_4$}]".

**Table S35.** Relaxation times and distributions for **3-Dy·$C_6H_6$**

| T (K) | $\tau_-$ (s) | $e^{\langle \ln \tau \rangle}$ (s) | $\tau_+$ (s) | $e^{\langle \ln \tau \rangle^{\text{err}}}$ (s) | $\sigma^2_{\ln \tau}$ | Fit? |
|---|---|---|---|---|---|---|
| **Magnetization Decays** | | | | | | |
| 2 | 2.652 | 259.0 | 25294 | 2.3 | 20.99 | Y |
| 3 | 3.554 | 277.7 | 21693 | 2.9 | 18.99 | Y |
| 4 | 4.863 | 306.4 | 19309 | 2.6 | 17.17 | Y |
| 6 | 11.83 | 387.6 | 12698 | 2.1 | 12.18 | Y |
| 8 | 19.54 | 431.7 | 9537 | 1.7 | 9.580 | Y |
| 10 | 28.12 | 407.9 | 5918 | 1.2 | 7.153 | Y |
| 12 | 33.06 | 356.8 | 3850 | 1.0 | 5.659 | Y |
| 14 | 33.47 | 297.2 | 2639 | 1.0 | 4.769 | Y |
| 16 | 31.68 | 244.8 | 1891 | 1.0 | 4.180 | Y |
| 18 | 29.14 | 203.2 | 1416 | 1.0 | 3.770 | Y |
| 20 | 26.79 | 172.01 | 1105 | 0.94 | 3.458 | Y |
| 24 | 23.16 | 129.00 | 718.7 | 0.74 | 2.950 | Y |
| 28 | 21.61 | 103.24 | 493.3 | 0.51 | 2.446 | Y |
| 32 | 20.53 | 85.78 | 358.5 | 0.32 | 2.045 | N |
| 36 | 19.91 | 73.19 | 269.1 | 0.18 | 1.695 | N |
| 40 | 20.24 | 64.570 | 206.0 | 0.075 | 1.346 | N |
| 44 | 19.31 | 55.689 | 160.6 | 0.061 | 1.122 | N |
| 48 | 17.20 | 46.711 | 126.8 | 0.093 | 0.9978 | N |
| 52 | 15.40 | 39.05 | 99.02 | 0.12 | 0.8659 | N |
| 56 | 13.39 | 31.62 | 74.63 | 0.12 | 0.7376 | N |
| **Waveform Measurements** | | | | | | |
| 32 | 4.380 | 29.2 | 194.0 | 1.5 | 3.593 | Y |
| 40 | 2.402 | 17.1 | 122.3 | 1.1 | 3.862 | Y |
| 44 | 1.830 | 13.88 | 105.4 | 0.97 | 4.107 | Y |
| 48 | 1.630 | 11.64 | 83.17 | 0.76 | 3.866 | Y |
| 52 | 1.491 | 10.05 | 67.75 | 0.83 | 3.641 | Y |
| 56 | 1.602 | 8.93 | 49.80 | 0.68 | 2.953 | Y |
| **Ac Measurements** | | | | | | |
| 60 | 0.9843 | 4.5 | 20.29 | 1.3 | 2.289 | Y |
| 63 | 0.6291 | 2.22 | 7.811 | 0.18 | 1.586 | Y |
| 66 | 0.4068 | 1.115 | 3.054 | 3.8E–2 | 1.016 | Y |

| | | | | | | |
|---|---|---|---|---|---|---|
| 69 | 0.2306 | 0.620 | 1.664 | 1.5E–2 | 0.9765 | Y |
| 72 | 0.1008 | 0.3292 | 1.075 | 7.7E–3 | 1.401 | Y |
| 75 | 4.847E–2 | 0.1500 | 0.4645 | 2.5E–3 | 1.277 | Y |
| 78 | 2.289E–2 | 6.87E–2 | 0.2060 | 1.2E–3 | 1.207 | Y |
| 81 | 1.178E–2 | 3.139E–2 | 8.365E–2 | 4.5E–4 | 0.9608 | Y |
| 84 | 4.912E–3 | 1.453E–2 | 4.299E–2 | 2.6E–4 | 1.177 | Y |
| 87 | 2.479E–3 | 6.91E–3 | 1.928E–2 | 1.6E–4 | 1.052 | Y |
| 90 | 1.172E–3 | 3.531E–3 | 1.064E–2 | 6.8E–5 | 1.217 | Y |
| 93 | 5.762E–4 | 1.773E–3 | 5.454E–3 | 3.5E–5 | 1.263 | Y |
| 96 | 2.930E–4 | 9.41E–4 | 3.025E–3 | 1.8E–5 | 1.362 | Y |
| 99 | 1.637E–4 | 5.03E–4 | 1.543E–3 | 1.2E–5 | 1.258 | Y |
| 102 | 8.092E–5 | 2.75E–4 | 9.379E–4 | 1.1E–5 | 1.501 | Y |
| 105 | 4.069E–5 | 1.47E–4 | 5.303E–4 | 1.3E–5 | 1.648 | Y |

**Table S36.** Relaxation times and distributions for **4-Dy**

| T (K) | $\tau_-$ (s) | $e^{\langle \ln \tau \rangle}$ (s) | $\tau_+$ (s) | $e^{\langle \ln \tau \rangle^{\mathrm{err}}}$ (s) | $\sigma^2_{\ln \tau}$ | Fit? |
|---|---|---|---|---|---|---|
| | | | **Magnetization Decays** | | | |
| 2 | 17.07 | 141.37 | 1171 | 0.94 | 4.468 | Y |
| 3 | 18.38 | 142.10 | 1098 | 0.76 | 4.182 | Y |
| 4 | 19.01 | 139.73 | 1027 | 0.68 | 3.980 | Y |
| 5 | 21.09 | 143.09 | 970.7 | 0.74 | 3.665 | Y |
| 6 | 22.30 | 143.21 | 919.6 | 0.70 | 3.458 | Y |
| 8 | 22.25 | 135.12 | 820.5 | 0.68 | 3.254 | Y |
| 10 | 25.55 | 131.65 | 678.4 | 0.55 | 2.688 | Y |
| 12 | 28.34 | 120.64 | 513.5 | 0.44 | 2.098 | Y |
| 14 | 31.19 | 107.07 | 367.5 | 0.35 | 1.521 | Y |
| 16 | 29.39 | 87.55 | 260.8 | 0.28 | 1.191 | Y |
| 18 | 27.92 | 72.26 | 187.1 | 0.28 | 0.9046 | Y |
| 20 | 24.93 | 58.46 | 137.1 | 0.26 | 0.7268 | Y |
| 22 | 23.35 | 49.02 | 102.9 | 0.32 | 0.5499 | Y |
| 24 | 19.57 | 39.73 | 80.66 | 0.28 | 0.5014 | Y |
| 26 | 17.34 | 33.28 | 63.87 | 0.30 | 0.4252 | Y |
| 28 | 14.39 | 27.52 | 52.62 | 0.26 | 0.4202 | Y |
| 30 | 12.60 | 23.37 | 43.36 | 0.28 | 0.3820 | Y |
| 32 | 10.26 | 19.57 | 37.33 | 0.25 | 0.4172 | Y |
| 34 | 9.082 | 17.02 | 31.90 | 0.27 | 0.3946 | Y |
| 36 | 7.603 | 14.56 | 27.90 | 0.34 | 0.4225 | Y |
| 38 | 6.479 | 12.58 | 24.42 | 0.28 | 0.4401 | Y |
| | | | **Waveform Measurements** | | | |
| 20 | 21.54 | 54.8 | 139.6 | 2.2 | 0.8730 | N |
| 24 | 19.11 | 37.85 | 74.97 | 0.65 | 0.4670 | N |
| 28 | 15.29 | 26.15 | 44.70 | 0.53 | 0.2876 | N |
| 32 | 13.63 | 18.86 | 26.08 | 0.40 | 0.1052 | N |
| 36 | 14.2078 | 14.2079 | 14.2080 | 0.39 | 2.128E–11 | N |
| 40 | 11.1205 (6.127) [a] | 11.1210 | 11.1216 (20.17) [a] | 0.33 | 2.472E–9 (0.3554) [a] | Y |

| | | | | | | |
|---|---|---|---|---|---|---|
| 44 | 8.95888 (4.936) [a] | 8.95891 | 8.95895 (16.26) [a] | 0.26 | 1.706E–11 (0.3554) [a] | Y |
| 47 | 7.5392 (4.154) [a] | 7.5395 | 7.5398 (13.69) [a] | 0.23 | 1.450E–9 (0.3554) [a] | Y |
| 50 | 6.353226 (3.500) [a] | 6.353228 | 6.353229 (11.53) [a] | 0.29 | 4.675E–14 (0.3554) [a] | Y |
| 54 | 5.0578 (2.787) [a] | 5.0585 | 5.0591 (9.182) [a] | 0.21 | 1.439E–8 (0.3554) [a] | Y |
| **Ac Measurements** | | | | | | |
| 60 | 1.230 | 2.73 | 6.065 | 0.26 | 0.6367 | Y |
| 63 | 0.8914 | 1.691 | 3.208 | 7.4E–2 | 0.4101 | Y |
| 66 | 0.5448 | 1.025 | 1.928 | 2.1E–2 | 0.3992 | Y |
| 69 | 0.3009 | 0.5408 | 0.9719 | 8.4E–3 | 0.3437 | Y |
| 72 | 0.1708 | 0.2533 | 0.3756 | 2.2E–3 | 0.1553 | Y |
| 75 | 7.930E–2 | 0.11509 | 0.1670 | 6.7E–4 | 0.1388 | Y |
| 78 | 3.425E–2 | 5.087E–2 | 7.554E–2 | 3.3E–4 | 0.1564 | Y |
| 81 | 1.548E–2 | 2.311E–2 | 3.450E–2 | 2.6E–4 | 0.1606 | Y |
| 84 | 7.916E–3 | 1.0563E–2 | 1.409E–2 | 7.0E–5 | 0.08319 | Y |
| 87 | 4.059E–3 | 5.052E–3 | 6.288E–3 | 3.9E–5 | 0.04791 | Y |
| 90 | 1.768E–3 | 2.545E–3 | 3.665E–3 | 2.3E–5 | 0.1330 | Y |
| 93 | 9.969E–4 | 1.296E–3 | 1.685E–3 | 1.3E–5 | 0.06880 | Y |
| 96 | 5.023E–4 | 6.759E–4 | 9.097E–4 | 6.6E–6 | 0.08820 | Y |
| 99 | 2.846E–4 | 3.741E–4 | 4.917E–4 | 5.6E–6 | 0.07476 | Y |
| 102 | 1.236E–4 | 1.975E–4 | 3.156E–4 | 8.1E–6 | 0.2196 | Y |
| 105 | 5.892E–5 | 1.04E–4 | 1.845E–4 | 1.0E–5 | 0.3258 | Y |
| 108 | 2.691E–5 | 4.71E–5 | 8.252E–5 | 6.1E–6 | 0.3139 | Y |

[a] α = 0.05 used for fitting relaxation profile so as not to over-bias these points in fitting the relaxation profile. Corresponding values with α = 0.05 are given in brackets.

**Table S37.** Relaxation times and distributions for “[{Dy(Cp$^{ttt}$)(Cp*)}{Al[OC($CF_3$)$_3$]$_4$}]”

| T (K) | $\tau_-$ (s) | $e^{\langle \ln \tau \rangle}$ (s) | $\tau_+$ (s) | $e^{\langle \ln \tau \rangle^{\mathrm{err}}}$ (s) | $\sigma^2_{\ln \tau}$ |
|---|---|---|---|---|---|
| **Magnetization Decays – single stretched exponential model** | | | | | |
| 2 | 9.501 | 164.7 | 2856 | 1.5 | 8.139 |
| 3 | 11.20 | 168.9 | 2547 | 1.5 | 7.363 |
| 4 | 12.12 | 173.3 | 2478 | 1.4 | 7.077 |
| 6 | 14.17 | 180.5 | 2299 | 1.5 | 6.476 |
| 8 | 16.60 | 185.2 | 2067 | 1.5 | 5.819 |
| 10 | 17.76 | 175.2 | 1728 | 1.4 | 5.240 |
| 12 | 18.46 | 161.5 | 1412 | 1.7 | 4.704 |
| 14 | 16.17 | 134.0 | 1111 | 1.9 | 4.473 |
| 16 | 13.42 | 109.5 | 893.2 | 2.0 | 4.406 |
| 18 | 10.81 | 89.2 | 735.9 | 2.1 | 4.454 |
| 20 | 8.658 | 73.5 | 623.9 | 2.0 | 4.574 |
| 22 | 6.925 | 61.6 | 547.1 | 1.7 | 4.773 |
| 24 | 5.968 | 53.8 | 485.7 | 1.5 | 4.838 |

| | | | | | |
|---|---|---|---|---|---|
| 26 | 5.376 | 48.5 | 437.1 | 1.4 | 4.836 |
| 28 | 4.751 | 44.0 | 407.8 | 1.2 | 4.956 |
| 30 | 4.446 | 42.5 | 406.0 | 1.0 | 5.095 |
| 32 | 4.448 | 42.34 | 403.0 | 0.85 | 5.077 |
| 34 | 4.530 | 42.25 | 394.0 | 0.69 | 4.985 |
| 36 | 4.935 | 43.34 | 380.6 | 0.59 | 4.721 |
| 38 | 6.326 | 47.77 | 360.7 | 0.57 | 4.087 |
| 40 | 7.217 | 48.98 | 332.4 | 0.60 | 3.667 |
| 42 | 8.964 | 51.43 | 295.0 | 0.65 | 3.052 |
| 44 | 10.81 | 52.95 | 259.3 | 0.68 | 2.524 |
| 46 | 12.26 | 52.57 | 225.4 | 0.67 | 2.119 |
| 48 | 14.16 | 52.68 | 196.0 | 0.63 | 1.726 |
| 50 | 14.22 | 48.72 | 166.9 | 0.54 | 1.516 |
| 52 | 15.35 | 46.70 | 142.1 | 0.55 | 1.238 |
| **Magnetization Decays – two stretched exponential model, fast component** | | | | | |
| 2 | 14.70 | 115.63 | 909.4 | 0.70 | 4.253 |
| 3 | 15.88 | 118.96 | 890.9 | 0.84 | 4.054 |
| 4 | 16.67 | 121.20 | 881.3 | 0.76 | 3.936 |
| 6 | 18.79 | 126.86 | 856.2 | 0.83 | 3.646 |
| 8 | 21.79 | 130.36 | 779.7 | 0.63 | 3.199 |
| 10 | 23.40 | 124.00 | 657.0 | 0.53 | 2.780 |
| 12 | 27.38 | 117.55 | 504.8 | 0.35 | 2.124 |
| 14 | 28.59 | 100.52 | 353.4 | 0.20 | 1.581 |
| 16 | 29.10 | 84.28 | 244.1 | 0.11 | 1.131 |
| 18 | 28.22 | 68.65 | 167.0 | 0.14 | 0.7903 |
| 20 | 26.01 | 55.778 | 119.6 | 9.7E–2 | 0.5822 |
| 22 | 23.29 | 45.645 | 89.45 | 5.7E–2 | 0.4526 |
| 24 | 20.63 | 37.448 | 67.97 | 4.1E–2 | 0.3554 |
| 26 | 17.97 | 31.093 | 53.79 | 3.7E–2 | 0.3004 |
| 28 | 14.98 | 25.886 | 44.73 | 2.4E–2 | 0.2991 |
| 30 | 13.78 | 22.064 | 35.32 | 3.3E–2 | 0.2214 |
| 32 | 11.59 | 18.728 | 30.26 | 2.8E–2 | 0.2301 |
| 34 | 9.867 | 15.981 | 25.88 | 1.9E–2 | 0.2325 |
| 36 | 8.304 | 13.705 | 22.62 | 2.5E–2 | 0.2511 |
| 38 | 7.421 | 11.923 | 19.16 | 5.0E–2 | 0.2248 |
| 40 | 6.011 | 10.226 | 17.40 | 4.2E–2 | 0.2824 |
| 42 | 4.912 | 8.872 | 16.02 | 7.4E–2 | 0.3496 |
| 44 | 5.024 | 8.02 | 12.82 | 0.20 | 0.2192 |
| 46 | 4.533 | 7.220 | 11.50 | 8.9E–2 | 0.2167 |
| 48 | 4.037 | 6.43 | 10.24 | 0.11 | 0.2167 |
| 50 | 4.135 | 6.59 | 10.49 | 0.16 | 0.2167 |
| 52 | 3.258 | 5.19 | 8.266 | 0.29 | 0.2167 |
| **Magnetization Decays – two stretched exponential model, slow component** | | | | | |
| 2 | 166.4 | 1198 | 8631 | 21 | 3.898 |
| 3 | 149.9 | 1047 | 7309 | 22 | 3.776 |
| 4 | 157.6 | 1045 | 6922 | 19 | 3.577 |
| 6 | 157.7 | 991 | 6223 | 19 | 3.377 |
| 8 | 178.1 | 970 | 5285 | 14 | 2.873 |

| | | | | | |
|---|---|---|---|---|---|
| 10 | 183.5 | 879 | 4207 | 11 | 2.453 |
| 12 | 198.1 | 814.4 | 3348 | 6.3 | 1.998 |
| 14 | 201.4 | 721.6 | 2585 | 3.2 | 1.629 |
| 16 | 210.7 | 650.7 | 2009 | 1.6 | 1.271 |
| 18 | 177.0 | 526 | 1562 | 11 | 1.186 |
| 20 | 161.7 | 448.6 | 1245 | 6.8 | 1.042 |
| 22 | 149.4 | 389.4 | 1015 | 3.5 | 0.9182 |
| 24 | 133.4 | 332.6 | 829.3 | 2.2 | 0.8345 |
| 26 | 126.5 | 294.9 | 687.5 | 1.7 | 0.7165 |
| 28 | 120.8 | 262.5 | 570.1 | 1.0 | 0.6018 |
| 30 | 105.7 | 226.0 | 483.5 | 1.1 | 0.5782 |
| 32 | 95.41 | 198.35 | 412.4 | 0.80 | 0.5357 |
| 34 | 86.30 | 174.89 | 354.4 | 0.46 | 0.4989 |
| 36 | 80.50 | 156.39 | 303.8 | 0.49 | 0.4411 |
| 38 | 72.47 | 138.43 | 264.4 | 0.66 | 0.4189 |
| 40 | 66.99 | 124.25 | 230.4 | 0.40 | 0.3815 |
| 42 | 62.40 | 111.99 | 201.0 | 0.47 | 0.3421 |
| 44 | 56.59 | 99.98 | 176.7 | 0.65 | 0.3240 |
| 46 | 52.71 | 90.19 | 154.3 | 0.42 | 0.2885 |
| 48 | 47.92 | 80.72 | 136.0 | 0.37 | 0.2718 |
| 50 | 44.78 | 72.58 | 117.6 | 0.53 | 0.2332 |
| 52 | 42.23 | 64.87 | 99.66 | 0.63 | 0.1843 |
| **Ac Measurements** | | | | | |
| 63 | 0.8789 | 1.675 | 3.192 | 9.5E–2 | 0.4159 |
| 66 | 0.5698 | 1.046 | 1.919 | 2.8E–2 | 0.3686 |
| 69 | 0.2735 | 0.5585 | 1.141 | 8.5E–3 | 0.5099 |
| 72 | 0.1301 | 0.2971 | 0.6782 | 4.3E–3 | 0.6813 |
| 75 | 5.480E–2 | 0.1378 | 0.3464 | 2.3E–3 | 0.8499 |
| 78 | 2.653E–2 | 6.309E–2 | 0.1500 | 9.9E–4 | 0.7504 |
| 81 | 1.120E–2 | 2.808E–2 | 7.041E–2 | 4.7E–4 | 0.8449 |
| 84 | 5.336E–3 | 1.324E–2 | 3.284E–2 | 2.3E–4 | 0.8256 |
| 87 | 2.528E–3 | 6.31E–3 | 1.574E–2 | 1.2E–4 | 0.8360 |
| 90 | 1.175E–3 | 3.143E–3 | 8.405E–3 | 5.8E–5 | 0.9675 |
| 93 | 5.768E–4 | 1.575E–3 | 4.302E–3 | 2.8E–5 | 1.009 |
| 96 | 2.890E–4 | 8.23E–4 | 2.346E–3 | 1.9E–5 | 1.096 |
| 99 | 1.340E–4 | 4.171E–4 | 1.298E–3 | 9.0E–6 | 1.289 |
| 102 | 6.134E–5 | 2.152E–4 | 7.554E–4 | 6.2E–6 | 1.576 |
| 105 | 2.857E–5 | 1.08E–4 | 4.098E–4 | 1.5E–5 | 1.773 |
| 108 | 1.918E–5 | 6.2E–5 | 2.032E–4 | 1.0E–5 | 1.393 |

**Table S38.** Relaxation times and distributions for **5%Dy@4-Y**

| T (K) | $\tau_{-}$ (s) | $e^{\langle \ln \tau \rangle}$ (s) | $\tau_{+}$ (s) | $e^{\langle \ln \tau \rangle^{\mathrm{err}}}$ (s) | $\sigma^2_{\ln \tau}$ | Fit? |
|---|---|---|---|---|---|---|
| **Magnetization Decays – single stretched exponential model** | | | | | | |
| 2 | 38.5 | 338.1 | 2970 | 0.16 | 2.173 | Y |
| 3 | 38.1 | 332.6 | 2905 | 0.13 | 2.167 | Y |
| 4 | 27.7 | 309.5 | 3460 | 0.14 | 2.414 | Y |
| 6 | 33.6 | 312.7 | 2913 | 0.08 | 2.232 | Y |
| 8 | 34.1 | 270.2 | 2143 | 0.06 | 2.071 | Y |
| 10 | 40.5 | 228.2 | 1286 | 0.16 | 1.730 | Y |
| 12 | 30.1 | 126.7 | 533.3 | 9.1E–2 | 1.437 | Y |
| 14 | 27.7 | 106.8 | 412.3 | 6.3E–2 | 1.351 | Y |
| 16 | 23.4 | 83.86 | 299.9 | 6.1E–2 | 1.274 | Y |
| 18 | 20.1 | 66.78 | 221.5 | 6.9E–2 | 1.199 | Y |
| **Magnetization Decays – two stretched exponential model, fast component** | | | | | | |
| 20 | 20.86 | 55.59 | 148.1 | 6.1E–2 | 0.9602 | Y |
| 24 | 15.40 | 37.07 | 89.25 | 4.1E–2 | 0.7719 | Y |
| 28 | 11.98 | 25.21 | 53.06 | 3.9E–2 | 0.5540 | Y |
| 32 | 9.020 | 17.74 | 34.89 | 3.7E–2 | 0.4574 | Y |
| 36 | 7.833 | 13.61 | 23.66 | 2.3E–2 | 0.3054 | Y |
| 40 | 6.028 | 10.04 | 16.71 | 2.6E–2 | 0.2598 | Y |
| 44 | 5.116 | 7.855 | 12.06 | 2.5E–2 | 0.1839 | Y |
| 48 | 4.582 | 6.276 | 8.594 | 3.2E–2 | 0.0989 | Y |
| **Magnetization Decays – two stretched exponential model, slow component** | | | | | | |
| 20 | 3.892 | 57.46 | 848.1 | 1.8 | 7.247 | N |
| 24 | 17.43 | 125.9 | 909.3 | 12 | 3.909 | N |
| 28 | 11.09 | 78.59 | 556.8 | 10 | 3.834 | N |
| 32 | 5.211 | 47.25 | 428.5 | 6.1 | 4.861 | N |
| 36 | 1.954 | 23.50 | 282.6 | 1.2 | 6.185 | N |
| 40 | 3.002 | 27.97 | 260.6 | 3.4 | 4.981 | N |
| 44 | 2.121 | 20.93 | 206.5 | 2.6 | 5.241 | N |
| 48 | 1.160 | 13.46 | 156.2 | 1.8 | 6.009 | N |
| **Ac Measurements** | | | | | | |
| 66 | 0.1162 | 0.6620 | 3.773 | 9.2E–2 | 3.029 | Y |
| 69 | 8.446E–2 | 0.4519 | 2.418 | 5.4E–2 | 2.813 | Y |
| 72 | 4.370E–2 | 0.1752 | 0.7022 | 8.0E–3 | 1.928 | Y |
| 75 | 2.292E–2 | 7.581E–2 | 0.2507 | 2.5E–3 | 1.431 | Y |
| 78 | 1.197E–2 | 3.505E–2 | 0.1026 | 1.0E–3 | 1.154 | Y |
| 81 | 5.644E–3 | 1.476E–2 | 3.858E–2 | 4.4E–4 | 0.9237 | Y |
| 84 | 2.857E–3 | 7.430E–3 | 1.932E–2 | 3.4E–4 | 0.9132 | Y |
| 87 | 1.474E–3 | 3.647E–3 | 9.024E–3 | 1.2E–4 | 0.8205 | Y |
| 90 | 6.321E–4 | 1.850E–3 | 5.412E–3 | 1.3E–4 | 1.153 | Y |
| 93 | 3.610E–4 | 9.646E–4 | 2.577E–3 | 5.0E–5 | 0.9660 | Y |
| 96 | 2.539E–4 | 4.955E–4 | 9.669E–4 | 4.1E–5 | 0.4470 | Y |
| 99 | 1.295E–4 | 2.802E–4 | 6.061E–4 | 2.6E–5 | 0.5952 | Y |
| 102 | 1.119E–4 | 1.682E–4 | 2.527E–4 | 2.6E–5 | 0.1659 | Y |

**Table S39.** Relaxation times and distributions for **4-Dy** in benzene

| T (K) | $\tau_{-}$ (s) | $e^{\langle \ln \tau \rangle}$ (s) | $\tau_{+}$ (s) | $e^{\langle \ln \tau \rangle^{\text{err}}}$ (s) | $\sigma^2_{\ln \tau}$ |
|---|---|---|---|---|---|
| **Magnetization Decays – two stretched exponential model, fast component** | | | | | |
| 2 | 0.9645 | 19.34 | 387.9 | 0.11 | 8.990 |
| 3 | 0.8141 | 16.88 | 350.1 | 0.13 | 9.193 |
| 4 | 1.402 | 23.30 | 387.0 | 0.15 | 7.897 |
| 6 | 1.327 | 20.17 | 306.4 | 2.9E–2 | 7.404 |
| 8 | 1.931 | 26.61 | 366.9 | 3.4E–2 | 6.883 |
| 10 | 2.679 | 27.74 | 287.4 | 3.5E–2 | 5.465 |
| 12 | 2.668 | 22.44 | 188.7 | 3.1E–2 | 4.534 |
| 14 | 2.948 | 19.42 | 128.0 | 2.5E–2 | 3.555 |
| 16 | 4.577 | 20.59 | 92.64 | 2.5E–2 | 2.261 |
| 18 | 6.478 | 20.91 | 67.51 | 4.6E–2 | 1.373 |
| 20 | 12.04 | 24.61 | 50.33 | 0.13 | 0.5117 |
| 24 | 21.87 | 26.61 | 32.38 | 0.50 | 0.0385 |
| **Magnetization Decays – two stretched exponential model, slow component** | | | | | |
| 2 | 6.003 | 1.380E+3 | 3.170E+5 | 1.5E+2 | 29.56 |
| 3 | 9.027 | 1.374E+3 | 2.092E+5 | 2.2E+2 | 25.26 |
| 4 | 13.87 | 1.439E+3 | 1.493E+5 | 1.6E+2 | 21.55 |
| 6 | 41.50 | 1.468E+3 | 5.196E+4 | 38 | 12.72 |
| 8 | 851.1 | 4.461E+3 | 2.338E+4 | 24 | 2.744 |
| 10 | 400.7 | 2.080E+3 | 1.080E+4 | 8.5 | 2.713 |
| 12 | 136.0 | 8.730E+2 | 5.604E+3 | 3.5 | 3.457 |
| 14 | 55.66 | 4.215E+2 | 3.192E+3 | 1.5 | 4.099 |
| 16 | 28.01 | 2.367E+2 | 2.000E+3 | 0.77 | 4.555 |
| 18 | 17.08 | 1.531E+2 | 1.371E+3 | 0.54 | 4.808 |
| 20 | 12.55 | 1.129E+2 | 1.015E+3 | 0.47 | 4.825 |
| 24 | 9.429 | 77.92 | 6.439E+2 | 0.65 | 4.460 |
| **Magnetization Decays – single stretched exponential model** | | | | | |
| 28 | 5.487 | 43.81 | 349.8 | 9.2E–2 | 4.316 |
| 32 | 5.343 | 37.38 | 261.5 | 9.7E–2 | 3.785 |
| 36 | 5.019 | 31.81 | 201.6 | 0.10 | 3.409 |
| 40 | 4.905 | 28.26 | 162.8 | 9.8E–2 | 3.067 |
| 44 | 4.276 | 23.37 | 127.7 | 0.10 | 2.884 |
| 48 | 3.397 | 18.45 | 100.3 | 8.9E–2 | 2.865 |
| 52 | 3.171 | 15.74 | 78.10 | 9.3E–2 | 2.566 |
| **Ac Measurements – double generalized Debye model, fast component** | | | | | |
| 63.0 | 3.674E–3 | 1.530E–2 | 6.369E–2 | 1.7E–3 | 2.035 |
| 64.5 | 2.787E–3 | 1.059E–2 | 4.027E–2 | 8.3E–4 | 1.783 |
| 66.0 | 1.696E–3 | 6.974E–3 | 2.868E–2 | 5.9E–4 | 2.000 |
| 67.5 | 1.036E–3 | 4.767E–3 | 2.193E–2 | 3.3E–4 | 2.329 |
| 69.0 | 7.687E–4 | 3.286E–3 | 1.404E–2 | 1.9E–4 | 2.110 |
| 70.5 | 6.180E–4 | 2.251E–3 | 8.198E–3 | 1.4E–4 | 1.671 |
| 72.0 | 4.881E–4 | 1.515E–3 | 4.705E–3 | 8.8E–5 | 1.284 |
| 73.5 | 2.933E–4 | 1.071E–3 | 3.913E–3 | 6.5E–5 | 1.678 |
| 75.0 | 2.319E–4 | 7.331E–4 | 2.318E–3 | 3.9E–5 | 1.325 |
| 76.5 | 1.448E–4 | 4.948E–4 | 1.690E–3 | 3.5E–5 | 1.510 |

| | | | | | |
|---|---|---|---|---|---|
| **Ac Measurements – double generalized Debye model, slow component** | | | | | |
| 63.0 | 0.2002 | 1.173 | 6.867 | 4.6E–2 | 3.124 |
| 64.5 | 0.1652 | 0.9206 | 5.129 | 2.3E–2 | 2.950 |
| 66.0 | 0.1370 | 0.7325 | 3.917 | 1.7E–2 | 2.811 |
| 67.5 | 0.1106 | 0.5472 | 2.708 | 8.2E–3 | 2.557 |
| 69.0 | 8.717E–2 | 0.3947 | 1.787 | 4.8E–3 | 2.281 |
| 70.5 | 6.383E–2 | 0.2758 | 1.191 | 3.6E–3 | 2.141 |
| 72.0 | 4.719E–2 | 0.1872 | 0.7427 | 2.2E–3 | 1.899 |
| 73.5 | 3.398E–2 | 0.1286 | 0.4867 | 1.5E–3 | 1.771 |
| 75.0 | 2.406E–2 | 8.859E–2 | 0.3262 | 8.7E–4 | 1.699 |
| 76.5 | 1.708E–2 | 5.932E–2 | 0.2061 | 6.4E–4 | 1.550 |
| **Ac Measurements – single generalized Debye model** | | | | | |
| 78.0 | 1.036E–2 | 3.851E–2 | 0.1431 | 4.1E–4 | 1.723 |
| 79.5 | 7.386E–3 | 2.597E–2 | 9.133E–2 | 2.8E–4 | 1.581 |
| 81.0 | 4.992E–3 | 1.785E–2 | 6.384E–2 | 2.3E–4 | 1.624 |
| 82.5 | 3.538E–3 | 1.220E–2 | 4.207E–2 | 1.4E–4 | 1.532 |
| 84.0 | 2.478E–3 | 8.478E–3 | 2.900E–2 | 8.7E–5 | 1.513 |
| 87.0 | 1.204E–3 | 4.115E–3 | 1.406E–2 | 3.9E–5 | 1.510 |
| 88.5 | 8.496E–4 | 2.909E–3 | 9.961E–3 | 3.2E–5 | 1.515 |
| 90.0 | 6.076E–4 | 2.079E–3 | 7.111E–3 | 2.0E–5 | 1.513 |
| 91.5 | 4.617E–4 | 1.509E–3 | 4.933E–3 | 1.9E–5 | 1.403 |
| 93.0 | 3.335E–4 | 1.080E–3 | 3.494E–3 | 1.2E–5 | 1.380 |
| 94.5 | 2.492E–4 | 7.861E–4 | 2.480E–3 | 1.0E–5 | 1.320 |
| 96.0 | 1.848E–4 | 5.782E–4 | 1.809E–3 | 8.6E–6 | 1.301 |
| 97.5 | 1.466E–4 | 4.366E–4 | 1.300E–3 | 6.9E–6 | 1.190 |
| 99.0 | 1.099E–4 | 3.248E–4 | 9.602E–4 | 8.3E–6 | 1.175 |
| 100.5 | 7.580E–5 | 2.353E–4 | 7.303E–4 | 7.7E–6 | 1.283 |
| 102.0 | 6.344E–5 | 1.819E–4 | 5.217E–4 | 8.9E–6 | 1.110 |
| 103.5 | 4.718E–5 | 1.352E–4 | 3.875E–4 | 7.5E–6 | 1.109 |
| 105.0 | 3.629E–5 | 9.890E–5 | 2.695E–4 | 8.2E–6 | 1.005 |

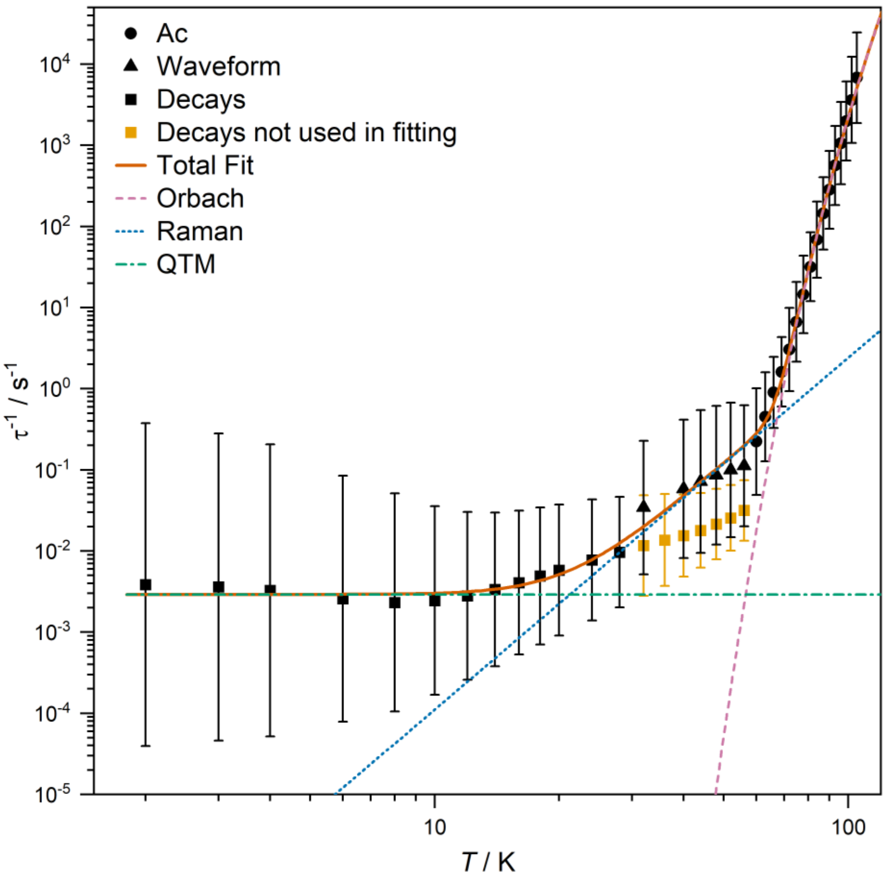


**Figure S154.** Fitting of **3-Dy·$C_6H_6$** relaxation profile to Equation S9, showing Orbach, Raman and QTM components. Data from magnetisation decays not used in the fit are shown in yellow. Error bars represent one ESD in the distribution of rates.

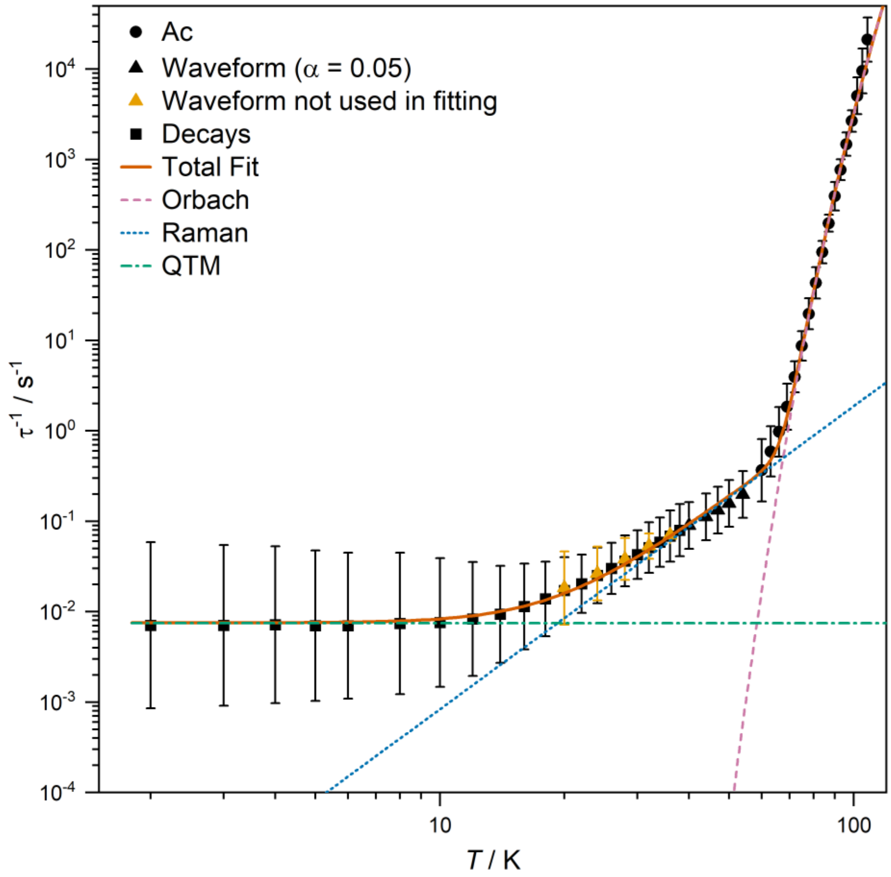


**Figure S155.** Fitting of relaxation profile for **4-Dy** to Equation S9 showing Orbach, Raman and QTM components. Error bars represent one ESD in the distribution of rates. For waveform data used for fitting the ESDs are calculated with α = 0.05.

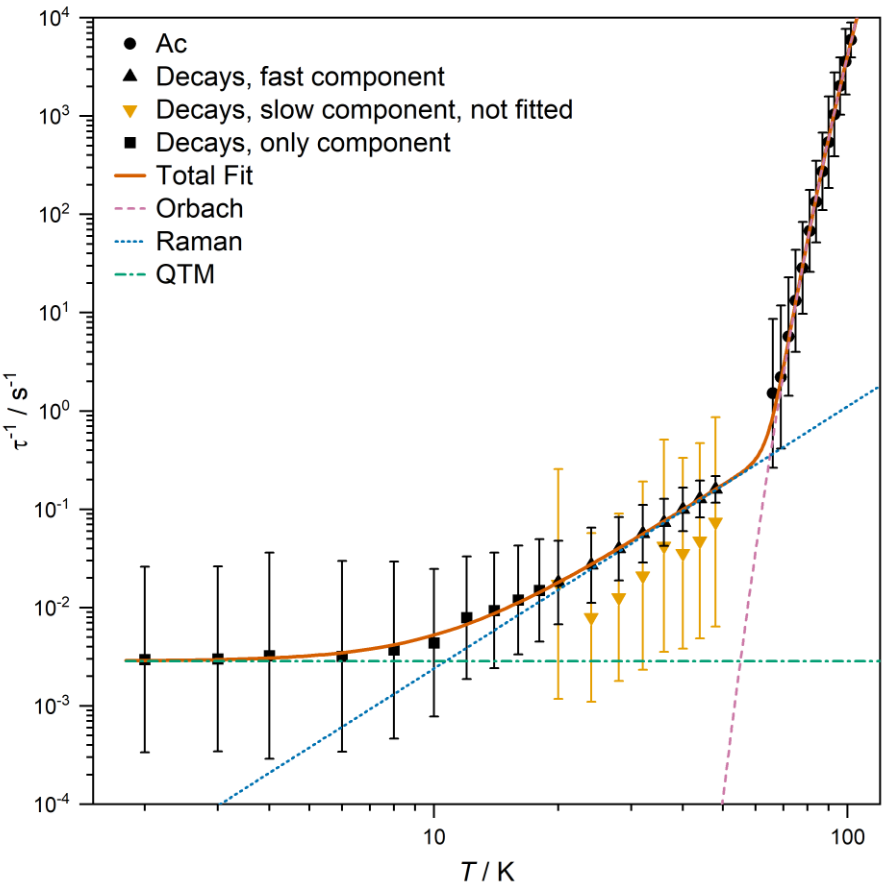


**Figure S156.** Fitting of relaxation profile for **5%Dy@4-Y**. The ac, single stretched exponential, and major components of the sum of two stretched exponentials were fit to Equation S9 with Orbach, Raman and QTM components shown. The minor decay components (4-10%) are shown but were not fit in the relaxation profile. Error bars represent one ESD in the distribution of rates.

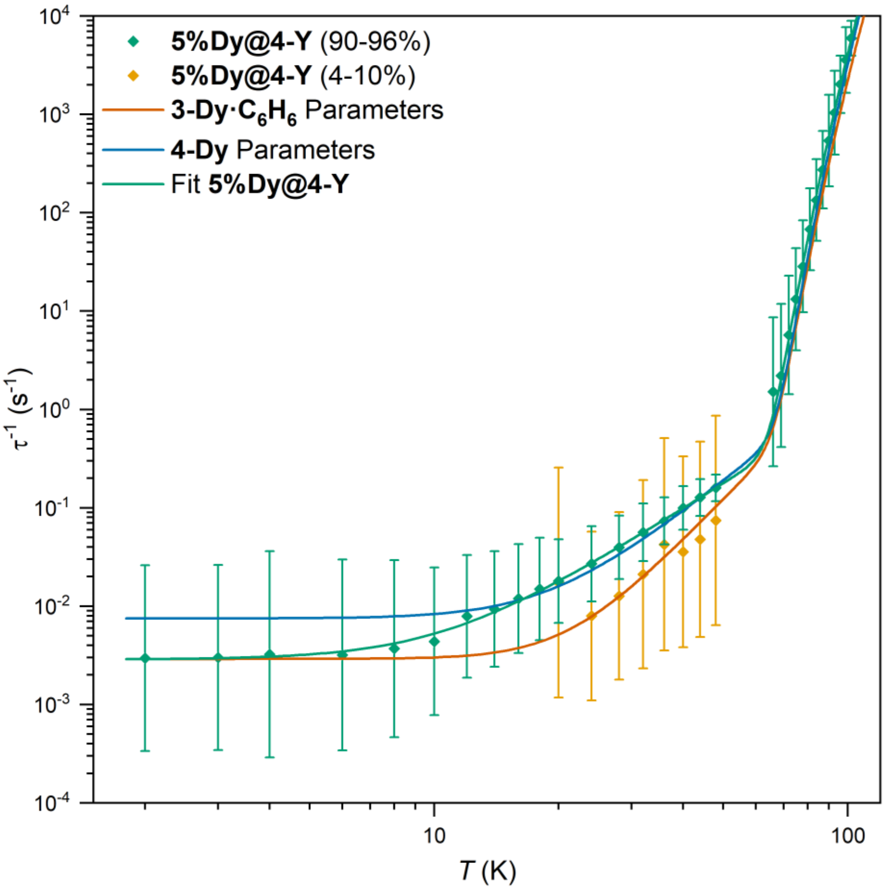


**Figure S157.** Comparison of **5%Dy@4-Y** rates and parameters from relaxation profiles of **3-Dy·$C_6H_6$** and **4-Dy**. Error bars represent one ESD in the distribution of rates.

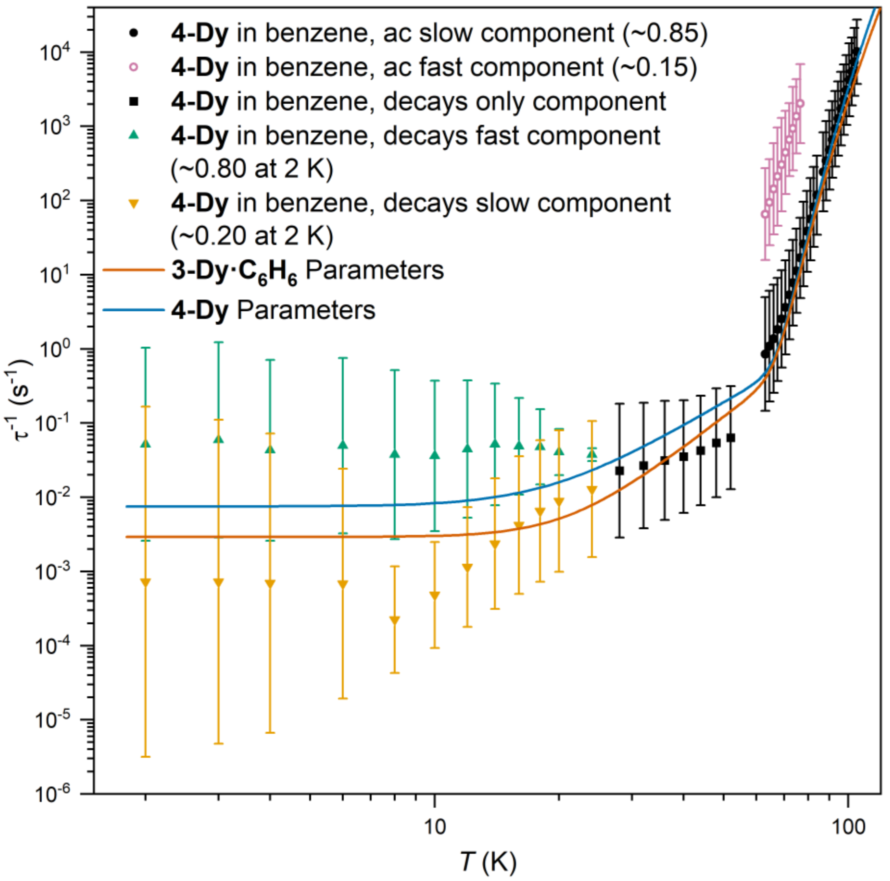


**Figure S158.** Comparison of **4-Dy** in benzene rates and parameters from relaxation profiles of **3-Dy·$C_6H_6$** and **4-Dy**. Error bars represent one ESD in the distribution of rates.

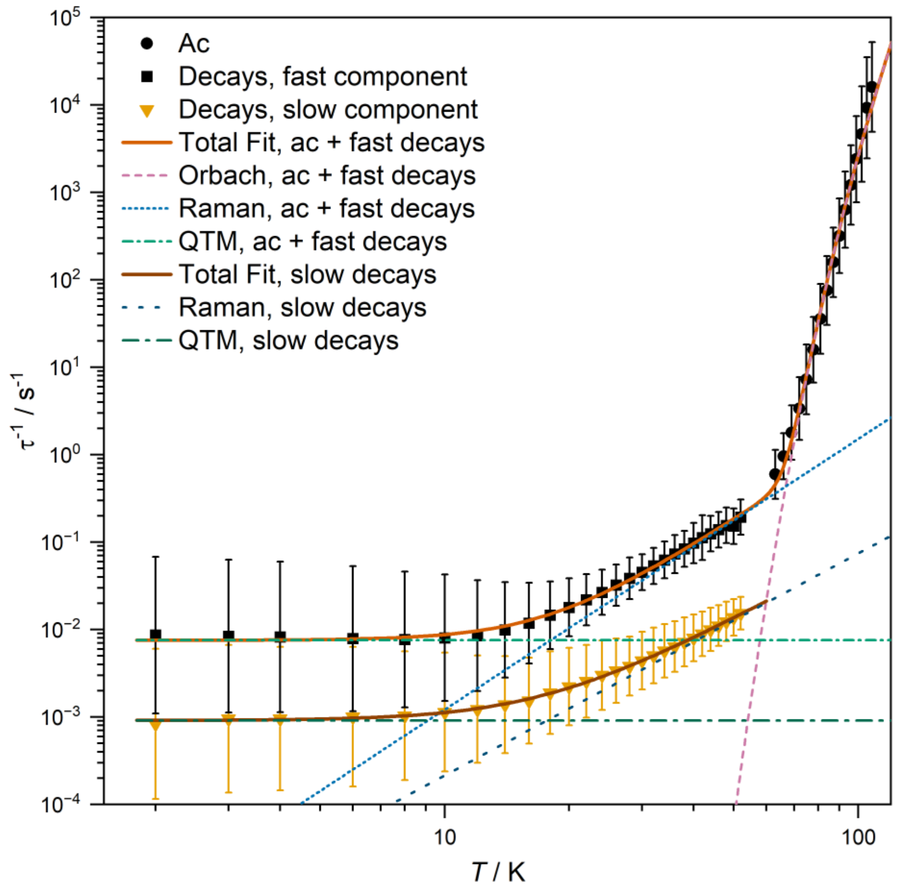


**Figure S159.** Fitting of relaxation profile for "[{Dy(Cp$^{ttt}$)(Cp*)}{Al[OC($CF_3$)$_3$]$_4$}]". The ac and fast components from magnetization decays were fit to Equation S9 with Orbach, Raman and QTM components shown. The slow components from magnetization decays were fit to Equation S10 with Raman and QTM components shown. Error bars represent one ESD in the distribution of rates.

**Table S40.** Selected SMM parameters from temperature-dependence of relaxation times for "[{Dy(Cp$^{ttt}$)(Cp*)}{Al[OC($CF_3$)$_3$]$_4$}]" with standard deviations

| | Fast component + ac | Slow component |
|---|---|---|
| $U_{eff}$ / cm$^{-1}$ | 1220(22) | – |
| $A$ ($\tau_0 = 10^A$ / s) | –11.0(2) | – |
| $R$ ($C = 10^R$ / s$^{-1}$ K$^{-n}$) | –6.0(2) | –6.21(4) |
| $n$ | 3.1(2) | 2.54(3) |
| $Q$ ($\tau_{QTM} = 10^Q$ / s) | 2.12(6) | 3.040(9) |
| $T_H$ / K | 50 | 50 |
| $T_{100}$ / K | 14 | 44 |

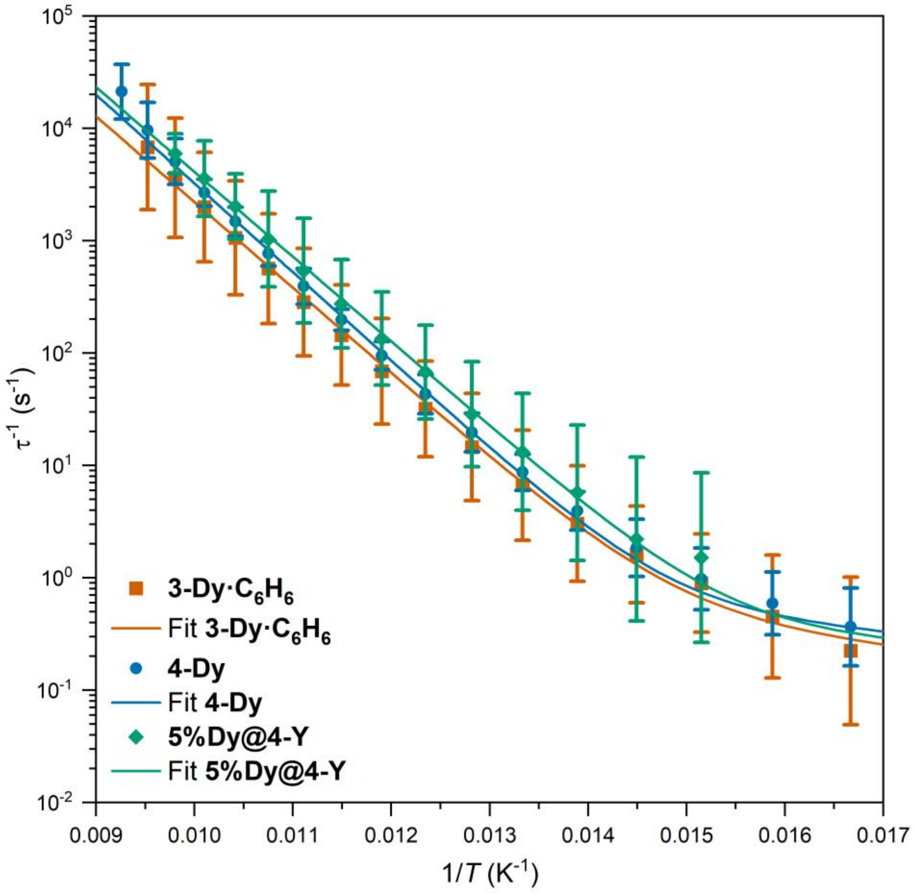


**Figure S160.** Temperature dependence of the ac relaxation rates in the Orbach region for **3-Dy·$C_6H_6$**, **4-Dy** and **5%Dy@4-Y** with corresponding fits. Error bars represent one ESD in the distribution of rates. The fit for **5%Dy@4-Y** includes the major component (> 89%) in the magnetization decays.

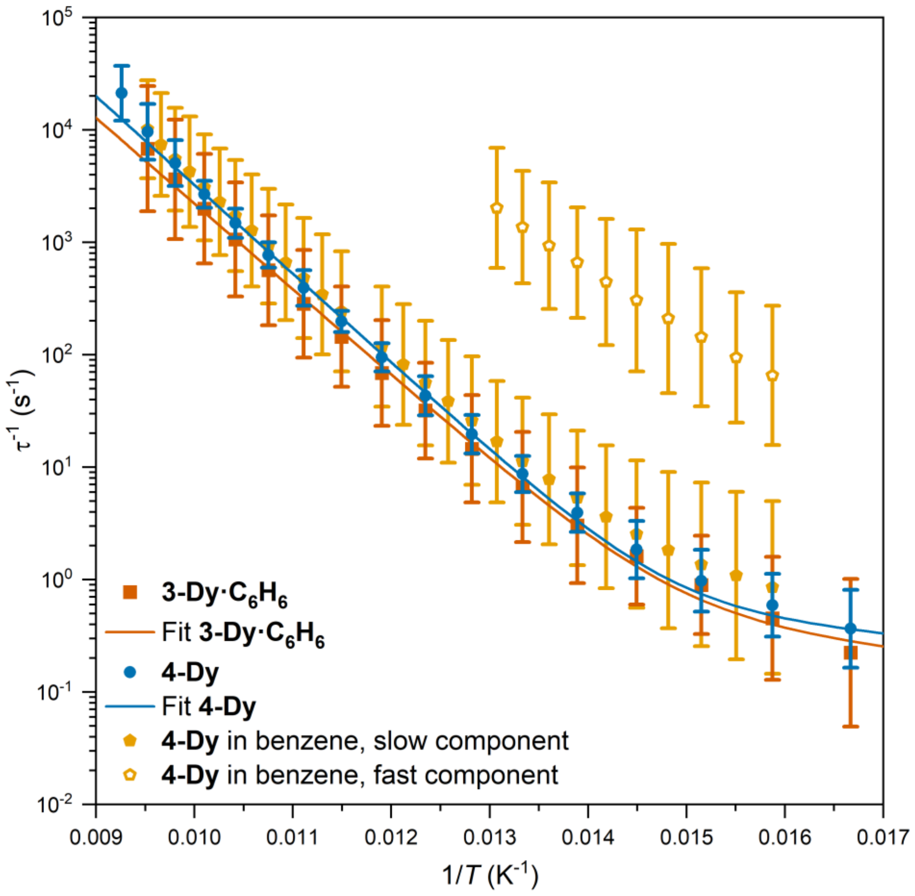


**Figure S161.** Temperature dependence of the ac relaxation rates in the Orbach region for **3-Dy·$C_6H_6$**, **4-Dy** and **4-Dy** in benzene including the slow component (approx. 85%) and fast component (approx. 15%). Error bars represent one ESD in the distribution of rates.

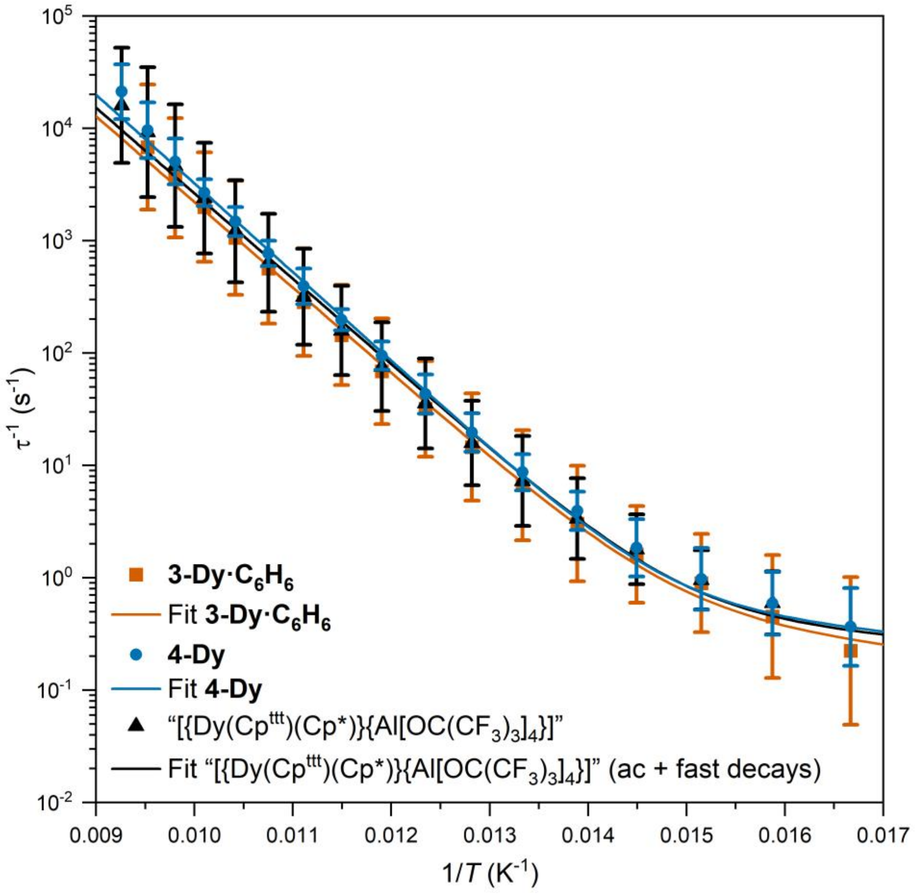


**Figure S162.** Temperature dependence of the ac relaxation rates in the Orbach region for **3-Dy·C$_6$H$_6$**, **4-Dy** and "[{Dy(Cp$^{ttt}$)(Cp*)}{Al[OC(CF$_3$)$_3$]$_4$}]" with corresponding fits. Error bars represent one ESD in the distribution of rates. The fit for "[{Dy(Cp$^{ttt}$)(Cp*)}{Al[OC(CF$_3$)$_3$]$_4$}]" includes only the fast-relaxing component from the magnetization decays.

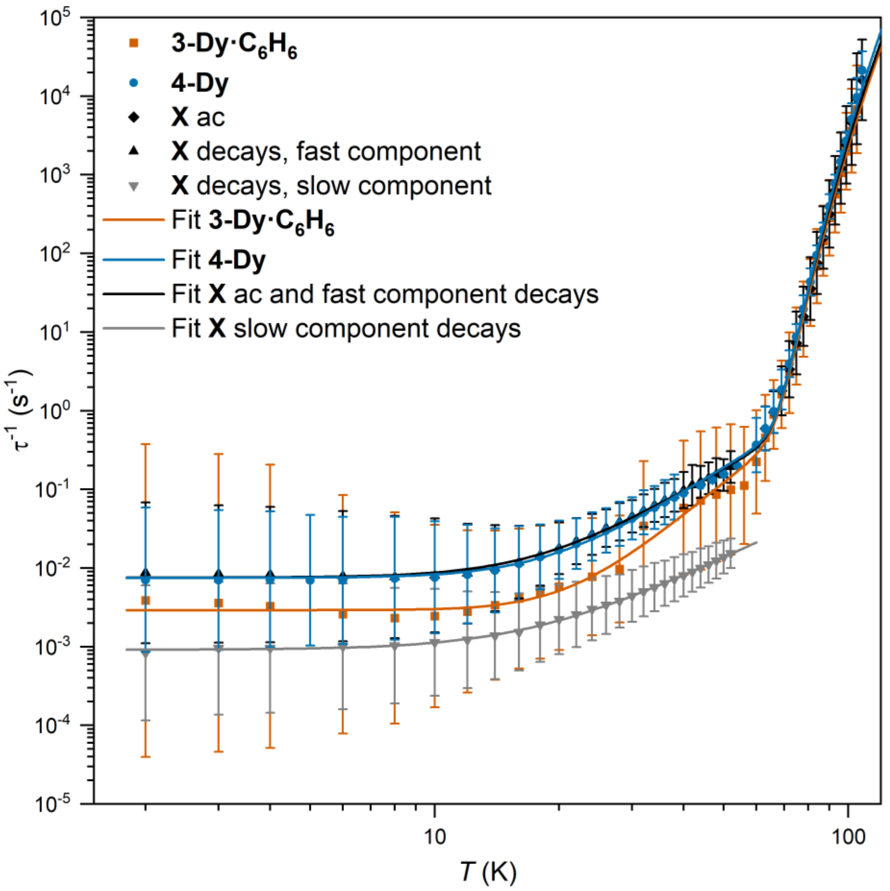


**Figure S163.** Direct comparison of selected rates and corresponding fits for **3-Dy·$C_6H_6$**, **4-Dy** and “[{Dy(Cp$^{ttt}$)(Cp*)}{Al[OC($CF_3$)$_3$]$_4$}]” (**X**). Error bars represent one ESD in the distribution of rates.

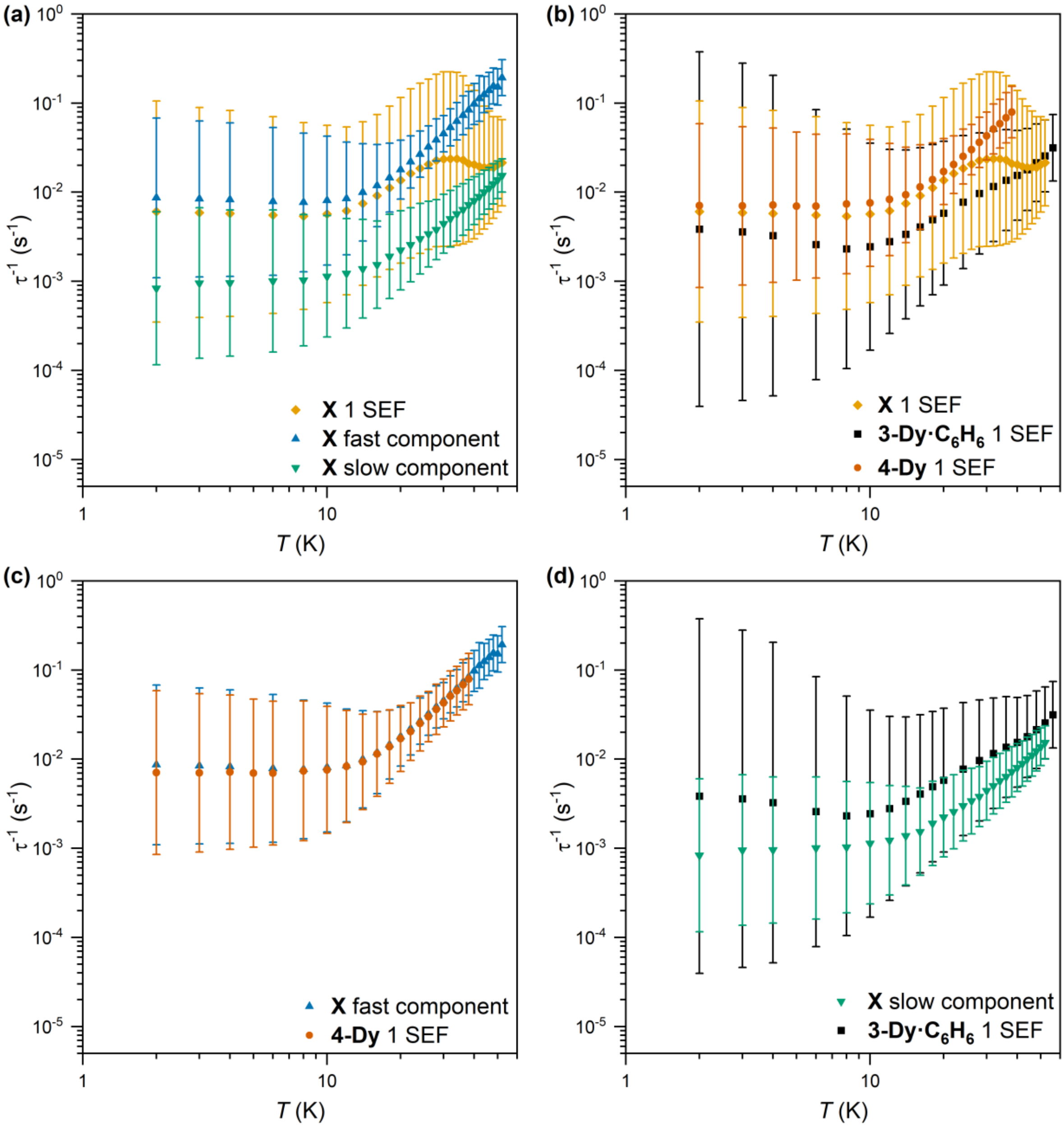


**Figure S164.** (a) Relaxation rates for "[{Dy(Cp$^{ttt}$)(Cp*)}{Al[OC($CF_3$)$_3$]$_4$}]" (**X**) extracted from magnetization decays using a single stretched exponential function (yellow), or a sum of two stretched exponential functions showing the fast component (blue) and slow component (green). (b–d) Select comparisons of the relaxation rates for **X** with relaxation rates for **3-Dy·$C_6H_6$** (black) and **4-Dy** (red-brown) extracted from magnetization decays with single stretched exponential functions.

## 10. CASSCF-SO Calculations

OpenMolcas[44] was used to perform CASSCF-SO calculations on **1-4-Dy** to determine their electronic structures. The molecular geometries from the single crystal XRD structures were used with no optimization, taking the largest disorder component only. Both cation and anion were included for **3-Dy** and **4-Dy**, the benzene co-crystallized solvent was not included for **3-Dy**. Integrals were performed in the SEWARD module using basis sets from ANO-RCC library[45–48] with VTZP quality for Dy atoms, VDZP quality for the cyclopentadienyl C atoms, the THF O atom (**1-Dy**) the borohydride B atom (**1-2-Dy**) and the closest $C(CF_3)_3$ F atom (**4-Dy**) and VDZ quality for all remaining atoms, employing the second-order DKH transformation. Cholesky decomposition of the two-electron integrals with a threshold of $10^{-8}$ was performed to save disk space and reduce computational demand. The molecular orbitals (MOs) were optimized in state-averaged CASSCF calculations in the RASSCF module, where the active space was defined by the nine 4f electrons in the seven 4f orbitals of Dy(III). Three such calculations were performed independently for each possible spin state, where 21 roots were included for $S$ = 5/2, 224 roots were included for $S$ = 3/2, and 490 roots were included for $S$ = 1/2. The wavefunctions obtained from these CASSCF calculations were then mixed by spin orbit coupling in the RASSI module, where all 21 $S$ = 5/2 states, 128 of the $S$ = 3/2 states, and 130 of the $S$ = 1/2 states were included**.** SINGLE_ANISO was used to decompose the resulting spin-orbit wave functions into the CF Hamiltonian formalism.[49] Diamond was employed for molecular graphics.[50]

**Table S41.** Electronic structure of **1-Dy** calculated with the crystal field parameters obtained from CASSCF-SO using the solid state geometry of **1-Dy**. Each row corresponds to a Kramers doublet.

| Energy ($cm^{-1}$) | Energy (K) | $g_x$ | $g_y$ | $g_z$ | Angle[a] (deg) | Wavefunction | $<J_z>$ |
|---|---|---|---|---|---|---|---|
| 0.00 | 0.00 | 0.011 | 0.019 | 19.7 | -- | 98% \|± 15/2⟩ | ±7.438 |
| 141.64 | 203.82 | 0.45 | 1.0 | 17.7 | 42.0 | 34% \|± 13/2⟩ + 23% \|± 9/2⟩ + 20% \|± 11/2⟩ + 13% \|± 7/2⟩ + 5% \|± 5/2⟩ | ±4.970 |
| 183.77 | 264.44 | 0.042 | 1.4 | 15.4 | 43.0 | 26% \|± 11/2⟩ + 22% \|± 7/2⟩ + 18% \|± 13/2⟩ + 13% \|± 9/2⟩ + 8% \|± 5/2⟩ + 6% \|± 3/2⟩ | ±4.265 |
| 235.06 | 338.25 | 0.92 | 2.0 | 13.1 | 20.0 | 36% \|± 13/2⟩ + 27% \|± 9/2⟩ + 17% \|± 5/2⟩ + 10% \|± 11/2⟩ + 5% \|± 7/2⟩ | ±4.683 |
| 294.21 | 423.37 | 2.0 | 4.7 | 10.5 | 29.4 | 30% \|± 7/2⟩ + 25% \|± 11/2⟩ + 12% \|± 3/2⟩ + 10% \|± 13/2⟩ + 8% \|± 9/2⟩ + 6% \|± 5/2⟩ | ±3.534 |
| 341.10 | 490.84 | 3.7 | 4.9 | 12.8 | 74.6 | 34% \|± 5/2⟩ + 13% \|± 11/2⟩ + 13% \|± 9/2⟩ + 12% \|± 3/2⟩ + 6% \|± 7/2⟩ + 6% \|∓ 7/2⟩ + 6% \|∓ 3/2⟩ | ±2.004 |
| 423.67 | 609.66 | 0.46 | 1.4 | 15.5 | 81.8 | 17% \|± 3/2⟩ + 17% \|∓ 3/2⟩ + 14% \|± 7/2⟩ + 14% \|∓ 1/2⟩ + 10% \|± 5/2⟩ + 9% \|± 9/2⟩ + 9% \|± 1/2⟩ + 7% \|∓ 5/2⟩ | ±0.960 |
| 461.88 | 664.64 | 0.53 | 2.0 | 17.8 | 82.0 | 47% \|± 1/2⟩ + 25% \|± 3/2⟩ + 12% \|± 5/2⟩ + 11% \|∓ 1/2⟩ | ±0.971 |

[a] The angle between the $g_z$ value of the excited Kramers doublet and the ground Kramers doublet.

**Table S42.** Electronic structure of **2-Dy** calculated with the crystal field parameters obtained from CASSCF-SO using the solid state geometry of **2-Dy**. Each row corresponds to a Kramers doublet.

| Energy ($cm^{-1}$) | Energy (K) | $g_x$ | $g_y$ | $g_z$ | Angle[a] (deg) | Wavefunction | $<J_z>$ |
|---|---|---|---|---|---|---|---|
| 0.00 | 0.00 | 0.017 | 0.030 | 19.6 | -- | 95% \|± 15/2⟩ + 5% \|± 11/2⟩ | ±7.392 |
| 245.26 | 352.93 | 0.66 | 1.3 | 15.7 | 2.2 | 81% \|± 13/2⟩ + 14% \|± 9/2⟩ | ±5.946 |
| 361.35 | 519.98 | 4.1 | 5.6 | 10.8 | 87.2 | 28% \|± 11/2⟩ + 23% \|± 7/2⟩ + 18% \|± 3/2⟩ + 14% \|∓ 1/2⟩ + 8% \|∓ 5/2⟩ + 5% \|∓ 13/2⟩ | ±2.150 |
| 442.23 | 636.37 | 0.068 | 2.6 | 8.5 | 88.7 | 41% \|± 11/2⟩ + 19% \|∓ 5/2⟩ + 13% \|∓ 9/2⟩ + 12% \|∓ 1/2⟩ + 7% \|∓ 13/2⟩ | ±0.920 |
| 540.70 | 778.06 | 1.4 | 1.7 | 11.6 | 89.1 | 39% \|± 9/2⟩ + 18% \|∓ 3/2⟩ + 17% \|∓ 11/2⟩ + 11% \|∓ 7/2⟩ +5% \|± 13/2⟩ | ±0.598 |
| 643.94 | 926.63 | 0.23 | 0.25 | 14.4 | 88.0 | 26% \|± 7/2⟩ + 16% \|∓ 9/2⟩ + 12% \|∓ 1/2⟩ + 10% \|∓ 5/2⟩ + 9% \|∓ 7/2⟩+ 8% \|± 9/2⟩ + 6% \|± 11/2⟩ + 5% \|± 1/2⟩ | ±0.209 |
| 786.91 | 1132.40 | 0.0053 | 0.0069 | 17.1 | 89.2 | 20% \|± 5/2⟩ + 17% \|∓ 5/2⟩ + 14% \|∓ 3/2⟩ + 13% \|± 3/2⟩ + 12% \|∓ 7/2⟩ + 11% \|± 7/2⟩ | ±0.076 |
| 1071.48 | 1541.85 | 0.0009 | 0.0015 | 19.8 | 89.7 | 23% \|± 1/2⟩ + 23% \|∓ 1/2⟩ + 16% \|± 3/2⟩ + 16% \|∓ 3/2⟩ + 8% \|± 5/2⟩ + 7% \|∓ 5/2⟩ | ±0.043 |

[a] The angle between the $g_z$ value of the excited Kramers doublet and the ground Kramers doublet.

**Table S43.** Electronic structure of **3-Dy** calculated with the crystal field parameters obtained from CASSCF-SO using the solid state geometry of **3-Dy·$C_6H_6$**. Each row corresponds to a Kramers doublet.

| Energy ($cm^{-1}$) | Energy (K) | $g_x$ | $g_y$ | $g_z$ | Angle[a] (deg) | Wavefunction | $\langle J_z \rangle$ |
|---|---|---|---|---|---|---|---|
| 0.00 | 0.00 | 0.0000 | 0.0000 | 19.88 | -- | 99.6% $\lvert \pm 15/2 \rangle$ | ±7.491 |
| 443.50 | 638.20 | 0.0001 | 0.0001 | 17.0 | 1.3 | 99.8% $\lvert \pm 13/2 \rangle$ | ±6.496 |
| 713.99 | 1027.43 | 0.0023 | 0.0027 | 14.4 | 1.6 | 99.1% $\lvert \pm 11/2 \rangle$ | ±5.504 |
| 895.46 | 1288.56 | 0.011 | 0.015 | 11.8 | 1.3 | 99.1% $\lvert \pm 9/2 \rangle$ | ±4.496 |
| 1053.69 | 1516.26 | 0.19 | 0.23 | 9.1 | 1.8 | 99.4% $\lvert \pm 7/2 \rangle$ | ±3.502 |
| 1192.79 | 1716.43 | 0.093 | 0.33 | 6.4 | 2.8 | 98% $\lvert \pm 5/2 \rangle$ | ±2.487 |
| 1289.35 | 1855.37 | 3.0 | 6.6 | 8.6 | 88.7 | 85% $\lvert \pm 3/2 \rangle$ + 14% $\lvert \mp 1/2 \rangle$ | ±1.208 |
| 1381.12 | 1987.44 | 0.53 | 2.0 | 17.1 | 89.6 | 84% $\lvert \pm 1/2 \rangle$ + 14% $\lvert \mp 3/2 \rangle$ | ±0.230 |

[a] The angle between the $g_z$ value of the excited Kramers doublet and the ground Kramers doublet.

**Table S44.** Electronic structure of **4-Dy** calculated with the crystal field parameters obtained from CASSCF-SO using the solid state geometry of **4-Dy**. Each row corresponds to a Kramers doublet.

| Energy ($cm^{-1}$) | Energy (K) | $g_x$ | $g_y$ | $g_z$ | Angle[a] (deg) | Wavefunction | $<J_z>$ |
|---|---|---|---|---|---|---|---|
| 0.00 | 0.00 | 0.0000 | 0.0000 | 19.87 | -- | 99.4% \|± 15/2⟩ | ±7.489 |
| 430.20 | 619.05 | 0.0003 | 0.0003 | 17.0 | 0.4 | 99.6% \|± 13/2⟩ | ±6.492 |
| 702.97 | 1011.57 | 0.0030 | 0.0035 | 14.4 | 0.6 | 99.3% \|± 11/2⟩ | ±5.508 |
| 887.75 | 1277.47 | 0.029 | 0.036 | 11.8 | 0.4 | 99.4% \|± 9/2⟩ | ±4.502 |
| 1041.49 | 1498.70 | 0.45 | 0.49 | 9.1 | 0.4 | 98.8% \|± 7/2⟩ | ±3.479 |
| 1168.13 | 1680.95 | 2.4 | 3.3 | 6.0 | 1.3 | 91% \|± 5/2⟩ + 7% \|± 1/2⟩ | ±2.309 |
| 1253.88 | 1804.33 | 2.3 | 6.8 | 10.4 | 89.3 | 73% \|± 3/2⟩ + 21% \|∓ 1/2⟩ + 5% \|∓ 5/2⟩ | ±0.900 |
| 1385.91 | 1994.32 | 0.16 | 0.46 | 18.4 | 89.7 | 69% \|± 1/2⟩ + 23% \|∓ 3/2⟩ | ±0.079 |

[a] The angle between the $g_z$ value of the excited Kramers doublet and the ground Kramers doublet.

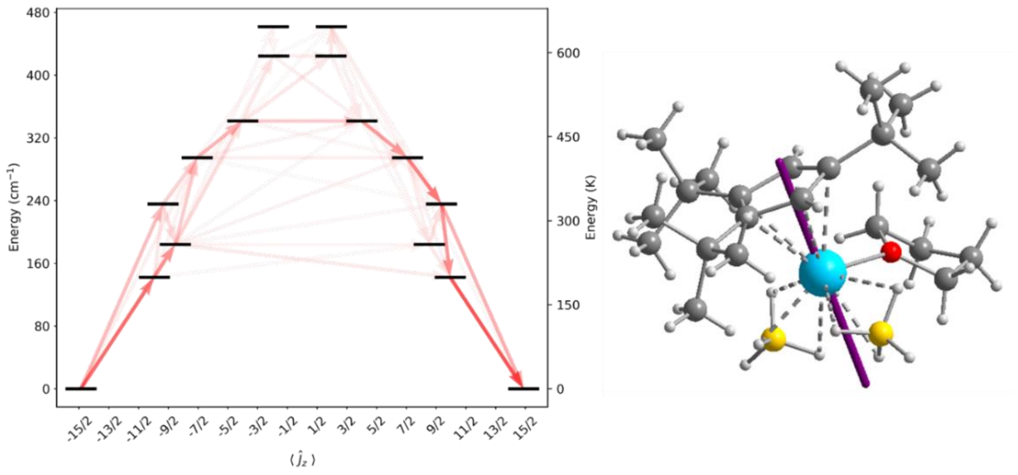


**Figure S165.** Energy barrier to magnetic relaxation for a model of **1-Dy**. Electronic states from CASSCF-SO calculations, labelled with their dominant $m_J$ composition in the $J$ = 15/2 basis. Arrows represent the Orbach relaxation pathway, where the opacity of the arrows is proportional to the transition probability approximated with the average matrix elements of magnetic moment connecting the states, $\gamma_{ij} = (1/3)[|\langle i|\mu_x|j\rangle|^2 + |\langle i|\mu_y|j\rangle|^2 + |\langle i|\mu_z|j\rangle|^2]$, normalized from each departing state and commencing from |–15/2> (left). Denotation of the $g_z$ axis (purple) within the solid state structure at the ground state (right).

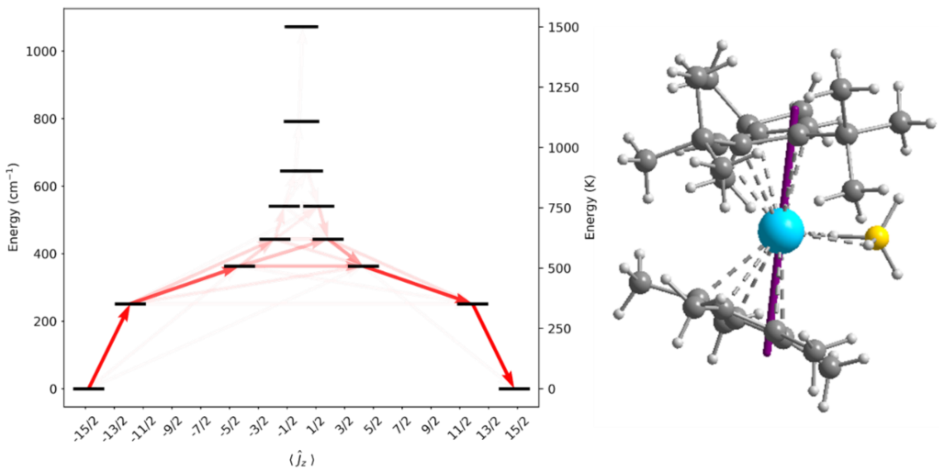


**Figure S166.** Energy barrier to magnetic relaxation for a model of **2-Dy**. Electronic states from CASSCF-SO calculations, labelled with their dominant $m_J$ composition in the $J$ = 15/2 basis. Arrows represent the Orbach relaxation pathway, where the opacity of the arrows is proportional to the transition probability approximated with the average matrix elements of magnetic moment connecting the states, $\gamma_{ij} = (1/3)[|<i|\mu_x|j>|^2 + |<i|\mu_y|j>|^2 + |<i|\mu_z|j>|^2]$, normalized from each departing state and commencing from |–15/2> (left). Denotation of the $g_z$ axis (purple) within the solid state structure at the ground state (right).

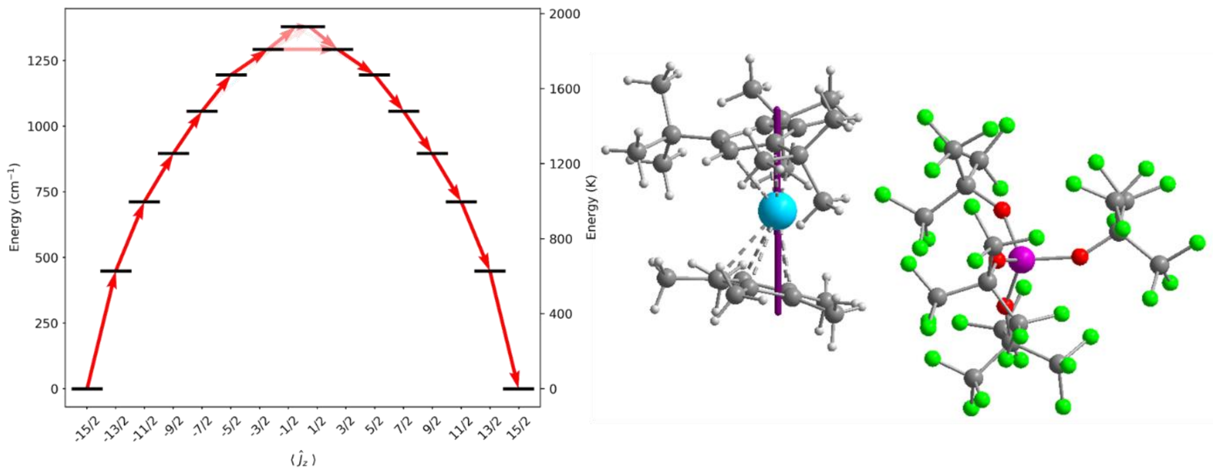


**Figure S167.** Energy barrier to magnetic relaxation for a model of **3-Dy**. Electronic states from CASSCF-SO calculations, labelled with their dominant $m_J$ composition in the $J$ = 15/2 basis. Arrows represent the Orbach relaxation pathway, where the opacity of the arrows is proportional to the transition probability approximated with the average matrix elements of magnetic moment connecting the states, $\gamma_{ij} = (1/3)[|\langle i|\mu_x|j\rangle|^2 + |\langle i|\mu_y|j\rangle|^2 + |\langle i|\mu_z|j\rangle|^2]$, normalized from each departing state and commencing from $|-15/2\rangle$ (left). Denotation of the $g_z$ axis (purple) within the solid state structure at the ground state (right).

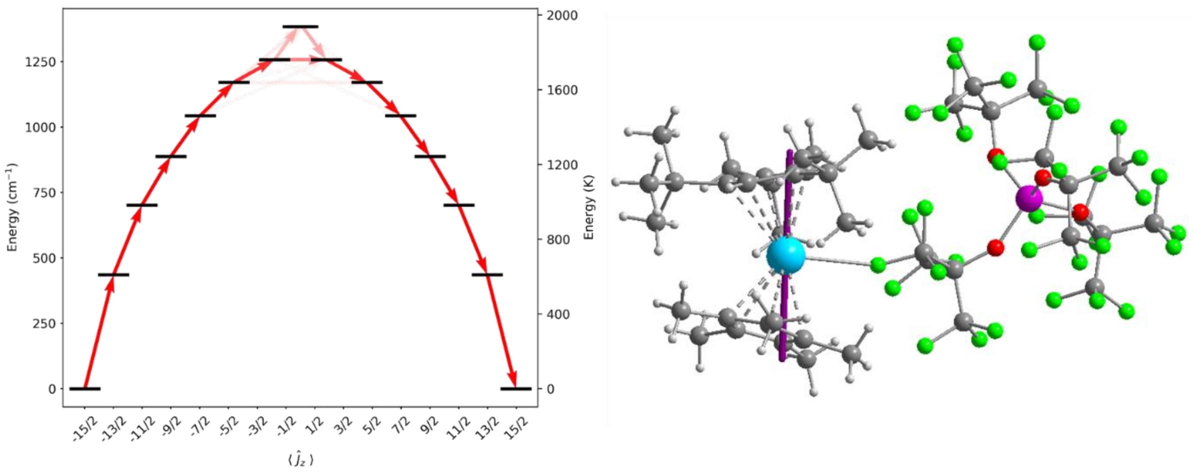


**Figure S168.** Energy barrier to magnetic relaxation for a model of **4-Dy**. Electronic states from CASSCF-SO calculations, labelled with their dominant $m_J$ composition in the $J$ = 15/2 basis. Arrows represent the Orbach relaxation pathway, where the opacity of the arrows is proportional to the transition probability approximated with the average matrix elements of magnetic moment connecting the states, $\gamma_{ij} = (1/3)[|<i|\mu_x|j>|^2 + |<i|\mu_y|j>|^2 + |<i|\mu_z|j>|^2]$, normalized from each departing state and commencing from |–15/2> (left). Denotation of the $g_z$ axis (purple) within the solid state structure at the ground state (right).

## 10. References


(1) Pedregosa, F.; Varoquaux, G.; Gramfort, A.; Michel, V.; Thirion, B.; Grisel, O.; Blondel, M.; Müller, A.; Nothman, J.; Louppe, G.; Prettenhofer, P.; Weiss, R.; Dubourg, V.; Vanderplas, J.; Passos, A.; Cournapeau, D.; Brucher, M.; Perrot, M.; Duchesnay, É. Scikit-Learn: Machine Learning in Python. *J. Mach. Learn. Res.* **2011**, *12*, 2825–2830. https://doi.org/10.5555/1953048.2078195.

(2) Gransbury, G. K.; Kragskow, J. G. C.; Chilton, N. F. *AtomAccess Manual* https://chilton-group.gitlab.io/atom_access/index.html (accessed 2023-02-22).

(3) Guo, F.-S.; He, M.; Huang, G.-Z.; Giblin, S. R.; Billington, D.; Heinemann, F. W.; Tong, M.-L.; Mansikkamäki, A.; Layfield, R. A. Discovery of a Dysprosium Metallocene Single-Molecule Magnet with Two High-Temperature Orbach Processes. *Inorg. Chem.* **2022**, *61* (16), 6017–6025. https://doi.org/10.1021/acs.inorgchem.1c03980.

(4) Groom, C. R.; Bruno, I. J.; Lightfoot, M. P.; Ward, S. C. The Cambridge Structural Database. *Acta Crystallogr. Sect. B Struct. Sci. Cryst. Eng. Mater.* **2016**, *72* (2), 171–179. https://doi.org/10.1107/S2052520616003954.

(5) Bruno, I. J.; Cole, J. C.; Edgington, P. R.; Kessler, M.; Macrae, C. F.; McCabe, P.; Pearson, J.; Taylor, R. New Software for Searching the Cambridge Structural Database and Visualizing Crystal Structures. *Acta Crystallogr. Sect. B Struct. Sci.* **2002**, *58* (3), 389–397. https://doi.org/10.1107/S0108768102003324.

(6) Corner, S. C.; Goodwin, C. A. P.; Ortu, F.; Evans, P.; Zhang, H.; Gransbury, G. K.; Whitehead, G. F. S.; Mills, D. P. Synthesis of Heteroleptic Yttrium and Dysprosium 1,2,4-Tris(Trimethylsilyl)Cyclopentadienyl Complexes. *Aust. J. Chem.* **2022**, *75*, 684–697. https://doi.org/10.1071/ch21314.

(7) O'Boyle, N. M.; Banck, M.; James, C. A.; Morley, C.; Vandermeersch, T.; Hutchison, G. R. Open Babel: An Open Chemical Toolbox. *J. Cheminform.* **2011**, *3* (1), 1–33.

https://doi.org/10.1186/1758-2946-3-33.

(8) OpenBabel. *Generate multiple conformers*. [Search Algorithm] https://open-babel.readthedocs.io/en/latest/3DStructureGen/multipleconformers.html#genetic-algorithm (accessed 2020-05-20).

(9) *GaussView*; Dennington, R.; Keith, T. A.; Millam, J. M. Semichem Inc: Shawnee Mission, KS, USA, 2016.

(10) Ortu, F.; Packer, D.; Liu, J.; Burton, M.; Formanuik, A.; Mills, D. P. Synthesis and Structural Characterization of Lanthanum and Cerium Substituted Cyclopentadienyl Borohydride Complexes. *J. Organomet. Chem.* **2018**, *857*, 45–51. https://doi.org/10.1016/j.jorganchem.2017.09.010.

(11) Jaroschik, F.; Nief, F.; Le Goff, X.-F. F.; Ricard, L. Synthesis and Reactivity of Organometallic Complexes of Divalent Thulium with Cyclopentadienyl and Phospholyl Ligands. *Organometallics* **2007**, *26* (14), 3552–3558. https://doi.org/10.1021/om700316a.

(12) Rabe, G.; Roesky, H. W.; Stalke, D.; Pauer, F.; Sheldrick, G. M. The Preparation and Crystal Structures of Sodium and Potassium Pentamethylcyclopentadienyl Pyridine Solvates. *J. Organomet. Chem.* **1991**, *403* (1–2), 11–19. https://doi.org/10.1016/0022-328X(91)83082-F.

(13) Krossing, I.; Brands, H.; Feuerhake, R.; Koenig, S. New Reagents to Introduce Weakly Coordinating Anions of Type $Al(OR_F)_4^-$: Synthesis, Structure and Characterization of Cs and Trityl Salts. *J. Fluor. Chem.* **2001**, *112* (1), 83–90. https://doi.org/10.1016/S0022-1139(01)00490-0.

(14) Stoyanov, E. S.; Stoyanova, I. V.; Reed, C. A. The Basicity of Unsaturated Hydrocarbons as Probed by Hydrogen-Bond-Acceptor Ability: Bifurcated $N\text{-}H^+\cdots\pi$ Hydrogen Bonding. *Chem. Eur. J.* **2008**, *14* (26), 7880–7891.

https://doi.org/10.1002/chem.200800337.

(15) Lambert, J. B.; Zhang, S.; Ciro, S. M. Silyl Cations in the Solid and in Solution. *Organometallics* **1994**, *13* (6), 2430–2443. https://doi.org/10.1021/om00018a041.

(16) Chien, J. C. W.; Tsai, W. M.; Rausch, M. D. Isospecific Polymerization of Propylene Catalyzed by Rac-Ethylenebis(Indenyl)Methylzirconium Cation. *J. Am. Chem. Soc.* **1991**, *113* (22), 8570–8571. https://doi.org/10.1021/ja00022a081.

(17) Gabbaï, F. P.; Chirik, P. J.; Fogg, D. E.; Meyer, K.; Mindiola, D. J.; Schafer, L. L.; You, S.-L. An Editorial About Elemental Analysis. *Organometallics* **2016**, *35* (19), 3255–3256. https://doi.org/10.1021/acs.organomet.6b00720.

(18) Fadeeva, V. P.; Tikhova, V. D.; Nikulicheva, O. N. Elemental Analysis of Organic Compounds with the Use of Automated CHNS Analyzers. *J. Anal. Chem.* **2008**, *63* (11), 1094–1106. https://doi.org/10.1134/S1061934808110142.

(19) Kabova, E. A.; Blundell, C. D.; Muryn, C. A.; Whitehead, G. F. S.; Vitorica-Yrezabal, I. J.; Ross, M. J.; Shankland, K. SDPD-SX: Combining a Single Crystal X-Ray Diffraction Setup with Advanced Powder Data Structure Determination for Use in Early Stage Drug Discovery. *CrystEngComm* **2022**, *24* (24), 4337–4340. https://doi.org/10.1039/D2CE00387B.

(20) *CrysAlis PRO*. Agilent Technologies Ltd: Yarnton, Oxfordfordshire, England 2014.

(21) Pawley, G. S. Unit-Cell Refinement from Powder Diffraction Scans. *J. Appl. Crystallogr.* **1981**, *14* (6), 357–361. https://doi.org/10.1107/S0021889881009618.

(22) Coelho, A. A. An Indexing Algorithm Independent of Peak Position Extraction for X-Ray Powder Diffraction Patterns. *J. Appl. Crystallogr.* **2017**, *50* (5), 1323–1330. https://doi.org/10.1107/S1600576717011359.

(23) Petříček, V.; Dušek, M.; Palatinus, L. Crystallographic Computing System JANA2006: General Features. *Z. Kristallogr. - Cryst. Mater.* **2014**, *229* (5), 345–352.

https://doi.org/10.1515/zkri-2014-1737.

(24) Sheldrick, G. M. A Short History of *SHELX*. *Acta Crystallogr. Sect. A Found. Crystallogr.* **2008**, *64* (1), 112–122. https://doi.org/10.1107/S0108767307043930.

(25) Sheldrick, G. M. Crystal Structure Refinement with *SHELXL*. *Acta Crystallogr. Sect. C Struct. Chem.* **2015**, *71* (1), 3–8. https://doi.org/10.1107/S2053229614024218.

(26) Dolomanov, O. V.; Bourhis, L. J.; Gildea, R. J.; Howard, J. A. K.; Puschmann, H. OLEX2 : A Complete Structure Solution, Refinement and Analysis Program. *J. Appl. Crystallogr.* **2009**, *42* (2), 339–341. https://doi.org/10.1107/S0021889808042726.

(27) Farrugia, L. J. WinGX and ORTEP for Windows : An Update. *J. Appl. Crystallogr.* **2012**, *45* (4), 849–854. https://doi.org/10.1107/S0021889812029111.

(28) *Persistence of Vision Raytracer*. Persistence of Vision Raytracer Pty. Ltd., v.3.7, 2013. Retrieved from http://www.povray.org/download/.

(29) Adamo, C.; Barone, V. Toward Reliable Density Functional Methods without Adjustable Parameters: The PBE0 Model. *J. Chem. Phys.* **1999**, *110* (13), 6158–6170. https://doi.org/10.1063/1.478522.

(30) Perdew, J. P.; Ernzerhof, M.; Burke, K. Rationale for Mixing Exact Exchange with Density Functional Approximations. *J. Chem. Phys.* **1996**, *105* (22), 9982–9985. https://doi.org/10.1063/1.472933.

(31) Grimme, S.; Antony, J.; Ehrlich, S.; Krieg, H. A Consistent and Accurate Ab Initio Parametrization of Density Functional Dispersion Correction (DFT-D) for the 94 Elements H-Pu. *J. Chem. Phys.* **2010**, *132* (15), 154104. https://doi.org/10.1063/1.3382344.

(32) Dunning, T. H. Gaussian Basis Sets for Use in Correlated Molecular Calculations. I. The Atoms Boron through Neon and Hydrogen. *J. Chem. Phys.* **1989**, *90* (2), 1007–1023. https://doi.org/10.1063/1.456153.

(33) Andrae, D.; Häußermann, U.; Dolg, M.; Stoll, H.; Preuß, H. Energy-Adjusted *Ab Initio* Pseudopotentials for the Second and Third Row Transition Elements. *Theor. Chim. Acta* **1990**, *77* (2), 123–141. https://doi.org/10.1007/BF01114537.

(34) Martin, J. M. L.; Sundermann, A. Correlation Consistent Valence Basis Sets for Use with the Stuttgart–Dresden–Bonn Relativistic Effective Core Potentials: The Atoms Ga–Kr and In–Xe. *J. Chem. Phys.* **2001**, *114* (8), 3408–3420. https://doi.org/10.1063/1.1337864.

(35) Frisch, M. J.; Trucks, G. W.; Schlegel, H. B.; Scuseria, G. E.; Robb, M. A.; Cheeseman, J. R.; Scalmani, G.; Barone, V.; Petersson, G. A.; Nakatsuji, H.; Li, X.; Caricato, M.; Marenich, A. V.; Bloino, J.; Janesko, B. G.; Gomperts, R.; Mennucci, B.; Hratchian, H. P.; Ortiz, J. V.; Izmaylov, A. F.; Sonnenberg, J. L.; Williams-Young, D.; Ding, F.; Lipparini, F.; Egidi, F.; Goings, J.; Peng, B.; Petrone, A.; Henderson, T.; Ranasinghe, D.; Zakrzewski, V. G.; Gao, J.; Rega, N.; Zheng, G.; Liang, W.; Hada, M.; Ehara, M.; Toyota, K.; Fukuda, R.; Hasegawa, J.; Ishida, M.; Nakajima, T.; Honda, Y.; Kitao, O.; Nakai, H.; Vreven, T.; Throssell, K.; Montgomery, Jr., J. A.; Peralta, J. E.; Ogliaro, F.; Bearpark, M. J.; Heyd, J. J.; Brothers, E. N.; Kudin, K. N.; Staroverov, V. N.; Keith, T. A.; Kobayashi, R.; Normand, J.; Raghavachari, K.; Rendell, A. P.; Burant, J. C.; Iyengar, S. S.; Tomasi, J.; Cossi, M.; Millam, J. M.; Klene, M.; Adamo, C.; Cammi, R.; Ochterski, J. W.; Martin, R. L.; Morokuma, K.; Farkas, O.; Foresman, J. B.; Fox, D. J. Gaussian 16. Gaussian, Inc.: Wallingford CT 2016.

(36) Lu, T.; Chen, F. Multiwfn: A Multifunctional Wavefunction Analyzer. *J. Comput. Chem.* **2012**, *33* (5), 580–592. https://doi.org/10.1002/jcc.22885.

(37) Bain, G. A.; Berry, J. F. Diamagnetic Corrections and Pascal's Constants. *J. Chem. Educ.* **2008**, *85* (4), 532. https://doi.org/10.1021/ed085p532.

(38) Reta, D.; Chilton, N. F. Uncertainty Estimates for Magnetic Relaxation Times and

Magnetic Relaxation Parameters. *Phys. Chem. Chem. Phys.* **2019**, *21* (42), 23567–23575. https://doi.org/10.1039/C9CP04301B.

(39) Blackmore, W. J. A.; Gransbury, G. K.; Evans, P.; Kragskow, J. G. C.; Mills, D. P.; Chilton, N. F. Characterisation of Magnetic Relaxation on Extremely Long Timescales. *Phys. Chem. Chem. Phys.* **2023**, *25* (25), 16735–16744. https://doi.org/10.1039/D3CP01278F.

(40) Hilgar, J. D.; Butts, A. K.; Rinehart, J. D. A Method for Extending AC Susceptometry to Long-Timescale Magnetic Relaxation. *Phys. Chem. Chem. Phys.* **2019**, *21* (40), 22302–22307. https://doi.org/10.1039/C9CP03936H.

(41) Topping, C. V; Blundell, S. J. A.C. Susceptibility as a Probe of Low-Frequency Magnetic Dynamics. *J. Phys. Condens. Matter* **2019**, *31* (1), 013001. https://doi.org/10.1088/1361-648X/aaed96.

(42) *OriginPro*; OriginLab Corporation: Northampton, England, 2022.

(43) Zorn, R. Logarithmic Moments of Relaxation Time Distributions. *J. Chem. Phys.* **2002**, *116* (8), 3204–3209. https://doi.org/10.1063/1.1446035.

(44) Fdez. Galván, I.; Vacher, M.; Alavi, A.; Angeli, C.; Aquilante, F.; Autschbach, J.; Bao, J. J.; Bokarev, S. I.; Bogdanov, N. A.; Carlson, R. K.; Chibotaru, L. F.; Creutzberg, J.; Dattani, N.; Delcey, M. G.; Dong, S. S.; Dreuw, A.; Freitag, L.; Frutos, L. M.; Gagliardi, L.; Gendron, F.; Giussani, A.; González, L.; Grell, G.; Guo, M.; Hoyer, C. E.; Johansson, M.; Keller, S.; Knecht, S.; Kovačević, G.; Källman, E.; Li Manni, G.; Lundberg, M.; Ma, Y.; Mai, S.; Malhado, J. P.; Malmqvist, P. Å.; Marquetand, P.; Mewes, S. A.; Norell, J.; Olivucci, M.; Oppel, M.; Phung, Q. M.; Pierloot, K.; Plasser, F.; Reiher, M.; Sand, A. M.; Schapiro, I.; Sharma, P.; Stein, C. J.; Sørensen, L. K.; Truhlar, D. G.; Ugandi, M.; Ungur, L.; Valentini, A.; Vancoillie, S.; Veryazov, V.; Weser, O.; Wesołowski, T. A.; Widmark, P. O.; Wouters, S.; Zech, A.; Zobel, J. P.; Lindh, R. OpenMolcas: From

Source Code to Insight. *J. Chem. Theory Comput.* **2019**, *15* (11), 5925–5964. https://doi.org/10.1021/acs.jctc.9b00532.

(45) Roos, B. O.; Veryazov, V.; Widmark, P.-O. Relativistic Atomic Natural Orbital Type Basis Sets for the Alkaline and Alkaline-Earth Atoms Applied to the Ground-State Potentials for the Corresponding Dimers. *Theor. Chem. Acc.* **2004**, *111* (2–6), 345–351. https://doi.org/10.1007/s00214-003-0537-0.

(46) Roos, B. O.; Lindh, R.; Malmqvist, P. Å.; Veryazov, V.; Widmark, P. O. Main Group Atoms and Dimers Studied with a New Relativistic ANO Basis Set. *J. Phys. Chem. A* **2004**, *108* (15), 2851–2858. https://doi.org/10.1021/jp031064+.

(47) Roos, B. O.; Lindh, R.; Malmqvist, P.-Å.; Veryazov, V.; Widmark, P.-O. New Relativistic ANO Basis Sets for Transition Metal Atoms. *J. Phys. Chem. A* **2005**, *109* (29), 6575–6579. https://doi.org/10.1021/jp0581126.

(48) Roos, B. O.; Lindh, R.; Malmqvist, P.-Å.; Veryazov, V.; Widmark, P.-O.; Borin, A. C. New Relativistic Atomic Natural Orbital Basis Sets for Lanthanide Atoms with Applications to the Ce Diatom and $LuF_3$. *J. Phys. Chem. A* **2008**, *112* (45), 11431–11435. https://doi.org/10.1021/jp803213j.

(49) Chibotaru, L. F.; Ungur, L. Ab Initio Calculation of Anisotropic Magnetic Properties of Complexes. I. Unique Definition of Pseudospin Hamiltonians and Their Derivation. *J. Chem. Phys.* **2012**, *137* (6), 064112. https://doi.org/10.1063/1.4739763.

(50) *Diamond-Crystal and Molecular Structure Visualization, v.4.6.6.*; Putz, H.; Brandenburg, K.; Crystal Impact GbR, Bonn, Germany, 2021.
